# Integrated Analyses of lncRNA and mRNA Profiles Reveal Characteristic and Functional Changes of Leukocytes in Qi-Deficiency Constitution and Pi-Qi-Deficiency Syndrome of Chronic Superficial Gastritis

**DOI:** 10.1155/2020/8518053

**Published:** 2020-07-16

**Authors:** Leiming You, Aijie Liu, Xiaopu Sang, Xinhui Gao, Ting'An Li, Shen Zhang, Kunyu Li, Wei Wang, Guangrui Huang, Ting Wang, Anlong Xu

**Affiliations:** ^1^School of Life Sciences, Beijing University of Chinese Medicine, Beijing 100029, China; ^2^State Key Laboratory of Biocontrol, Guangdong Province Key Laboratory of Pharmaceutical Functional Genes, School of Life Sciences, Sun Yat-sen University, Higher Education Mega Center, Guangzhou 510006, China

## Abstract

**Methods:**

We adopted RNA-sequencing approach to identify differential lncRNAs and genes in leukocytes, clustered expression profiles, and analyzed biological functions and pathways of differential genes to decode their potential roles in contributing to characteristics and functions of leukocytes. In addition, interaction networks were created to detail the interactions between differential genes. In particular, we explored differential lncRNAs-mediated regulation of differential genes and predicted the subcellular location of lncRNAs to reveal their potential roles.

**Results:**

Compared with TCM-defined balanced constitution (BC), 183 and 93 genes as well as 749 and 651 lncRNAs were differentially expressed (*P* < 0.05 and |log_2_ (fold change)| ≥1) in leukocytes of individuals from case populations 1 (QDC) and 2 (PQDS), respectively. Of them, 12 genes and 111 lncRNAs were common to each case population. Several networks were created to detail the interactions among case-specific genes, especially case-specific lncRNAs-mediated regulation of case-specific genes. Also, interaction networks were created for the common lncRNAs and genes. HCL analyses showed that differential genes and lncRNAs, especially the common genes and lncRNAs, kept similar expression patterns in both case populations. Furthermore, function enrichment analyses just indicated the common biological processes, namely, extracellular matrix organization and cell adhesion via plasma membrane adhesion molecules. In addition, most common genes underwent very tight and complex regulation of many *trans*- and *cis*-acting lncRNAs. In particular, of them, ADAMTSL5, COL26A1, COL27A1, MSH5, and LOC390937 could be regulated by multiple case-specific and common lncRNAs, including the means that directs binding of the common lncRNAs to their coded proteins. The common changes in the extracellular matrix and integral components of plasma membrane related to cell-cell adhesion/junction and communication may implicate the linkage between QDC and PQDS, contributing to alterations in characteristics and functions of leukocytes.

**Conclusions:**

These results may provide new insights into the characteristic and functional changes of leukocytes in QDC and PQDS, especially the mechanism underlying the linkage of QDC to PQDS, with potential leukocytes biomarkers for future application in integrative medicine.

## 1. Introduction

Traditional Chinese medicine (TCM) is an ancient medical practice system with the longest history in Asia [1]. Qi, pronounced “chee,” means energy in TCM. It is spelled “Chi” or even “Ki” in Japanese, but they all carry the same meaning. Qi is the energy of the body, of the meridians. TCM-defined constitution is innate and relatively stable, relying on the intrinsic characteristics of body. It can be influenced by postnatal environment and defined by integrating the morphological structure and physiological function with psychological state [2–5]. Qi-deficiency constitution (QDC) is one of nine typical body constitutions defined in TCM [3, 4]. Following TCM theories, especially the clinical data, individuals with QDC seem to have a tendency toward the Pi-qi-deficiency syndrome (PQDS), one of the commonly matched TCM syndromes in patients with chronic superficial gastritis (CSG) [6–8]. Also, individuals with the QDC or PQDS share very similar features including lacking vitality (Qi) and getting sick easily, being characterized by languid lazy words, low voice, pale tongue, and weak pulse [4, 8]. These phenomena together could suggest that there is a possible linkage between QDC and PQDS. However, little is known about the underlying molecular characteristics of QDC and PQDS, particularly the potential biomarkers implicated in linking QDC to PQDS of CSG. In recent years, advances in next-generation sequencing (NGS) technology enable the multiomics analysis of human diseases, especially the discovery of disease-related biomarkers [9–11]. Long noncoding RNAs (lncRNAs), a type of noncoding RNAs (ncRNAs) longer than 200 nucleotides, have been reported to be involved in multiple pathological processes such as inflammation, carcinogenesis, and tumor progression [12–15].

Thus, in this study, the popular RNA-sequencing (RNA-seq) approach was adopted to identify differentially expressed lncRNAs and genes in the blood leukocytes of individuals from different populations, including the control population (balanced constitution, BC), case population 1 (QDC), and case population 2 (PQDS of CSG). The specific lncRNAs and genes identified in a certain case population are probably case-specific, and the common lncRNAs and genes may be the potential candidate biomarkers involved in linking QDC to PQDS. Enrichment analyses, including GO, pathway, disease, and domain, were performed on the common genes and the case-specific genes to explore their potential roles in contributing to characteristics and functions of leukocytes. Also, interactions network analyses were done for the case-specific genes to detail the physical and functional protein-protein associations. We analyzed the lncRNA-mediated *trans*- and *cis-*regulation of gene expression for each case population. In particular, we detailed lncRNA-mediated regulation of candidate biomarkers, including direct binding of lncRNAs to mRNAs and mRNAs-corresponding proteins. We also analyzed the subcellular location of common lncRNAs regulating these biomarker genes and predicted the RNA-binding proteins (RBPs) for the exosome-contained lncRNAs to reveal their potential roles when traveling throughout the body.

## 2. Materials and Methods

### 2.1. Ethics Approval

The study was registered at *ClinicalTrials.gov* (identifier: NCT02915393). The protocol was approved (JDF-IRB-2016031002) by the Institutional Review Board of Dongfang Hospital affiliated to Beijing University of Chinese Medicine. All the methods were performed in accordance with the relevant guidelines and regulations. Participants were informed of the purpose, general contents, and data use of the study, and they all signed the informed consent.

### 2.2. Participants and Experimental Design

All the subjects (detailed in Supplemental Table S1) were recruited at the hepatobiliary and gastroenterological outpatient's department, Dongfang Hospital affiliated to Beijing University of Chinese Medicine. According to the *Constitution of TCM Classification and Judgment* published by China Association of Chinese Medicine in 2009 [16], individuals with various TCM constitutions (BC and QDC) were identified and recruited. CSG patients with PQDS were diagnosed and recruited, based on the *Guiding Principle for Clinical Research on New Drugs of TCM* published in 2002 [17] and the CSG pathological diagnosis and grading standards which were proposed on the *China Chronic Gastritis Consensus* in Shanghai, 2012 [18]. The experimental design (Supplemental Figure S1), including the diagnosis, inclusion, and exclusion criteria for all the subjects in this study, is detailed in the supplemental information (Supplemental Methods).

### 2.3. Leukocytes

Blood samples (5 mL) were collected in the additive-free blood collection tubes, and the leukocytes were isolated using the lymphocyte separation reagent (Solarbio) according to the manufacture's instruction.

### 2.4. RNA Sequencing

RNA sequencing was performed by OEbiotech Company (Shanghai, China). Briefly, total RNAs of leukocytes were extracted using the RNA isolation kit (Ambion) following the manufacturer's instructions and stored at −80°C. RNA integrity number (RIN) was detected using the popular Agilent 2100 Bioanalyzer. RNA samples (RIN ≥7) were subjected to the subsequent high-throughput sequencing analysis. All the transcriptomic sequencing libraries were constructed using the commercial kit (Illumina, RS-122-2301) termed “TruSeq Stranded Total RNA with Ribo-Zero Globin” (this kit keeps an efficient work flow enabling removal of ribosomal RNA and globin mRNA in a single step). These resultant sequencing libraries were sequenced by the well-known Illumina sequencing platform (HiSeqTM 2500), and 150 bp/125 bp paired-end raw reads were generated. The generated raw reads were filtered, mapped to the human reference genome (GRCh38.p7) (Supplemental Table S2), and processed for the subsequent annotation and differential expression analyses of lncRNAs and mRNAs.

### 2.5. Identifying Differentially Expressed lncRNAs and Genes

The expression levels of lncRNAs and mRNAs, including the novel lncRNAs discovered in this study, were standardized and indicated using FPKM (fragments per kilobase of exon model per million mapped reads). The identification of lncRNAs and genes between groups and the *P* value calculations were performed using the *R* package of DESeq [19]. The differential lncRNAs and genes among groups were filtered (*P* value <0.05 and |log_2_ (fold change) ≥1|). Hierarchical clustering (HCL) analyses of differential lncRNAs and genes were performed by Cluster v3.0, and TreeView package v1.1.6 was used to browse and draw HCL analysis-based heatmaps [20].

### 2.6. GO, Pathway, and Disease-Based Enrichment Analyses of Differential Genes

The GO-based functional enrichment analyses of differential genes were performed by the popular DAVID tool v6.8 (a database for annotation, visualization, and integrated discovery of gene sets) [21]. The pathway enrichment analyses of differential genes were conducted using the well-updated KOBAS v3.0 (a web server for identification of statistically significantly enriched pathways, human diseases, and functional terms for an input set of genes), integrating the annotation information from multiple well-known databases covering pathways (KEGG PATHWAY, Panther, Reactome) and diseases (KEGG DISEASE, OMIM, NHGRI GWAS Catalog) [22]. Also, using the KOBAS server, the disease enrichment analyses of differential genes were performed.

### 2.7. Gene Set Enrichment Analyses of Genome-Wide Expression Profile

The GO, KEGG, and Reactome-based gene set enrichment analyses (GSEA), a knowledge-based approach for interpreting genome-wide expression profiles [23], were performed for the obtained gene expression data. The published R/Bioconductor packages were used to directly identify the statistically significantly enriched GO and pathway terms of all the detected genes in each case population [24, 25].

### 2.8. Interaction Network and Domain Enrichment Analyses of Differential Genes

The genes were listed by names (or UniProt protein ids) to be input into the online STRING database v11 (addressed at http://string-db.org) [26], for the corresponding protein-protein network analysis containing the direct (physical) and indirect (functional) protein-protein associations. The popular Cytoscape software (Windows, v3.7.2) [27] was used to visualize the obtained complex networks integrated with any type of attribute data. Also, in particular, the functional enrichment in the generated interaction network was listed, including the enrichment related to the “InterPro protein domains and features” that were defined and detailed in the InterPro database [28].

### 2.9. Predicting Targets of *trans*- and *cis*-Acting lncRNA

We first identified all the coexpression pairs of lncRNAs and genes in each case population by calculating the Pearson's correlation coefficients (|r| >0.8 and *P* value <0.05) between the expression levels of differential lncRNAs and protein-coding transcripts. Then, two independent algorithms were adopted to predict target genes for *cis*- or *trans*-acting lncRNAs. *cis*-Acting lncRNAs usually control their neighboring genes [29, 30], so, for each coexpression pair (|r| >0.8) of gene and lncRNA, if the gene is close (less than 100 kb) to the lncRNA in a genome, it was considered as a candidate target of the lncRNA. However, *trans*-acting lncRNA and its possible target genes are located in different chromosomes, and especially lncRNA is capable of binding to mRNA. Thus, we first eliminated the coexpression pairs (*r* >0.8) which have lncRNAs and genes encoded in the same chromosome. Further, using the software “RIsearch” v2.0 (a large scale RNA-RNA interaction prediction tool) [31], we evaluated the binding ability of lncRNA and mRNA for each obtained coexpression pair, setting parameters as hybridization sites length >20 nt, binding free energy <50.

### 2.10. Predicting Subcellular Location of lncRNA

The tool termed “lncLocator” (an ensemble classifier-based computational predictor) [32] was used to predict the subcellular localization for lncRNA, including cytoplasm, nucleus, cytosol, ribosome, and exosome. The default parameters were used to output the confident location prediction for a specific lncRNA.

### 2.11. Evaluation of lncRNA-Protein Interaction

The possible interactions of a lncRNA and multiple proteins were evaluated by the publically available RPISeq tool [33], a computational tool for investigating RNA-protein interaction partners. The probability control parameters were used, SVM (support vector machine) >0.5 and RF (random forest) >0.5. Accordingly, the predictions with probabilities >0.5 were considered “positive,” indicating that the corresponding RNA and protein are likely to interact with each other.

### 2.12. Predicting RNA-Binding Proteins (RBPs) of lncRNA

The lncRNAs were applied to analyze their possible binding proteins, using the web interface of RBPDB (v1.3.1) [34], an RNA-binding protein database collecting the experimental observations of RNA-binding sites, both in vitro and in vivo. The threshold was set to 0.8 (match scores that are greater than 80% of the maximum score for that PWM).

## 3. Results

### 3.1. Differential lncRNAs and Genes in Leukocytes

**Figure 1:**
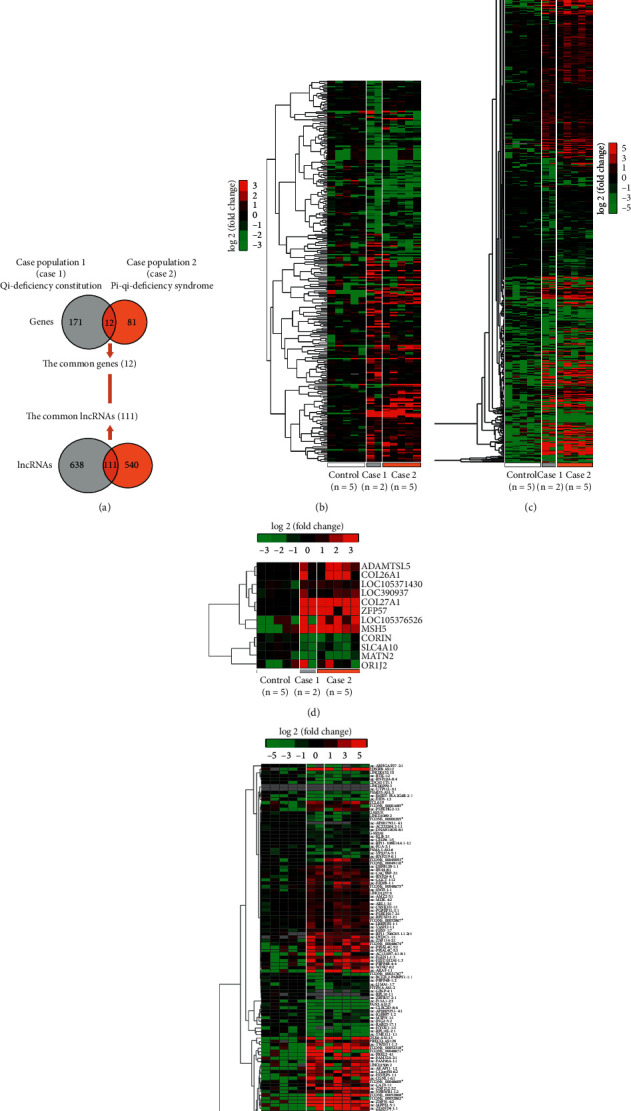
Expression profile analyses of differential genes and lncRNAs in leukocytes of individuals from different populations. (a) Venn diagram of the differential genes and lncRNAs identified in the two case populations. (b) Hierarchical clustering (HCL) analysis for expression profiles of all the differential genes. (c) HCL analysis for expression profiles of all the differential lncRNAs. (d) HCL heatmap generated for expression patterns of the common differential genes. (e) HCL analysis for expression patterns of the common differential lncRNAs. Control: balanced constitution; case 1: qi-deficiency constitution; case 2: chronic superficial gastritis patients with Pi-qi-deficiency syndrome.

Compared with BC (control population, *n* = 5), 183 and 93 differential genes (Supplemental Tables S3 and 4) as well as 749 and 651 differential lncRNAs (Supplemental Tables S5 and 6) were discovered in the QDC (case population 1, *n* = 2) and the CSG patients with PQDS (case population 2, *n* = 5), respectively. In particular, 12 common genes and 111 common lncRNAs were found in the case populations (Figure 1(a)), considered as the leukocyte candidate biomarkers implicated in QDC and PQDS of CSG. We performed HCL analyses of expression profiles of differential genes and lncRNAs in leukocytes from the three populations. HCL heatmaps showed that the expression patterns of the common differential genes and lncRNAs of each person in both case populations were so similar (Figures 1(d) and 1(e)), obviously distinguished from the control population (BC). Also, for all the identified differential genes and lncRNAs, similar expression profiles were observed in both case populations (Figures 1(b) and 1(c)). In particular, the expression profiles of lncRNAs of the individuals in the two case populations were clustered into a big group consisting of two subgroups which, respectively, corresponded to those of individuals in case populations 1 and 2 (Figure 1(c)), suggesting that there is a general linkage between QDC and PQDS, but there are also differences between them.

### 3.2. GO Functions of Differential Genes

**Figure 2:**
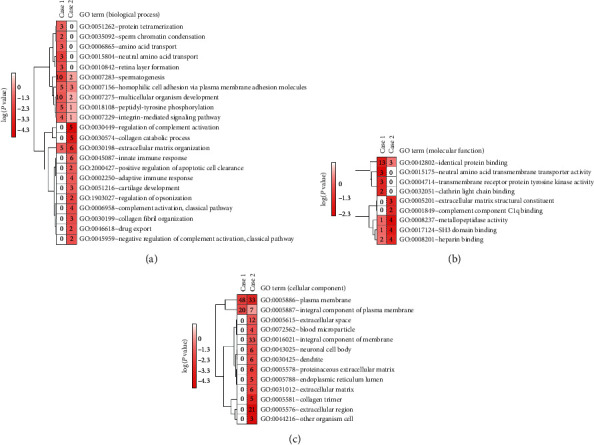
Comparison of GO function enrichments of differential genes identified in the two case populations. (a) Comparison of biological process enrichments of differential genes. (b) Comparison of molecular function enrichments of differential genes. (c) Comparison of cellular component enrichments of differential genes. Case 1: qi-deficiency constitution; case 2: Pi-qi-deficiency syndrome of chronic superficial gastritis.

A total of 183 differential genes were found in case population 1 (QDC), containing 114 upregulated genes and 69 downregulated genes. GO enrichment analysis was performed to explore their functions. We found that 10 GO (biological process) terms were significantly enriched, including peptidyl-tyrosine phosphorylation, protein tetramerization, amino acid transport, and especially the terms related to cell-cell adhesion and communication such as extracellular matrix organization, cell adhesion via plasma membrane adhesion molecules, and integrin-mediated signaling (Figure 2(a)). Moreover, GO (cellular component, molecular function) terms indicated that these enriched genes were responsible for encoding plasma membrane proteins (Figure 2(c)) and maintaining the protein binding and transmembrane transporter activity (Figure 2(b)).

Also, 93 differential genes were identified in case population 2 (PQDS of CSG), consisting of 51 upregulated genes and 42 downregulated genes. Interestingly, these genes shared the GO (biological process) terms which were also matched in QDC, associated with cell-cell adhesion and communication including extracellular matrix organization and cell adhesion via plasma membrane adhesion molecules (Figure 2(a)). However, the GO (biological process) terms specifically enriched in the PQDS of CSG were these terms such as the regulation of complement activation, collagen catabolic process, and innate/adaptive immune response (Figure 2(a)), belonging to extracellular region/space proteins or the integral components of membrane (Figure 2(c)). Considering all results together, common biological processes, namely, extracellular matrix organization and cell adhesion via plasma membrane adhesion molecules, were implicated in both QDC and PQDS of CSG, indicating the possible alternation of cell-cell adhesion and communication that contributed to the characteristics and functions of circling leukocytes in a body.

### 3.3. GO Functions, KEGG, and Reactome Pathways Identified by GSEA

Using the healthy population as control, the observed common GO biological processes with positive correlation with QDC and PQDS included negative regulation of execution phase of apoptosis, as well as regulation of apoptotic cell clearance, excitatory synapse assembly, postsynapse assembly, and postsynaptic density assembly. Also, common biological processes that had negative correlation with QDC and PQDS were implicated in the cotranslational protein and SRP-dependent cotranslational protein targeting to membrane, maintenance of organelle location, and DNA replication-dependent nucleosome organization and assembly (Supplemental Figure S2). In addition, regarding the enriched pathways, the identified common pathways keeping positive correlation with QDC and PQDS were the amphetamine addiction and other Reactome pathways, such as activation of C3 and C5, synthesis of 12-eicosatetraenoic acid derivatives, WNT5A-dependent internalization of FZD4, cargo concentration in the ER, and disinhibition of SNARE formation (Supplemental Figure S3). The obtained GSEA results of genome-wide expression profiles revealed that the TCM-defined QDC and PQDS share several common enriched biological processes and pathways, suggesting the potential common contribution to the characteristics and functions of leukocytes in a body.

### 3.4. Enriched Pathways and Diseases: Interaction Network of QDC-Specific Genes

**Figure 3:**
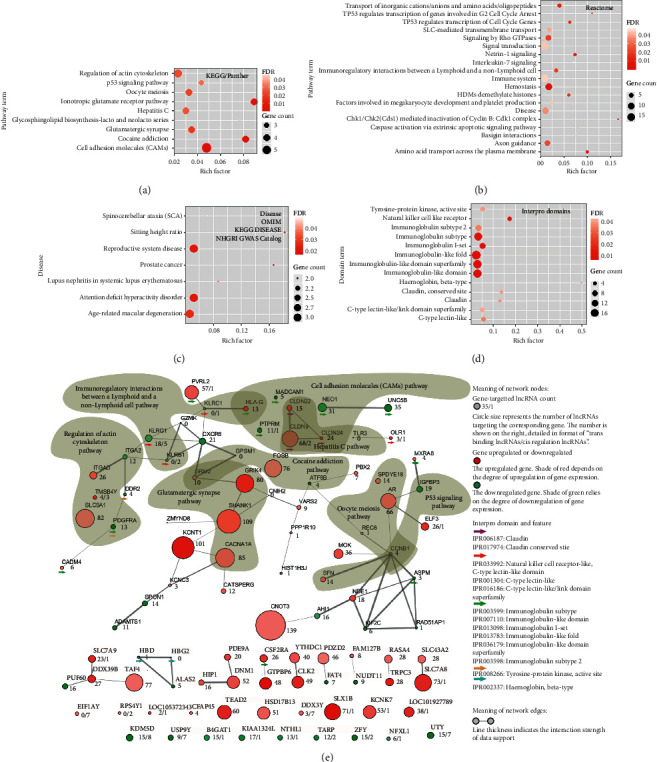
Enrichment and interaction network analysis for the qi-deficiency constitution- (QDC-) specific genes. (a) Enriched KEGG and Panther pathways of QDC-specific genes. (b) Enriched Reactome pathways of QDC-specific genes. (c) Enriched diseases associated with QDC-specific genes. (d) Enriched domains and features of the proteins coded by QDC-specific genes. (e) Network to detail the interactions among QDC-specific genes. The number close to a node denotes the number of QDC-lncRNAs targeting the corresponding node gene.

The QDC-specific genes in this study mean the differential genes which appeared only in individuals from QDC population rather than CSG patients with PQDS. As indicated (Figure 1(a)), a total of 171 differential genes were QDC-specific, and the pathway-based enrichment analyses of them were performed, including the KEGG, Panther, and Reactome pathways. The enriched KEGG/Panther pathways with higher rich factor (Figure 3(a)) contained the ionotropic glutamate receptor, cocaine addiction, and cell adhesion molecules. The matched Reactome pathways (Figure 3(b)) included the immune cell and system pathways (immunoregulatory interactions between a lymphoid and a nonlymphoid cell, interleukin-7 signaling, caspase activation via extrinsic apoptotic signaling, factors involved in megakaryocyte development and platelet production), the cell cycle regulation pathways (CHK1/CHK2-mediated inactivation of cyclin B, TP53-regulated transcription of genes involved in G2 cell cycle arrest, TP53-regulated transcription of cell cycle genes), the transmembrane transport pathways (amino acid transport across the plasma membrane, transport of inorganic cations/anions and amino acids/oligopeptides, SLC-mediated transmembrane transport), and other pathways. In particular, the InterPro domain and feature enrichment analyses demonstrated that these QDC-specific genes-corresponding proteins contained immune-related domains including immunoglobulin-like domain, natural killer cell receptor, C-type lectin-like domain, tyrosine-protein kinase active sites, and claudin conserved sites (Figure 3(d)). The corresponding proteins containing various immune-related domains were specially marked by colorful arrows (Figure 3(e)). Moreover, the disease-based enrichment analysis showed that they were related to diseases such as age-related macular degeneration, lupus nephritis in systemic lupus erythematosus, and spinocerebellar ataxia (Figure 3(c)).

To detail the interactions of QDC-specific genes (Supplemental Table S7), as well as the regulatory relationships of a gene and its *trans*- and *cis*-acting lncRNAs (QDC-specific) (Supplemental Tables S8 and 9), an interaction complex network was carefully created, integrated with any type of edge and node attribute data such as gene expression pattern and the number of *trans*- and *cis*-acting lncRNAs (Figure 3(e)). In particular, several enriched pathways were highlighted and annotated in the created network. The resultant network not only detailed the interactions of QDC-specific genes (edge thickness indicates interaction strength of data support), but also presented the gene expression pattern (shade of green or red depends on degree of downregulation or upregulation of gene expression) and the number of *trans*- and *cis*-acting lncRNAs (node size denotes the number of lncRNAs targeting the node gene, and the number is specifically shown in format of “*trans*-acting lncRNAs number/*cis*-acting lncRNAs number”). As shown in the network, overall, almost each gene was regulated by multiple *trans*-acting lncRNAs and even additional *cis*-acting lncRNAs. For instance, each of these genes could be regulated by many *trans*-acting lncRNAs (more than 80), including CNOT3, SHANK1, KCNT1, CACNA1A, SLC9A1, and GRIK4. Also, each of the genes such as KDM5D, UTY, USP9Y, DDX3Y, and KLRG1 could be controlled by additional multiple *cis*-acting lncRNAs (over 5). Although many genes had no interactions with each other (no edge between each other in network), they were also found to be regulated by *trans*- or *cis*-acting lncRNAs. Notably, multiple *trans*-acting lncRNAs and *cis*-acting lncRNAs were observed to target the genes encoding for the immune-related domains-contained proteins in the pathways associated with the cell-cell adhesion and communication, such as cell adhesion molecules (PTPRM, CLDN9) and immunoregulatory interactions between a lymphoid and a nonlymphoid cell (KLRB1, KLRC, KLRG1). Therefore, the above-mentioned results indicated that the QDC-specific lncRNAs played important roles in regulating the QDC-specific gene expression profiles which determined the characteristics and functions of leukocytes in QDC population.

### 3.5. Interaction Network of PQDS-Specific Genes for Enriched Pathways and Diseases

**Figure 4:**
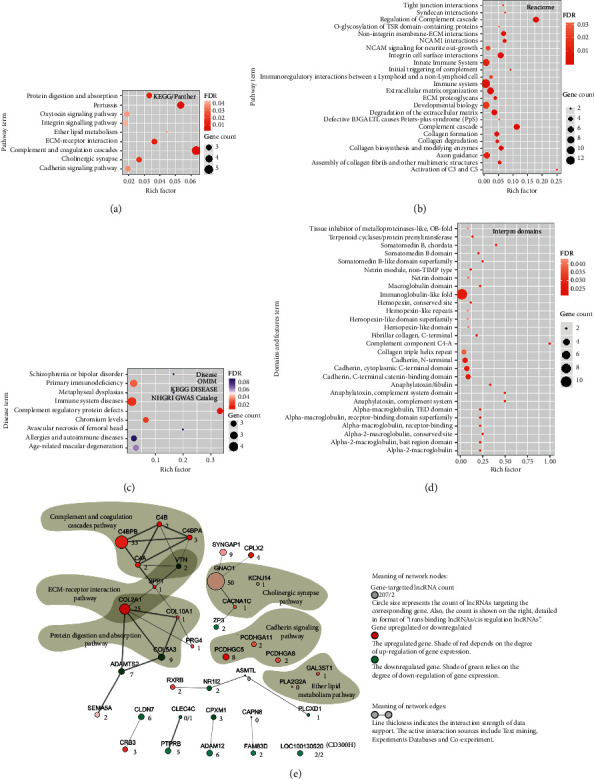
Enrichment and interaction network analysis for the Pi-qi-deficiency syndrome- (PQDS-) specific genes. (a) Enriched KEGG and Panther pathways of PQDS-specific genes. (b) Enriched Reactome pathways of PQDS-specific genes. (c) Enriched diseases associated with PQDS-specific genes. (d) Enriched domains and features of the proteins coded by PQDS-specific genes. (e) Network to detail the interactions among PQDS genes. The number near a node denotes the number of PQDS-lncRNAs targeting the corresponding node gene.

A total of 81 PQDS-specific genes were found in CSG patients with PQDS (Figure 1(a)). They were enriched in the KEGG/Panther pathways including complement and coagulation cascades; ether lipid metabolism; and pathways involved in cell-cell junction/adhesion and communication such as cadherin signaling, extracellular matrix- (ECM-) receptor interaction, protein digestion and absorption, integrin signaling, and cholinergic synapse pathway (Figure 4(a)). Furthermore, the enriched Reactome pathways contained the immune-related pathways implicated in complement cascades and regulation of complement cascades and immunoregulatory interactions between a lymphoid and a nonlymphoid cell. Also, in particular, multiple enriched Reactome pathways were associated with cell-cell interactions such as adhesion, junction, and communication (integrin cell surface interactions, extracellular matrix organization, nonintegrin membrane-ECM interactions, degradation of the extracellular matrix, collagen biosynthesis and modifying enzymes, collagen formation/degradation, assembly of collagen fibrils and other multimeric structures, ECM proteoglycans, syndecan interactions, tight junction interactions, NCAM1 interactions and signaling) (Figure 4(b)). In particular, domain and feature enrichment analysis showed that these PQDS-specific proteins generally contained domains and features such as immunoglobulin-like fold, collagen triple helix repeat, cadherin N-terminal or C-terminal domain, and complement system and component domain (Figure 4(d)). In addition, the disease enrichment analysis indicated that these PQDS genes were associated with the diseases related to complement regulatory protein defects, immune system diseases, and primary immunodeficiency.

The interaction network (Figure 4(e)) not only shows the possible interactions and expression pattern of PQDS-specific genes (Supplemental Table S10), but also indicates the number of possible *trans*/*cis*-acting lncRNAs directly targeting to node genes (Supplemental Tables S11 and 12). Obviously, almost each gene has the corresponding trans-acting lncRNAs, and the genes CLEC4C and LOC100130520 (CD300H) have additional *cis*-acting lncRNAs. C4BPB, an important negative factor of complement activation, was regulated by over 30 *trans*-acting lncRNAs in the complement and coagulation cascades pathway. COL2A1 was regulated by 25 *trans*-acting lncRNAs; it is an important gene responsible for cross talk of two pathways involved in cell-cell adhesion and communication such as extracellular matrix- (ECM-) receptor interaction pathway and protein digestion and absorption pathway. Also, GNAO1 and PCDHGC5 were governed by multiple *trans*-acting lncRNAs, which were matched in the other two pathways related to cell-cell adhesion and communication, namely, cholinergic synapse pathway and cadherin signaling pathway (Figure 4(e)). Together, these results suggested that the PQDS-specific lncRNAs seemed to be crucial in the regulation of expression profile of PQDS-specific genes, especially the expression regulation of genes in multiple pathways associated with complement and coagulation cascades as well as cell-cell adhesion/junction and communication, which contributed to the characteristics and functions of leukocytes in CSG patients of PQDS.

### 3.6. lncRNA-Gene Regulation Pairs

**Figure 5:**
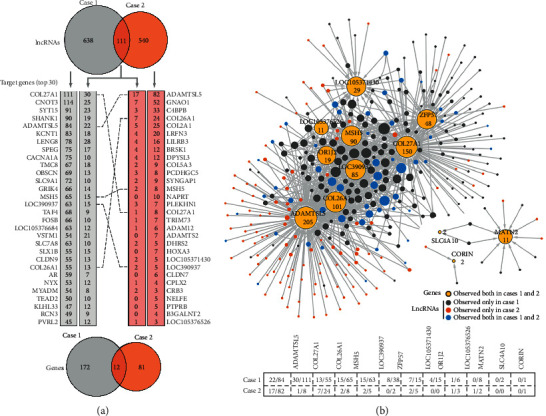
Overview of differential genes undergoing regulation of differential lncRNAs in each case population. (a) Top 30 targeting genes regulated by different differential lncRNAs in each case population. Venn diagrams denote the numbers of differential lncRNAs and genes found in each case population. 111 lncRNAs and 12 genes were common in both case populations. (b) Interaction network to detail the common genes and their possible regulating lncRNAs. Node size depends on the number of edges, and the number of regulating lncRNAs is shown near the corresponding node. The numbers of lncRNAs targeting each common gene were carefully presented in format of “number of the common regulating lncRNAs (blue)/number of the case-specific lncRNAs identified in case 1 (grey) or case 2 (brown).” Case 1: qi-deficiency constitution; case 2: Pi-qi-deficiency syndrome of chronic superficial gastritis.

Based on the results of target gene prediction of lncRNAs, we drew Venn diagrams to show the numbers of differential lncRNAs and genes identified in the leukocytes of individuals from the two case populations, adding lists of differential genes for both case populations to display and compare the top 30 genes which have corresponding *trans*- or *cis*-acting lncRNAs (Figure 5(a)). In the gene lists, for case population 1 (QDC), the black font denotes the number of QDC-specific lncRNAs regulating the QDC genes, and the blue font shows the number of common lncRNAs targeting the QDC genes. Similarly, regarding case population 2 (CSG patients with PQDS), the black font denotes the number of PQDS-specific lncRNAs controlling the PQDS genes, and the blue font shows the number of common lncRNAs targeting the PQDS genes. For the listed top 30 genes in each case population, each gene was regulated by many lncRNAs. Interestingly, five (bold font) of them, COL27A1, ADAMTSL5, MSH5, COL26A1, and LOC390937, belonged to the common differential genes found in both case populations (Figure 5(a)), indicating that the common genes seemed to be undergoing tight and complex regulation of more lncRNAs due to their potential roles in linking QDC to PQDS.

Thus, in order to detail the interactions and regulation between the differential lncRNAs and all the 12 common genes (Supplemental Table S13), an interaction network was carefully created (Figure 5(b)). The 12 common genes and their corresponding regulation lncRNAs were shown, including the QDC-specific lncRNAs (grey nodes), PQDS-specific lncRNAs (brown nodes), and the common lncRNAs (blue nodes) identified in both QDC and PQDS. Also, the numbers of *trans*- and *cis*-acting lncRNAs targeting each common gene were carefully presented (Figure 5(b)). As indicated, most common genes underwent very tight regulation of many lncRNAs, not only the specific lncRNAs in QDC or PQDS but also the common lncRNAs in both QDC and PQDS. In particular, the genes ADAMTSL5, COL26A1, MSH5, LOC390937, and COL27A1 could be regulated by the QDC-specific lncRNAs, PQDS-specific lncRNAs, and the common lncRNAs. Overall, these results show that lncRNAs play important roles in maintenance and regulation of gene expression profiles which determine the characteristics and functions of leukocytes in QDC and PQDS populations. Notably, the common differential genes undergo so tight and complex regulation of lncRNAs including the common lncRNAs, which may be due to their potential roles in linking QDC to PQDS.

### 3.7. The Common lncRNAs-Mediated Regulation of Common Genes

**Figure 6:**
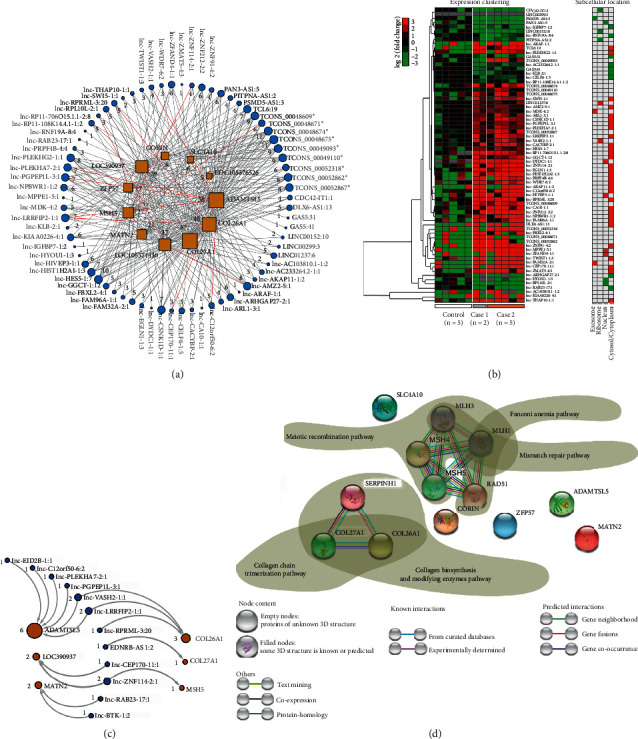
The common differential lncRNAs-mediated regulation of the common differential genes in two case populations. (a) Network to detail the possible direct binding of lncRNAs to the mRNAs of common genes. 70 lncRNAs could directly bind to the mRNAs of 11 genes. Nodes with red font labels indicate a consistent upregulation expression in both case populations. Nodes with green font denote a consistent downregulation expression in both case populations. Node size depends on the number of edges, and the number is shown near the corresponding node. The “^*∗*^”-labeled lncRNAs were novel in this work. (b) Expression clustering and subcellular location analyses for the 70 common lncRNAs capable of directly binding to the mRNAs of common genes. Prediction of subcellular location involves exosome, ribosome, nucleus, cytosol, and cytoplasm. Red or green labels indicate that the corresponding lncRNA is consistent upregulation or downregulation expression in both case populations. (c) Network to detail the possible interactions between the common lncRNAs and the common genes-coded proteins. 17 lncRNAs could directly bind to 7 proteins. Node size depends on the number of edges, and the number is shown near the corresponding node. (d) Interaction network analysis of the common genes.

lncRNA may directly bind to mRNAs, mediating the posttranscriptional regulation of mRNAs. Also, if possible, it also binds to the mRNA-coded protein to affect protein functions. Herein, in order to decode the common differential lncRNAs-mediated regulation of the common genes and their coded proteins, we first carefully analyzed the RNA-binding ability between the common lncRNAs (total 111) and genes (total 12). A total of 70 lncRNAs possibly bind to the genes' (total 11) corresponding mRNAs in both case populations (Supplemental Table S14), and an interaction network was drawn to detail the binding pairs of lncRNAs and genes (Figure 6(a)). Besides, the HCL heatmap was generated to review the expression patterns of all the 70 lncRNAs (Figure 6(b)). The upregulated lncRNAs and the downregulated lncRNAs identified in both case populations were specifically labeled in red or green font. Also, we predicted the subcellular location for all the 70 lncRNAs in the network. As indicated (Figure 6(b)), four lncRNAs might be located in nucleus of leukocyte, two lncRNAs could be ribosome-related, and 62 lncRNAs were distributed in cytosol or cytoplasm. Notably, two lncRNAs, lnc-FAM32A-2:1 and lnc-MDK-4:2, could be encapsulated in exosomes which were capable of transferring them to other recipient cells all over the body.

Moreover, to explore the common differential lncRNAs-mediated regulation of the common genes-corresponding proteins, we evaluated the RNA-protein interactions between the common lncRNAs and genes-corresponding proteins under the prerequisite of coexpression of a lncRNA and a gene. A total of 12 lncRNAs were capable of directly binding to 6 genes-coded proteins in both case populations (Supplemental Table S15). The resultant network was shown to detail the RNA-protein interactions between the lncRNAs and proteins (Figure 6(c)). The upregulated lncRNAs (red font) and the downregulated lncRNAs (green font) identified in the two case populations were labeled, respectively.

These results indicated that the common lncRNAs seemed to be very important posttranscriptional regulators, controlling the common genes by direct binding to either the transcribed mRNAs or the translated proteins. The common genes underwent so complex regulation of the common lncRNAs, suggesting once again their possible potential roles in linking QDC to PQDS of CSG. Therefore, we performed another interaction network analysis of the common genes, and the obtained interaction network was drawn and annotated (Figure 6(d)). The functional enrichment in the generated network including GO and pathway was detailed (Supplemental Table S16to 20). The enrichment analyses results showed that they mainly belonged to intracellular organelle lumen and extracellular matrix component including collagen-containing extracellular matrix and were involved in pathways such as collagen chain trimerization and collagen biosynthesis and modifying enzymes. In particular, relying on functional partners of CORIN and MSH5, several additional pathways were involved, including mismatch repair pathway, meiotic recombination pathway, and Fanconi anemia pathway (Figure 6(d)). The obtained results demonstrate the common changes in the extracellular matrix related to cell-cell adhesion/junction and communication, which contribute to alteration in characteristics and functions of leukocytes of individuals in both case populations and may be involved in the linkage between QDC and PQDS.

### 3.8. Functions of the Exosome-Contained lnc-MDK-4:2 and lnc-FAM32A-2:1

**Figure 7:**
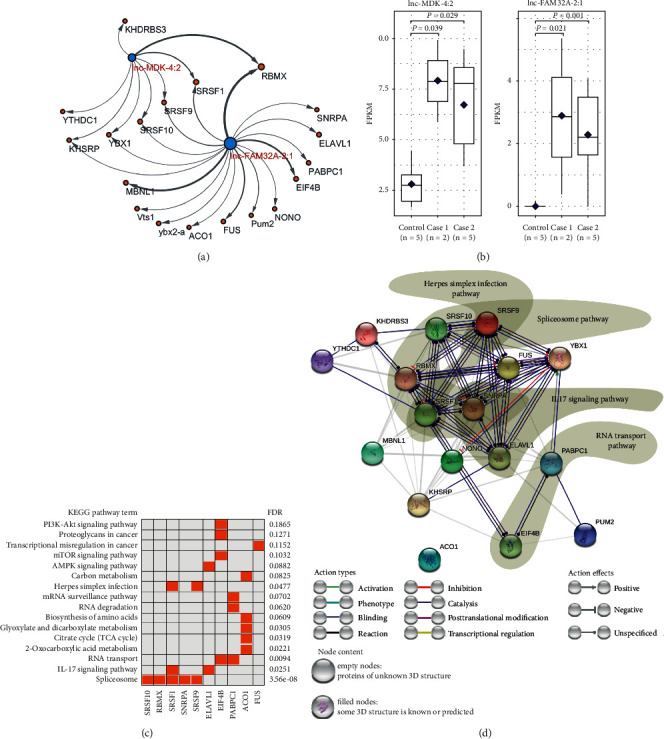
Potential functions of lnc-MDK-4:2 and lnc-FAM32A-2:1 contained in leukocytes-derived exosome. (a) Network to detail RNA-binding proteins (RBPs) of lnc-MDK-4:2 and lnc-FAM32A-2:1. Node size depends on the number of edges. (b) Expression patterns of lnc-MDK-4:2 and lnc-FAM32A-2:1. (c) Pathway enrichment analysis of the RBPs of lnc-MDK-4:2 and lnc-FAM32A-2:1. (d) Interaction network analysis for the RBPs of lnc-MDK-4:2 and lnc-FAM32A-2:1. Control: balanced constitution; case 1: qi-deficiency constitution; case 2: Pi-qi-deficiency syndrome of chronic superficial gastritis.

The mentioned lncRNAs, lnc-FAM32A-2:1 and lnc-MDK-4:2, were predicted to be encapsulated in exosomes, especially keeping higher transcript levels in both case populations (Figure 7(b)). The exosomes might transfer the lncRNAs to other far away recipient cells, making them function all over the body. Thus, in order to analyze their potential roles, we predicted their possible RBPs (Supplemental Table S21), based on the well-known RBP database that collects the experimental observations of RNA-binding sites, both *in vitro* and *in vivo*. As shown (Figure 7(a)), the interaction network was drawn to detail RNA-protein interactions between the lncRNAs and their RBPs (edge thickness indicates interaction strength of RNA-binding sites support). Furthermore, the enrichment analysis of the obtained RBPs revealed their potential pathways related to RNA processing and transport, IL17 signaling, biosynthesis and metabolism, and regulation of autophagy initiation (Figure 7(c)). Also, the interaction network was created to detail the interactions between multiple RBPs, including their physical and functional association (Figure 7(d)). These results demonstrated that the exosomes-contained lnc-MDK-4:2 and lnc-FAM32A-2:1 might play potential important roles in regulating biosynthesis and metabolism in the recipient cells all over the body, especially the IL17-mediated signaling and autophagy initiation regulation which contributed to host defense and pathogenesis of various autoimmune diseases.

## 4. Discussion

TCM was developed through thousands of years of empirical testing and refinement. Nowadays, it is getting more frequently adopted in countries in the west [1]. Syndrome, a thousand-year-old key therapeutic concept in TCM, is defined as a pattern of symptoms and signs in a patient at a specific stage during the course of a disease [35, 36]. Persons with QDC seem to have a tendency toward PQDS, one of the commonly matched TCM syndromes in CSG patients [6–8]. In particular, they have common features including lacking vitality (Qi) and getting sick easily, being generally characterized by languid lazy words, low voice, pale tongue, and weak pulse [4, 8]. That is to say, there seemed to be a linkage between QDC and PQDS, indicating a decrease in immunity in both QDC and PQDS. Leukocytes, as important immune cells throughout the body, play crucial roles in host defense against infection and contribute to pathogenesis of various autoimmune diseases. Therefore, the alternations in characteristics and functions of leukocytes may implicate linking QDC to PQDS. Because gene expression profile determines cell characteristics and functions, the similar gene expression profiles observed in the leukocytes from QDC and PQDS populations (Figure 1) suggested several similar alterations in characteristics and functions of leukocytes. Function enrichment analyses of differential genes just showed that common biological processes, including extracellular matrix organization and cell adhesion via plasma membrane adhesion molecules, were involved in both QDC and PQDS, indicating the possible alternations in cell-cell adhesion/junction and communication which contributed to characteristics and functions of leukocytes all over the body (Figure 2(a)).

For QDC-specific genes, their enriched pathways (Figures 3(a) and 3(b)) included cell cycle regulation pathways, transmembrane transport pathways, and especially immune pathways related to cell adhesion and signaling. In particular, they were enriched in proteins that contained immune-related domains (Figure 3(d)). Notably, multiple QDC-specific lncRNAs targeted genes (coding for immune domains-contained proteins) in the pathways associated with cell-cell adhesion and communication (Figure 3(e)), including the cell adhesion molecules (PTPRM, CLDN9) and immunoregulatory interactions between a lymphoid and a nonlymphoid cell (KLRB1, KLRC, KLRG1). PQDS-specific genes were enriched in pathways including complement and coagulation cascades; ether lipid metabolism; and pathways involved in cell-cell junction/adhesion and communication (Figures 4(a) and 4(b)). In particular, C4BPB, a negative factor of complement activation, was regulated by over 30 lncRNAs in the complement and coagulation cascades pathway. COL2A1, an important gene responsible for cross talk of two pathways involved in cell-cell adhesion and communication, was regulated by 25 lncRNAs. GNAO1 and PCDHGC5 were governed by multiple lncRNAs, which were in the pathways related to cell-cell adhesion and communication (Figure 4(e)). All the results indicated that QDC- or PQDS-specific lncRNAs played very important roles in regulating the QDC- or PQDS-specific gene expression which determined characteristics and functions of leukocytes in different case populations.

Regarding the common differential genes appearing in both QDC and PQDS, most of them underwent very tight regulation of many lncRNAs. In particular, the genes ADAMTSL5, COL26A1, MSH5, LOC390937, and COL27A1 could be regulated by the QDC-specific lncRNAs, the PQDS-specific lncRNAs, and the common lncRNAs (Figures 5(b) and 6(a)). A total of 12 lncRNAs were capable of directly binding to 6 genes-coded proteins in both case populations (Figure 6(c); Supplemental Figures S4 and S5). These results indicated that the common lncRNAs seemed to be very important posttranscriptional regulators, controlling the common genes by direct binding to either the transcribed mRNAs or the translated proteins. The common genes underwent so complex regulation of common lncRNAs, suggesting their potential roles in linking QDC to PQDS. In particular, the common changes in the extracellular matrix and integral components of plasma membrane, which are related to cell-cell adhesion/junction and communication, may be involved in the linkage between QDC and PQDS, contributing to the alterations in characteristics and functions of leukocytes of individuals in both case populations.

In addition, the high expression of the exosomes-carried lnc-MDK-4:2 and lnc-FAM32A-2:1 (Figures 6(b) and 7) in both case populations probably implicates linking QDC to PQDS, because of their potential roles in regulating biosynthesis and metabolism in the recipient cells all over the body, especially the IL17-mediated signaling and autophagy initiation which contributed to host defense and pathogenesis of various autoimmune diseases.

The estimation of lncRNA from RNA sequencing enabled the identification of both lncRNAs and mRNAs only by constructing an RNA-seq library, but such an approach could underestimate the possible lncRNAs, especially the novel and unannotated lncRNAs. The transcriptomic analyses of blood leukocytes revealed the lncRNA-mediated complex regulation of candidates implicated in the possible link between QDC and PQDS of CSG. However, the study population was not large enough to draw definitive conclusions; in particular, only two female individuals were included in case population 1. Also, there were significant differences in age between CSG and health control groups, which may lead to biased conclusions. Hence, further studies involving larger sample sizes are required to strengthen the conclusions of this study. Additionally, the PCR validation of the expression of the common differential lncRNAs and genes should be performed in larger populations.

## 5. Conclusions

A total of 12 common differential genes, obtained in QDC and PQDS, could be regulated by the QDC-specific lncRNAs, the PQDS-specific lncRNAs, or the common lncRNAs. Notably, some of them underwent very tight and complex regulation of the common differential lncRNAs by directly binding the transcribed mRNAs and the translated proteins. Several common biological processes, including extracellular matrix organization and cell adhesion via plasma membrane adhesion molecules, were implicated in both QDC and PQDS, thereby indicating the possible alternations in cell-cell adhesion/junction and communication, which contributed to characteristics and functions of leukocytes all over the body. These results may provide new insights into the characteristic and functional changes of leukocytes in QDC and PQDS, especially the mechanism underlying the linkage of QDC to PQDS, with potential leukocytes biomarkers for future application in integrative medicine.

## Data Availability

All sequence data have been deposited in GenBank under BioProject accession number PRJNA591186 (https://www.ncbi.nlm.nih.gov/bioproject/PRJNA591186). The RNA-seq reads have been deposited in the NCBI Sequence Read Archive (SRA) (http://www.ncbi.nlm.nih.gov/sra) under accession numbers SRR10513209, SRR10513208, SRR10513204, SRR10513203, SRR10513202, SRR10513205, SRR10513201, SRR10513200, SRR10513199, SRR10513198, SRR10513207, and SRR10513206.

## Supplementary Materials

Supplementary Materials Supplemental Methods: the inclusion and exclusion criteria for subjects and the experimental design and route. Table S1: list for leukocyte samples of the clinical subjects. Table S2: statistical results of the alignment of the cleaned raw reads to the reference genome. Table S3: differential genes identified in the QDC population compared with the BC control population. Table S4: differential genes identified in the PQDS population compared with the BC control population. Table S5: differential lncRNAs identified in the QDC population compared with the BC control population. Table S6: differential lncRNAs identified in the PQDS population compared with the BC control population. Table S7: interactions between QDC-specific genes-coded proteins. Table S8: the QDC-specific differential genes regulated by QDC-specific differential *trans*-acting lncRNAs order by genes. Table S9: detailed *cis*-regulation pairs between the QDC-specific differential genes and lncRNAs in QDC population. Table S10: interactions between PQDS-specific genes-coded proteins. Table S11: the PQDS-specific differential genes regulated by the PQDS-specific differential lncRNAs order by genes. Table S12: detailed *cis*-regulation pairs between the PQDC-specific differential genes and lncRNAs in PQDS population. Table S13: detailed list for binding pairs of the differential lncRNAs and the 12 common differential genes. Table S14: the possible lncRNA-mRNA binding among the common differential lncRNAs and genes. Table S15: the lncRNA-protein interactions between the common differential lncRNAs and genes-coded proteins. Table S16: the 17 biological process (GO) terms enriched with the common targets and the predicted functional partners. Table S17: the 11 cellular component (GO) terms enriched with the common targets and the predicted functional partners. Table S18: the 11 molecular function (GO) terms enriched with the common targets and the predicted functional partners. Table S19: reactome pathways significantly enriched with the common targets and the predicted functional partners. Table S20: KEGG pathways significantly enriched with the common targets and the predicted functional partners. Table S21: the RNA-binding proteins (RBPs) and binding motifs of lnc-FAM32A-2:1 and lnc-MDK-4:2. Figure S2: comparison of GO function enrichments identified by the GSEA method in two case populations. Figure S3: comparison of pathway enrichments identified by GSEA method in two case populations. Figure S4: mismatch repair pathway (hsadd03430). Figure S5: Fanconi anemia pathway (hsadd03460).
