# Supplementary material for: Integrated Analyses of lncRNA and mRNA Profiles Reveal Characteristic and Functional Changes of Leukocytes in Qi-Deficiency Constitution and Pi-Qi-Deficiency Syndrome of Chronic Superficial Gastritis
Source: Evid Based Complement Alternat Med. 2020 Jul 16;2020:8518053. doi: 10.1155/2020/8518053 (PMC7381957; doi:10.1155/2020/8518053)
Supplement: Supplementary Materials — Supplemental Methods: the inclusion and exclusion criteria for subjects and the experimental design and route. Table S1: list for leukocyte samples of the clinical subjects. Table S2: statistical results of the alignment of the cleaned raw reads to the reference genome. Table S3: differential genes identified in the QDC population compared with the BC control population. Table S4: differential genes identified in the PQDS population compared with the BC control population. Table S5: differential lncRNAs identified in the QDC population compared with the BC control population. Table S6: differential lncRNAs identified in the PQDS population compared with the BC control population. Table S7: interactions between QDC-specific genes-coded proteins. Table S8: the QDC-specific differential genes regulated by QDC-specific differential trans-acting lncRNAs order by genes. Table S9: detailed cis-regulation pairs between the QDC-specific differential genes and lncRNAs in QDC population. Table S10: interactions between PQDS-specific genes-coded proteins. Table S11: the PQDS-specific differential genes regulated by the PQDS-specific differential lncRNAs order by genes. Table S12: detailed cis-regulation pairs between the PQDC-specific differential genes and lncRNAs in PQDS population. Table S13: detailed list for binding pairs of the differential lncRNAs and the 12 common differential genes. Table S14: the possible lncRNA-mRNA binding among the common differential lncRNAs and genes. Table S15: the lncRNA-protein interactions between the common differential lncRNAs and genes-coded proteins. Table S16: the 17 biological process (GO) terms enriched with the common targets and the predicted functional partners. Table S17: the 11 cellular component (GO) terms enriched with the common targets and the predicted functional partners. Table S18: the 11 molecular function (GO) terms enriched with the common targets and the predicted functional partners. Table S19: reactome pathways signif [file 8518053.f1.doc]

**Supplementary Information**

Leiming You, Aijie Liu, *et al*. Integrated analyses of lncRNA and mRNA profiles reveal characteristic and functional changes of leukocytes in qi deficiency constitution and Pi-qi-deficiency syndrome of chronic superficial gastritis

**Contents**

[Supplemental Methods 1](#__RefHeading___Toc42039116)

[**The inclusion and exclusion criteria for subjects** 1](#__RefHeading___Toc42039117)

[**The experimental design and route** 2](#__RefHeading___Toc42039118)

[Supplemental Tables 3](#__RefHeading___Toc42039119)

[**Table S1** List for leukocyte samples of the clinical subjects. 4](#__RefHeading___Toc42039120)

[**Table S2** Statistical results of the alignment of the cleaned raw reads to the reference genome 4](#__RefHeading___Toc42039121)

[**Table S3** Differential genes identified in the QDC population compared with the BC control population 4](#__RefHeading___Toc42039122)

[**Table S4** Differential genes identified in the PQDS population compared with the BC control population 7](#__RefHeading___Toc42039123)

[**Table S5** Differential lncRNAs identified in the QDC population compared with the BC control population 9](#__RefHeading___Toc42039124)

[**Table S6** Differential lncRNAs identified in the PQDS population compared with the BC control population 21](#__RefHeading___Toc42039125)

[**Table S7** Interactions between QDC-specific genes coded proteins 32](#__RefHeading___Toc42039126)

[**Table S8** The QDC-specific differential genes regulated by QDC-specific differential *trans*-acting lncRNAs order by genes 34](#__RefHeading___Toc42039127)

[**Table S9** Detailed the *cis*-regulation pairs between the QDC-specific differential genes and lncRNAs in QDC population 42](#__RefHeading___Toc42039128)

[**Table S10** Interactions between PQDS-specific genes coded proteins 44](#__RefHeading___Toc42039129)

[**Table S11** The PQDS**-**specific differential genes regulated by the PQDS-specific differential lncRNAs order by genes 45](#__RefHeading___Toc42039130)

[**Table S12** Detailed the *cis*-regulation pairs between the PQDC-specific differential genes and lncRNAs in PQDS population 47](#__RefHeading___Toc42039131)

[**Table S13** Detailed list for binding pairs of the differential lncRNAs and the 12 common differential genes 48](#__RefHeading___Toc42039132)

[**Table S14** The possible lncRNA-mRNA binding among the common differential lncRNAs and genes. 58](#__RefHeading___Toc42039133)

[**Table S15** The lncRNA-protein interactions between the common differential lncRNAs and genes-coded proteins 59](#__RefHeading___Toc42039134)

[**Table S16** The 17 biological process (GO) terms enriched with the common targets and the predicted functional partners. 59](#__RefHeading___Toc42039135)

[**Table S17** The 11 cellular component (GO) terms enriched with the common targets and the predicted functional partners. 60](#__RefHeading___Toc42039136)

[**Table S18** The 11 molecular function (GO) terms enriched with the common targets and the predicted functional partners. 60](#__RefHeading___Toc42039137)

[**Table S19** Reactome pathways significantly enriched with the common targets and the predicted functional partners. 61](#__RefHeading___Toc42039138)

[**Table S20** KEGG pathways significantly enriched with the common targets and the predicted functional partners. 61](#__RefHeading___Toc42039139)

[**Table S21** The RNA binding proteins (RBPs) and binding motifs of lnc-FAM32A-2:1 and lnc-MDK-4:2 62](#__RefHeading___Toc42039140)

[Supplemental Figures 64](#__RefHeading___Toc42039141)

[**Figure S2** Comparison of GO function enrichments identified by GSEA method in two case population. 64](#__RefHeading___Toc42039142)

[**Figure S3** Comparison of pathway enrichments identified by GSEA method in two case population. 65](#__RefHeading___Toc42039143)

[**Figure S4** Mismatch repair pathway (hsadd03430) 66](#__RefHeading___Toc42039144)

[**Figure S5** Fanconi anemia pathway (hsadd03460) 67](#__RefHeading___Toc42039145)

[References 68](#__RefHeading___Toc42039146)

**Supplemental Methods**

**The inclusion and exclusion criteria for subjects**

(**I**) According to the Traditional Chinese medicine (TCM) constitution assessment , subject was assessed as Balanced constitution (BC) or Qi deficiency constitution (QDC) only. Also, no abnormality was found in blood, urine, stool routine, biochemical tests, chest X-ray and electrocardiogram. Endoscopic diagnosis was also performed to exclude healthy persons suffering from chronic superficial gastritis.

***TCM constitution measurement*** Based on the *Constitution in Chinese Medicine Questionnaire* (CCMQ), addressed at <http://genome.bucm.edu.cn/ctz>, a further face-to-face discrimination analysis was applied to determine the constitution type of each subject [Balanced constitution (BC) or Qi deficiency constitution (QDC) only].

TCM constitutions were measured and classified using the Constitution in Chinese Medicine Questionnaire (CCMQ) . Actually, the CCMQ was based on the data from 542 subjects with typical constitutions diagnosed by TCM constitution experts . The CCMQ is a self-rating scale with good reliability and validity. It has 60 items, which contains nine subscales: gentleness, qi-deficiency, yang-deficiency, yin-deficiency, phlegmdampness, dampness-heat, blood-stasis, qi-depression, and special diathesis (allergic constitution). Score of each subscale is standardized from 0 to 100. Gentleness type (balanced constitution) is a balanced one with higher score indicating a better constitution status, while the other eight types are unbalanced types with higher score indicating worse constitution status.

(**II**) Gastroscopy and pathological examination verified the diagnosis of chronic superficial gastritis (CSG) , and two senior TCM practitioners confirmed Pi-Qi deficiency syndrome (PQDS).

***Diagnosis criteria*** Both endoscopic and pathological diagnosis criteria of CSG was defined according to the 2012 consensus on chronic gastritis in China .

*Endoscopic Diagnosis* Chronic superficial gastritis (CSG): red plaques (punctuates, patches, striae), coarse and uneven mucosa, hemorrhagic spots or plaques, edematous mucosa and exudates.

*Pathological Diagnosis* Two to five biopsies are required. The endoscopist should provide the department of pathology with information on the site of biopsy, endoscopy findings, and a brief history of the patients. The pathologist should report the histopathological changes in each biopsy specimen. The severity of *H. pylori* infection, chronic inflammation, active inflammation, atrophy, and intestinal metaplasia and dysplasia should be graded. Chronic gastritis (CG) with inherent gland atrophy revealed by biopsy can be diagnosed as atrophic gastritis, regardless of the number of biopsy specimens and degree of atrophy. Combining histopathological and endoscopic findings, clinicians can make a judgment on the scope and extent of the lesions.

***TCM syndrome diagnosis*** According to the consensus on diagnosis and treatment of chronic superficial gastritis with TCM, the CSG patients with PQDS were selected.

The diagnosis criteria of PQDS: primary symptoms include loss of appetite, fatigue, weakness, abdominal distention after eating or noontime, abnormal stool (loose stool, shapeless stool, sometimes loose stool or sometimes dry stool); secondary symptoms include lassitude, and no desire to talk, bland in mouth and absence of thirst, continuous mild abdominal pain, nausea and vomiting, fullness in the stomach, borborygmus, sallow complexion, edema, lack of strength in defecating, pale tongue, enlarged and teeth-printed tongue, thin-white tongue coating and weak pulse. A diagnosis of PQDS should include two of primary symptoms or one of primary symptoms with two of secondary symptoms simultaneously.

(**III**) Exclusion criteria

Patients would be excluded if they meet one of the following criteria: (1) Being under 18 years old or over 65 years old; (2) Being accompanied with gastric ulcer, duodenal ulcer, special types of gastritis or gastrointestinal hemorrhage; (3) With histopathological examination showing atrophic changes, dysplasia or suspected malignant changes in gastric mucosa; (4) Not PQDS according to syndrome differentiation in TCM; (5) Varied diagnosis of syndrome differentiation in TCM before treatment; (6) With unclear diagnosis of syndrome differentiation in TCM; (7) With history of gastric surgery; (8) Currently being accompanied with *H. pylori* infection; (9) With serious comorbidities of heart, lung, liver, kidney or blood system (such as cardiac function above grade II, value of alanine aminotransferase (ALT) and/or aspartate aminotransferase (AST) 1.5 times higher than the upper normal limit, creatinine (Cr) higher than the upper normal limit, etc.) or having a life-threatening illness (such as tumor or AIDS); (10) With psychiatric disorders or a history of alcohol or drug abuse; (11) Female patients preparing for a baby, pregnant or lactating.

(**IV**) Age, between 18 and 65 years. Subjects participated in the research signed the informed consent.

**The experimental design and route**


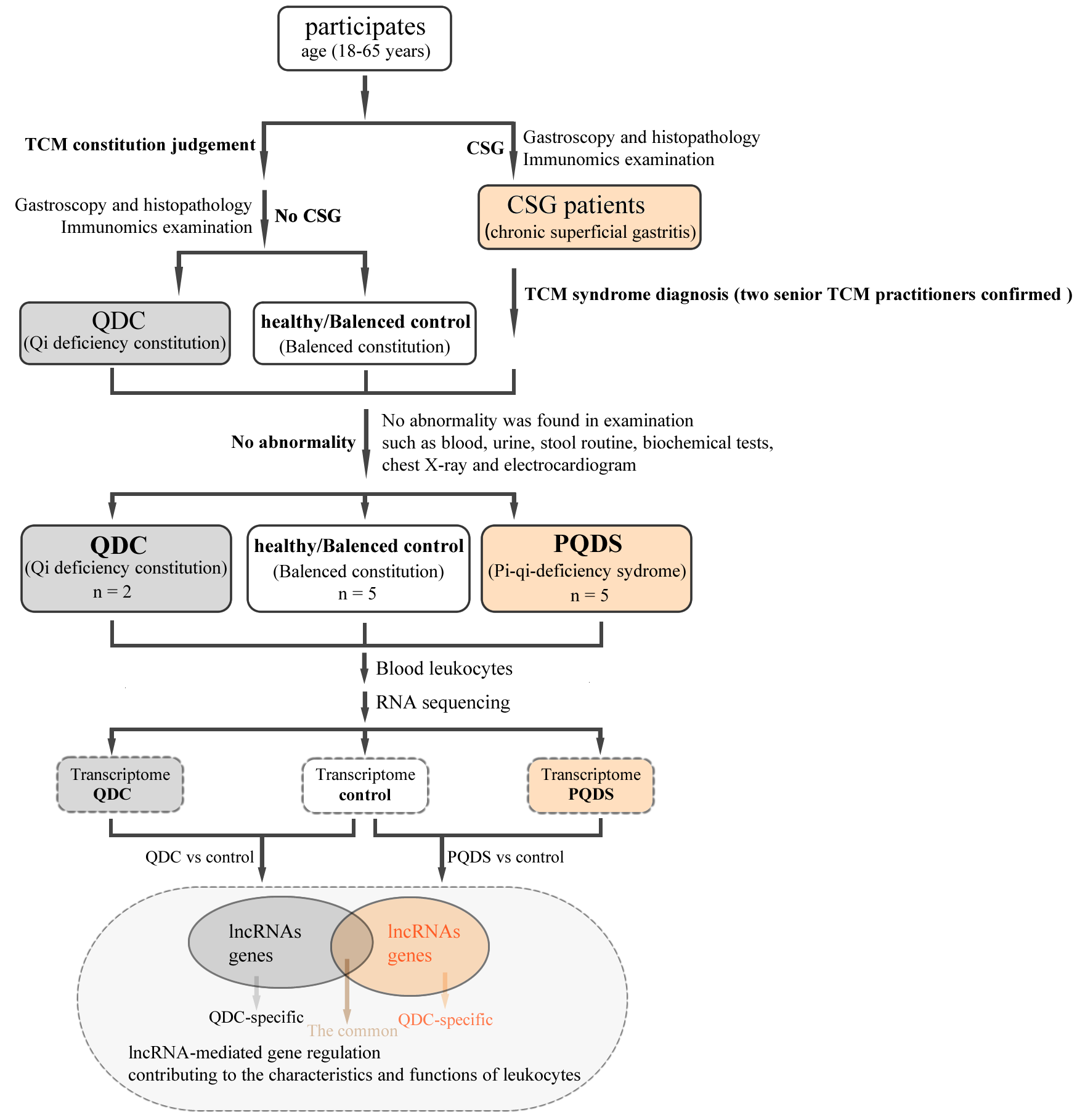


**Fig. S1** Experimental design and route of the study. TCM, traditional Chinese medicine; CSG, chronic superficial gastritis; QDC, Qi deficiency constitution; PQDS, Pi-qi-deficiency syndrome.

**Supplemental Tables**

**Table S1** List for leukocyte samples of the clinical subjects.

| **population** | **id** | **sample** | **Sex** | **Age** | **RNA-seq ^a^** | **SRA accession ^b^** | **Clinical diagnosis** | **TCM diagnosis** |
| --- | --- | --- | --- | --- | --- | --- | --- | --- |
| *Control population*  (BC) | 05 | bA_1 | Male | 26 | Yes | SRR10513209 | Health | Balanced constitution |
|  | 47 | bA_2 | Female | 28 | Yes | SRR10513208 | Health | Balanced constitution |
|  | 49 | bA_3 | Female | 24 | Yes | SRR10513204 | Health | Balanced constitution |
|  | 51 | bA_4 | Male | 25 | Yes | SRR10513203 | Health | Balanced constitution |
|  | 60 | bA_5 | Male | 26 | Yes | SRR10513202 | Health | Balanced constitution |
| *Case population 1*  (QDC) | 10 | bB_1 | Female | 24 | Yes | SRR10513205 | QDC | Qi deficiency constitution |
|  | 13 | bB_2 | Female | 26 | Yes | SRR10513201 | QDC | Qi deficiency constitution |
| *Case population 2*  (PQDS of CSG) | 03 | bD_1 | Female | 31 | Yes | SRR10513200 | CSG | Pi-qi-deficiency syndrome |
|  | 04 | bD_2a | Female | 33 | Yes | SRR10513199 | CSG | Pi-qi-deficiency syndrome |
|  | 09 | bD_4 | Male | 47 | Yes | SRR10513198 | CSG | Pi-qi-deficiency syndrome |
|  | 36 | bD_5 | Male | 45 | Yes | SRR10513207 | CSG | Pi-qi-deficiency syndrome |
|  | 39 | bD_6 | Female | 32 | Yes | SRR10513206 | CSG | Pi-qi-deficiency syndrome |

**Note:** TCM: traditional Chinese medicine; BC: balanced constitution; QDC: qi deficiency constitution; PQDS: Pi-qi-deficiency syndrome; CSG: chronic superficial gastritis.

^a^ LncRNA and mRNA expression is analyzed using the high throughput RNA sequencing (RNA-seq).

^b^ The RNA-seq reads are deposited in the National Center for Biotechnology Information (NCBI) Sequence Read Archive (SRA) (http://www.ncbi.nlm.nih.gov/sra) under the corresponding accession numbers.

**Table S2** Statistical results of the alignment of the cleaned raw reads to the reference genome

| **Sample** | **BC**  **(bA_1)** | **BC**  **(bA_2)** | **BC**  **(bA_3)** | **BC**  **(bA_4)** | **BC**  **(bA_5)** | **QDC**  **(bB_1)** | **QDC**  **(bB_2)** | **PQDS**  **(bD_1)** | **PQDS**  **(bD_2a)** | **PQDS**  **(bD_4)** | **PQDS**  **(bD_5)** | **PQDS**  **(bD_6)** |
| --- | --- | --- | --- | --- | --- | --- | --- | --- | --- | --- | --- | --- |
| Total reads | 93467646 | 90734668 | 92166436 | 92886594 | 93057334 | 93171850 | 97164614 | 96025516 | 97523158 | 96642800 | 95591480 | 96796864 |
| Total mapped reads | 91911163  (98.33%) | 88952043  (98.04%) | 90609013  (98.31%) | 91324730  (98.32%) | 91599595  (98.43%) | 91522398  (98.23%) | 95960230  (98.76%) | 95000860  (98.93%) | 96249605  (98.69%) | 94343423  (97.62%) | 94150430  (98.49%) | 95456827  (98.62%) |
| Multiple mapped | 5114736  (5.47%) | 4776164  (5.26%) | 4761925  (5.17%) | 5727822  (6.17%) | 6051355  (6.50%) | 5073076  (5.44%) | 5029140  (5.18%) | 5366292  (5.59%) | 6424706  (6.59%) | 5803749  (6.01%) | 5680418  (5.94%) | 5846260  (6.04%) |
| Uniquely mapped | 86796427  (92.86%) | 84175879  (92.77%) | 85847088  (93.14%) | 85596908  (92.15%) | 85548240  (91.93%) | 86449322  (92.78%) | 90931090  (93.58%) | 89634568  (93.34%) | 89824899  (92.11%) | 88539674  (91.62%) | 88470012  (92.55%) | 89610567  (92.58%) |
| Read-1 | 43677444  (46.73%) | 42487546  (46.83%) | 43240647  (46.92%) | 42895260  (46.18%) | 42881434  (46.08%) | 43508929  (46.70%) | 45491383  (46.82%) | 44847518  (46.70%) | 44967347  (46.11%) | 44276066  (45.81%) | 44244937  (46.29%) | 44810688  (46.29%) |
| Read-2 | 43118983  (46.13%) | 41688333  (45.95%) | 42606441  (46.23%) | 42701648  (45.97%) | 42666806  (45.85%) | 42940393  (46.09%) | 45439707  (46.77%) | 44787050  (46.64%) | 44857552  (46.00%) | 44263608  (45.80%) | 44225075  (46.26%) | 44799879  (46.28%) |
| Reads map to '+' | 43358599  (46.39%) | 42008972  (46.30%) | 42876081  (46.52%) | 42795253  (46.07%) | 42776098  (45.97%) | 43176246  (46.34%) | 45454612  (46.78%) | 44815565  (46.67%) | 44919393  (46.06%) | 44274629  (45.81%) | 44238288  (46.28%) | 44814295  (46.30%) |
| Reads map to '-' | 43437828  (46.47%) | 42166907  (46.47%) | 42971007  (46.62%) | 42801655  (46.08%) | 42772142  (45.96%) | 43273076  (46.44%) | 45476478  (46.80%) | 44819003  (46.67%) | 44905506  (46.05%) | 44265045  (45.80%) | 44231724  (46.27%) | 44796272  (46.28%) |
| Non-splice reads | 75999370  (81.31%) | 75204311  (82.88%) | 75455591  (81.87%) | 73558960  (79.19%) | 73388075  (78.86%) | 76703126  (82.32%) | 80934201  (83.30%) | 78594344  (81.85%) | 79600655  (81.62%) | 78038653  (80.75%) | 77276506  (80.84%) | 78526418  (81.12%) |
| Splice reads | 10797057  (11.55%) | 8971568  (9.89%) | 10391497  (11.27%) | 12037948  (12.96%) | 12160165  (13.07%) | 9746196  (10.46%) | 9996889  (10.29%) | 11040224  (11.50%) | 10224244  (10.48%) | 10501021  (10.87%) | 11193506  (11.71%) | 11084149  (11.45%) |
| Reads mapped  in proper pairs | 84483498  (90.39%) | 81800548  (90.15%) | 83729026  (90.85%) | 83304466  (89.68%) | 83274564  (89.49%) | 83871424  (90.02%) | 89055938  (91.65%) | 88002192  (91.64%) | 86585824  (88.78%) | 85598920  (88.57%) | 86257154  (90.24%) | 87391388  (90.28%) |

**Note:** BC: balanced constitution; QDC: qi deficiency constitution; PQDS: Pi-qi-deficiency syndrome.

**Table S3** Differential genes identified in the QDC population compared with the BC control population

| **Gene name** | **Base Mean ^a^**  **(BC, n=5)** | **Base Mean ^a^**  **(QDC, n=2)** | **Fold Change ^b^** | **log_2_ (Fold Change)** | ***P*-value** | **Padj ^c^** | **up_down** |
| --- | --- | --- | --- | --- | --- | --- | --- |
| AATK | 1580.861131 | 3801.094895 | 2.4044458 | 1.265704402 | 0.027607505 | 1 | Up |
| ACTL7B | 0.788836547 | 10.08472826 | 12.784307 | 3.676302007 | 0.022443011 | 1 | Up |
| ADAMTS1 | 245.3266597 | 65.15502296 | 0.2655848 | -1.912755711 | 0.000416667 | 1 | Down |
| ADAMTSL5 | 8.61320873 | 41.4586852 | 4.8133845 | 2.267051666 | 0.000502396 | 0.641272 | Up |
| AHI1 | 450.4981075 | 220.3202295 | 0.4890592 | -1.031919078 | 0.044745503 | 0.201728 | Down |
| ALAS2 | 282.0650829 | 70.81670851 | 0.2510651 | -1.993866389 | 0.003175085 | 1 | Down |
| ANKHD1-EIF4EBP3 | 25.08586205 | 57.23737077 | 2.2816585 | 1.190082887 | 0.006282441 | 1 | Up |
| APOBEC3A_B | 38.55332565 | 88.68322303 | 2.3002743 | 1.20180589 | 0.037447694 | 0.615736 | Up |
| AR | 18.88400085 | 45.49733133 | 2.4093057 | 1.268617473 | 0.017844968 | 0.994032 | Up |
| ARHGEF25 | 16.07905777 | 1.135621577 | 0.0706274 | -3.823628796 | 0.0209183 | 0.666048 | Down |
| ARPIN | 470.9184613 | 202.6365562 | 0.4303007 | -1.216582817 | 0.048503936 | 1 | Down |
| ASPM | 158.9435681 | 49.99169645 | 0.3145248 | -1.668754247 | 0.000192142 | 0.646904 | Down |
| ATF6B | 62.23759322 | 13.24363526 | 0.2127916 | -2.232487088 | 3.68E-05 | 1 | Down |
| B3GALT2 | 143.892482 | 70.0015129 | 0.4864849 | -1.039533209 | 0.0122204 | 1 | Down |
| B4GAT1 | 58.36903459 | 26.4714211 | 0.4535182 | -1.14076756 | 0.035148017 | 1 | Down |
| BIVM | 111.1365642 | 54.51833334 | 0.4905526 | -1.027520181 | 0.038857273 | 1 | Down |
| C14orf183 | 136.8548901 | 284.2428156 | 2.076965 | 1.054476898 | 0.00496293 | 0.68274 | Up |
| CACNA1A | 19.47814827 | 50.05566706 | 2.569837 | 1.361676882 | 0.005609841 | 0.924676 | Up |
| CADM4 | 63.11293076 | 127.8061216 | 2.0250386 | 1.017949415 | 0.000153763 | 0.876027 | Up |
| CATSPERG | 117.7192213 | 278.0942851 | 2.3623524 | 1.240224193 | 0.017341005 | 0.80621 | Up |
| CCNB1 | 66.20778433 | 27.16717311 | 0.410332 | -1.285136398 | 0.009447101 | 0.813198 | Down |
| CFAP45 | 221.3678186 | 503.9074895 | 2.2763358 | 1.186713394 | 0.001833815 | 0.360367 | Up |
| CFH | 259.9820164 | 92.13820895 | 0.3544022 | -1.496540372 | 0.043367157 | 0.239246 | Down |
| CHI3L2 | 171.6178705 | 83.75691305 | 0.4880431 | -1.034919611 | 0.001216229 | 0.53072 | Down |
| CLDN22 | 1.52461773 | 13.73955073 | 9.0118004 | 3.171815366 | 0.01686168 | 0.897759 | Up |
| CLDN24 | 0.564096047 | 9.708829306 | 17.211305 | 4.105284612 | 0.033106394 | 0.981642 | Up |
| CLDN9 | 49.93555685 | 127.982184 | 2.562947 | 1.357803626 | 0.009735525 | 0.203351 | Up |
| CLEC18A | 62.11956325 | 137.3711602 | 2.2113993 | 1.144959564 | 0.003744909 | 0.435923 | Up |
| CLIC5 | 141.1029873 | 64.22716264 | 0.4551793 | -1.135493063 | 0.015924991 | 0.478423 | Down |
| CLK2 | 11.41694005 | 51.95893598 | 4.5510387 | 2.186195854 | 9.21E-05 | 0.481961 | Up |
| CNIH2 | 10.24190212 | 36.31611634 | 3.5458371 | 1.826126252 | 0.03073751 | 0.775546 | Up |
| CNOT3 | 93.36690547 | 202.5663796 | 2.1695737 | 1.117411574 | 0.031359241 | 0.191593 | Up |
| COL26A1 | 0 | 5.542241952 | +∞ | +∞ | 0.000384346 | 1 | Up |
| COL27A1 | 0 | 5.798124397 | +∞ | +∞ | 0.004433332 | 1 | Up |
| CORIN | 98.56223273 | 26.27158455 | 0.2665482 | -1.90753172 | 0.000111959 | 0.07856 | Down |
| CSF2RA | 226.7695924 | 850.7708506 | 3.7516972 | 1.907543402 | 0.001176965 | 1.61E-07 | Up |
| CXCR6 | 342.4737063 | 122.9919034 | 0.359128 | -1.477429883 | 0.00484617 | 0.689701 | Down |
| CYB5D1 | 232.1604614 | 508.2252193 | 2.1891119 | 1.130345675 | 0.026368958 | 1 | Up |
| DDR2 | 31.56772664 | 10.26871538 | 0.3252916 | -1.620194657 | 0.008661237 | 1 | Down |
| DDX39B | 85.02392433 | 383.7314388 | 4.5132172 | 2.174156215 | 0.017076561 | 8.40E-10 | Up |
| DDX3Y | 3148.078405 | 2.583171498 | 0.0008206 | -10.25111233 | 0.019944307 | 0.07856 | Down |
| DNM1 | 17.86984399 | 48.52829711 | 2.7156531 | 1.441299197 | 2.89E-07 | 0.813198 | Up |
| DPCD | 20.86830436 | 4.286603864 | 0.2054122 | -2.283406375 | 0.046576734 | 0.996763 | Down |
| ECH1 | 4.25447954 | 65.97814328 | 15.507924 | 3.954933619 | 0.049558291 | 0.929174 | Up |
| EIF1AY | 618.9700189 | 0.567810789 | 0.0009173 | -10.09024355 | 0.00972993 | 0.191593 | Down |
| ELF3 | 16.06972978 | 63.81106721 | 3.9708861 | 1.989460993 | 0.025433044 | 0.19859 | Up |
| FAM110B | 53.44446614 | 24.71194283 | 0.4623854 | -1.112832134 | 0.01345432 | 1 | Down |
| FAM127B | 57.05022534 | 127.5825109 | 2.2363191 | 1.161126087 | 0.020090255 | 0.45442 | Up |
| FAM153B | 61.11317682 | 150.1109553 | 2.4562781 | 1.296473887 | 0.021122059 | 0.641272 | Up |
| FAM60A | 38.10667323 | 14.12337439 | 0.3706273 | -1.431958842 | 0.021064193 | 1 | Down |
| FAM72D | 12.47723974 | 0.503840177 | 0.0403807 | -4.63018883 | 0.02990151 | 0.868804 | Down |
| FAT4 | 159.366017 | 45.69716788 | 0.2867435 | -1.802167363 | 0.033924527 | 0.900559 | Down |
| FLJ44635 | 14.30481029 | 47.48834499 | 3.3197466 | 1.731073113 | 0.009574588 | 1 | Up |
| FOSB | 158.4750893 | 320.9676755 | 2.025351 | 1.018171931 | 0.003732558 | 0.123895 | Up |
| GOLGA8N | 303.9365002 | 117.218126 | 0.3856665 | -1.374574263 | 0.046588968 | 0.05094 | Down |
| GPSM1 | 45.38668809 | 95.04915822 | 2.0942078 | 1.066404633 | 0.03858481 | 0.801306 | Up |
| GRIK4 | 1.879209913 | 20.40948954 | 10.860676 | 3.441041966 | 0.044158731 | 1 | Up |
| GRM2 | 29.27323836 | 61.97176891 | 2.117011 | 1.082028796 | 0.04104178 | 1 | Up |
| GSTM3 | 60.66471417 | 135.5318619 | 2.2341136 | 1.159702534 | 0.015899465 | 0.581475 | Up |
| GTPBP6 | 11.22537837 | 92.99417395 | 8.2842797 | 3.05037626 | 0.004471628 | 0.615736 | Up |
| GZMK | 902.6646009 | 416.3678127 | 0.4612652 | -1.116331486 | 0.011241658 | 0.106365 | Down |
| H1FNT | 1.104329803 | 13.17966465 | 11.934537 | 3.577070666 | 0.010968489 | 0.858887 | Up |
| HBD | 13.3428794 | 0.503840177 | 0.037761 | -4.726960054 | 0.046811576 | 0.740272 | Down |
| HBG2 | 48.62469638 | 7.693468596 | 0.1582214 | -2.659983153 | 0.024550399 | 0.385101 | Down |
| HEPH | 23.06688762 | 78.18239934 | 3.3893779 | 1.761020503 | 6.72E-05 | 1 | Up |
| HIP1 | 2446.619364 | 6630.783352 | 2.7101818 | 1.438389649 | 0.013352287 | 0.301371 | Up |
| HIST1H3J | 109.1498618 | 49.91980113 | 0.457351 | -1.128626211 | 1.02E-06 | 0.858887 | Down |
| HLA-G | 0.585582844 | 36.90770126 | 63.027293 | 5.977904788 | 0.003611977 | 1 | Up |
| HSD17B13 | 50.10039718 | 117.7853639 | 2.3509866 | 1.233266334 | 0.002434602 | 0.744835 | Up |
| IGFBP3 | 53.20580392 | 15.31504187 | 0.2878453 | -1.796634319 | 0.02687857 | 0.481961 | Down |
| ITGA2 | 66.02979469 | 28.24674879 | 0.4277879 | -1.225032338 | 0.016114482 | 0.846668 | Down |
| ITGAD | 63.65856524 | 196.880347 | 3.092755 | 1.628892559 | 0.021704131 | 0.07856 | Up |
| JAG2 | 10.68181925 | 32.01366305 | 2.9970235 | 1.583530384 | 0.01440069 | 1 | Up |
| KCNC3 | 70.24257632 | 144.8325205 | 2.0618908 | 1.043967914 | 0.000570639 | 1 | Up |
| KCNK7 | 31.47241095 | 97.47211673 | 3.0970655 | 1.630901874 | 0.001315161 | 0.187674 | Up |
| KCNT1 | 3.365176599 | 23.88032489 | 7.0963066 | 2.827068342 | 0.039688594 | 0.54927 | Up |
| KDM5D | 2237.159994 | 2.271243155 | 0.0010152 | -9.943970555 | 0.019482464 | 0.084488 | Down |
| KIAA1324L | 341.1408195 | 130.9095556 | 0.3837405 | -1.381796983 | 0.037366445 | 0.300615 | Down |
| KIAA1731NL | 347.0735286 | 862.6614596 | 2.4855294 | 1.313553169 | 0.041158671 | 0.574687 | Up |
| KIF2C | 26.72527607 | 3.782763687 | 0.1415425 | -2.820692293 | 0.033764847 | 0.981642 | Down |
| KLHL33 | 12.43470602 | 56.64578585 | 4.5554584 | 2.187596231 | 0.045856751 | 0.733642 | Up |
| KLRB1 | 1864.374952 | 798.6528475 | 0.4283757 | -1.223051591 | 0.00373347 | 0.858887 | Down |
| KLRC1 | 606.8103026 | 276.9258188 | 0.4563631 | -1.131746013 | 0.009208501 | 0.897332 | Down |
| KLRG1 | 1849.849893 | 816.7107009 | 0.4415011 | -1.179511171 | 0.022050726 | 0.00096 | Down |
| LENG8 | 149.3585206 | 475.7196512 | 3.1850855 | 1.671332079 | 0.037964971 | 1 | Up |
| LOC101060181 | 20.37089029 | 45.25729831 | 2.2216652 | 1.151641431 | 0.000602542 | 1 | Up |
| LOC101927789 | 18.25367375 | 85.32390657 | 4.6743416 | 2.22476317 | 0.038759723 | 0.744835 | Up |
| LOC101927853 | 2.384975614 | 15.05915942 | 6.3141775 | 2.658594823 | 0.007068943 | 0.981642 | Up |
| LOC102724378 | 40.09058637 | 84.97235467 | 2.1195089 | 1.083730025 | 0.026775862 | 0.809057 | Up |
| LOC102725016 | 48.23957376 | 18.48187358 | 0.3831268 | -1.384106144 | 0.018768255 | 0.897759 | Down |
| LOC105369154 | 10.99052208 | 34.80459581 | 3.1667828 | 1.663017901 | 0.020605077 | 0.901461 | Up |
| LOC105371430 | 29.09835532 | 63.3394988 | 2.1767381 | 1.122167839 | 0.005535988 | 0.901309 | Up |
| LOC105372343 | 7.934587598 | 26.84732005 | 3.383581 | 1.758550937 | 0.045634007 | 1 | Up |
| LOC105376526 | 0.368048579 | 14.67533576 | 39.873366 | 5.3173535 | 0.000784877 | 1 | Up |
| LOC105376684 | 27.42723066 | 69.48125039 | 2.5332944 | 1.341014757 | 0.012789944 | 0.742791 | Up |
| LOC105377808 | 16.51308682 | 53.64651892 | 3.2487275 | 1.699874728 | 0.012218037 | 0.481961 | Up |
| LOC390937 | 8.144158333 | 24.64004751 | 3.0254873 | 1.597167523 | 0.0407678 | 1 | Up |
| LTB | 8.010997562 | 211.6575582 | 26.420874 | 4.723606294 | 3.60E-05 | 1 | Up |
| MADCAM1 | 10.46471866 | 0.503840177 | 0.0481466 | -4.376423544 | 0.012259478 | 1 | Down |
| MAP1LC3B2 | 34.36696615 | 69.23329266 | 2.0145302 | 1.010443464 | 0.00205443 | 1 | Up |
| MATN2 | 26.72306098 | 5.862095008 | 0.2193647 | -2.188597015 | 0.007810131 | 0.846668 | Down |
| ME3 | 88.70768013 | 34.9166876 | 0.3936152 | -1.345142312 | 0.001904304 | 0.549825 | Down |
| MGC57346-CRHR1 | 0.377630309 | 10.33268599 | 27.361909 | 4.774096962 | 0.006701805 | 0.775546 | Up |
| MOK | 30.98579684 | 91.49850284 | 2.9529175 | 1.562141068 | 0.003284896 | 0.775546 | Up |
| MSH5 | 2.774857118 | 14.17942029 | 5.1099641 | 2.353313159 | 0.008478584 | 1 | Up |
| MXRA8 | 22.08560783 | 56.05362801 | 2.5380161 | 1.343701215 | 5.03E-08 | 0.970749 | Up |
| MYADM | 2921.460227 | 5929.630025 | 2.0296802 | 1.021252443 | 0.025829409 | 0.07856 | Up |
| NDE1 | 288.6493085 | 934.4241695 | 3.2372299 | 1.694759822 | 0.00114488 | 0.80621 | Up |
| NEO1 | 380.1956898 | 131.5254875 | 0.3459416 | -1.531399779 | 9.14E-14 | 0.002815 | Down |
| NFE2L3 | 276.2153615 | 96.35234458 | 0.3488305 | -1.519401879 | 0.011937589 | 0.481961 | Down |
| NFXL1 | 2298.595461 | 1148.877817 | 0.4998173 | -1.000527207 | 3.68E-05 | 0.39242 | Down |
| NSG1 | 250.4383058 | 114.1469638 | 0.4557888 | -1.133562761 | 0.002414171 | 0.987252 | Down |
| NTHL1 | 68.77278404 | 28.41488648 | 0.4131705 | -1.275190798 | 0.007970265 | 0.846668 | Down |
| NUDT11 | 11.22944331 | 0.503840177 | 0.0448678 | -4.478176428 | 0.042086775 | 0.91434 | Down |
| NUP210L | 32.85268224 | 73.94391666 | 2.2507726 | 1.170420299 | 0.038017781 | 0.813198 | Up |
| NYX | 3.950507915 | 31.33376046 | 7.9315777 | 2.987607865 | 0.004147731 | 0.204434 | Up |
| OBSCN | 1793.858589 | 3634.053975 | 2.0258308 | 1.018513681 | 2.84E-05 | 0.311919 | Up |
| OLR1 | 13.68587803 | 39.03515377 | 2.8522214 | 1.512085958 | 0.017526491 | 1 | Up |
| OPLAH | 150.2951009 | 350.8448155 | 2.3343729 | 1.22303506 | 0.015563101 | 0.894838 | Up |
| OR1J2 | 1.670933254 | 12.3480467 | 7.3899102 | 2.885556834 | 0.023374705 | 1 | Up |
| OTX1 | 28.31278687 | 58.62887479 | 2.0707561 | 1.050157606 | 0.002120487 | 1 | Up |
| PABPN1L | 34.38564112 | 70.61687197 | 2.0536733 | 1.038206672 | 0.034345061 | 1 | Up |
| PBX2 | 99.94865884 | 230.9721956 | 2.3109084 | 1.208460077 | 0.035747826 | 1 | Up |
| PDE9A | 37.79232926 | 119.601461 | 3.164702 | 1.662069668 | 0.011315288 | 0.481961 | Up |
| PDGFRA | 19.15467279 | 2.143301932 | 0.1118945 | -3.159789375 | 0.020647361 | 0.740272 | Down |
| PDZD2 | 40.13670965 | 80.72594728 | 2.0112747 | 1.008110114 | 0.004558355 | 0.919814 | Up |
| PPP1R10 | 21.45055756 | 50.11963768 | 2.3365191 | 1.224360837 | 0.020224185 | 1 | Up |
| PRSS21 | 15.04573819 | 60.00452939 | 3.9881413 | 1.995716514 | 0.013547628 | 0.208404 | Up |
| PTPRM | 380.2291448 | 91.21827334 | 0.2399034 | -2.059474353 | 0.017786364 | 1 | Down |
| PUF60 | 73.58984691 | 23.38440942 | 0.3177668 | -1.65395974 | 0.018381251 | 0.6709 | Down |
| PVRL2 | 50.30272722 | 138.7473877 | 2.7582478 | 1.463752085 | 0.000194029 | 0.98566 | Up |
| RAD51AP1 | 39.30665408 | 12.60392915 | 0.3206564 | -1.640900012 | 9.94E-05 | 0.878422 | Down |
| RAG1 | 31.68361055 | 9.077047906 | 0.2864903 | -1.803441673 | 0.048482229 | 0.900559 | Down |
| RASA4 | 48.33283158 | 108.5084794 | 2.2450263 | 1.166732364 | 0.017494426 | 0.615736 | Up |
| RASA4B | 56.37758247 | 114.8664899 | 2.0374497 | 1.026764461 | 0.02866412 | 1 | Up |
| RCN3 | 571.7425923 | 1503.139617 | 2.6290496 | 1.394541345 | 0.000225865 | 0.239246 | Up |
| REC8 | 751.558228 | 1547.31734 | 2.0588123 | 1.041812322 | 0.000223647 | 0.80621 | Up |
| RNF225 | 2.809462808 | 18.78587721 | 6.6866439 | 2.741282278 | 0.024356603 | 0.801306 | Up |
| ROR2 | 28.5651884 | 9.460871574 | 0.3312028 | -1.594213044 | 0.001280239 | 1 | Down |
| RPS4Y1 | 1753.452858 | 0.503840177 | 0.0002873 | -11.76494485 | 0.017475203 | 0.05094 | Down |
| S100P | 700.589941 | 62.87528218 | 0.0897462 | -3.478005399 | 0.000336441 | 0.801306 | Down |
| SDK2 | 155.8311615 | 385.867389 | 2.4761889 | 1.308121364 | 0.027607505 | 0.385101 | Up |
| SFN | 37.30161023 | 99.18347381 | 2.6589596 | 1.410861846 | 0.022443011 | 0.689701 | Up |
| SGCD | 85.83941635 | 23.13645169 | 0.2695318 | -1.891472644 | 0.000416667 | 0.385101 | Down |
| SHANK1 | 8.325628348 | 33.52518358 | 4.0267452 | 2.009614168 | 0.000502396 | 0.662282 | Up |
| SLC43A2 | 268.9383696 | 574.2922531 | 2.1354047 | 1.094509501 | 0.044745503 | 0.462168 | Up |
| SLC4A10 | 1040.81433 | 292.2895548 | 0.2808278 | -1.832242551 | 0.003175085 | 0.721854 | Down |
| SLC7A8 | 34.72462461 | 155.7494396 | 4.4852735 | 2.165195969 | 0.006282441 | 0.000209 | Up |
| SLC7A9 | 5.010701959 | 21.80891828 | 4.3524677 | 2.121833582 | 0.037447694 | 0.981642 | Up |
| SLC9A1 | 748.3422164 | 1548.981149 | 2.0698834 | 1.049549517 | 0.017844968 | 0.296989 | Up |
| SLX1B | 11.97626806 | 138.179004 | 11.537735 | 3.528288095 | 0.0209183 | 8.40E-10 | Up |
| SPDYE18 | 39.61558306 | 85.72415258 | 2.1638998 | 1.113633702 | 0.048503936 | 0.797691 | Up |
| SPEG | 41.63391643 | 128.2544887 | 3.080529 | 1.623178138 | 0.000192142 | 0.05094 | Up |
| SPON1 | 80.47282098 | 27.27926491 | 0.3389873 | -1.560696839 | 3.68E-05 | 0.39242 | Down |
| SUSD2 | 29.42922537 | 73.95976609 | 2.5131401 | 1.329491094 | 0.0122204 | 0.692787 | Up |
| SUSD4 | 49.83830969 | 20.40948954 | 0.4095141 | -1.288015039 | 0.035148017 | 1 | Down |
| SYT15 | 0.353768017 | 7.813485107 | 22.086465 | 4.465090657 | 0.038857273 | 1 | Up |
| TAF4 | 133.8124946 | 281.5651204 | 2.1041766 | 1.073255795 | 0.00496293 | 0.521671 | Up |
| TARP | 2101.720455 | 839.6801607 | 0.3995204 | -1.323658987 | 0.005609841 | 0.05094 | Down |
| TEAD2 | 5.650305978 | 25.47166545 | 4.5080152 | 2.172492392 | 0.000153763 | 0.858887 | Up |
| TGM3 | 100.1430708 | 214.4337874 | 2.1412743 | 1.098469643 | 0.017341005 | 0.846668 | Up |
| TIAM2 | 234.4992595 | 507.4660696 | 2.1640412 | 1.113727996 | 0.009447101 | 0.943039 | Up |
| TLR3 | 87.99737689 | 31.89364654 | 0.3624386 | -1.464191463 | 0.001833815 | 0.385101 | Down |
| TMC8 | 5232.348913 | 10633.13705 | 2.0321919 | 1.023036639 | 0.043367157 | 1 | Up |
| TMEM119 | 14.58169737 | 39.53899395 | 2.7115495 | 1.439117498 | 0.001216229 | 1 | Up |
| TMEM171 | 19.43977386 | 0.503840177 | 0.025918 | -5.269901455 | 0.01686168 | 0.775546 | Down |
| TMEM88 | 135.8460793 | 282.5473079 | 2.0799077 | 1.056519515 | 0.033106394 | 0.900559 | Up |
| TMSB4Y | 42.40758938 | 0 | 0 | -∞ | 0.009735525 | 0.552393 | Down |
| TNNT3 | 69.41539249 | 174.9825382 | 2.5208031 | 1.333883447 | 0.003744909 | 0.897759 | Up |
| TRPC3 | 10.4269691 | 53.30289172 | 5.1120216 | 2.353893944 | 0.015924991 | 0.809057 | Up |
| TSSK6 | 43.29323685 | 89.52276569 | 2.0678233 | 1.048112939 | 9.21E-05 | 0.868498 | Up |
| UNC5B | 15.81827792 | 1.007680355 | 0.0637035 | -3.972482566 | 0.03073751 | 0.876027 | Down |
| USP9Y | 2322.278437 | 3.782763687 | 0.0016289 | -9.261884591 | 0.031359241 | 0.123895 | Down |
| UTY | 3935.19795 | 4.350574476 | 0.0011056 | -9.821014578 | 0.000384346 | 0.07856 | Down |
| VARS2 | 42.86807207 | 87.9716216 | 2.0521478 | 1.03713467 | 0.004433332 | 1 | Up |
| VSTM1 | 1.182255214 | 15.87492795 | 13.427666 | 3.747136634 | 0.000111959 | 0.858887 | Up |
| YTHDC1 | 292.163139 | 744.4549401 | 2.5480796 | 1.349410358 | 0.001176965 | 1 | Up |
| ZFP57 | 0 | 14.30736151 | +∞ | +∞ | 0.00484617 | 0.133922 | Up |
| ZFY | 601.5546505 | 0.567810789 | 0.0009439 | -10.04906983 | 0.026368958 | 0.133922 | Down |
| ZMYND10 | 39.48871584 | 80.24588124 | 2.0321218 | 1.022986893 | 0.008661237 | 0.967388 | Up |
| ZNF215 | 24.52373093 | 2.079331321 | 0.0847885 | -3.55998692 | 0.017076561 | 0.31091 | Down |
| ZNF695 | 8.2230915 | 29.82223993 | 3.6266458 | 1.858635834 | 0.019944307 | 0.858887 | Up |
| ZSWIM5 | 80.01896534 | 22.37672907 | 0.2796428 | -1.83834281 | 2.89E-07 | 0.16926 | Down |

**Note:** QDC: Qi deficiency constitution; BC: balanced constituion

^a^ normalized mean count value; ^b^ normalized mean count value (PQDS of CSG) / normalized mean count value (BC); ^c^ *P*-value adjusted by false discovery rate (FDR).

**Table S4** Differential genes identified in the PQDS population compared with the BC control population

| **Gene name** | **Base Mean ^a^**  **(BC, n=5)** | **Base Mean ^a^**  **(PQDS of CSG, n=5)** | **Fold Change ^b^** | **log_2_ (Fold Change)** | ***P*-value ^c^** | **padj** | **up_down** |
| --- | --- | --- | --- | --- | --- | --- | --- |
| ADAM12 | 176.3018 | 77.66999 | 0.440551 | -1.18262 | 0.030509 | 1 | Down |
| ADAMTS2 | 4.411645 | 0.168894 | 0.038284 | -4.70713 | 0.027887 | 1 | Down |
| ADAMTSL5 | 8.955645 | 36.47797 | 4.073181 | 2.026156 | 0.009895 | 1 | Up |
| AKR1C1 | 6.234749 | 16.72008 | 2.681757 | 1.423178 | 0.033491 | 1 | Up |
| ARMCX1 | 33.46875 | 14.90526 | 0.445348 | -1.16699 | 0.016576 | 1 | Down |
| ASMTL | 80.66661 | 26.75332 | 0.331653 | -1.59225 | 0.029565 | 1 | Down |
| B3GALNT2 | 34.66762 | 9.45325 | 0.272682 | -1.87471 | 0.022699 | 1 | Down |
| BRSK1 | 36.42845 | 11.72242 | 0.321793 | -1.6358 | 0.0009 | 1 | Down |
| C14orf132 | 21.64143 | 45.87111 | 2.119597 | 1.08379 | 0.007093 | 1 | Up |
| C4A | 1.642175 | 8.29495 | 5.051198 | 2.336626 | 0.035945 | 1 | Up |
| C4B | 0.615271 | 5.702153 | 9.267709 | 3.212213 | 0.03499 | 1 | Up |
| C4BPA | 23.99381 | 284.7853 | 11.86912 | 3.569141 | 0.03258 | 1 | Up |
| C4BPB | 2.601223 | 10.7754 | 4.142437 | 2.05048 | 0.030296 | 1 | Up |
| CACNA1C | 5.422877 | 15.91922 | 2.935567 | 1.553639 | 0.026082 | 1 | Up |
| CAPN8 | 5.356675 | 14.46966 | 2.701239 | 1.433621 | 0.048443 | 1 | Up |
| CD300LD | 36.97509 | 14.6723 | 0.396816 | -1.33346 | 0.013007 | 1 | Down |
| CEP170 | 167.6116 | 75.66352 | 0.451422 | -1.14745 | 0.047076 | 1 | Down |
| CLDN7 | 18.33063 | 5.187342 | 0.282988 | -1.82119 | 0.00791 | 1 | Down |
| CLEC4C | 240.6699 | 102.2334 | 0.424787 | -1.23519 | 0.009397 | 1 | Down |
| COL10A1 | 3.025039 | 10.63941 | 3.517115 | 1.814393 | 0.046868 | 1 | Up |
| COL26A1 | 0 | 4.378854 | +∞ | +∞ | 0.013492 | 1 | Up |
| COL27A1 | 0 | 4.131851 | +∞ | +∞ | 0.016933 | 1 | Up |
| COL2A1 | 0 | 9.466327 | +∞ | +∞ | 7.62E-05 | 0.894919 | Up |
| COL5A3 | 214.0656 | 37.41568 | 0.174786 | -2.51634 | 0.00686 | 1 | Down |
| CORIN | 102.498 | 46.70555 | 0.455673 | -1.13393 | 0.000298 | 1 | Down |
| CPLX2 | 0.451986 | 5.026945 | 11.1219 | 3.475331 | 0.035614 | 1 | Up |
| CPXM1 | 14.23679 | 2.573899 | 0.180792 | -2.4676 | 0.018003 | 1 | Down |
| CRB3 | 3.683726 | 14.833 | 4.026629 | 2.009573 | 0.010244 | 1 | Up |
| CYGB | 32.30242 | 16.12446 | 0.499172 | -1.00239 | 0.043113 | 1 | Down |
| DHRS2 | 0.431083 | 7.566861 | 17.55312 | 4.133656 | 0.028653 | 1 | Up |
| DHX16 | 49.26941 | 9.447953 | 0.191761 | -2.38262 | 0.005326 | 1 | Down |
| DPYSL3 | 0.398746 | 6.028586 | 15.11885 | 3.918276 | 0.020832 | 1 | Up |
| DYRK1B | 4.309098 | 14.12883 | 3.278837 | 1.713184 | 0.032587 | 1 | Up |
| FAM171B | 22.21019 | 8.220003 | 0.370101 | -1.43401 | 0.018265 | 1 | Down |
| FAM83D | 42.13451 | 19.66295 | 0.466671 | -1.09952 | 0.011638 | 1 | Down |
| GAL3ST1 | 5.064077 | 14.57829 | 2.878766 | 1.52545 | 0.042542 | 1 | Up |
| GGACT | 332.6328 | 157.5148 | 0.47354 | -1.07844 | 0.001709 | 1 | Down |
| GJB7 | 6.052368 | 0.812944 | 0.134318 | -2.89627 | 0.04575 | 1 | Down |
| GLB1L2 | 40.68778 | 83.59938 | 2.054656 | 1.038897 | 0.003783 | 1 | Up |
| GNAO1 | 68.28416 | 140.2762 | 2.0543 | 1.038647 | 0.009041 | 1 | Up |
| GPM6B | 7.742591 | 1.349066 | 0.17424 | -2.52086 | 0.032111 | 1 | Down |
| HOXA3 | 8.440324 | 1.4924 | 0.176818 | -2.49966 | 0.025536 | 1 | Down |
| IL23R | 86.73718 | 42.05798 | 0.48489 | -1.04427 | 0.032361 | 1 | Down |
| KCNJ14 | 18.08029 | 37.68799 | 2.08448 | 1.059687 | 0.020853 | 1 | Up |
| KIR2DL1 | 6.128575 | 0 | 0 | -∞ | 0.001333 | 1 | Down |
| LILRB3 | 83.51951 | 703.9589 | 8.428676 | 3.075306 | 0.002358 | 1 | Up |
| LOC100130520 | 484.8917 | 218.9154 | 0.451473 | -1.14729 | 0.013442 | 1 | Down |
| LOC101930332 | 15.10296 | 4.142548 | 0.274287 | -1.86624 | 0.015032 | 1 | Down |
| LOC102724994 | 10.67759 | 1.181215 | 0.110626 | -3.17624 | 0.019488 | 1 | Down |
| LOC105371430 | 30.26026 | 62.8178 | 2.075918 | 1.053749 | 0.024396 | 1 | Up |
| LOC105376526 | 0.383463 | 4.92895 | 12.85378 | 3.68412 | 0.040753 | 1 | Up |
| LOC390937 | 8.467089 | 21.17389 | 2.500728 | 1.322348 | 0.030656 | 1 | Up |
| LRFN3 | 40.9425 | 100.3976 | 2.45216 | 1.294053 | 0.018202 | 1 | Up |
| LRRC66 | 14.1546 | 28.56444 | 2.018032 | 1.012949 | 0.045015 | 1 | Up |
| MATN2 | 27.80933 | 12.6111 | 0.453485 | -1.14087 | 0.029328 | 1 | Down |
| MEIS3 | 0 | 3.729842 | +∞ | +∞ | 0.025243 | 1 | Up |
| MLF1 | 10.05256 | 22.70006 | 2.258137 | 1.175133 | 0.041672 | 1 | Up |
| MSH5 | 2.884859 | 12.91068 | 4.475325 | 2.161993 | 0.023636 | 1 | Up |
| NAPRT | 13.1981 | 82.53641 | 6.253657 | 2.6447 | 0.003412 | 1 | Up |
| NEGR1 | 26.78224 | 11.74384 | 0.438493 | -1.18937 | 0.025632 | 1 | Down |
| NELFE | 25.28774 | 4.342328 | 0.171717 | -2.5419 | 0.018696 | 1 | Down |
| NR1I2 | 12.52715 | 3.165443 | 0.252687 | -1.98458 | 0.020352 | 1 | Down |
| OR1J2 | 1.738076 | 9.852553 | 5.668655 | 2.503006 | 0.019161 | 1 | Up |
| PCDHGA11 | 15.49193 | 43.3182 | 2.796179 | 1.483456 | 0.027749 | 1 | Up |
| PCDHGA8 | 7.085747 | 20.19249 | 2.849734 | 1.510827 | 0.017683 | 1 | Up |
| PCDHGC5 | 0.630123 | 6.49666 | 10.31015 | 3.365993 | 0.020261 | 1 | Up |
| PLA2G2A | 0 | 3.439824 | +∞ | +∞ | 0.03864 | 1 | Up |
| PLCXD1 | 17.70733 | 7.034523 | 0.397266 | -1.33182 | 0.04711 | 1 | Down |
| PLEKHN1 | 8.485507 | 25.96201 | 3.059571 | 1.613329 | 0.004987 | 1 | Up |
| PRG4 | 3.983745 | 12.58892 | 3.16007 | 1.659957 | 0.038844 | 1 | Up |
| PTPRB | 37.41425 | 14.45471 | 0.386342 | -1.37205 | 0.02657 | 1 | Down |
| RAI14 | 15.35514 | 4.925576 | 0.320777 | -1.64036 | 0.02467 | 1 | Down |
| REG4 | 9.604262 | 2.498886 | 0.260185 | -1.94239 | 0.045126 | 1 | Down |
| RXRB | 22.48139 | 75.65558 | 3.365253 | 1.750715 | 0.001472 | 1 | Up |
| SAMD14 | 10.07802 | 24.73703 | 2.454553 | 1.29546 | 0.029279 | 1 | Up |
| SEMA5A | 14.77895 | 30.87039 | 2.088808 | 1.06268 | 0.027187 | 1 | Up |
| SLC35G2 | 10.64894 | 25.56205 | 2.400431 | 1.263294 | 0.018871 | 1 | Up |
| SLC47A1 | 57.9764 | 20.71751 | 0.357344 | -1.48462 | 0.008923 | 1 | Down |
| SLC4A10 | 1082.565 | 525.0878 | 0.485041 | -1.04382 | 0.00798 | 1 | Down |
| SLMO1 | 1.743271 | 8.602461 | 4.934667 | 2.302953 | 0.028835 | 1 | Up |
| SOGA3 | 18.47207 | 42.50552 | 2.301069 | 1.202304 | 0.041471 | 1 | Up |
| SORCS3 | 20.06657 | 58.64423 | 2.922484 | 1.547195 | 0.015103 | 1 | Up |
| SPP1 | 7.298104 | 42.25657 | 5.790075 | 2.533582 | 0.000259 | 1 | Up |
| SYNGAP1 | 26.45961 | 60.98621 | 2.30488 | 1.204691 | 0.011067 | 1 | Up |
| TBC1D3 | 9.43014 | 1.592365 | 0.168859 | -2.56611 | 0.017771 | 1 | Down |
| TMEM132B | 5.34504 | 0.506161 | 0.094697 | -3.40053 | 0.044718 | 1 | Down |
| TNFRSF17 | 62.04006 | 27.43129 | 0.442155 | -1.17738 | 0.042764 | 1 | Down |
| TRIM73 | 7.408658 | 19.74934 | 2.665711 | 1.414521 | 0.026888 | 1 | Up |
| VTN | 5.354024 | 0.684116 | 0.127776 | -2.96831 | 0.043358 | 1 | Down |
| ZFP57 | 0 | 9.204233 | +∞ | +∞ | 0.000631 | 1 | Up |
| ZNF415 | 101.2107 | 48.67072 | 0.480885 | -1.05624 | 0.000713 | 1 | Down |
| ZNF683 | 76.70423 | 245.1225 | 3.195685 | 1.676125 | 0.030172 | 1 | Up |
| ZP3 | 36.5716 | 13.14884 | 0.359537 | -1.47579 | 0.002049 | 1 | Down |

**Note:** PQDS: Pi-qi-deficiency syndrome; CSG: chronic superficial gastritis; BC: balanced constituion.

^a^ normalized mean count value;

^b^ normalized mean count value (PQDS of CSG) / normalized mean count value (BC);

^c^ *P*-value adjusted by false discovery rate (FDR).

**Table S5** Differential lncRNAs identified in the QDC population compared with the BC control population

| **lncRNAs** | **Base Mean ^a^**  **(BC, n=5)** | **Base Mean ^a^**  **(QDC, n=2)** | **Fold Change ^b^** | **log_2_ (Fold Change)** | ***P*-value ^c^** | **padj** | **up_down** |
| --- | --- | --- | --- | --- | --- | --- | --- |
| A2M-AS1:3 | 273.824821 | 110.7791321 | 0.404562054 | -1.305567086 | 0.00618024 | 1 | Down |
| APTR:11 | 9.233222835 | 29.54193845 | 3.199526209 | 1.677858284 | 0.04660472 | 1 | Up |
| BISPR:14 | 2.447622154 | 15.66564823 | 6.400353995 | 2.678151701 | 0.03548586 | 1 | Up |
| BZRAP1-AS1:19 | 15.29076374 | 0.492975847 | 0.032240106 | -4.954999692 | 0.00480361 | 1 | Down |
| BZRAP1-AS1:20 | 15.47773107 | 0.492975847 | 0.031850653 | -4.972533222 | 0.0044611 | 1 | Down |
| BZRAP1-AS1:21 | 76.69990281 | 31.51985781 | 0.410950427 | -1.282963723 | 0.04455778 | 1 | Down |
| C21orf62-AS1:13 | 33.35535727 | 9.323912739 | 0.27953269 | -1.838911087 | 0.0321621 | 1 | Down |
| CAPN10-AS1:2 | 33.03211598 | 2.623102454 | 0.07941067 | -3.654523326 | 0.02553475 | 1 | Down |
| CARD8-AS1:4 | 0.419869519 | 17.61322945 | 41.94929299 | 5.390574591 | 0.03037885 | 1 | Up |
| CD27-AS1:5 | 30.6619648 | 8.964837944 | 0.2923765 | -1.774100738 | 0.04235945 | 1 | Down |
| CDC42-IT1:1 | 21.07926463 | 0 | 0 | -∞ | 0.00045747 | 0.31 | Down |
| CHKB-AS1:14 | 4.566677912 | 21.81254811 | 4.776458627 | 2.255941368 | 0.02262327 | 1 | Up |
| DANT1:2 | 11.04302311 | 49.00520219 | 4.437661834 | 2.149799733 | 0.0143369 | 1 | Up |
| DICER1-AS1:2 | 12.47588954 | 0 | 0 | -∞ | 0.00430282 | 1 | Down |
| DLEU1:39 | 11.58003339 | 36.51055084 | 3.152888219 | 1.656674022 | 0.03224095 | 1 | Up |
| DLEU2:11 | 36.34236361 | 0.426025321 | 0.011722554 | -6.41456926 | 0.01353154 | 1 | Down |
| DLX6-AS1:13 | 0 | 13.20678496 | +∞ | +∞ | 0.02322834 | 1 | Up |
| EDNRB-AS1:2 | 0 | 5.873081808 | +∞ | +∞ | 0.03659742 | 1 | Up |
| FAM201A:1 | 0 | 6.140883912 | +∞ | +∞ | 0.0334457 | 1 | Up |
| FAM74A6:1 | 2.204688276 | 14.32062174 | 6.495531316 | 2.699447539 | 0.04509943 | 1 | Up |
| FTX:1 | 0 | 8.094481107 | +∞ | +∞ | 0.03599054 | 1 | Up |
| FTX:25 | 0 | 9.366541099 | +∞ | +∞ | 0.03652083 | 1 | Up |
| GAS5:31 | 46.09241637 | 9.214333853 | 0.199909976 | -2.322577626 | 0.0156167 | 1 | Down |
| GAS5:41 | 128.4232794 | 38.73195014 | 0.301596022 | -1.729310697 | 0.0019715 | 0.82 | Down |
| GAS6-AS2:1 | 1.185014956 | 18.02696455 | 15.21243632 | 3.927179318 | 0.04891727 | 1 | Up |
| GMDS-AS1:11 | 0 | 11.33844449 | +∞ | +∞ | 0.03073973 | 1 | Up |
| HOXA-AS2:2 | 0 | 6.834711337 | +∞ | +∞ | 0.0261022 | 1 | Up |
| IFNG-AS1:2 | 112.2891202 | 34.56898559 | 0.307856946 | -1.699667976 | 0.01095662 | 1 | Down |
| INHBA-AS1:2 | 0.209934759 | 7.778034671 | 37.04977059 | 5.211392704 | 0.02958044 | 1 | Up |
| LBX2-AS1:2 | 1.839216237 | 17.1932201 | 9.348123272 | 3.224676759 | 0.01108801 | 1 | Up |
| LINC00152:10 | 19.61972406 | 2.464879237 | 0.125632717 | -2.992715881 | 0.03490638 | 1 | Down |
| LINC00189:4 | 94.86222475 | 24.65480834 | 0.259901224 | -1.943964666 | 0.04134989 | 1 | Down |
| LINC00278:1 | 94.15221019 | 0 | 0 | -∞ | 0.00340215 | 1 | Down |
| LINC00278:4 | 11.74895189 | 0 | 0 | -∞ | 0.04313309 | 1 | Down |
| LINC00299:3 | 0 | 6.816405143 | +∞ | +∞ | 0.04179907 | 1 | Up |
| LINC00339:21 | 14.28155837 | 0.426025321 | 0.029830451 | -5.06707042 | 0.00709898 | 1 | Down |
| LINC00476:3 | 2.12133959 | 19.40860343 | 9.14922039 | 3.193648816 | 0.01040045 | 1 | Up |
| LINC00623:19 | 22.94378097 | 1.704101286 | 0.074272906 | -3.751020165 | 0.01821841 | 1 | Down |
| LINC00649:8 | 1.03483645 | 13.06686794 | 12.62698848 | 3.658438694 | 0.01739637 | 1 | Up |
| LINC00654:15 | 9.421196916 | 0 | 0 | -∞ | 0.03392102 | 1 | Down |
| LINC00654:4 | 34.81250204 | 74.88944216 | 2.151222629 | 1.105156836 | 0.04989945 | 1 | Up |
| LINC00663:1 | 55.25811977 | 20.3092984 | 0.367535097 | -1.444046072 | 0.04645225 | 1 | Down |
| LINC00843:2 | 8.265704653 | 0 | 0 | -∞ | 0.04816032 | 1 | Down |
| LINC00847:14 | 0 | 18.74511414 | +∞ | +∞ | 0.0168212 | 1 | Up |
| LINC00861:6 | 9.196164714 | 44.65367628 | 4.855684698 | 2.279674744 | 0.00224176 | 0.88 | Up |
| LINC00869:34 | 136.8037138 | 8.094481107 | 0.059168577 | -4.079024985 | 0.00421245 | 1 | Down |
| LINC00887:5 | 0 | 15.76293689 | +∞ | +∞ | 0.01975143 | 1 | Up |
| LINC00888:6 | 22.22480976 | 2.69005298 | 0.12103829 | -3.046464578 | 0.01053759 | 1 | Down |
| LINC00893:4 | 1.821526497 | 17.35144332 | 9.525770474 | 3.251835787 | 0.01005745 | 1 | Up |
| LINC00894:20 | 8.859485586 | 87.04358318 | 9.824902624 | 3.296443109 | 0.00986773 | 1 | Up |
| LINC00894:32 | 0 | 17.41237787 | +∞ | +∞ | 0.00284922 | 1 | Up |
| LINC00920:3 | 0 | 6.098255552 | +∞ | +∞ | 0.03096163 | 1 | Up |
| LINC00936:1 | 239.0323335 | 18.74511414 | 0.078420831 | -3.672619267 | 0.02958695 | 1 | Down |
| LINC00987:3 | 116.8309078 | 50.00946007 | 0.428049914 | -1.224149057 | 0.02680623 | 1 | Down |
| LINC00996:2 | 27.77391315 | 5.288833269 | 0.190424491 | -2.392709057 | 0.01529602 | 1 | Down |
| LINC01089:2 | 19.67417803 | 2.69005298 | 0.136730133 | -2.870596871 | 0.01972388 | 1 | Down |
| LINC01184:20 | 24.05946846 | 1.278075964 | 0.053121538 | -4.234559277 | 0.00125163 | 0.6 | Down |
| LINC01184:24 | 24.8335302 | 2.556151929 | 0.102931476 | -3.280243869 | 0.02124945 | 1 | Down |
| LINC01237:6 | 19.617295 | 49.74165797 | 2.535602282 | 1.342328471 | 0.03899498 | 1 | Up |
| LINC01299:2 | 0.570113187 | 13.11551227 | 23.00510243 | 4.523881975 | 0.00724504 | 1 | Up |
| LINC01341:3 | 46.03935397 | 16.09167355 | 0.349519969 | -1.516553214 | 0.03637671 | 1 | Down |
| LINC01347:8 | 15.80959869 | 54.06284574 | 3.419621636 | 1.773836707 | 0.01024951 | 1 | Up |
| LINC01410:6 | 143.494481 | 40.27782821 | 0.280692525 | -1.83293745 | 0.04644441 | 1 | Down |
| LINC01426:3 | 2.412240228 | 14.01019127 | 5.807958558 | 2.538031159 | 0.04964882 | 1 | Up |
| LINC01506:2 | 1.950974133 | 25.06252747 | 12.84616082 | 3.683265358 | 0.00110999 | 0.56 | Up |
| LINC01550:2 | 2.042160437 | 14.16841449 | 6.937953669 | 2.794510206 | 0.03250008 | 1 | Up |
| LINC01572:7 | 0 | 6.901661863 | +∞ | +∞ | 0.04787287 | 1 | Up |
| LOH12CR2:1 | 7.763771726 | 27.41181184 | 3.530733877 | 1.819968084 | 0.04653455 | 1 | Up |
| MIF-AS1:4 | 80.31313475 | 0.426025321 | 0.005304554 | -7.55855296 | 1.32E-05 | 0.02 | Down |
| MIR22HG:3 | 16.63284834 | 1.278075964 | 0.076840475 | -3.701989755 | 0.01289949 | 1 | Down |
| MIR4435-2HG:10 | 34.01366349 | 5.538329178 | 0.1628266 | -2.618591692 | 0.00639612 | 1 | Down |
| MIR4435-2HG:5 | 23.13038921 | 0.985951695 | 0.042625815 | -4.552128766 | 0.00518503 | 1 | Down |
| MIR4435-2HG:9 | 9.761895036 | 36.58351734 | 3.747583559 | 1.905960646 | 0.01679045 | 1 | Up |
| NDUFA6-AS1:5 | 13.01120144 | 37.92252786 | 2.914606158 | 1.54330095 | 0.04000469 | 1 | Up |
| NEAT1:14 | 2361.99114 | 5207.062688 | 2.204522532 | 1.140466222 | 0.01107224 | 1 | Up |
| PAN3-AS1:5 | 27.10424803 | 1.971903389 | 0.072752558 | -3.780858206 | 0.00818791 | 1 | Down |
| PART1:10 | 0 | 8.094481107 | +∞ | +∞ | 0.03599054 | 1 | Up |
| PCBP1-AS1:169 | 17.31016668 | 52.54128984 | 3.035285033 | 1.601832001 | 0.01652843 | 1 | Up |
| PCBP1-AS1:181 | 5.667555009 | 29.0489626 | 5.125484015 | 2.357688249 | 0.01174621 | 1 | Up |
| PCBP1-AS1:198 | 59.68622903 | 152.4569053 | 2.554306208 | 1.352931485 | 0.00594045 | 1 | Up |
| PCBP1-AS1:72 | 0 | 7.887613557 | +∞ | +∞ | 0.04256077 | 1 | Up |
| PCED1B-AS1:12 | 161.1775452 | 47.0149926 | 0.291696914 | -1.77745797 | 0.02874074 | 1 | Down |
| PCF11-AS1:2 | 0 | 5.246204909 | +∞ | +∞ | 0.04964881 | 1 | Up |
| PITPNA-AS1:2 | 32.20033899 | 6.390379821 | 0.198456911 | -2.333102289 | 0.01471599 | 1 | Down |
| PRKCQ-AS1:16 | 15.94162947 | 53.72809311 | 3.370301211 | 1.752877534 | 0.00876373 | 1 | Up |
| PRKCQ-AS1:28 | 2.150396001 | 16.35947565 | 7.607657215 | 2.927452243 | 0.04558171 | 1 | Up |
| PRKCQ-AS1:3 | 43.81090132 | 0 | 0 | -∞ | 0.00424772 | 1 | Down |
| PSMA3-AS1:6 | 38.57987512 | 10.20028555 | 0.264393949 | -1.919238934 | 0.02337322 | 1 | Down |
| PSMB8-AS1:1 | 12.60422085 | 61.04950604 | 4.843576351 | 2.276072683 | 0.03835574 | 1 | Up |
| PSMB8-AS1:3 | 74.28971076 | 204.383092 | 2.751162844 | 1.460041537 | 0.01564158 | 1 | Up |
| PSMB8-AS1:5 | 0.195097413 | 10.71758356 | 54.93452415 | 5.779641205 | 0.03050905 | 1 | Up |
| PSMD5-AS1:10 | 27.65667396 | 0 | 0 | -∞ | 0.02377354 | 1 | Down |
| PSMD5-AS1:22 | 258.9163188 | 109.8052123 | 0.424095371 | -1.237539359 | 0.01143903 | 1 | Down |
| PSMD5-AS1:3 | 31.83303919 | 0.852050643 | 0.026766236 | -5.22344191 | 0.00016221 | 0.16 | Down |
| PTPRG-AS1:7 | 10.17192975 | 0 | 0 | -∞ | 0.0122127 | 1 | Down |
| RGPD4-AS1:6 | 1.351583954 | 14.81359759 | 10.96017568 | 3.454199018 | 0.01804467 | 1 | Up |
| SATB1-AS1:8 | 25.11641324 | 0.426025321 | 0.016962029 | -5.881547462 | 0.01935427 | 1 | Down |
| SCHLAP1:3 | 0 | 7.619811454 | +∞ | +∞ | 0.01535926 | 1 | Up |
| SLC25A25-AS1:15 | 4.160256685 | 21.61169653 | 5.194798824 | 2.37706788 | 0.02118694 | 1 | Up |
| SMIM2-AS1:11 | 3.606515622 | 22.21425127 | 6.159477345 | 2.622807938 | 0.01336242 | 1 | Up |
| SNAI3-AS1:16 | 2.615737545 | 15.26394507 | 5.835426838 | 2.544838187 | 0.04321298 | 1 | Up |
| SNHG4:3 | 22.35964557 | 68.79720258 | 3.07684674 | 1.621452584 | 0.006851 | 1 | Up |
| ST3GAL5-AS1:4 | 0.382064743 | 12.84771017 | 33.62704984 | 5.071550309 | 0.03900563 | 1 | Up |
| STXBP5-AS1:13 | 2.924631509 | 21.17965524 | 7.241820098 | 2.856352338 | 0.01289084 | 1 | Up |
| TCL6:1 | 7.56010598 | 0 | 0 | -∞ | 0.04201117 | 1 | Down |
| TCL6:19 | 2.537347486 | 20.57710051 | 8.109689595 | 3.019646695 | 0.00965232 | 1 | Up |
| TCONS_00000037 | 236.5470618 | 504.2151952 | 2.131563974 | 1.091912355 | 0.00603296 | 1 | Up |
| TCONS_00000205 | 91.87750542 | 8.788308532 | 0.09565245 | -3.386054262 | 8.78E-05 | 0.11 | Down |
| TCONS_00001685 | 7.999982882 | 33.57701792 | 4.197136221 | 2.069405288 | 0.01363975 | 1 | Up |
| TCONS_00002746 | 105.3956527 | 35.32374757 | 0.335153744 | -1.577105046 | 0.04329867 | 1 | Down |
| TCONS_00002748 | 48.07636987 | 5.605279704 | 0.116591159 | -3.100469695 | 0.00037023 | 0.27 | Down |
| TCONS_00003855 | 22.08470353 | 95.21103078 | 4.311175409 | 2.108081263 | 0.00993015 | 1 | Up |
| TCONS_00003873 | 2.252819158 | 52.37679237 | 23.24944378 | 4.539124297 | 0.03280904 | 1 | Up |
| TCONS_00003875 | 59.61438455 | 193.1424527 | 3.239863233 | 1.695932913 | 0.00031163 | 0.25 | Up |
| TCONS_00011350 | 30.23439387 | 7.668455786 | 0.253633521 | -1.979182663 | 0.04204728 | 1 | Down |
| TCONS_00011352 | 2.746499379 | 46.16921621 | 16.81020449 | 4.07126537 | 0.00010535 | 0.12 | Up |
| TCONS_00012972 | 375.9707233 | 0 | 0 | -∞ | 0.00032819 | 0.25 | Down |
| TCONS_00013625 | 48.61942573 | 14.01019127 | 0.288160361 | -1.795056201 | 0.01745267 | 1 | Down |
| TCONS_00018893 | 55.80016688 | 13.17644683 | 0.236136334 | -2.082308053 | 0.00289212 | 1 | Down |
| TCONS_00020149 | 1224.2654 | 458.4238765 | 0.374448119 | -1.417162252 | 0.0069544 | 1 | Down |
| TCONS_00025364 | 34.07686343 | 76.57523725 | 2.247132792 | 1.168085382 | 0.03207487 | 1 | Up |
| TCONS_00026996 | 114.0257579 | 43.73467511 | 0.383550839 | -1.382510278 | 0.02187317 | 1 | Down |
| TCONS_00031339 | 18.23510869 | 59.18116557 | 3.245451759 | 1.698419312 | 0.00787222 | 1 | Up |
| TCONS_00031535 | 1031.701159 | 381.9754913 | 0.370238502 | -1.433473163 | 0.00100039 | 0.52 | Down |
| TCONS_00031767 | 127.8486673 | 0.426025321 | 0.003332263 | -8.229282225 | 2.43E-06 | 0.01 | Down |
| TCONS_00032780 | 33.44081037 | 0 | 0 | -∞ | 0.01603916 | 1 | Down |
| TCONS_00038322 | 494.9600555 | 241.2827974 | 0.487479332 | -1.03658704 | 0.02641929 | 1 | Down |
| TCONS_00044060 | 45.84244075 | 139.9196256 | 3.052185341 | 1.609842571 | 0.00827307 | 1 | Up |
| TCONS_00046326 | 60.36513997 | 137.0954133 | 2.271102385 | 1.183392746 | 0.01429021 | 1 | Up |
| TCONS_00048609 | 40.18394969 | 189.9105212 | 4.726029241 | 2.240628556 | 3.58E-06 | 0.01 | Up |
| TCONS_00048621 | 44.99383985 | 8.897887418 | 0.197757903 | -2.338192744 | 0.04945351 | 1 | Down |
| TCONS_00048629 | 900.8934468 | 308.2006607 | 0.342105564 | -1.547486527 | 0.01498944 | 1 | Down |
| TCONS_00048671 | 0 | 36.6078395 | +∞ | +∞ | 5.17E-09 | #### | Up |
| TCONS_00048674 | 52.35001465 | 237.3086525 | 4.533115303 | 2.180502858 | 0.03339551 | 1 | Up |
| TCONS_00048675 | 66.18224949 | 197.3964316 | 2.982618953 | 1.576579677 | 0.02234614 | 1 | Up |
| TCONS_00049082 | 52.63901677 | 1.904952863 | 0.03618899 | -4.788305337 | 0.00080956 | 0.44 | Down |
| TCONS_00049089 | 2524.974008 | 948.5878018 | 0.375682205 | -1.412415315 | 0.00045704 | 0.31 | Down |
| TCONS_00049090 | 52.42819725 | 11.83142034 | 0.225669028 | -2.147719663 | 0.00290221 | 1 | Down |
| TCONS_00049091 | 198.831928 | 73.06373004 | 0.367464777 | -1.444322128 | 0.00332662 | 1 | Down |
| TCONS_00049092 | 29.02956896 | 2.957855084 | 0.101891113 | -3.294899874 | 0.02807401 | 1 | Down |
| TCONS_00049093 | 23.16848978 | 2.130126607 | 0.091940676 | -3.44315292 | 0.01020077 | 1 | Down |
| TCONS_00049095 | 11.21751756 | 0.426025321 | 0.037978574 | -4.718670451 | 0.02402614 | 1 | Down |
| TCONS_00049096 | 115.9888842 | 32.51809973 | 0.280355311 | -1.834671693 | 0.00099778 | 0.52 | Down |
| TCONS_00049110 | 382.7804072 | 173.1679069 | 0.452394908 | -1.144345406 | 0.01132866 | 1 | Down |
| TCONS_00049113 | 2417.312408 | 1181.910794 | 0.48893589 | -1.032282785 | 0.00775874 | 1 | Down |
| TCONS_00049141 | 15.94105272 | 111.5639739 | 6.998532396 | 2.807052418 | 0.04700309 | 1 | Up |
| TCONS_00052318 | 0 | 6.816405143 | +∞ | +∞ | 0.04179907 | 1 | Up |
| TCONS_00052323 | 79.35617114 | 681.1681691 | 8.583682394 | 3.101596696 | 1.25E-07 | 0 | Up |
| TCONS_00052658 | 0 | 52.82713986 | +∞ | +∞ | 0.00625035 | 1 | Up |
| TCONS_00052860 | 0.833787104 | 9.281284379 | 11.13147989 | 3.476573502 | 0.04639786 | 1 | Up |
| TCONS_00052862 | 0 | 56.32085743 | +∞ | +∞ | 1.03E-07 | 0 | Up |
| TCONS_00052867 | 412.3346353 | 1049.437625 | 2.545111508 | 1.347728866 | 0.00075462 | 0.42 | Up |
| TCONS_00052868 | 0 | 17.8930635 | +∞ | +∞ | 0.01756522 | 1 | Up |
| TCONS_00052884 | 2036.502132 | 773.1851722 | 0.379663326 | -1.397207449 | 0.01978771 | 1 | Down |
| TCONS_00052885 | 2215.058807 | 677.9989802 | 0.306086221 | -1.707989992 | 0.00126336 | 0.6 | Down |
| TCONS_00052886 | 3096.886219 | 1315.354525 | 0.424734534 | -1.23536668 | 0.01446997 | 1 | Down |
| TCONS_00052887 | 2264.57466 | 592.3305034 | 0.26156369 | -1.934765816 | 0.00577189 | 1 | Down |
| TCONS_00052889 | 3006.452988 | 1160.620527 | 0.386043132 | -1.373166049 | 0.00134438 | 0.62 | Down |
| TCONS_00052890 | 2730.185578 | 1031.059926 | 0.377651957 | -1.404870832 | 0.01497093 | 1 | Down |
| THAP9-AS1:5 | 262.0759446 | 128.1308337 | 0.488907266 | -1.032367247 | 0.02235647 | 1 | Down |
| THUMPD3-AS1:27 | 63.88410744 | 144.9037861 | 2.268229016 | 1.181566312 | 0.01317316 | 1 | Up |
| THUMPD3-AS1:5 | 39.37045571 | 102.2099813 | 2.596108667 | 1.376350773 | 0.01113922 | 1 | Up |
| TP53TG1:8 | 17.29543986 | 0 | 0 | -∞ | 0.01987639 | 1 | Down |
| TPT1-AS1:16 | 36.85917183 | 10.89411297 | 0.295560438 | -1.758474923 | 0.03699117 | 1 | Down |
| TRAF3IP2-AS1:24 | 0.196178527 | 7.844985197 | 39.98901059 | 5.321531681 | 0.02932536 | 1 | Up |
| TTC21B-AS1:3 | 9.970786369 | 35.16552435 | 3.526855661 | 1.818382534 | 0.02542988 | 1 | Up |
| TTN-AS1:1 | 27.37507954 | 6.925984029 | 0.253003248 | -1.982772191 | 0.04182942 | 1 | Down |
| TTTY10:1 | 23.97746718 | 0 | 0 | -∞ | 0.01163033 | 1 | Down |
| TTTY14:13 | 10.03253826 | 0 | 0 | -∞ | 0.02653011 | 1 | Down |
| TTTY15:1 | 79.7809101 | 0 | 0 | -∞ | 0.00816891 | 1 | Down |
| TTTY15:2 | 51.64612167 | 0 | 0 | -∞ | 0.00280484 | 1 | Down |
| TTTY15:5 | 44.13932105 | 0 | 0 | -∞ | 0.00982704 | 1 | Down |
| TTTY15:6 | 42.52697398 | 0 | 0 | -∞ | 0.00999824 | 1 | Down |
| UBA6-AS1:1 | 1.235153143 | 15.58039151 | 12.61413744 | 3.656969653 | 0.00838483 | 1 | Up |
| UGDH-AS1:6 | 59.43359247 | 134.1132361 | 2.256522457 | 1.174101136 | 0.01602568 | 1 | Up |
| XIST:20 | 22.14765721 | 117.2181562 | 5.292575875 | 2.403970047 | 0.00668272 | 1 | Up |
| XIST:25 | 0 | 5.9643545 | +∞ | +∞ | 0.04687905 | 1 | Up |
| XIST:6 | 1299.581899 | 4413.442991 | 3.39604837 | 1.763857008 | 0.02808691 | 1 | Up |
| ZEB1-AS1:11 | 55.7758302 | 20.55277834 | 0.368488972 | -1.440306651 | 0.03580761 | 1 | Down |
| ZNF528-AS1:3 | 0 | 12.23913946 | +∞ | +∞ | 0.00118697 | 0.58 | Up |
| ZNF674-AS1:3 | 20.60627671 | 1.971903389 | 0.095694308 | -3.385423076 | 0.02100289 | 1 | Down |
| lnc-AAGAB-3:2 | 12.02740188 | 0 | 0 | -∞ | 0.00561878 | 1 | Down |
| lnc-ABCC6-2:1 | 0.195097413 | 12.06261005 | 61.8286519 | 5.950203645 | 0.01728965 | 1 | Up |
| lnc-ABHD2-4:1 | 12.88934237 | 40.55164628 | 3.146137725 | 1.653581827 | 0.0241924 | 1 | Up |
| lnc-ABT1-5:1 | 38.63144231 | 88.40064161 | 2.288308081 | 1.194281299 | 0.03364779 | 1 | Up |
| lnc-AC006455.1-10:1 | 200.8968736 | 408.2614344 | 2.032194066 | 1.02303818 | 0.00894252 | 1 | Up |
| lnc-AC007952.2.1-2:1 | 52360.33645 | 24228.46908 | 0.462725619 | -1.111771119 | 0.00477649 | 1 | Down |
| lnc-AC008686.1-6:1 | 73.46712377 | 166.4793868 | 2.266039261 | 1.180172857 | 0.0360691 | 1 | Up |
| lnc-AC022098.1-1:5 | 2.309282352 | 14.05281963 | 6.085362243 | 2.605343145 | 0.0451278 | 1 | Up |
| lnc-AC037487.1-3:1 | 7.977599969 | 0 | 0 | -∞ | 0.03625538 | 1 | Down |
| lnc-AC069257.9.1-5:5 | 0.18696733 | 24.70946864 | 132.1592849 | 7.046133975 | 0.02368236 | 1 | Up |
| lnc-AC091132.2-7:1 | 0 | 17.46703818 | +∞ | +∞ | 0.01796262 | 1 | Up |
| lnc-AC092329.1-1:24 | 28.57909902 | 1.478927542 | 0.051748571 | -4.272337159 | 0.02138097 | 1 | Down |
| lnc-AC103810.1-1:2 | 0 | 9.798582393 | +∞ | +∞ | 0.0303972 | 1 | Up |
| lnc-AC104024.2.1-4:1 | 15.43209303 | 1.278075964 | 0.082819353 | -3.593888253 | 0.02044994 | 1 | Down |
| lnc-AC107021.1-7:1 | 0 | 17.28449279 | +∞ | +∞ | 7.72E-05 | 0.1 | Up |
| lnc-AC110373.1-8:26 | 12.27284349 | 0 | 0 | -∞ | 0.01360434 | 1 | Down |
| lnc-AC131097.4.1-8:1 | 0.18696733 | 11.928709 | 63.80103408 | 5.995507902 | 0.04396518 | 1 | Up |
| lnc-AC233264.2-1:1 | 167.3126054 | 30.643485 | 0.183151084 | -2.448893857 | 0.00011292 | 0.13 | Down |
| lnc-ACP1-15:1 | 1.368925234 | 15.08741566 | 11.02135842 | 3.462230147 | 0.01334067 | 1 | Up |
| lnc-ACR-2:5 | 0 | 29.39574718 | +∞ | +∞ | 0.01100488 | 1 | Up |
| lnc-ACSBG2-2:1 | 1.006242872 | 10.30986443 | 10.24590059 | 3.356974894 | 0.04693045 | 1 | Up |
| lnc-ACTG1-2:3 | 56.05311286 | 0 | 0 | -∞ | 0.00179558 | 0.76 | Down |
| lnc-ACTR1B-3:10 | 47.84497018 | 14.95351461 | 0.312540995 | -1.677882657 | 0.02784233 | 1 | Down |
| lnc-ADA-2:1 | 0.382064743 | 12.91466069 | 33.8022833 | 5.079048797 | 0.02532442 | 1 | Up |
| lnc-ADAM32-2:5 | 18.29306713 | 0.852050643 | 0.04657779 | -4.424213995 | 0.00492425 | 1 | Down |
| lnc-AIF1-2:1 | 47.77744437 | 125.3858621 | 2.624373567 | 1.391973095 | 0.00663924 | 1 | Up |
| lnc-AKAP11-1:2 | 2.354142325 | 32.53013167 | 13.81825191 | 3.788503212 | 0.00341675 | 1 | Up |
| lnc-AKAP12-1:2 | 4.33709338 | 57.3491792 | 13.22295237 | 3.724972427 | 0.0065786 | 1 | Up |
| lnc-AKAP7-1:1 | 0 | 10.22460771 | +∞ | +∞ | 0.02926433 | 1 | Up |
| lnc-AL359853.2-2:1 | 162.1521184 | 43.89289833 | 0.270689639 | -1.885288429 | 0.00055383 | 0.35 | Down |
| lnc-AL669831.1-3:42 | 1.770714065 | 15.89082197 | 8.97424507 | 3.165790582 | 0.01882452 | 1 | Up |
| lnc-AL901608.1-3:27 | 27.15201842 | 0 | 0 | -∞ | 0.00584891 | 1 | Down |
| lnc-ALKBH4-8:1 | 59.68078716 | 1.478927542 | 0.024780631 | -5.334643288 | 0.00038166 | 0.28 | Down |
| lnc-AMPH-10:7 | 260.7501418 | 104.5286693 | 0.40087675 | -1.31876935 | 0.00692182 | 1 | Down |
| lnc-AMPH-2:2 | 29.27949665 | 0.852050643 | 0.029100591 | -5.10280776 | 0.00426875 | 1 | Down |
| lnc-AMPH-5:1 | 53.4345826 | 19.54852045 | 0.365840239 | -1.450714329 | 0.04285284 | 1 | Down |
| lnc-AMZ2-3:1 | 8.401079132 | 28.76285431 | 3.423709485 | 1.775560289 | 0.03388812 | 1 | Up |
| lnc-AMZ2-5:1 | 42.79587825 | 114.4308146 | 2.673874664 | 1.418931842 | 0.00593561 | 1 | Up |
| lnc-ANKRD18B-6:4 | 11.06639258 | 0.426025321 | 0.038497217 | -4.699102017 | 0.02567198 | 1 | Down |
| lnc-ANKRD20A4-1:1 | 16.23876394 | 0.492975847 | 0.030357966 | -5.041781046 | 0.0031901 | 1 | Down |
| lnc-ANKRD30B-7:2 | 0 | 8.520506428 | +∞ | +∞ | 0.03440391 | 1 | Up |
| lnc-ANKS1B-3:1 | 18.15228757 | 47.83068914 | 2.634967574 | 1.397785208 | 0.03673254 | 1 | Up |
| lnc-ANXA3-8:1 | 0.195097413 | 13.80332373 | 70.75093152 | 6.144677238 | 0.04487761 | 1 | Up |
| lnc-AP000525.1-4:1 | 17.27253903 | 0 | 0 | -∞ | 0.00057884 | 0.36 | Down |
| lnc-AP000769.1-1:2 | 4.835391047 | 23.28545968 | 4.815631137 | 2.26772489 | 0.02631609 | 1 | Up |
| lnc-AP001793.1-4:1 | 19.59516729 | 0 | 0 | -∞ | 0.00022902 | 0.21 | Down |
| lnc-AP006621.5.1-2:2 | 3.716580877 | 22.99333541 | 6.186690449 | 2.629167851 | 0.04341452 | 1 | Up |
| lnc-APBA2-5:8 | 0 | 7.668455786 | +∞ | +∞ | 0.03773428 | 1 | Up |
| lnc-APH1A-1:6 | 17.31090509 | 46.39438995 | 2.68006726 | 1.422269207 | 0.04301574 | 1 | Up |
| lnc-APOBEC3A-2:1 | 490.691317 | 233.3959587 | 0.475647216 | -1.072036162 | 0.0140822 | 1 | Down |
| lnc-ARAF-1:1 | 0.419869519 | 16.61498754 | 39.5717879 | 5.306400344 | 0.04454056 | 1 | Up |
| lnc-ARHGAP27-2:1 | 10.69741899 | 0 | 0 | -∞ | 0.01031228 | 1 | Down |
| lnc-ARL1-3:1 | 18.96697994 | 48.840963 | 2.575052177 | 1.364601665 | 0.04445272 | 1 | Up |
| lnc-ARL13B-1:5 | 0.419869519 | 13.04254577 | 31.06333086 | 4.95714063 | 0.01358664 | 1 | Up |
| lnc-ASAH2-4:1 | 36.30533614 | 10.44376549 | 0.287664751 | -1.797539643 | 0.02666762 | 1 | Down |
| lnc-ATAD5-3:5 | 132.3400552 | 60.03949046 | 0.453675876 | -1.140266148 | 0.02612431 | 1 | Down |
| lnc-ATF7IP2-3:4 | 52.06516848 | 3.049127776 | 0.058563678 | -4.09385002 | 9.46E-06 | 0.02 | Down |
| lnc-ATF7IP2-7:1 | 707.016497 | 184.4940612 | 0.260947322 | -1.938169501 | 0.03021264 | 1 | Down |
| lnc-ATL3-2:1 | 93.50486965 | 0 | 0 | -∞ | 4.63E-05 | 0.07 | Down |
| lnc-B3GNT9-1:1 | 12.88227697 | 37.30166693 | 2.895580262 | 1.533852487 | 0.03669201 | 1 | Up |
| lnc-BARHL2-5:1 | 160.7329374 | 70.0632466 | 0.435898502 | -1.19793585 | 0.01686511 | 1 | Down |
| lnc-BCL11B-1:2 | 20.22393534 | 2.397928711 | 0.118568848 | -3.076203082 | 0.01115944 | 1 | Down |
| lnc-BCL2L2-PABPN1-1:1 | 85.77833821 | 4.686278536 | 0.054632424 | -4.194098756 | 1.69E-06 | 0.01 | Down |
| lnc-BCL2L2-PABPN1-1:2 | 20.00550432 | 243.6268407 | 12.17799046 | 3.606204182 | 9.08E-05 | 0.11 | Up |
| lnc-BCL9L-1:1 | 25.28163557 | 64.90204013 | 2.567161446 | 1.360174028 | 0.04260639 | 1 | Up |
| lnc-BDP1-5:1 | 0.419869519 | 10.24291391 | 24.3954692 | 4.608541326 | 0.0171434 | 1 | Up |
| lnc-BIRC2-3:1 | 0 | 11.928709 | +∞ | +∞ | 0.02547658 | 1 | Up |
| lnc-BMS1-12:1 | 850.3960271 | 362.250958 | 0.425979128 | -1.231145352 | 0.00345655 | 1 | Down |
| lnc-BRI3-2:3 | 63.53183578 | 236.8470479 | 3.728005732 | 1.898404078 | 0.01109668 | 1 | Up |
| lnc-BTK-1:2 | 22.91490219 | 2.197077133 | 0.095879839 | -3.382628703 | 0.00551731 | 1 | Down |
| lnc-C10orf90-2:2 | 10.86021613 | 36.53487301 | 3.364101836 | 1.750221378 | 0.02727894 | 1 | Up |
| lnc-C11orf54-1:1 | 2063.32144 | 1029.707334 | 0.499053281 | -1.002734243 | 0.01464111 | 1 | Down |
| lnc-C11orf54-2:1 | 37.04528484 | 9.390863265 | 0.253496857 | -1.979960236 | 0.01690331 | 1 | Down |
| lnc-C11orf94-1:1 | 0 | 5.940032334 | +∞ | +∞ | 0.03577548 | 1 | Up |
| lnc-C12orf39-2:4 | 22.93375365 | 0.492975847 | 0.021495646 | -5.539811729 | 0.00029265 | 0.24 | Down |
| lnc-C12orf50-6:2 | 1.600404603 | 12.79906584 | 7.997393794 | 2.999529928 | 0.03760075 | 1 | Up |
| lnc-C14orf178-1:4 | 2.306306385 | 20.08412466 | 8.708350628 | 3.122399497 | 0.00705893 | 1 | Up |
| lnc-C14orf183-1:13 | 19.99304287 | 53.03426568 | 2.65263602 | 1.407426731 | 0.03186105 | 1 | Up |
| lnc-C14orf79-3:1 | 0.18696733 | 6.658181925 | 35.61147245 | 5.154270184 | 0.04849582 | 1 | Up |
| lnc-C15orf39-1:1 | 196.3685156 | 510.185824 | 2.59810399 | 1.377459176 | 0.00060095 | 0.36 | Up |
| lnc-C16orf72-7:1 | 39.9506185 | 92.5209778 | 2.31588349 | 1.211562675 | 0.02893015 | 1 | Up |
| lnc-C17orf100-2:3 | 40.1954338 | 0 | 0 | -∞ | 0.00243532 | 0.92 | Down |
| lnc-C17orf109-3:1 | 52.15248294 | 129.0861889 | 2.475168615 | 1.307526808 | 0.00859434 | 1 | Up |
| lnc-C17orf62-6:6 | 0 | 10.35249279 | +∞ | +∞ | 0.03337778 | 1 | Up |
| lnc-C17orf77-1:6 | 1.469543315 | 14.41189443 | 9.807056575 | 3.2938202 | 0.01832512 | 1 | Up |
| lnc-C19orf52-1:1 | 11.17231342 | 0 | 0 | -∞ | 0.00780241 | 1 | Down |
| lnc-C1orf132-1:5 | 73.16774726 | 194.1284044 | 2.65319641 | 1.407731479 | 0.01165045 | 1 | Up |
| lnc-C1orf138-1:1 | 238.2922828 | 519.7652486 | 2.181208902 | 1.125127949 | 0.00479307 | 1 | Up |
| lnc-C1orf186-3:1 | 15.69473598 | 130.0844309 | 8.288411542 | 3.051095639 | 0.00038731 | 0.28 | Up |
| lnc-C1orf227-3:1 | 66.75021263 | 21.922127 | 0.328420332 | -1.60638465 | 0.01549601 | 1 | Down |
| lnc-C20orf24-3:1 | 0 | 12.98161122 | +∞ | +∞ | 0.00439127 | 1 | Up |
| lnc-C2orf89-2:2 | 0 | 11.07665836 | +∞ | +∞ | 0.02723757 | 1 | Up |
| lnc-C3orf62-2:1 | 2.749102767 | 38.11108921 | 13.86310096 | 3.793178097 | 9.05E-05 | 0.11 | Up |
| lnc-C3orf79-2:1 | 38.46695317 | 8.563134788 | 0.222610165 | -2.167408624 | 0.03945599 | 1 | Down |
| lnc-C4orf36-2:5 | 2.553477357 | 15.5986977 | 6.108805962 | 2.610890416 | 0.04250527 | 1 | Up |
| lnc-C4orf42-7:1 | 0 | 8.563134788 | +∞ | +∞ | 0.00846747 | 1 | Up |
| lnc-C4orf46-2:1 | 10.96147513 | 0.492975847 | 0.044973495 | -4.474781185 | 0.02605777 | 1 | Down |
| lnc-C4orf46-2:2 | 29.43359429 | 2.98217725 | 0.101318827 | -3.303025814 | 0.02729348 | 1 | Down |
| lnc-C5orf25-3:3 | 6.481148946 | 24.97125477 | 3.852905554 | 1.945946822 | 0.0427299 | 1 | Up |
| lnc-C6orf123-6:1 | 0 | 7.05988508 | +∞ | +∞ | 0.02035627 | 1 | Up |
| lnc-C6orf195-28:1 | 1.177071163 | 11.02801403 | 9.369029142 | 3.227899558 | 0.0449853 | 1 | Up |
| lnc-C7orf42-4:5 | 11.10484265 | 0.852050643 | 0.076727845 | -3.704105958 | 0.04777002 | 1 | Down |
| lnc-C8orf31-1:1 | 7.263471572 | 0 | 0 | -∞ | 0.0472667 | 1 | Down |
| lnc-CA10-1:1 | 2.170045757 | 24.61819595 | 11.34455155 | 3.503927675 | 0.03548786 | 1 | Up |
| lnc-CACYBP-2:1 | 7.842687829 | 33.93007675 | 4.326332692 | 2.113144612 | 0.04009623 | 1 | Up |
| lnc-CADM4-2:1 | 31.55172571 | 73.31322595 | 2.323588466 | 1.216354574 | 0.04118363 | 1 | Up |
| lnc-CALHM1-1:5 | 16.29556593 | 0 | 0 | -∞ | 0.0033 | 1 | Down |
| lnc-CAMK1-1:2 | 0 | 11.09496455 | +∞ | +∞ | 0.00228287 | 0.88 | Up |
| lnc-CAMK1D-1:1 | 58.72757599 | 0 | 0 | -∞ | 0.00617826 | 1 | Down |
| lnc-CARKD-4:1 | 0 | 10.65063304 | +∞ | +∞ | 0.02821406 | 1 | Up |
| lnc-CCDC103-4:1 | 13.16297123 | 0.985951695 | 0.07490343 | -3.738824404 | 0.02887469 | 1 | Down |
| lnc-CCNT1-1:1 | 111.2672951 | 2.623102454 | 0.023574784 | -5.406611634 | 0.03980262 | 1 | Down |
| lnc-CCT5-2:1 | 10.66750824 | 35.42103623 | 3.32046017 | 1.731383193 | 0.03649741 | 1 | Up |
| lnc-CD177-3:1 | 66.63770107 | 268.8107207 | 4.033913481 | 2.012180141 | 4.43E-06 | 0.01 | Up |
| lnc-CDH9-3:1 | 12.56557871 | 52.87604247 | 4.208006944 | 2.073137085 | 0.00285454 | 1 | Up |
| lnc-CDK2AP1-1:4 | 9.771062788 | 0 | 0 | -∞ | 0.04090937 | 1 | Down |
| lnc-CELF6-1:4 | 22.20767553 | 2.957855084 | 0.133190666 | -2.908435118 | 0.04130906 | 1 | Down |
| lnc-CELF6-1:5 | 109.2079629 | 47.36805143 | 0.433741736 | -1.205091824 | 0.03632142 | 1 | Down |
| lnc-CEP170-11:1 | 0 | 7.668455786 | +∞ | +∞ | 0.03773428 | 1 | Up |
| lnc-CEP170-9:2 | 5.235277778 | 22.28120179 | 4.255973177 | 2.089489058 | 0.03942178 | 1 | Up |
| lnc-CERS3-2:1 | 24.81708945 | 5.605279704 | 0.225863702 | -2.146475655 | 0.04327669 | 1 | Down |
| lnc-CGNL1-6:1 | 0 | 20.44921543 | +∞ | +∞ | 0.01550826 | 1 | Up |
| lnc-CHCHD7-5:2 | 0 | 7.39463771 | +∞ | +∞ | 0.04505675 | 1 | Up |
| lnc-CHRNA1-4:2 | 13.01743424 | 0 | 0 | -∞ | 0.03853891 | 1 | Down |
| lnc-CLCN1-2:1 | 0 | 24.70946864 | +∞ | +∞ | 0.01297828 | 1 | Up |
| lnc-CLDN23-3:1 | 3.183604973 | 25.71372653 | 8.076921209 | 3.013805466 | 0.00342129 | 1 | Up |
| lnc-CLDN9-1:1 | 102.734288 | 227.0238851 | 2.209816114 | 1.143926323 | 0.01029394 | 1 | Up |
| lnc-CLEC2D-8:4 | 172.1953097 | 58.2438582 | 0.338243 | -1.563868016 | 0.03793428 | 1 | Down |
| lnc-CLEC2D-8:6 | 25.72449609 | 0 | 0 | -∞ | 0.02679046 | 1 | Down |
| lnc-CLIC3-2:1 | 1.158648769 | 13.74238917 | 11.86070321 | 3.568117644 | 0.01643252 | 1 | Up |
| lnc-CLK3-1:1 | 7.675641997 | 0 | 0 | -∞ | 0.03910883 | 1 | Down |
| lnc-CLLU1.1-1:10 | 236.6495812 | 80.48243164 | 0.340091164 | -1.556006569 | 0.02872281 | 1 | Down |
| lnc-CLTC-2:1 | 0 | 16.76117881 | +∞ | +∞ | 0.02146239 | 1 | Up |
| lnc-COBL-1:1 | 0 | 10.64461706 | +∞ | +∞ | 0.00626006 | 1 | Up |
| lnc-COG6-7:1 | 26.7644375 | 4.595005844 | 0.171683259 | -2.542178731 | 0.01249377 | 1 | Down |
| lnc-COIL-6:1 | 104.5729496 | 212.3502045 | 2.030641819 | 1.021935788 | 0.02259362 | 1 | Up |
| lnc-COMTD1-6:3 | 9.122653673 | 0 | 0 | -∞ | 0.02114971 | 1 | Down |
| lnc-CORIN-1:1 | 527.240069 | 233.7301948 | 0.443308862 | -1.173615893 | 0.01032709 | 1 | Down |
| lnc-COX19-2:2 | 0.196178527 | 10.9610635 | 55.87290139 | 5.804076834 | 0.0066127 | 1 | Up |
| lnc-CRYBA4-1:34 | 0 | 14.88656408 | +∞ | +∞ | 0.00031971 | 0.25 | Up |
| lnc-CRYBA4-1:55 | 1.212120551 | 26.09110752 | 21.52517545 | 4.427953092 | 0.00019888 | 0.2 | Up |
| lnc-CSNK1D-1:1 | 14.56062712 | 46.71083639 | 3.208023665 | 1.681684785 | 0.01922066 | 1 | Up |
| lnc-CSNK1D-2:9 | 4.666724984 | 26.15805805 | 5.605228107 | 2.486773085 | 0.01037154 | 1 | Up |
| lnc-CTNNA3-3:1 | 42.84433578 | 105.5206369 | 2.462884183 | 1.300348787 | 0.03346942 | 1 | Up |
| lnc-CTU2-2:1 | 0 | 7.887613557 | +∞ | +∞ | 0.04256077 | 1 | Up |
| lnc-CYorf15A.1-2:6 | 498.9243749 | 1.278075964 | 0.002561663 | -8.608703756 | 0.00066958 | 0.39 | Down |
| lnc-CYorf15A.1-2:7 | 96.72744792 | 0 | 0 | -∞ | 0.00158382 | 0.7 | Down |
| lnc-CYorf15A.1-3:1 | 22.47444599 | 0 | 0 | -∞ | 0.01281848 | 1 | Down |
| lnc-DBX2-4:1 | 13.33234724 | 0 | 0 | -∞ | 0.00311718 | 1 | Down |
| lnc-DDX52-3:1 | 18.1002082 | 62.16334282 | 3.434399325 | 1.780057795 | 0.00515289 | 1 | Up |
| lnc-DEFB128-1:1 | 6.758852715 | 36.71140242 | 5.431602665 | 2.441377947 | 0.0043885 | 1 | Up |
| lnc-DFNB59-2:14 | 2.833802388 | 18.26442852 | 6.445201894 | 2.688225551 | 0.04570953 | 1 | Up |
| lnc-DHRS1-1:3 | 11.55266717 | 0.426025321 | 0.036876793 | -4.761142974 | 0.02159632 | 1 | Down |
| lnc-DHX35-6:2 | 0 | 6.658181925 | +∞ | +∞ | 0.02333562 | 1 | Up |
| lnc-DMKN-1:9 | 9.165095659 | 0 | 0 | -∞ | 0.03398786 | 1 | Down |
| lnc-DNAH10OS-4:1 | 19.08961222 | 2.130126607 | 0.111585641 | -3.16377671 | 0.01630264 | 1 | Down |
| lnc-DNAH10OS-8:1 | 38.48015531 | 9.908161279 | 0.257487559 | -1.957425365 | 0.01927899 | 1 | Down |
| lnc-DNAJC8-1:1 | 17.42527213 | 0 | 0 | -∞ | 0.00926909 | 1 | Down |
| lnc-DOC2B-3:2 | 4.078353494 | 29.0672688 | 7.12720681 | 2.833336788 | 0.01271972 | 1 | Up |
| lnc-DPH5-1:3 | 41.00056974 | 9.932483444 | 0.242252327 | -2.045417569 | 0.02995447 | 1 | Down |
| lnc-DPY19L3-1:1 | 2.099599523 | 17.26017063 | 8.220696587 | 3.039260647 | 0.01732415 | 1 | Up |
| lnc-DUOXA1-1:1 | 11.29917067 | 0 | 0 | -∞ | 0.00756662 | 1 | Down |
| lnc-DYDC1-1:1 | 0.951096817 | 15.37953993 | 16.17032006 | 4.015276329 | 0.00656449 | 1 | Up |
| lnc-DYNLL1-5:1 | 62.91451636 | 133.462037 | 2.12132342 | 1.084964593 | 0.026586 | 1 | Up |
| lnc-EAF2-1:1 | 10.75245066 | 0 | 0 | -∞ | 0.02843767 | 1 | Down |
| lnc-EDEM1-1:3 | 10.55655342 | 52.88205844 | 5.009405658 | 2.324639445 | 0.00711957 | 1 | Up |
| lnc-EFNB2-6:1 | 0.833787104 | 11.77048578 | 14.11689595 | 3.819350995 | 0.01752071 | 1 | Up |
| lnc-EGLN1-1:3 | 5.204699587 | 29.34108687 | 5.637421793 | 2.495035514 | 0.00729097 | 1 | Up |
| lnc-EID2B-1:1 | 4.430824154 | 19.39029724 | 4.376228115 | 2.129687942 | 0.04288895 | 1 | Up |
| lnc-EID3-1:3 | 79.0161512 | 0.492975847 | 0.006238925 | -7.3244868 | 0.01977424 | 1 | Down |
| lnc-EIF1-2:5 | 0.833787104 | 33.02938177 | 39.61368751 | 5.307927098 | 0.04408162 | 1 | Up |
| lnc-EIF1AX-1:1 | 15.41114399 | 2.130126607 | 0.138219889 | -2.854962873 | 0.04866619 | 1 | Down |
| lnc-ELF2-1:1 | 1.250680655 | 13.02423958 | 10.41372114 | 3.380413775 | 0.0210402 | 1 | Up |
| lnc-ELK1-2:3 | 58.89760176 | 16.31684729 | 0.27703755 | -1.851846561 | 0.03134407 | 1 | Down |
| lnc-ERICH1-19:8 | 58.71827643 | 21.922127 | 0.373344184 | -1.421421838 | 0.03559498 | 1 | Down |
| lnc-ETFA-3:1 | 16.7016367 | 59.07786093 | 3.537249792 | 1.822628102 | 0.01913432 | 1 | Up |
| lnc-ETNK1-8:1 | 0 | 8.94653175 | +∞ | +∞ | 0.03295376 | 1 | Up |
| lnc-EXOC2-8:4 | 6.127286326 | 23.02367355 | 3.757564495 | 1.909797867 | 0.04415801 | 1 | Up |
| lnc-EXOC3-3:1 | 23.09631376 | 5.021031165 | 0.217395348 | -2.201607027 | 0.0375029 | 1 | Down |
| lnc-EXT1-1:1 | 1.982811052 | 20.29099221 | 10.2334472 | 3.355220302 | 0.04297335 | 1 | Up |
| lnc-EXTL3-6:9 | 0.396902089 | 14.18672069 | 35.74362813 | 5.159614176 | 0.00327536 | 1 | Up |
| lnc-F13A1-2:3 | 15.18550314 | 0 | 0 | -∞ | 0.01886261 | 1 | Down |
| lnc-FAM106A-2:15 | 85.95966535 | 33.39447254 | 0.388490025 | -1.364050539 | 0.01901352 | 1 | Down |
| lnc-FAM113B-5:5 | 10.59757119 | 0.426025321 | 0.040200279 | -4.636650666 | 0.03042432 | 1 | Down |
| lnc-FAM156B-1:1 | 1.459127431 | 15.79954928 | 10.8280805 | 3.436705613 | 0.01901215 | 1 | Up |
| lnc-FAM160A1-6:3 | 7.715364301 | 0 | 0 | -∞ | 0.03871625 | 1 | Down |
| lnc-FAM160A1-8:1 | 0 | 7.668455786 | +∞ | +∞ | 0.03773428 | 1 | Up |
| lnc-FAM164A-3:1 | 0 | 11.928709 | +∞ | +∞ | 0.02547658 | 1 | Up |
| lnc-FAM174A-2:1 | 28.63177694 | 0 | 0 | -∞ | 0.03173694 | 1 | Down |
| lnc-FAM200B-1:13 | 0.18696733 | 9.299590573 | 49.73912057 | 5.636309095 | 0.04176377 | 1 | Up |
| lnc-FAM22B-1:7 | 7.224468881 | 37.20437827 | 5.149773482 | 2.364508975 | 0.01069941 | 1 | Up |
| lnc-FAM22E-1:3 | 24.75435877 | 0 | 0 | -∞ | 0.00025437 | 0.23 | Down |
| lnc-FAM27A-4:1 | 15.98280408 | 2.330978185 | 0.14584288 | -2.777513135 | 0.03948759 | 1 | Down |
| lnc-FAM27D1.1-2:2 | 73.27936062 | 11.27750993 | 0.153897494 | -2.699958359 | 0.02130087 | 1 | Down |
| lnc-FAM32A-2:1 | 0.416893552 | 24.44794079 | 58.64312529 | 5.873890087 | 0.02083037 | 1 | Up |
| lnc-FAM96A-1:1 | 1.761095999 | 14.18672069 | 8.055620305 | 3.009995685 | 0.03276712 | 1 | Up |
| lnc-FANCM-7:1 | 27.69726442 | 6.658181925 | 0.240391319 | -2.056543297 | 0.03530142 | 1 | Down |
| lnc-FBXL2-4:1 | 0 | 35.54892131 | +∞ | +∞ | 1.05E-08 | #### | Up |
| lnc-FBXO3-3:2 | 11.2417683 | 0 | 0 | -∞ | 0.00736875 | 1 | Down |
| lnc-FGA-3:1 | 23.20801949 | 3.742955201 | 0.161278527 | -2.632373726 | 0.03810587 | 1 | Down |
| lnc-FLRT2-2:10 | 0 | 13.31034788 | +∞ | +∞ | 0.0265554 | 1 | Up |
| lnc-FPR2-1:10 | 11.57072811 | 34.87340008 | 3.013933069 | 1.591647379 | 0.04653553 | 1 | Up |
| lnc-FRYL-3:3 | 0 | 10.22460771 | +∞ | +∞ | 0.02926433 | 1 | Up |
| lnc-FRYL-3:4 | 26.65137513 | 6.524280873 | 0.24480091 | -2.030319177 | 0.04413789 | 1 | Down |
| lnc-FSD1L-3:1 | 50.29002446 | 0 | 0 | -∞ | 0.02591617 | 1 | Down |
| lnc-FTSJ1-2:1 | 2.376516595 | 23.62622828 | 9.941537262 | 3.313468953 | 0.0030704 | 1 | Up |
| lnc-FUK-2:1 | 27.18052695 | 66.55148111 | 2.448498561 | 1.291897348 | 0.04352506 | 1 | Up |
| lnc-FXYD4-4:2 | 14.73457034 | 49.45554967 | 3.356429712 | 1.746927431 | 0.03794764 | 1 | Up |
| lnc-GAS8-1:4 | 8.072207181 | 31.83028828 | 3.943195159 | 1.979365115 | 0.01795442 | 1 | Up |
| lnc-GBP5-2:6 | 40.81133885 | 2.69005298 | 0.065914353 | -3.923263548 | 7.34E-05 | 0.1 | Down |
| lnc-GET4-1:2 | 27.33873767 | 4.260253214 | 0.155832111 | -2.681935543 | 0.01172486 | 1 | Down |
| lnc-GET4-2:1 | 54.96376394 | 16.6576159 | 0.303065414 | -1.722298873 | 0.01833 | 1 | Down |
| lnc-GFAP-4:1 | 12.44331129 | 1.278075964 | 0.102711885 | -3.28332496 | 0.04976158 | 1 | Down |
| lnc-GFM1-2:1 | 0 | 24.43565057 | +∞ | +∞ | 1.96E-06 | 0.01 | Up |
| lnc-GGCT-1:12 | 7.90933328 | 36.80869108 | 4.653829821 | 2.218418456 | 0.00733059 | 1 | Up |
| lnc-GGCT-1:22 | 11.3605419 | 0 | 0 | -∞ | 0.00745582 | 1 | Down |
| lnc-GGCT-1:32 | 67.49720294 | 18.03925477 | 0.267259294 | -1.903687978 | 0.00341994 | 1 | Down |
| lnc-GIMAP5-2:1 | 3.121558612 | 16.83414531 | 5.392865359 | 2.431052015 | 0.0418209 | 1 | Up |
| lnc-GIMAP6-5:1 | 52.71100402 | 19.68242151 | 0.373402516 | -1.421196447 | 0.04677756 | 1 | Down |
| lnc-GMDS-14:1 | 18.62407812 | 2.330978185 | 0.125159386 | -2.998161607 | 0.01887146 | 1 | Down |
| lnc-GNA14-3:1 | 28.33319905 | 455.6297441 | 16.081126 | 4.007296523 | 7.16E-20 | #### | Up |
| lnc-GNLY-2:3 | 58.57719426 | 12.46431321 | 0.212784401 | -2.232535701 | 0.00239963 | 0.92 | Down |
| lnc-GOLGA8A-2:2 | 23.53137687 | 2.98217725 | 0.126731949 | -2.980147824 | 0.01920224 | 1 | Down |
| lnc-GOLGA8F-6:3 | 106.6191867 | 44.96410674 | 0.421726222 | -1.245621368 | 0.03470546 | 1 | Down |
| lnc-GOLGA8IP-2:1 | 47.01073218 | 13.31636385 | 0.283262209 | -1.819789955 | 0.0229425 | 1 | Down |
| lnc-GPR152-1:7 | 7.657785195 | 29.18286366 | 3.810875196 | 1.930122362 | 0.03036863 | 1 | Up |
| lnc-GRID2-1:1 | 1.121803981 | 12.37304052 | 11.02959227 | 3.463307555 | 0.02902894 | 1 | Up |
| lnc-GRK5-2:1 | 21.53721408 | 107.5048305 | 4.991584805 | 2.319497937 | 3.10E-05 | 0.05 | Up |
| lnc-GSDMD-1:1 | 50.48569451 | 12.7504215 | 0.252555137 | -1.985329707 | 0.04089231 | 1 | Down |
| lnc-GSR-3:1 | 10.32974467 | 32.29894196 | 3.126789965 | 1.644682313 | 0.04777914 | 1 | Up |
| lnc-GTPBP1-1:1 | 2.354142325 | 15.44649046 | 6.561408922 | 2.714005636 | 0.0315521 | 1 | Up |
| lnc-GUSB-12:1 | 22.63420988 | 1.704101286 | 0.075288746 | -3.731421954 | 0.00364477 | 1 | Down |
| lnc-HAO2-2:2 | 63.97742176 | 21.54474601 | 0.336755459 | -1.570226764 | 0.0229854 | 1 | Down |
| lnc-HAX1-4:1 | 23.59900691 | 70.30672653 | 2.979223949 | 1.574936575 | 0.02257046 | 1 | Up |
| lnc-HEATR2-3:1 | 2.173289099 | 13.94324075 | 6.415732153 | 2.681613913 | 0.04344843 | 1 | Up |
| lnc-HES5-1:7 | 18.16438396 | 53.36901831 | 2.938113312 | 1.554890036 | 0.01786705 | 1 | Up |
| lnc-HIST1H2AI-1:3 | 1.373209648 | 19.43894157 | 14.15584401 | 3.823325864 | 0.03132319 | 1 | Up |
| lnc-HIST1H4C-2:1 | 14.77686955 | 1.971903389 | 0.133445273 | -2.905679894 | 0.03791949 | 1 | Down |
| lnc-HIVEP3-1:1 | 5.936547212 | 35.9992688 | 6.064007834 | 2.600271617 | 0.00220072 | 0.88 | Up |
| lnc-HIVEP3-1:2 | 62.54505142 | 18.11222127 | 0.2895868 | -1.787932254 | 0.00923358 | 1 | Down |
| lnc-HJURP-12:1 | 609.3543905 | 84.14614609 | 0.138090654 | -2.856312417 | 0.00856606 | 1 | Down |
| lnc-HMMR-1:2 | 29.81199237 | 5.221882743 | 0.175160475 | -2.513250828 | 0.00957773 | 1 | Down |
| lnc-HNRNPD-2:2 | 53.79976551 | 0 | 0 | -∞ | 0.01208099 | 1 | Down |
| lnc-HPRT1-7:2 | 1.66906219 | 12.93296689 | 7.748642898 | 2.953943658 | 0.03618761 | 1 | Up |
| lnc-HSFY2-13:1 | 80.77327021 | 0.492975847 | 0.006103205 | -7.356217174 | 0.00965107 | 1 | Down |
| lnc-HSPA4-5:1 | 455.4515175 | 1.278075964 | 0.002806173 | -8.477180091 | 1.28E-07 | 0 | Down |
| lnc-HSPG2-2:1 | 0 | 8.094481107 | +∞ | +∞ | 0.03599054 | 1 | Up |
| lnc-HTRA2-1:1 | 0 | 11.928709 | +∞ | +∞ | 0.02547658 | 1 | Up |
| lnc-HTRA4-2:1 | 0 | 7.887613557 | +∞ | +∞ | 0.04256077 | 1 | Up |
| lnc-HYOU1-1:3 | 12.63623583 | 0 | 0 | -∞ | 0.04760022 | 1 | Down |
| lnc-IFI44-8:1 | 9.99480682 | 33.91177055 | 3.39293907 | 1.762535522 | 0.03288286 | 1 | Up |
| lnc-IFT52-1:2 | 60.95979555 | 138.8238368 | 2.277301548 | 1.187325338 | 0.01486753 | 1 | Up |
| lnc-IGF2BP3-2:4 | 39.43241427 | 143.1512988 | 3.630295061 | 1.860086812 | 0.02266328 | 1 | Up |
| lnc-IGFBP7-1:2 | 33.17090929 | 0.492975847 | 0.014861692 | -6.072257785 | 0.00319614 | 1 | Down |
| lnc-IL18R1-3:1 | 225.1289181 | 53.28376159 | 0.236681107 | -2.078983546 | 0.04454332 | 1 | Down |
| lnc-IL22RA2-1:2 | 17.47003075 | 0.492975847 | 0.028218373 | -5.147221372 | 0.04645819 | 1 | Down |
| lnc-IL32-1:3 | 59.93220062 | 151.8240125 | 2.533262769 | 1.340996732 | 0.00694528 | 1 | Up |
| lnc-ING2-5:2 | 23.72273326 | 1.478927542 | 0.062342207 | -4.003646966 | 0.00166834 | 0.71 | Down |
| lnc-INTS2-4:2 | 0 | 7.887613557 | +∞ | +∞ | 0.04256077 | 1 | Up |
| lnc-IRAK1-1:1 | 6.99147836 | 26.18238021 | 3.744898985 | 1.904926804 | 0.03446132 | 1 | Up |
| lnc-IRF2-2:3 | 0 | 16.2985411 | +∞ | +∞ | 0.00012728 | 0.14 | Up |
| lnc-JMJD6-1:5 | 112.6839469 | 53.88030035 | 0.478154181 | -1.064452203 | 0.04740426 | 1 | Down |
| lnc-JMJD6-1:6 | 42.39240563 | 3.742955201 | 0.08829306 | -3.501556151 | 0.00162991 | 0.71 | Down |
| lnc-JMJD7-PLA2G4B-2:3 | 18.43454065 | 0.492975847 | 0.026741965 | -5.224750692 | 0.04111107 | 1 | Down |
| lnc-KBTBD4-3:1 | 13.00388075 | 41.79938411 | 3.214377687 | 1.684539454 | 0.0263775 | 1 | Up |
| lnc-KCND3-3:1 | 171.9738678 | 437.8946455 | 2.546285963 | 1.348394452 | 0.00079434 | 0.44 | Up |
| lnc-KDM5D-1:1 | 73.57838839 | 0 | 0 | -∞ | 0.00441682 | 1 | Down |
| lnc-KDM5D-4:1 | 21.86152741 | 0 | 0 | -∞ | 0.01413906 | 1 | Down |
| lnc-KIAA0226-4:1 | 0 | 15.76293689 | +∞ | +∞ | 0.01975143 | 1 | Up |
| lnc-KIAA0240-3:1 | 107.0038879 | 0 | 0 | -∞ | 0.00217932 | 0.88 | Down |
| lnc-KIAA0355-5:1 | 64.31772512 | 152.706143 | 2.374246644 | 1.247469814 | 0.00768858 | 1 | Up |
| lnc-KIAA0754-3:1 | 103.7028878 | 47.16719985 | 0.454830148 | -1.136600209 | 0.04906818 | 1 | Down |
| lnc-KIAA0754-5:1 | 10.14940301 | 0 | 0 | -∞ | 0.01279544 | 1 | Down |
| lnc-KLB-2:1 | 241.0409106 | 67.75659058 | 0.281099961 | -1.830844841 | 0.01086359 | 1 | Down |
| lnc-KLC2-2:1 | 111.7165681 | 223.8892423 | 2.004082708 | 1.00294205 | 0.0196963 | 1 | Up |
| lnc-KLF13-2:4 | 4.020109869 | 17.93569186 | 4.461492956 | 2.157526562 | 0.04899521 | 1 | Up |
| lnc-KLF14-1:1 | 0 | 46.99668641 | +∞ | +∞ | 0.0049979 | 1 | Up |
| lnc-KLF14-1:6 | 0 | 25.92686833 | +∞ | +∞ | 0.00693993 | 1 | Up |
| lnc-KLHDC1-2:5 | 4.742231251 | 27.2596046 | 5.748265564 | 2.523126714 | 0.00830764 | 1 | Up |
| lnc-KLHL5-2:1 | 32.38468312 | 80.87811882 | 2.497418873 | 1.320437814 | 0.01855406 | 1 | Up |
| lnc-KLHL9-1:1 | 38.07005872 | 12.08091625 | 0.317333796 | -1.655926919 | 0.03822614 | 1 | Down |
| lnc-KLRG1-3:2 | 290.3234186 | 124.0042232 | 0.427124425 | -1.227271694 | 0.00925815 | 1 | Down |
| lnc-KLRG1-5:1 | 109.5195433 | 47.5872092 | 0.434508835 | -1.202542582 | 0.0304387 | 1 | Down |
| lnc-L3MBTL4-4:1 | 7.125911697 | 26.42586015 | 3.708418133 | 1.89080392 | 0.04420621 | 1 | Up |
| lnc-LAG3-1:1 | 0.77225957 | 12.54956993 | 16.2504557 | 4.02240827 | 0.02518755 | 1 | Up |
| lnc-LAT-1:9 | 32.51342524 | 104.2548512 | 3.206517013 | 1.681007062 | 0.00162203 | 0.71 | Up |
| lnc-LGALS3BP-3:1 | 5.909852269 | 23.86970822 | 4.038968681 | 2.013986959 | 0.04095444 | 1 | Up |
| lnc-LILRB5-1:1 | 125.9444603 | 36.76004675 | 0.291875059 | -1.776577159 | 0.01515963 | 1 | Down |
| lnc-LIMA1-1:7 | 55.80452562 | 0 | 0 | -∞ | 0.00011559 | 0.13 | Down |
| lnc-LIPI-9:1 | 5.876480571 | 22.39679665 | 3.811260223 | 1.930268115 | 0.04449048 | 1 | Up |
| lnc-LONRF2-3:1 | 2.099347593 | 13.98586911 | 6.662007357 | 2.735956947 | 0.03861319 | 1 | Up |
| lnc-LPCAT1-3:2 | 18.49710161 | 46.75948072 | 2.527935549 | 1.337959682 | 0.0464307 | 1 | Up |
| lnc-LPP-2:1 | 135.0476409 | 0.985951695 | 0.007300769 | -7.097735757 | 6.52E-11 | #### | Down |
| lnc-LPP-2:2 | 165.300767 | 67.04471524 | 0.405592282 | -1.301897897 | 0.03075958 | 1 | Down |
| lnc-LPP-6:3 | 14.86912674 | 0 | 0 | -∞ | 0.00211869 | 0.87 | Down |
| lnc-LRCH4-2:1 | 46.24131681 | 99.1122092 | 2.143369092 | 1.099880306 | 0.03986016 | 1 | Up |
| lnc-LRRC56-3:4 | 0.979811522 | 10.98538567 | 11.2117335 | 3.486937453 | 0.03273419 | 1 | Up |
| lnc-LRRFIP2-1:1 | 7.6984791 | 28.62895325 | 3.718780409 | 1.89482956 | 0.02724058 | 1 | Up |
| lnc-LST1-1:5 | 26.24104446 | 0 | 0 | -∞ | 0.00029096 | 0.24 | Down |
| lnc-LYAR-2:1 | 72.67784773 | 10.44376549 | 0.143699433 | -2.798873729 | 0.00086676 | 0.46 | Down |
| lnc-MAGEB18-3:1 | 44.46980116 | 6.792082977 | 0.152734728 | -2.710899965 | 0.00131556 | 0.61 | Down |
| lnc-MAP3K3-2:2 | 50.44243127 | 190.0080682 | 3.766830096 | 1.913350961 | 0.0004987 | 0.34 | Up |
| lnc-MAPK6-4:2 | 0 | 6.524280873 | +∞ | +∞ | 0.02444662 | 1 | Up |
| lnc-MARCH7-1:1 | 196.0074242 | 29.57227659 | 0.150873247 | -2.728591084 | 1.77E-06 | 0.01 | Down |
| lnc-MARCH7-1:2 | 100.5988689 | 296.5091574 | 2.94744027 | 1.559462577 | 0.02661757 | 1 | Up |
| lnc-MBOAT2-7:1 | 0 | 5.647908064 | +∞ | +∞ | 0.04334636 | 1 | Up |
| lnc-MDH1B-8:2 | 0 | 6.901661863 | +∞ | +∞ | 0.04787287 | 1 | Up |
| lnc-MDK-4:2 | 25.09267986 | 60.07584457 | 2.394158173 | 1.259518469 | 0.03945499 | 1 | Up |
| lnc-METRNL-7:4 | 2.518362806 | 17.77145267 | 7.056748388 | 2.819003571 | 0.03742823 | 1 | Up |
| lnc-METTL14-2:3 | 96.30886092 | 231.1015929 | 2.399588062 | 1.262786759 | 0.02448703 | 1 | Up |
| lnc-MFN1-1:3 | 25.14399661 | 4.595005844 | 0.182747632 | -2.452075382 | 0.03629009 | 1 | Down |
| lnc-MFSD2A-4:2 | 13.95899416 | 0.919001169 | 0.065835773 | -3.924984482 | 0.02852215 | 1 | Down |
| lnc-MICA-9:1 | 57.12399992 | 21.31957226 | 0.373215676 | -1.421918509 | 0.04694377 | 1 | Down |
| lnc-MMP23B-5:2 | 21.72377641 | 0 | 0 | -∞ | 0.00026462 | 0.23 | Down |
| lnc-MOGAT2-3:1 | 33.52115121 | 75.86310363 | 2.263141357 | 1.178326699 | 0.02927145 | 1 | Up |
| lnc-MPPE1-5:1 | 0 | 93.37878614 | +∞ | +∞ | 1.50E-05 | 0.02 | Up |
| lnc-MPZL3-1:3 | 688.259201 | 290.7885411 | 0.422498589 | -1.242981572 | 0.01004795 | 1 | Down |
| lnc-MRFAP1L1-1:1 | 81.06728847 | 27.99606038 | 0.345343491 | -1.533896064 | 0.01342853 | 1 | Down |
| lnc-MTA2-4:1 | 10.84007649 | 38.32423101 | 3.535420717 | 1.821881907 | 0.02079142 | 1 | Up |
| lnc-MTIF2-1:1 | 13.79536436 | 43.97815505 | 3.187893695 | 1.672603521 | 0.0198218 | 1 | Up |
| lnc-MTPN-1:1 | 256.9150483 | 30.07152669 | 0.117048522 | -3.094821377 | 0.01359921 | 1 | Down |
| lnc-MTR-4:1 | 0.825982805 | 9.707309701 | 11.75243557 | 3.554887867 | 0.03958861 | 1 | Up |
| lnc-MYO5B-2:1 | 84.52108414 | 34.49601909 | 0.408135076 | -1.292881391 | 0.03232902 | 1 | Down |
| lnc-N6AMT1-1:5 | 112.0547854 | 50.05810441 | 0.446728841 | -1.162528697 | 0.04042763 | 1 | Down |
| lnc-NAT1-6:1 | 14.20372148 | 44.91546241 | 3.162231988 | 1.660943211 | 0.02019583 | 1 | Up |
| lnc-NBPF15-4:5 | 58.19660201 | 0.426025321 | 0.00732045 | -7.093851927 | 0.00892606 | 1 | Down |
| lnc-NCDN-2:1 | 37.7119233 | 83.89690846 | 2.224678593 | 1.15359692 | 0.0420892 | 1 | Up |
| lnc-NDRG1-1:4 | 0 | 53.73436736 | +∞ | +∞ | 0.00704764 | 1 | Up |
| lnc-NDUFB8-7:1 | 15.88657078 | 48.46959798 | 3.050979262 | 1.609272374 | 0.04526875 | 1 | Up |
| lnc-NFKB2-3:1 | 9.827818478 | 32.07376821 | 3.263569457 | 1.706450744 | 0.04174657 | 1 | Up |
| lnc-NKAIN3-1:2 | 5.781610291 | 23.98530308 | 4.148550641 | 2.052607397 | 0.03190399 | 1 | Up |
| lnc-NMRAL1-2:1 | 16.23256198 | 51.79881808 | 3.191043912 | 1.674028462 | 0.02698583 | 1 | Up |
| lnc-NPBWR1-1:2 | 5.429415204 | 21.922127 | 4.03765897 | 2.013519062 | 0.04740877 | 1 | Up |
| lnc-NR5A1-4:1 | 2.501361311 | 18.91562758 | 7.562133268 | 2.918793274 | 0.01425824 | 1 | Up |
| lnc-NRARP-3:1 | 1.37164058 | 13.74238917 | 10.01894328 | 3.324658448 | 0.02095121 | 1 | Up |
| lnc-NSA2-4:1 | 9.171054871 | 0 | 0 | -∞ | 0.02324278 | 1 | Down |
| lnc-NT5C-3:3 | 0 | 83.07493768 | +∞ | +∞ | 0.0040078 | 1 | Up |
| lnc-OLFML3-5:1 | 2.190102383 | 16.27421893 | 7.430802808 | 2.893518085 | 0.02226074 | 1 | Up |
| lnc-OPRD1-4:1 | 3.421052197 | 18.40434554 | 5.379732457 | 2.427534427 | 0.02993533 | 1 | Up |
| lnc-OR2AT4-1:1 | 7.46035814 | 27.14400974 | 3.63843253 | 1.863317058 | 0.04414228 | 1 | Up |
| lnc-OR4F16-5:16 | 22.25424985 | 0.852050643 | 0.038287098 | -4.706997879 | 0.00428039 | 1 | Down |
| lnc-OR4F29-3:4 | 13.99863435 | 0 | 0 | -∞ | 0.02616516 | 1 | Down |
| lnc-P2RX7-1:2 | 31.32819697 | 0 | 0 | -∞ | 6.26E-05 | 0.09 | Down |
| lnc-PAIP2-1:1 | 0 | 10.55936034 | +∞ | +∞ | 0.00274195 | 1 | Up |
| lnc-PAPOLA-4:1 | 7.380594502 | 34.80644955 | 4.715941181 | 2.237545725 | 0.03082747 | 1 | Up |
| lnc-PCBP3-3:9 | 1.17058448 | 12.03828789 | 10.28399752 | 3.362329262 | 0.0299843 | 1 | Up |
| lnc-PCCB-1:1 | 46.48048464 | 6.299107129 | 0.135521546 | -2.883405859 | 0.00944376 | 1 | Down |
| lnc-PCF11-3:1 | 0 | 8.337961045 | +∞ | +∞ | 0.01001032 | 1 | Up |
| lnc-PCID2-1:2 | 0 | 8.873565252 | +∞ | +∞ | 0.03833018 | 1 | Up |
| lnc-PDHX-5:3 | 30.56914568 | 7.01725672 | 0.229553576 | -2.123097185 | 0.03489462 | 1 | Down |
| lnc-PDK3-12:1 | 0 | 6.901661863 | +∞ | +∞ | 0.04787287 | 1 | Up |
| lnc-PFKP-27:2 | 0.975487066 | 41.16623296 | 42.20069582 | 5.399194882 | 0.0218148 | 1 | Up |
| lnc-PGPEP1L-3:1 | 25.98984106 | 61.17739112 | 2.353896316 | 1.235050774 | 0.0398239 | 1 | Up |
| lnc-PHB-3:1 | 797.1574179 | 305.8511181 | 0.38367719 | -1.382035097 | 0.00114696 | 0.56 | Down |
| lnc-PHLDA1-1:2 | 129.7151573 | 0 | 0 | -∞ | 0.03183867 | 1 | Down |
| lnc-PHLDA3-2:1 | 0 | 8.873565252 | +∞ | +∞ | 0.03833018 | 1 | Up |
| lnc-PIN4-1:1 | 0 | 6.457330347 | +∞ | +∞ | 0.02502655 | 1 | Up |
| lnc-PKD2L1-5:1 | 0 | 5.538329178 | +∞ | +∞ | 0.04993031 | 1 | Up |
| lnc-PKLR-7:1 | 5.504431595 | 24.92261044 | 4.527735519 | 2.178789688 | 0.02768633 | 1 | Up |
| lnc-PLCG2-5:1 | 0 | 13.80332373 | +∞ | +∞ | 0.0256832 | 1 | Up |
| lnc-PLEKHA7-2:1 | 44.81093991 | 113.4022345 | 2.530681899 | 1.339526176 | 0.00871041 | 1 | Up |
| lnc-PLEKHG2-1:1 | 14.95119162 | 76.52057694 | 5.118025297 | 2.355587278 | 0.00073513 | 0.42 | Up |
| lnc-PLEKHG5-3:1 | 0 | 12.68347098 | +∞ | +∞ | 0.01210329 | 1 | Up |
| lnc-PMS2-3:1 | 55.05857966 | 17.57661706 | 0.319234844 | -1.647309969 | 0.03934636 | 1 | Down |
| lnc-POFUT2-4:1 | 51.09539668 | 130.3765551 | 2.551630158 | 1.351419235 | 0.00777981 | 1 | Up |
| lnc-POFUT2-5:1 | 112.4636409 | 344.3822166 | 3.062164927 | 1.614551988 | 0.00014584 | 0.15 | Up |
| lnc-POTEF-8:2 | 0.18696733 | 15.82988742 | 84.66659609 | 6.403720983 | 0.02500681 | 1 | Up |
| lnc-PPIAL4C-5:2 | 2.071835129 | 18.47129607 | 8.91542759 | 3.156303992 | 0.00902863 | 1 | Up |
| lnc-PPIAL4C-5:3 | 2.071835129 | 18.47129607 | 8.91542759 | 3.156303992 | 0.00902863 | 1 | Up |
| lnc-PPIAL4G-8:1 | 19.74266288 | 2.69005298 | 0.136255833 | -2.875610101 | 0.025067 | 1 | Down |
| lnc-PRAGMIN.1-3:2 | 1.416901194 | 13.58416595 | 9.587235873 | 3.261114927 | 0.0229402 | 1 | Up |
| lnc-PRDM11-9:2 | 0 | 10.65063304 | +∞ | +∞ | 0.02821406 | 1 | Up |
| lnc-PRKCZ-1:1 | 14.65544624 | 46.01700896 | 3.139925473 | 1.650730317 | 0.02055733 | 1 | Up |
| lnc-PRKRIR-1:5 | 16.21794234 | 53.70377094 | 3.311380065 | 1.727432606 | 0.0114333 | 1 | Up |
| lnc-PRPF4B-1:2 | 64.50496749 | 9.798582393 | 0.151904307 | -2.718765318 | 0.00066689 | 0.39 | Down |
| lnc-PRPF4B-4:4 | 3.242882949 | 20.75362992 | 6.399746845 | 2.678014837 | 0.02011332 | 1 | Up |
| lnc-PRSS55-4:1 | 0.839739037 | 9.975111804 | 11.87882349 | 3.57032005 | 0.03754959 | 1 | Up |
| lnc-PSD4-5:1 | 48.72635082 | 15.5986977 | 0.320128584 | -1.643276594 | 0.0242063 | 1 | Down |
| lnc-PSMB9-6:4 | 54.06193802 | 9.99943397 | 0.184962551 | -2.434694894 | 0.01148346 | 1 | Down |
| lnc-PSME1-1:3 | 23.08609486 | 2.890904558 | 0.125222762 | -2.997431269 | 0.00809768 | 1 | Down |
| lnc-PSMG1-11:1 | 22.82024179 | 2.556151929 | 0.112012482 | -3.158268585 | 0.00872362 | 1 | Down |
| lnc-PTBP3-6:1 | 0 | 9.859516947 | +∞ | +∞ | 0.0348774 | 1 | Up |
| lnc-PTEN-2:1 | 0.74786932 | 12.73211531 | 17.02451881 | 4.089542116 | 0.01393084 | 1 | Up |
| lnc-PTGER1-1:2 | 13.77235881 | 43.89289833 | 3.187028376 | 1.672211865 | 0.01927502 | 1 | Up |
| lnc-PTP4A2-3:4 | 86.93197643 | 0 | 0 | -∞ | 0.00391119 | 1 | Down |
| lnc-PTPN12-1:1 | 9.066696802 | 34.83678769 | 3.842279989 | 1.941962653 | 0.01589434 | 1 | Up |
| lnc-PUF60-1:1 | 0.629804278 | 12.823388 | 20.36090966 | 4.347730112 | 0.00813906 | 1 | Up |
| lnc-PXDC1-14:4 | 10.09547462 | 0 | 0 | -∞ | 0.04002092 | 1 | Down |
| lnc-QRICH2-1:1 | 2.862663341 | 20.10844683 | 7.024384091 | 2.812371733 | 0.01174574 | 1 | Up |
| lnc-RAB23-17:1 | 15.68103048 | 0 | 0 | -∞ | 0.00112857 | 0.56 | Down |
| lnc-RAB3D-2:2 | 0 | 8.380589405 | +∞ | +∞ | 0.04033241 | 1 | Up |
| lnc-RABGEF1-1:1 | 19.15759601 | 62.8997986 | 3.283282442 | 1.715138862 | 0.00678753 | 1 | Up |
| lnc-RAD52-2:5 | 0 | 6.390379821 | +∞ | +∞ | 0.04418926 | 1 | Up |
| lnc-RAP1GAP2-3:1 | 18.80641639 | 47.5872092 | 2.530370922 | 1.339348882 | 0.04784931 | 1 | Up |
| lnc-RAP2C-4:1 | 154.8801496 | 0 | 0 | -∞ | 0.00137688 | 0.63 | Down |
| lnc-RBAK-2:10 | 7.048301612 | 46.18124815 | 6.552110096 | 2.711959599 | 0.00052507 | 0.34 | Up |
| lnc-RBM28-2:1 | 8.503852687 | 0 | 0 | -∞ | 0.02798176 | 1 | Down |
| lnc-RBPMS2-1:2 | 0 | 6.390379821 | +∞ | +∞ | 0.04418926 | 1 | Up |
| lnc-RC3H1-2:1 | 457.1117224 | 6.883355669 | 0.015058366 | -6.053290949 | 6.80E-09 | #### | Down |
| lnc-RCN1-2:1 | 1.38376258 | 13.09119011 | 9.460575312 | 3.241927919 | 0.02712265 | 1 | Up |
| lnc-RFXAP-4:6 | 43.29960707 | 9.03178847 | 0.208588232 | -2.26127033 | 0.00442245 | 1 | Down |
| lnc-RGMB-1:2 | 143.6638288 | 0 | 0 | -∞ | 3.01E-07 | 0 | Down |
| lnc-RGS21-1:3 | 85.28073031 | 35.81070744 | 0.419915581 | -1.251828773 | 0.02518092 | 1 | Down |
| lnc-RHCE-2:2 | 25.36084854 | 6.341735489 | 0.250060067 | -1.999653407 | 0.03674064 | 1 | Down |
| lnc-RHOXF1-3:15 | 7.241383046 | 0 | 0 | -∞ | 0.04753533 | 1 | Down |
| lnc-RHOXF1-3:16 | 7.241383046 | 0 | 0 | -∞ | 0.04753533 | 1 | Down |
| lnc-RIPK1-1:2 | 38.82240118 | 86.13661395 | 2.218734837 | 1.14973726 | 0.04144008 | 1 | Up |
| lnc-RNF121-1:1 | 12.13615429 | 0 | 0 | -∞ | 0.02429197 | 1 | Down |
| lnc-RNF150-2:1 | 90.0438169 | 17.61322945 | 0.195607317 | -2.353967756 | 0.01781957 | 1 | Down |
| lnc-RNF150-2:2 | 193.179045 | 64.19016479 | 0.33228327 | -1.589514437 | 0.0014872 | 0.67 | Down |
| lnc-RNF19A-8:4 | 89.62127214 | 24.28344332 | 0.270956244 | -1.883868199 | 0.01523909 | 1 | Down |
| lnc-RNF219-6:1 | 391.9286391 | 186.6730904 | 0.476293569 | -1.070077025 | 0.03279754 | 1 | Down |
| lnc-RNF24-4:1 | 18.42989385 | 55.99212077 | 3.038114121 | 1.603176063 | 0.01490098 | 1 | Up |
| lnc-ROPN1L-10:5 | 43.51321013 | 191.2249513 | 4.394641323 | 2.135745422 | 0.01455344 | 1 | Up |
| lnc-ROPN1L-3:1 | 6.914372151 | 28.62895325 | 4.140499329 | 2.049804762 | 0.02254651 | 1 | Up |
| lnc-RP11-108K14.4.1-1:2 | 53.3183429 | 19.65809934 | 0.368692991 | -1.439508103 | 0.04182139 | 1 | Down |
| lnc-RP11-158I9.5.1-2:1 | 22.37927707 | 0 | 0 | -∞ | 0.02303812 | 1 | Down |
| lnc-RP11-347C12.1.1-1:3 | 98.33108203 | 27.72224231 | 0.281927563 | -1.826603564 | 0.00898906 | 1 | Down |
| lnc-RP11-389E17.1.1-1:1 | 1.844900796 | 14.25367121 | 7.725982474 | 2.949718405 | 0.03171327 | 1 | Up |
| lnc-RP11-408E5.4.1-12:1 | 9.440945632 | 0 | 0 | -∞ | 0.02879408 | 1 | Down |
| lnc-RP11-408E5.4.1-14:1 | 9.900551484 | 0 | 0 | -∞ | 0.01770551 | 1 | Down |
| lnc-RP11-63E5.6.1-2:2 | 10.51797644 | 0.426025321 | 0.040504495 | -4.625774179 | 0.03089196 | 1 | Down |
| lnc-RP11-645C24.1.1-3:8 | 108.6463036 | 47.16719985 | 0.434135339 | -1.20378323 | 0.03686027 | 1 | Down |
| lnc-RP11-664D7.4.1-7:2 | 24.61160277 | 69.57027075 | 2.826726541 | 1.499132323 | 0.02198047 | 1 | Up |
| lnc-RP11-706O15.1.1-2:8 | 4.327003298 | 29.15854149 | 6.738737986 | 2.752478432 | 0.00408666 | 1 | Up |
| lnc-RP11-712L6.5.1-2:3 | 11.8141716 | 0 | 0 | -∞ | 0.03583337 | 1 | Down |
| lnc-RP11-796G6.2.1-4:2 | 26.88196154 | 0.852050643 | 0.031696 | -4.979555421 | 0.00020549 | 0.2 | Down |
| lnc-RP3-369A17.5.1-3:1 | 175.2479444 | 372.055298 | 2.123022324 | 1.086119541 | 0.00701565 | 1 | Up |
| lnc-RP3-377D14.1.1-7:10 | 1.167673676 | 10.82716245 | 9.272421457 | 3.212946142 | 0.04644511 | 1 | Up |
| lnc-RPL10-1:1 | 7.657974902 | 0 | 0 | -∞ | 0.03928519 | 1 | Down |
| lnc-RPL10L-2:1 | 15.31353328 | 1.411977016 | 0.092204522 | -3.439018683 | 0.02805324 | 1 | Down |
| lnc-RPL23A-2:1 | 11.0962719 | 32.28063576 | 2.909142464 | 1.540593948 | 0.04670623 | 1 | Up |
| lnc-RPRML-3:20 | 0.396902089 | 9.99943397 | 25.19370454 | 4.65499137 | 0.01684815 | 1 | Up |
| lnc-RPUSD2-2:1 | 16.20050261 | 45.57267745 | 2.813040963 | 1.492130561 | 0.03096438 | 1 | Up |
| lnc-RRM2B-7:3 | 20.11872211 | 4.102029996 | 0.20389118 | -2.294128725 | 0.04756756 | 1 | Down |
| lnc-RUNDC3A-4:2 | 7.194357397 | 31.22773354 | 4.34058691 | 2.117890129 | 0.0129622 | 1 | Up |
| lnc-SAG-7:2 | 5.76085433 | 24.70345267 | 4.288157842 | 2.100358011 | 0.02667676 | 1 | Up |
| lnc-SAMD8-1:1 | 59.39972384 | 128.465328 | 2.162726015 | 1.112850909 | 0.02246653 | 1 | Up |
| lnc-SATB1-8:5 | 0 | 6.633859759 | +∞ | +∞ | 0.02580271 | 1 | Up |
| lnc-SBDS-4:1 | 5.381121232 | 60.1427951 | 11.1766289 | 3.482413203 | 3.09E-06 | 0.01 | Up |
| lnc-SCAPER-7:1 | 32.38476526 | 71.95590924 | 2.221906154 | 1.151797884 | 0.04091361 | 1 | Up |
| lnc-SEC11A-1:3 | 24.50916612 | 83.03256759 | 3.387816917 | 1.760355911 | 0.00209921 | 0.87 | Up |
| lnc-SEC11A-2:1 | 27.21008155 | 87.02501871 | 3.198263796 | 1.677288939 | 0.0026622 | 0.99 | Up |
| lnc-SECISBP2L-2:1 | 8.990011889 | 0 | 0 | -∞ | 0.03325261 | 1 | Down |
| lnc-SERPINB6-1:7 | 25.63130847 | 74.57901169 | 2.909684138 | 1.540862549 | 0.0079007 | 1 | Up |
| lnc-SERPINB6-5:2 | 20.33266234 | 2.556151929 | 0.125716539 | -2.99175364 | 0.02745465 | 1 | Down |
| lnc-SETD5-1:1 | 20.55637161 | 0.985951695 | 0.047963313 | -4.381924862 | 0.00670481 | 1 | Down |
| lnc-SFPQ-2:4 | 225.789773 | 489.2125197 | 2.166672623 | 1.115481184 | 0.00341661 | 1 | Up |
| lnc-SH2B2-7:1 | 28.89377159 | 65.87595988 | 2.279936341 | 1.188993543 | 0.04819105 | 1 | Up |
| lnc-SH3BGR-2:1 | 18.69151509 | 59.28472848 | 3.17174548 | 1.665277005 | 0.01148051 | 1 | Up |
| lnc-SHFM1-4:2 | 0 | 8.94653175 | +∞ | +∞ | 0.03295376 | 1 | Up |
| lnc-SIX5-1:1 | 3.667449996 | 17.64356759 | 4.810854302 | 2.266293108 | 0.03862127 | 1 | Up |
| lnc-SLC12A8-1:1 | 0.977649294 | 10.37681496 | 10.61404639 | 3.407902854 | 0.04696261 | 1 | Up |
| lnc-SLC17A9-5:1 | 12.66346648 | 1.278075964 | 0.100926233 | -3.308626887 | 0.04654767 | 1 | Down |
| lnc-SLC26A11-1:1 | 22.20341236 | 70.20316362 | 3.161818665 | 1.660754629 | 0.00713903 | 1 | Up |
| lnc-SLC35E3-3:4 | 50.92859314 | 0 | 0 | -∞ | 2.46E-06 | 0.01 | Down |
| lnc-SLC35E3-8:1 | 1.593964714 | 41.32445618 | 25.92557778 | 4.696304236 | 0.04983128 | 1 | Up |
| lnc-SLC7A6OS-4:2 | 5.234563492 | 22.88375653 | 4.371664717 | 2.128182758 | 0.03806214 | 1 | Up |
| lnc-SLC9A7-1:2 | 0 | 12.37304052 | +∞ | +∞ | 0.00113438 | 0.56 | Up |
| lnc-SMYD1-6:1 | 47.20036005 | 133.0786401 | 2.819441206 | 1.495409258 | 0.00559826 | 1 | Up |
| lnc-SNAPC5-3:2 | 0 | 15.03877133 | +∞ | +∞ | 0.00029873 | 0.24 | Up |
| lnc-SNURF-1:1 | 26.09598726 | 1.704101286 | 0.065301277 | -3.936744991 | 0.02247455 | 1 | Down |
| lnc-SNURF-1:14 | 33.50172879 | 0.426025321 | 0.012716518 | -6.297152553 | 0.0112398 | 1 | Down |
| lnc-SNURF-1:35 | 10.580866 | 46.71083639 | 4.414651539 | 2.142299566 | 0.00448204 | 1 | Up |
| lnc-SNX24-3:1 | 30.10271872 | 7.64413362 | 0.253934991 | -1.977468887 | 0.04100085 | 1 | Down |
| lnc-SORBS3-2:1 | 47.46563858 | 10.9610635 | 0.230926283 | -2.11449571 | 0.04112168 | 1 | Down |
| lnc-SP6-1:1 | 44.43148425 | 91.50442969 | 2.059450213 | 1.04225925 | 0.04719651 | 1 | Up |
| lnc-SPATA21-2:12 | 14.46278135 | 0.919001169 | 0.063542492 | -3.976134518 | 0.02312117 | 1 | Down |
| lnc-SREK1-7:1 | 44.29071054 | 121.8497745 | 2.751136141 | 1.460027534 | 0.00406164 | 1 | Up |
| lnc-SS18L2-2:8 | 32.37810724 | 8.830936892 | 0.272744074 | -1.874380241 | 0.03292358 | 1 | Down |
| lnc-ST3GAL3-1:1 | 43.83990707 | 11.83743631 | 0.270015087 | -1.888888077 | 0.01803576 | 1 | Down |
| lnc-STAB1-3:1 | 0.208446776 | 8.313638879 | 39.88374895 | 5.31772912 | 0.04315092 | 1 | Up |
| lnc-STAMBPL1-1:6 | 45.57325413 | 7.485910402 | 0.164261046 | -2.605937703 | 0.02750778 | 1 | Down |
| lnc-STK35-3:1 | 0 | 14.05883561 | +∞ | +∞ | 0.02193968 | 1 | Up |
| lnc-STRAP-2:1 | 11.10361039 | 0 | 0 | -∞ | 0.00984641 | 1 | Down |
| lnc-STRN3-1:3 | 8.794638426 | 0 | 0 | -∞ | 0.02392122 | 1 | Down |
| lnc-SUN3-1:1 | 0 | 10.65063304 | +∞ | +∞ | 0.02821406 | 1 | Up |
| lnc-SWI5-1:1 | 8.331666691 | 31.42858512 | 3.77218464 | 1.915400295 | 0.02444247 | 1 | Up |
| lnc-SYAP1-2:1 | 0 | 31.55045423 | +∞ | +∞ | 0.01180389 | 1 | Up |
| lnc-SYNDIG1L-2:3 | 0.390194827 | 20.21200974 | 51.79978915 | 5.69487432 | 0.04482495 | 1 | Up |
| lnc-TACC2-3:3 | 12.7771795 | 51.34847059 | 4.018764126 | 2.006751904 | 0.0053786 | 1 | Up |
| lnc-TACR2-6:1 | 0 | 5.538329178 | +∞ | +∞ | 0.04993031 | 1 | Up |
| lnc-TAF13-2:1 | 40.92314456 | 94.59016986 | 2.311410105 | 1.208773254 | 0.02343195 | 1 | Up |
| lnc-TAGLN-3:1 | 9.742555143 | 0 | 0 | -∞ | 0.01601482 | 1 | Down |
| lnc-TAGLN2-1:1 | 53.3580386 | 109.2395283 | 2.0472928 | 1.033717449 | 0.04558958 | 1 | Up |
| lnc-TAOK3-1:11 | 7.430673772 | 26.83357928 | 3.6111906 | 1.852474568 | 0.03810281 | 1 | Up |
| lnc-TBC1D28-1:1 | 38.57323598 | 6.457330347 | 0.167404424 | -2.578590441 | 0.02382775 | 1 | Down |
| lnc-TBCA-8:1 | 7.366593111 | 31.2886681 | 4.247372921 | 2.086570783 | 0.01542796 | 1 | Up |
| lnc-TBL1Y-6:2 | 33.4262954 | 0 | 0 | -∞ | 0.0097254 | 1 | Down |
| lnc-TC2N-1:2 | 157.2999145 | 74.85282977 | 0.475860588 | -1.071389123 | 0.04132816 | 1 | Down |
| lnc-TFPI-1:4 | 29.65879063 | 6.098255552 | 0.205613763 | -2.281991257 | 0.01939579 | 1 | Down |
| lnc-TH1L-1:3 | 36.31042376 | 8.587456954 | 0.236501149 | -2.0800809 | 0.01959043 | 1 | Down |
| lnc-THAP10-1:1 | 0 | 15.76293689 | +∞ | +∞ | 0.01975143 | 1 | Up |
| lnc-TIAF1-3:1 | 33.17868235 | 6.232156604 | 0.18783617 | -2.412453201 | 0.00973979 | 1 | Down |
| lnc-TLR1-1:1 | 3.640018617 | 20.0414963 | 5.505877417 | 2.46097249 | 0.02179161 | 1 | Up |
| lnc-TM9SF2-8:1 | 3958.54612 | 1969.116898 | 0.497434371 | -1.007421899 | 0.01067061 | 1 | Down |
| lnc-TMCO1-4:1 | 17.71376691 | 0.852050643 | 0.048101042 | -4.377788048 | 0.03464023 | 1 | Down |
| lnc-TMEM120B-4:3 | 7.635184593 | 26.65103389 | 3.49055528 | 1.803456559 | 0.04722259 | 1 | Up |
| lnc-TMEM14C-3:2 | 10.33337845 | 0 | 0 | -∞ | 0.01173859 | 1 | Down |
| lnc-TMEM171-1:1 | 8.717612809 | 0 | 0 | -∞ | 0.03641251 | 1 | Down |
| lnc-TMEM71-3:6 | 0.820437741 | 33.5100674 | 40.84413201 | 5.352056919 | 3.51E-06 | 0.01 | Up |
| lnc-TMEM75-10:1 | 60.58875579 | 13.1824628 | 0.21757276 | -2.200430154 | 0.00289592 | 1 | Down |
| lnc-TMEM75-11:1 | 55.63180589 | 11.18623724 | 0.201076292 | -2.314185105 | 0.00224386 | 0.88 | Down |
| lnc-TMOD3-3:7 | 12.93495961 | 37.8798995 | 2.928489971 | 1.550156954 | 0.04090355 | 1 | Up |
| lnc-TMUB2-1:4 | 10.78866121 | 0 | 0 | -∞ | 0.00929031 | 1 | Down |
| lnc-TNFRSF1A-3:1 | 42.1469059 | 104.5532497 | 2.480686245 | 1.310739276 | 0.02194349 | 1 | Up |
| lnc-TNNT1-1:1 | 87.43843867 | 185.8330717 | 2.125301807 | 1.087667728 | 0.0188612 | 1 | Up |
| lnc-TOR3A-1:1 | 8.19491571 | 27.35087729 | 3.337542234 | 1.738786093 | 0.04044341 | 1 | Up |
| lnc-TPD52L1-1:1 | 0 | 7.39463771 | +∞ | +∞ | 0.04505675 | 1 | Up |
| lnc-TRIM41-1:6 | 50.72961874 | 14.7040187 | 0.289850763 | -1.786617811 | 0.01587316 | 1 | Down |
| lnc-TRIML1-9:1 | 26.76121307 | 0 | 0 | -∞ | 0.00456791 | 1 | Down |
| lnc-TRPM7-1:1 | 10.8520682 | 39.80917453 | 3.668349092 | 1.875130937 | 0.01363659 | 1 | Up |
| lnc-TRUB2-7:1 | 31.05383589 | 80.89642502 | 2.60503808 | 1.381304462 | 0.01681808 | 1 | Up |
| lnc-TSSC4-5:2 | 1.049673796 | 12.64084262 | 12.04263902 | 3.590079673 | 0.02029737 | 1 | Up |
| lnc-TTR-3:1 | 18.07242739 | 51.35448656 | 2.841593189 | 1.506700029 | 0.02488265 | 1 | Up |
| lnc-TULP2-2:1 | 0 | 17.01669069 | +∞ | +∞ | 0.00058622 | 0.36 | Up |
| lnc-TWIST1-1:3 | 1.859738142 | 15.3308956 | 8.243577553 | 3.043270575 | 0.02138726 | 1 | Up |
| lnc-TYSND1-1:7 | 0 | 13.60848812 | +∞ | +∞ | 0.00052708 | 0.34 | Up |
| lnc-UBAC1-2:3 | 0.209934759 | 13.80332373 | 65.7505397 | 6.038930831 | 0.04439824 | 1 | Up |
| lnc-UBL5-5:1 | 1.381600353 | 14.52147332 | 10.51061784 | 3.393775572 | 0.04629349 | 1 | Up |
| lnc-UGCG-1:3 | 0.39127594 | 7.869307363 | 20.11191221 | 4.329978352 | 0.04298069 | 1 | Up |
| lnc-UGCG-2:1 | 33.52031575 | 79.40546553 | 2.368875822 | 1.244202574 | 0.04421033 | 1 | Up |
| lnc-USP9Y-3:1 | 63.06448487 | 0 | 0 | -∞ | 0.00255534 | 0.96 | Down |
| lnc-USP9Y-4:1 | 36.28061912 | 0 | 0 | -∞ | 0.01108782 | 1 | Down |
| lnc-UTP11L-6:1 | 0 | 35.11688002 | +∞ | +∞ | 0.00030174 | 0.24 | Up |
| lnc-UTY-1:53 | 45.8259383 | 0 | 0 | -∞ | 0.02299506 | 1 | Down |
| lnc-VASH2-1:1 | 6.47465934 | 24.16784846 | 3.732682631 | 1.90021285 | 0.03996722 | 1 | Up |
| lnc-VAX1-4:2 | 3.962077606 | 30.32703857 | 7.654327245 | 2.936275582 | 0.0029657 | 1 | Up |
| lnc-VIPR1-4:1 | 0 | 7.242430464 | +∞ | +∞ | 0.03966018 | 1 | Up |
| lnc-VPREB1-7:11 | 57.90383699 | 15.15436619 | 0.261716096 | -1.933925437 | 0.02845276 | 1 | Down |
| lnc-VPS37A-5:1 | 11.07047425 | 0.919001169 | 0.083013713 | -3.590506521 | 0.04782696 | 1 | Down |
| lnc-VSTM5-1:12 | 78.83746146 | 34.6725485 | 0.439797881 | -1.185087441 | 0.04557465 | 1 | Down |
| lnc-VSTM5-1:14 | 490.8148757 | 221.9293709 | 0.452165128 | -1.145078364 | 0.01093225 | 1 | Down |
| lnc-VSTM5-1:9 | 25.91678758 | 1.971903389 | 0.076085949 | -3.71622613 | 0.00664902 | 1 | Down |
| lnc-VTI1A-1:3 | 23.61274429 | 69.59459292 | 2.947331833 | 1.559409499 | 0.01248042 | 1 | Up |
| lnc-WDR38-1:1 | 48.20190762 | 123.9495629 | 2.571465924 | 1.362591036 | 0.00905285 | 1 | Up |
| lnc-WDR7-6:2 | 3.016247357 | 19.81632256 | 6.569859898 | 2.715862606 | 0.01562833 | 1 | Up |
| lnc-WDR73-7:2 | 0 | 9.798582393 | +∞ | +∞ | 0.0303972 | 1 | Up |
| lnc-XPO1-3:1 | 17.76787185 | 2.556151929 | 0.143863708 | -2.7972254 | 0.03468252 | 1 | Down |
| lnc-XPO1-5:1 | 40.62402213 | 108.7342622 | 2.676600112 | 1.420401614 | 0.00610776 | 1 | Up |
| lnc-XRCC1-2:1 | 162.7301138 | 75.7231866 | 0.465329894 | -1.103674223 | 0.03247891 | 1 | Down |
| lnc-YME1L1-1:1 | 9.754409244 | 0.426025321 | 0.043675154 | -4.517043415 | 0.04199157 | 1 | Down |
| lnc-YPEL5-5:1 | 199.9555752 | 3.249979354 | 0.016253507 | -5.943105144 | 6.48E-06 | 0.01 | Down |
| lnc-ZBED5-1:2 | 29.02981841 | 111.1810935 | 3.829892834 | 1.937304024 | 0.04429304 | 1 | Up |
| lnc-ZBTB37-2:1 | 104.2542992 | 44.30663343 | 0.424986152 | -1.234512263 | 0.04414391 | 1 | Down |
| lnc-ZCCHC9-2:5 | 4.867538306 | 26.15805805 | 5.373980933 | 2.425991203 | 0.01384747 | 1 | Up |
| lnc-ZDHHC19-1:3 | 9.276852141 | 0 | 0 | -∞ | 0.0187219 | 1 | Down |
| lnc-ZFAND4-1:1 | 0 | 20.21200974 | +∞ | +∞ | 0.01801677 | 1 | Up |
| lnc-ZFAND5-3:1 | 0 | 22.18391313 | +∞ | +∞ | 0.01650461 | 1 | Up |
| lnc-ZKSCAN3-1:1 | 0 | 7.668455786 | +∞ | +∞ | 0.03773428 | 1 | Up |
| lnc-ZMAT3-4:1 | 62.33804574 | 0 | 0 | -∞ | 0.04029817 | 1 | Down |
| lnc-ZMAT5-4:3 | 1.005429133 | 20.43718348 | 20.32682644 | 4.345313084 | 0.03298775 | 1 | Up |
| lnc-ZNF100-2:2 | 75.83954856 | 30.67382314 | 0.404456827 | -1.305942383 | 0.04905674 | 1 | Down |
| lnc-ZNF100-3:1 | 110.712826 | 25.67109817 | 0.231871041 | -2.108605449 | 0.00644196 | 1 | Down |
| lnc-ZNF114-2:1 | 2.522379845 | 16.09768952 | 6.381945033 | 2.673996183 | 0.04084594 | 1 | Up |
| lnc-ZNF131-10:3 | 11.42755559 | 0.426025321 | 0.037280529 | -4.745433845 | 0.02125521 | 1 | Down |
| lnc-ZNF212-2:2 | 0 | 6.901661863 | +∞ | +∞ | 0.04787287 | 1 | Up |
| lnc-ZNF22-3:2 | 9.408942712 | 0 | 0 | -∞ | 0.04761617 | 1 | Down |
| lnc-ZNF320-2:4 | 11.06944285 | 0 | 0 | -∞ | 0.00868057 | 1 | Down |
| lnc-ZNF587-1:1 | 57.66650893 | 155.9137523 | 2.703714082 | 1.434942595 | 0.00303723 | 1 | Up |
| lnc-ZNF639-2:1 | 17.62420756 | 1.704101286 | 0.096690945 | -3.370475399 | 0.0164038 | 1 | Down |
| lnc-ZNF71-4:1 | 49.02848495 | 8.094481107 | 0.165097517 | -2.598609676 | 0.02440668 | 1 | Down |
| lnc-ZNF737-3:5 | 23.23867977 | 1.971903389 | 0.084854364 | -3.558867333 | 0.00280373 | 1 | Down |
| lnc-ZNF827-11:1 | 0 | 15.33691157 | +∞ | +∞ | 0.02025618 | 1 | Up |
| lnc-ZNF91-4:2 | 0 | 9.749938061 | +∞ | +∞ | 0.00471815 | 1 | Up |
| lnc-ZSCAN10-3:17 | 12.90033499 | 63.47803117 | 4.920649831 | 2.298848853 | 0.0005682 | 0.36 | Up |
| lnc-ZSWIM7-3:1 | 34.21005771 | 79.77657227 | 2.331962517 | 1.221544599 | 0.02989745 | 1 | Up |
| lnc-ZZZ3-5:1 | 9.444365174 | 0 | 0 | -∞ | 0.03746816 | 1 | Down |

**Note:** QDC: qi deficiency constitution; BC: balanced constituion.

^a^ normalized mean count value;

^b^ normalized mean count value (QDC) / normalized mean count value (BC);

^c^ *P*-value adjusted by false discovery rate (FDR).

**Table S6** Differential lncRNAs identified in the PQDS population compared with the BC control population

| **lncRNAs** | **Base Mean ^a^**  **(BC, n=5)** | **Base Mean ^a^**  **(PQDS of CSG, n=5)** | **Fold Change ^b^** | **log_2_ (FoldChange)** | ***P*-value** | **padj ^c^** | **up_down** |
| --- | --- | --- | --- | --- | --- | --- | --- |
| BHLHE40-AS1:2 | 1.037274669 | 7.005241082 | 6.753506367 | 2.755636732 | 0.0496624 | 1 | Up |
| CCDC18-AS1:11 | 46.81320972 | 2.941805175 | 0.062841347 | -3.992142076 | 0.0325319 | 1 | Down |
| CCDC18-AS1:25 | 0 | 7.338295544 | +∞ | +∞ | 0.0023325 | 1 | Up |
| CCDC18-AS1:37 | 0 | 3.993114159 | +∞ | +∞ | 0.0222493 | 1 | Up |
| CCDC18-AS1:9 | 20.7005934 | 3.820437986 | 0.184556931 | -2.437862177 | 0.0298468 | 1 | Down |
| CDC42-IT1:1 | 21.60976453 | 7.389738454 | 0.341962933 | -1.548088143 | 0.0449413 | 1 | Down |
| DLEU2:13 | 14.66806271 | 57.86019181 | 3.944637607 | 1.979892766 | 0.014505 | 1 | Up |
| DLX6-AS1:13 | 0 | 3.762571797 | +∞ | +∞ | 0.0272182 | 1 | Up |
| DNAJC27-AS1:8 | 21.66661229 | 47.1928352 | 2.178136322 | 1.12309425 | 0.0274393 | 1 | Up |
| EDNRB-AS1:2 | 0 | 3.823343422 | +∞ | +∞ | 0.027829 | 1 | Up |
| EIF1B-AS1:4 | 12.79144875 | 3.806087714 | 0.297549385 | -1.748798958 | 0.0341015 | 1 | Down |
| EML2-AS1:2 | 12.84258352 | 3.506107013 | 0.273006363 | -1.87299352 | 0.0289858 | 1 | Down |
| EMX2OS:2 | 4.501993827 | 16.50734387 | 3.666674034 | 1.874472017 | 0.0464379 | 1 | Up |
| GAS5:13 | 4.593060206 | 31.19903183 | 6.792645955 | 2.76397366 | 0.0019973 | 1 | Up |
| GAS5:31 | 47.25877965 | 15.31152354 | 0.323993206 | -1.625964533 | 0.0033319 | 1 | Down |
| GAS5:41 | 131.7172496 | 58.35739789 | 0.443050535 | -1.174456831 | 0.0037402 | 1 | Down |
| HCG21:1 | 22.48749477 | 45.55160465 | 2.02564159 | 1.018378931 | 0.044351 | 1 | Up |
| ILF3-AS1:5 | 0.19314855 | 4.876753492 | 25.24871919 | 4.6581383 | 0.0329059 | 1 | Up |
| INHBA-AS1:5 | 0 | 4.623786564 | +∞ | +∞ | 0.0339676 | 1 | Up |
| IQCH-AS1:1 | 2.49894532 | 11.2978983 | 4.52106663 | 2.17666318 | 0.0304928 | 1 | Up |
| IQCH-AS1:6 | 32.58606866 | 9.979498292 | 0.306250453 | -1.707216116 | 0.0028845 | 1 | Down |
| ITPKB-IT1:3 | 0 | 9.079182918 | +∞ | +∞ | 0.034523 | 1 | Up |
| KANSL1-AS1:1 | 17.34558837 | 6.588491404 | 0.379836721 | -1.39654871 | 0.0455628 | 1 | Down |
| LINC00092:1 | 0.58683418 | 6.283738088 | 10.70785974 | 3.420598241 | 0.0303525 | 1 | Up |
| LINC00152:10 | 20.17792838 | 2.263025856 | 0.112153528 | -3.156453091 | 0.0007348 | 1 | Down |
| LINC00152:12 | 26.52604435 | 7.326443721 | 0.276198125 | -1.856224568 | 0.0278032 | 1 | Down |
| LINC00200:1 | 8.211027724 | 1.297261744 | 0.15799018 | -2.66209321 | 0.0357957 | 1 | Down |
| LINC00265:7 | 0.19314855 | 7.497764771 | 38.81864391 | 5.278677815 | 0.0314414 | 1 | Up |
| LINC00299:3 | 0 | 4.822074517 | +∞ | +∞ | 0.0321805 | 1 | Up |
| LINC00299:5 | 3.052341743 | 11.94174673 | 3.912322976 | 1.968025475 | 0.0414083 | 1 | Up |
| LINC00311:2 | 2.058852737 | 9.759670731 | 4.740344249 | 2.244991833 | 0.0336841 | 1 | Up |
| LINC00426:19 | 0 | 5.250784189 | +∞ | +∞ | 0.0375824 | 1 | Up |
| LINC00485:1 | 1.609712301 | 10.72201615 | 6.660827615 | 2.735701445 | 0.0119662 | 1 | Up |
| LINC00507:7 | 0 | 6.534261108 | +∞ | +∞ | 0.0394864 | 1 | Up |
| LINC00547:1 | 29.14938795 | 10.05138378 | 0.34482315 | -1.53607146 | 0.0374801 | 1 | Down |
| LINC00857:1 | 0 | 3.480271139 | +∞ | +∞ | 0.0440088 | 1 | Up |
| LINC00869:36 | 4.723647883 | 15.94372945 | 3.375300159 | 1.755015804 | 0.0384516 | 1 | Up |
| LINC00869:40 | 0 | 16.09635819 | +∞ | +∞ | 0.0225378 | 1 | Up |
| LINC00926:14 | 1.28556447 | 10.61386478 | 8.2561902 | 3.045476207 | 0.034726 | 1 | Up |
| LINC00944:13 | 4.63730303 | 24.27217083 | 5.234113594 | 2.387945235 | 0.001254 | 1 | Up |
| LINC00957:3 | 8.108032151 | 29.49245098 | 3.637436364 | 1.862922008 | 0.0108226 | 1 | Up |
| LINC00958:13 | 17.96929341 | 1.368925052 | 0.076181351 | -3.714418313 | 0.0277225 | 1 | Down |
| LINC00963:35 | 19.57916928 | 3.497753206 | 0.17864666 | -2.48481915 | 0.0116167 | 1 | Down |
| LINC00963:36 | 7.639332786 | 0 | 0 | -∞ | 0.0169963 | 1 | Down |
| LINC00969:160 | 0 | 16.56052538 | +∞ | +∞ | 0.048343 | 1 | Up |
| LINC01011:8 | 0.414864785 | 6.678972915 | 16.09915604 | 4.008913155 | 0.0117968 | 1 | Up |
| LINC01032:1 | 8.697334447 | 28.86196892 | 3.318484427 | 1.730524504 | 0.005945 | 1 | Up |
| LINC01032:4 | 15.50880936 | 33.30142795 | 2.147258837 | 1.102496109 | 0.0463245 | 1 | Up |
| LINC01088:13 | 0.201343884 | 8.982812144 | 44.61427861 | 5.479433607 | 0.0007186 | 1 | Up |
| LINC01089:2 | 20.21258961 | 6.663007955 | 0.329646428 | -1.601008648 | 0.0228714 | 1 | Down |
| LINC01102:7 | 2.81311442 | 14.81886478 | 5.267778899 | 2.397194794 | 0.0085068 | 1 | Up |
| LINC01125:38 | 5.013089273 | 0.151197822 | 0.030160608 | -5.051190665 | 0.0470064 | 1 | Down |
| LINC01146:2 | 25.79458822 | 7.615452436 | 0.295234503 | -1.76006676 | 0.0474258 | 1 | Down |
| LINC01237:6 | 20.12043734 | 43.87886157 | 2.180810528 | 1.124864432 | 0.0367068 | 1 | Up |
| LINC01285:5 | 21.03751757 | 53.30639801 | 2.533873011 | 1.341344224 | 0.0072837 | 1 | Up |
| LINC01347:6 | 26.88713064 | 0.453593465 | 0.016870282 | -5.889372134 | 0.0024084 | 1 | Down |
| LINC01422:2 | 8.711871473 | 1.537594389 | 0.176494154 | -2.502307694 | 0.0407431 | 1 | Down |
| LINC01506:2 | 2.005370807 | 23.83898528 | 11.88756972 | 3.571381897 | 0.0005619 | 0.9351433 | Up |
| LINC01513:1 | 0 | 3.555774292 | +∞ | +∞ | 0.0394323 | 1 | Up |
| LINC01578:6 | 26.23705259 | 60.90222968 | 2.321229852 | 1.214889388 | 0.0298545 | 1 | Up |
| MAPKAPK5-AS1:15 | 37.72529392 | 14.18889322 | 0.376110873 | -1.410770083 | 0.0345979 | 1 | Down |
| MEG3:41 | 2.794228375 | 11.23634054 | 4.021267783 | 2.007650411 | 0.0383135 | 1 | Up |
| MIR4435-2HG:11 | 0.201343884 | 8.979660297 | 44.59862455 | 5.478927312 | 0.0102221 | 1 | Up |
| MIR663AHG:14 | 17.78079081 | 79.98777845 | 4.498550111 | 2.169460094 | 0.0475996 | 1 | Up |
| MIR663AHG:17 | 76.05100371 | 165.2228566 | 2.172526969 | 1.119374086 | 0.0446989 | 1 | Up |
| MIR663AHG:28 | 20.62405242 | 52.97522756 | 2.568613892 | 1.360990044 | 0.0148785 | 1 | Up |
| MIR663AHG:9 | 1579.140515 | 4261.739475 | 2.698771537 | 1.432302852 | 0.0296539 | 1 | Up |
| MIR9-3HG:31 | 13.13828296 | 0.973279521 | 0.074079659 | -3.754778732 | 0.0007586 | 1 | Down |
| MMP25-AS1:25 | 2.376890845 | 10.3231983 | 4.343152032 | 2.118742455 | 0.0370118 | 1 | Up |
| NIFK-AS1:14 | 1.229929969 | 7.82087648 | 6.358798203 | 2.668754126 | 0.0396823 | 1 | Up |
| NIFK-AS1:5 | 50.85193882 | 13.37466259 | 0.263011852 | -1.926800284 | 0.0005829 | 0.9351433 | Down |
| NIFK-AS1:9 | 17.87735945 | 4.497636815 | 0.251582837 | -1.990894588 | 0.0366616 | 1 | Down |
| OSER1-AS1:15 | 4.889605605 | 18.65687934 | 3.815620492 | 1.931917686 | 0.0155646 | 1 | Up |
| P3H2-AS1:2 | 39.40714621 | 95.48283703 | 2.422982789 | 1.276784157 | 0.0379418 | 1 | Up |
| PAN3-AS1:5 | 27.86578308 | 0 | 0 | -∞ | 1.00E-08 | 0.0001332 | Down |
| PCAT7:5 | 0.77998273 | 6.857069288 | 8.791309123 | 3.136078015 | 0.0282918 | 1 | Up |
| PCBP1-AS1:199 | 13.72604612 | 1.430700628 | 0.104232538 | -3.26212238 | 0.0212314 | 1 | Down |
| PITPNA-AS1:2 | 33.06351701 | 12.05538184 | 0.36461281 | -1.455562847 | 0.008596 | 1 | Down |
| PRKAR2A-AS1:8 | 19.57715347 | 40.33555226 | 2.06033795 | 1.042880997 | 0.0474707 | 1 | Up |
| PRKCQ-AS1:28 | 2.209135102 | 15.14951617 | 6.857668488 | 2.777718164 | 0.0132799 | 1 | Up |
| PSMA3-AS1:19 | 4.455546158 | 25.83322977 | 5.797993973 | 2.535553834 | 0.0177399 | 1 | Up |
| PSMA3-AS1:6 | 39.57978456 | 18.5265061 | 0.468080014 | -1.095172929 | 0.0405807 | 1 | Down |
| PSMD5-AS1:23 | 17.94968507 | 0.957884202 | 0.053364959 | -4.227963461 | 0.0195102 | 1 | Down |
| PSMD5-AS1:3 | 32.61301348 | 3.83025266 | 0.11744553 | -3.089936287 | 1.64E-05 | 0.1306777 | Down |
| RBFADN:7 | 3.597377822 | 0 | 0 | -∞ | 0.0235972 | 1 | Down |
| RPARP-AS1:17 | 1.28556447 | 8.651236186 | 6.7295234 | 2.750504333 | 0.0219694 | 1 | Up |
| SIRPG-AS1:1 | 6.462226645 | 20.06377387 | 3.104777188 | 1.634489738 | 0.0207265 | 1 | Up |
| SNHG11:14 | 1.220008353 | 9.530398839 | 7.811748843 | 2.965645566 | 0.0313796 | 1 | Up |
| SNHG15:2 | 0 | 8.620709041 | +∞ | +∞ | 0.0030518 | 1 | Up |
| SNHG17:19 | 0 | 8.184081754 | +∞ | +∞ | 0.0132755 | 1 | Up |
| SNHG23:12 | 0 | 6.126071709 | +∞ | +∞ | 0.0069281 | 1 | Up |
| ST8SIA6-AS1:3 | 0.427041803 | 5.742854766 | 13.44799205 | 3.749318872 | 0.0217051 | 1 | Up |
| STAM-AS1:1 | 1.461290137 | 8.558824723 | 5.857033115 | 2.550170051 | 0.0366257 | 1 | Up |
| STAU2-AS1:2 | 4.518722363 | 26.98010562 | 5.970737621 | 2.577909172 | 0.000805 | 1 | Up |
| TCL6:19 | 2.607788853 | 13.24596869 | 5.079386959 | 2.344654386 | 0.0449926 | 1 | Up |
| TCONS_00000205 | 94.19990984 | 43.93331196 | 0.466383801 | -1.100410416 | 0.0399465 | 1 | Down |
| TCONS_00001685 | 8.208636503 | 23.35030954 | 2.844602698 | 1.508227167 | 0.038281 | 1 | Up |
| TCONS_00003874 | 102.6565191 | 5.194010859 | 0.050596016 | -4.304832406 | 0.0042371 | 1 | Down |
| TCONS_00003879 | 260.9050137 | 113.3731614 | 0.434538071 | -1.202445514 | 0.0353018 | 1 | Down |
| TCONS_00007460 | 50.79997923 | 19.55901239 | 0.385020086 | -1.376994382 | 0.008274 | 1 | Down |
| TCONS_00007461 | 41.78672753 | 13.16985316 | 0.315168331 | -1.665805521 | 0.0197683 | 1 | Down |
| TCONS_00022458 | 16.31186861 | 0.914736713 | 0.056077984 | -4.156421694 | 0.0148346 | 1 | Down |
| TCONS_00022592 | 11.8256292 | 2.338033433 | 0.197709009 | -2.338549481 | 0.0478182 | 1 | Down |
| TCONS_00026722 | 7.288004124 | 0 | 0 | -∞ | 0.0311965 | 1 | Down |
| TCONS_00031767 | 131.1722869 | 49.31989014 | 0.375993217 | -1.411221459 | 0.0290473 | 1 | Down |
| TCONS_00040834 | 261.0777582 | 66.10559772 | 0.253202717 | -1.98163521 | 0.0382309 | 1 | Down |
| TCONS_00048607 | 0 | 10.10493746 | +∞ | +∞ | 0.0369686 | 1 | Up |
| TCONS_00048609 | 41.33782644 | 326.1887108 | 7.890804595 | 2.980172414 | 0.0137389 | 1 | Up |
| TCONS_00048613 | 136.9604429 | 367.515184 | 2.683367374 | 1.424044584 | 0.0274695 | 1 | Up |
| TCONS_00048617 | 22.52240204 | 85.2896948 | 3.786882707 | 1.921010737 | 0.0472886 | 1 | Up |
| TCONS_00048632 | 210.6002157 | 752.2809404 | 3.57208058 | 1.836764625 | 0.0307215 | 1 | Up |
| TCONS_00048664 | 14.38714303 | 272.9175509 | 18.96954457 | 4.245613137 | 0.0037076 | 1 | Up |
| TCONS_00048671 | 0 | 146.6407069 | +∞ | +∞ | 0.0165892 | 1 | Up |
| TCONS_00048674 | 53.78799439 | 333.1494708 | 6.193751498 | 2.630813501 | 0.0314736 | 1 | Up |
| TCONS_00048675 | 68.13192217 | 283.8447534 | 4.166105173 | 2.058699261 | 0.0375068 | 1 | Up |
| TCONS_00048677 | 518.9790505 | 2492.674158 | 4.803034257 | 2.263946099 | 0.0145238 | 1 | Up |
| TCONS_00048679 | 17727.02243 | 52883.95172 | 2.983239398 | 1.576879755 | 0.0102858 | 1 | Up |
| TCONS_00049079 | 729.1742965 | 1620.275179 | 2.222068424 | 1.151903242 | 0.032218 | 1 | Up |
| TCONS_00049080 | 0.401074161 | 21.14504156 | 52.72102666 | 5.72030656 | 0.0091728 | 1 | Up |
| TCONS_00049093 | 23.80086221 | 144.3169612 | 6.063518201 | 2.600155124 | 0.0004678 | 0.9351433 | Up |
| TCONS_00049094 | 250.9266413 | 712.2209736 | 2.838363316 | 1.505059269 | 0.0057691 | 1 | Up |
| TCONS_00049098 | 244.252186 | 778.9607241 | 3.189165824 | 1.673179114 | 0.0002258 | 0.5989887 | Up |
| TCONS_00049101 | 224.6323743 | 575.6055517 | 2.562433636 | 1.357514641 | 0.0427115 | 1 | Up |
| TCONS_00049102 | 947.5833575 | 2107.509064 | 2.224088306 | 1.153214071 | 0.0014438 | 1 | Up |
| TCONS_00049105 | 16.61862342 | 61.75700495 | 3.716132401 | 1.893801904 | 0.0425936 | 1 | Up |
| TCONS_00049107 | 87.58561491 | 476.4985473 | 5.440374516 | 2.44370597 | 0.0017286 | 1 | Up |
| TCONS_00049108 | 922.918586 | 2359.771936 | 2.55685818 | 1.354372142 | 0.024221 | 1 | Up |
| TCONS_00049110 | 393.1657168 | 801.3590401 | 2.038222067 | 1.027311244 | 0.022666 | 1 | Up |
| TCONS_00052318 | 0 | 49.44319449 | +∞ | +∞ | 7.71E-05 | 0.3067612 | Up |
| TCONS_00052321 | 328.4260299 | 783.9580118 | 2.387015463 | 1.255207912 | 0.0127006 | 1 | Up |
| TCONS_00052324 | 34.54052641 | 141.1229962 | 4.08572222 | 2.030591121 | 0.0428306 | 1 | Up |
| TCONS_00052331 | 223.7124377 | 1571.687489 | 7.025481039 | 2.812597011 | 0.0010505 | 1 | Up |
| TCONS_00052862 | 0 | 798.0724827 | +∞ | +∞ | 0.0181352 | 1 | Up |
| TCONS_00052867 | 423.361433 | 1639.814612 | 3.873320723 | 1.953570966 | 0.0009054 | 1 | Up |
| TCONS_00052868 | 0 | 30.92335945 | +∞ | +∞ | 0.0010752 | 1 | Up |
| TCONS_00052878 | 808.9862038 | 1848.121025 | 2.284490163 | 1.19187223 | 0.0493657 | 1 | Up |
| TCONS_00052892 | 29.68415761 | 59.74556595 | 2.012708824 | 1.009138474 | 0.0480642 | 1 | Up |
| THUMPD3-AS1:11 | 0.42852149 | 6.191477672 | 14.44846481 | 3.852844306 | 0.020541 | 1 | Up |
| THUMPD3-AS1:23 | 0 | 12.49263242 | +∞ | +∞ | 0.0343863 | 1 | Up |
| TMEM161B-AS1:24 | 0 | 5.737968939 | +∞ | +∞ | 0.0276545 | 1 | Up |
| TMEM161B-AS1:31 | 5.581713171 | 0 | 0 | -∞ | 0.0306015 | 1 | Down |
| TMEM44-AS1:4 | 18.12193412 | 4.432871726 | 0.24461361 | -2.031423419 | 0.0314673 | 1 | Down |
| TMEM9B-AS1:2 | 11.47940065 | 31.49789767 | 2.743862562 | 1.45620822 | 0.0306023 | 1 | Up |
| TP53TG1:7 | 6.472375383 | 0.302541517 | 0.046743506 | -4.419090243 | 0.008918 | 1 | Down |
| WEE2-AS1:6 | 3.618542291 | 0 | 0 | -∞ | 0.0328292 | 1 | Down |
| ZEB1-AS1:13 | 0 | 9.144910391 | +∞ | +∞ | 0.0079432 | 1 | Up |
| ZNF528-AS1:12 | 2.775771717 | 22.88707159 | 8.245300378 | 3.043572052 | 0.0179583 | 1 | Up |
| lnc-A1BG-1:4 | 0 | 4.938013347 | +∞ | +∞ | 0.0453818 | 1 | Up |
| lnc-AAMP-1:2 | 5.997262307 | 20.47749469 | 3.414473744 | 1.77166324 | 0.017388 | 1 | Up |
| lnc-AC006305.1-2:2 | 27.21767082 | 9.38008672 | 0.344632235 | -1.536870446 | 0.0178537 | 1 | Down |
| lnc-AC007390.5.1-1:1 | 0.42852149 | 6.170587515 | 14.39971543 | 3.847968396 | 0.0171429 | 1 | Up |
| lnc-AC007952.1-1:1 | 9.201374538 | 46.91884305 | 5.099112405 | 2.350246141 | 0.0009007 | 1 | Up |
| lnc-AC009958.1-3:1 | 148.7814628 | 62.95801682 | 0.423157668 | -1.240732786 | 0.0053938 | 1 | Down |
| lnc-AC010336.2-4:2 | 2.013438842 | 10.47171048 | 5.200908151 | 2.37876356 | 0.0254932 | 1 | Up |
| lnc-AC012652.1.1-4:1 | 16.52312132 | 37.69738988 | 2.281493257 | 1.18997839 | 0.0290155 | 1 | Up |
| lnc-AC073043.2.1-1:4 | 4.107439213 | 0 | 0 | -∞ | 0.030854 | 1 | Down |
| lnc-AC091801.1.1-5:1 | 36.51599874 | 81.62378599 | 2.235288334 | 1.16046094 | 0.0096848 | 1 | Up |
| lnc-AC092329.1-1:2 | 0 | 17.56508355 | +∞ | +∞ | 0.0148124 | 1 | Up |
| lnc-AC092329.1-1:3 | 0 | 7.294380985 | +∞ | +∞ | 0.0387476 | 1 | Up |
| lnc-AC103810.1-1:2 | 0 | 8.580500928 | +∞ | +∞ | 0.0298148 | 1 | Up |
| lnc-AC104024.2.1-3:1 | 14.17308837 | 1.898956692 | 0.133983268 | -2.899875253 | 0.0048189 | 1 | Down |
| lnc-AC106873.4.1-6:2 | 3.01696036 | 21.06550127 | 6.982359315 | 2.8037146 | 0.0004875 | 0.9351433 | Up |
| lnc-AC115989.1.1-5:1 | 7.698005117 | 22.33497297 | 2.901397522 | 1.536747973 | 0.04416 | 1 | Up |
| lnc-AC130352.1-2:15 | 9.447913315 | 37.63099634 | 3.982995513 | 1.993853855 | 0.0084475 | 1 | Up |
| lnc-AC131097.4.1-5:2 | 20.83272492 | 3.505931861 | 0.168289644 | -2.570981694 | 0.0334226 | 1 | Down |
| lnc-AC131097.4.1-8:1 | 0.19314855 | 9.675743954 | 50.094831 | 5.646589843 | 0.0166949 | 1 | Up |
| lnc-AC233264.1-1:2 | 23.35492799 | 4.712738185 | 0.201787742 | -2.309089557 | 0.0042634 | 1 | Down |
| lnc-AC233264.2-1:1 | 171.8563834 | 79.39649505 | 0.461993285 | -1.114056214 | 0.0024069 | 1 | Down |
| lnc-ACRC-3:2 | 38.461685 | 17.07216002 | 0.443874469 | -1.171776364 | 0.0439431 | 1 | Down |
| lnc-ACTR2-8:1 | 0 | 5.130484701 | +∞ | +∞ | 0.0065819 | 1 | Up |
| lnc-ADIPOR2-1:2 | 1.255157767 | 8.220383395 | 6.549282977 | 2.711336967 | 0.0235324 | 1 | Up |
| lnc-AF131215.2.1-1:2 | 8.708655666 | 39.19192349 | 4.500341384 | 2.170034445 | 0.015331 | 1 | Up |
| lnc-AGA-4:2 | 43.70043108 | 9.407116573 | 0.215263702 | -2.215823024 | 0.0001524 | 0.4332732 | Down |
| lnc-AGBL1-7:1 | 0.386297099 | 5.763638453 | 14.92022193 | 3.89919709 | 0.023023 | 1 | Up |
| lnc-AGPAT2-2:4 | 25.21704873 | 63.18474514 | 2.505636002 | 1.325176847 | 0.0285339 | 1 | Up |
| lnc-AKAP11-1:2 | 2.41612661 | 31.89012356 | 13.19886277 | 3.722341725 | 0.012002 | 1 | Up |
| lnc-AKR1C2-10:1 | 25.61977084 | 54.28257779 | 2.118776867 | 1.083231663 | 0.0297508 | 1 | Up |
| lnc-AKT2-2:1 | 9.9456997 | 24.75660493 | 2.489176797 | 1.315668703 | 0.0386527 | 1 | Up |
| lnc-AL033381.1-6:3 | 6.994156541 | 25.15788836 | 3.596986743 | 1.846788842 | 0.0237903 | 1 | Up |
| lnc-AL136218.1-1:2 | 7.116960487 | 26.6975613 | 3.75125889 | 1.907374833 | 0.0041031 | 1 | Up |
| lnc-AL669831.1-3:33 | 17.43294861 | 1.0224137 | 0.058648352 | -4.09176563 | 0.0046151 | 1 | Down |
| lnc-AL901608.1-3:20 | 32.24251674 | 6.196959376 | 0.192198377 | -2.379331944 | 0.0044916 | 1 | Down |
| lnc-AL901608.1-3:39 | 561.6234282 | 228.0345567 | 0.4060275 | -1.30035065 | 0.0258404 | 1 | Down |
| lnc-ALG10B-8:1 | 9.745399497 | 36.32302269 | 3.727196889 | 1.898091031 | 0.0412498 | 1 | Up |
| lnc-ALK-1:1 | 145.9000872 | 294.1142679 | 2.01586081 | 1.011396028 | 0.047418 | 1 | Up |
| lnc-AMZ1-7:1 | 15.94617126 | 5.05150936 | 0.316785094 | -1.658423643 | 0.0292256 | 1 | Down |
| lnc-AMZ2-5:1 | 43.92853966 | 92.13782611 | 2.097447965 | 1.06863502 | 0.0129181 | 1 | Up |
| lnc-ANGEL1-1:7 | 6.608002988 | 18.96699349 | 2.870306434 | 1.521204767 | 0.0439706 | 1 | Up |
| lnc-ANKRD18A-3:1 | 3.105278088 | 0 | 0 | -∞ | 0.0427699 | 1 | Down |
| lnc-ANKRD30BL-3:1 | 243.0025865 | 725.4591827 | 2.985396959 | 1.577922775 | 0.0184741 | 1 | Up |
| lnc-ANKRD65-4:2 | 15.23742337 | 4.556469004 | 0.299031463 | -1.741630807 | 0.0471995 | 1 | Down |
| lnc-AP000525.1-4:1 | 17.738547 | 4.599387939 | 0.25928775 | -1.947374047 | 0.0111257 | 1 | Down |
| lnc-AP001793.1-4:1 | 20.10097311 | 4.141656614 | 0.206042593 | -2.278985496 | 0.0048514 | 1 | Down |
| lnc-AP001793.1-7:1 | 1.470158356 | 8.216149804 | 5.58861552 | 2.482490925 | 0.0395652 | 1 | Up |
| lnc-APBA2-4:1 | 0 | 4.569149949 | +∞ | +∞ | 0.0110412 | 1 | Up |
| lnc-ARAF-1:1 | 0.42852149 | 75.80694594 | 176.9034872 | 7.466818677 | 8.04E-10 | 1.60E-05 | Up |
| lnc-ARHGAP27-2:1 | 10.96471872 | 0.735570799 | 0.067085241 | -3.897860785 | 0.0013884 | 1 | Down |
| lnc-ARHGEF3-3:2 | 52.81925447 | 8.765587456 | 0.165954396 | -2.591141252 | 0.0037396 | 1 | Down |
| lnc-ARL1-3:1 | 19.47460259 | 46.7829038 | 2.402252041 | 1.264387524 | 0.0323723 | 1 | Up |
| lnc-ARL10-3:2 | 21.50121338 | 43.35628521 | 2.016457604 | 1.011823073 | 0.0494733 | 1 | Up |
| lnc-ARVCF-1:9 | 23.36030965 | 55.05469277 | 2.356762115 | 1.236806144 | 0.0134918 | 1 | Up |
| lnc-ASAP1-5:1 | 1.216699554 | 9.514303536 | 7.819764133 | 2.967125092 | 0.015079 | 1 | Up |
| lnc-ATAD5-3:12 | 10.10078359 | 0.604791287 | 0.05987568 | -4.061886051 | 0.0493 | 1 | Down |
| lnc-ATHL1-1:6 | 14.51948332 | 1.505246604 | 0.10367081 | -3.269918348 | 0.040667 | 1 | Down |
| lnc-ATL3-1:9 | 8.273342002 | 0 | 0 | -∞ | 0.0108898 | 1 | Down |
| lnc-AUH-3:1 | 19.92767408 | 44.06776424 | 2.211385235 | 1.144950372 | 0.0287564 | 1 | Up |
| lnc-AXDND1-1:1 | 8.480355923 | 22.30448817 | 2.630135855 | 1.395137321 | 0.0377532 | 1 | Up |
| lnc-BACE2-1:1 | 0 | 3.873021798 | +∞ | +∞ | 0.0226634 | 1 | Up |
| lnc-BAG1-2:12 | 0.200537081 | 7.795829921 | 38.87475519 | 5.280761685 | 0.0422472 | 1 | Up |
| lnc-BAG1-2:9 | 0.642782235 | 10.35834081 | 16.11485234 | 4.010319064 | 0.0013663 | 1 | Up |
| lnc-BCL11A-6:1 | 4.895231924 | 0 | 0 | -∞ | 0.0448675 | 1 | Down |
| lnc-BCL11B-1:4 | 0.415604629 | 9.170389851 | 22.06517735 | 4.463699438 | 0.0428946 | 1 | Up |
| lnc-BCL2L2-PABPN1-1:1 | 88.06395334 | 42.09415512 | 0.477995292 | -1.064931685 | 0.0194252 | 1 | Down |
| lnc-BHLHA15-2:1 | 39.47646721 | 9.758917067 | 0.24720847 | -2.016199919 | 0.0298453 | 1 | Down |
| lnc-BRD1-6:1 | 24.30636387 | 9.093225496 | 0.374108836 | -1.418470054 | 0.0377142 | 1 | Down |
| lnc-BRD1-7:1 | 46.13171653 | 10.96128639 | 0.237608466 | -2.073341855 | 0.0024539 | 1 | Down |
| lnc-BTK-1:2 | 23.52175111 | 5.224863791 | 0.222129031 | -2.170530135 | 0.001165 | 1 | Down |
| lnc-BX284668.1-1:1 | 18.35467571 | 2.497350761 | 0.13606074 | -2.877677253 | 0.0074977 | 1 | Down |
| lnc-C10orf12-1:2 | 9.745113965 | 0 | 0 | -∞ | 0.003628 | 1 | Down |
| lnc-C10orf131-1:1 | 16.15480734 | 5.284746569 | 0.327131513 | -1.612057352 | 0.0461349 | 1 | Down |
| lnc-C12orf42-2:9 | 3.277211614 | 0 | 0 | -∞ | 0.0480774 | 1 | Down |
| lnc-C12orf44-4:4 | 28.41144685 | 11.28165336 | 0.397081269 | -1.332493788 | 0.0313012 | 1 | Down |
| lnc-C12orf50-6:2 | 1.640499518 | 12.15634217 | 7.410146749 | 2.889502114 | 0.0043116 | 1 | Up |
| lnc-C14orf149-1:5 | 5.615655863 | 17.92026799 | 3.191126456 | 1.67406578 | 0.0327848 | 1 | Up |
| lnc-C16orf5-1:1 | 1.568160794 | 12.26346494 | 7.820285384 | 2.967221256 | 0.0213143 | 1 | Up |
| lnc-C17orf77-1:1 | 43.44833775 | 18.72756017 | 0.431030533 | -1.214138026 | 0.0169201 | 1 | Down |
| lnc-C17orf77-1:5 | 35.72931477 | 15.36059327 | 0.429915697 | -1.217874307 | 0.0265038 | 1 | Down |
| lnc-C19orf69-2:2 | 3.771078496 | 15.0683741 | 3.995773123 | 1.99847467 | 0.0301659 | 1 | Up |
| lnc-C1orf132-1:7 | 21.1471026 | 45.81802018 | 2.166633465 | 1.11545511 | 0.0299821 | 1 | Up |
| lnc-C1orf98-2:1 | 0 | 5.972809708 | +∞ | +∞ | 0.0140465 | 1 | Up |
| lnc-C20orf24-2:1 | 4.72241001 | 0 | 0 | -∞ | 0.007414 | 1 | Down |
| lnc-C20orf29-1:5 | 0.772594199 | 9.254955578 | 11.97906429 | 3.582443316 | 0.0084904 | 1 | Up |
| lnc-C22orf43-2:3 | 17.41640289 | 0.61344822 | 0.035222441 | -4.827361303 | 0.0191373 | 1 | Down |
| lnc-C22orf46-2:1 | 0.427041803 | 5.488304533 | 12.85191403 | 3.68391133 | 0.0269053 | 1 | Up |
| lnc-C2orf54-1:1 | 59.31006967 | 20.91083442 | 0.35256803 | -1.504026434 | 0.0041079 | 1 | Down |
| lnc-C2orf55-1:7 | 12.15166321 | 28.16403802 | 2.31771055 | 1.212700405 | 0.0491487 | 1 | Up |
| lnc-C2orf89-9:1 | 12.25158318 | 3.211185579 | 0.262103724 | -1.931790242 | 0.0456276 | 1 | Down |
| lnc-C5orf13-6:1 | 3.253587577 | 0 | 0 | -∞ | 0.035379 | 1 | Down |
| lnc-C5orf49-2:1 | 4.009611691 | 14.61302324 | 3.644498361 | 1.865720252 | 0.0203277 | 1 | Up |
| lnc-C6orf120-3:1 | 63.95287041 | 135.7842212 | 2.123191975 | 1.086234823 | 0.0070755 | 1 | Up |
| lnc-C6orf201-2:2 | 8.726581575 | 27.26730636 | 3.124626306 | 1.643683659 | 0.0178469 | 1 | Up |
| lnc-C7orf41-3:1 | 0 | 8.032206341 | +∞ | +∞ | 0.0244441 | 1 | Up |
| lnc-C7orf55-1:2 | 1.996241369 | 9.874215728 | 4.946403716 | 2.306379995 | 0.0289565 | 1 | Up |
| lnc-C9orf107-1:1 | 3.972330889 | 25.04089734 | 6.303829676 | 2.656228555 | 0.0081873 | 1 | Up |
| lnc-C9orf146-7:1 | 26.80777513 | 59.69971159 | 2.226955101 | 1.155072472 | 0.015864 | 1 | Up |
| lnc-C9orf82-2:1 | 41.14996514 | 13.61192903 | 0.330788349 | -1.596019674 | 0.0030867 | 1 | Down |
| lnc-CA10-1:1 | 2.228521015 | 11.38701804 | 5.109674962 | 2.353231521 | 0.0172033 | 1 | Up |
| lnc-CACYBP-2:1 | 8.04651198 | 26.71502813 | 3.320075605 | 1.731216095 | 0.0278642 | 1 | Up |
| lnc-CARD11-1:1 | 1.232417339 | 7.488974144 | 6.076654316 | 2.603277223 | 0.0389 | 1 | Up |
| lnc-CCDC146-1:8 | 30.25421252 | 12.65363917 | 0.418243878 | -1.257583672 | 0.0381941 | 1 | Down |
| lnc-CD300C-2:1 | 105.9913868 | 47.98172143 | 0.452694534 | -1.143390208 | 0.0339615 | 1 | Down |
| lnc-CDH12-1:2 | 0 | 4.125435597 | +∞ | +∞ | 0.0427865 | 1 | Up |
| lnc-CDHR4-3:1 | 10.67550458 | 33.29500796 | 3.118822882 | 1.641001624 | 0.0072072 | 1 | Up |
| lnc-CDK10-2:5 | 0.828115964 | 6.567549543 | 7.930712398 | 2.987450466 | 0.0499035 | 1 | Up |
| lnc-CELF6-1:5 | 112.0516242 | 49.58921499 | 0.44255686 | -1.176065269 | 0.006004 | 1 | Down |
| lnc-CEP170-11:1 | 0 | 16.6385607 | +∞ | +∞ | 4.38E-05 | 0.2178755 | Up |
| lnc-CGNL1-6:1 | 0 | 24.99019815 | +∞ | +∞ | 0.0134384 | 1 | Up |
| lnc-CHCHD7-3:1 | 27.20392097 | 10.68110711 | 0.392631162 | -1.348753414 | 0.0229357 | 1 | Down |
| lnc-CHMP4C-10:1 | 0.213520901 | 5.534989794 | 25.92247298 | 4.696131451 | 0.0147896 | 1 | Up |
| lnc-CIDEA-2:3 | 0.604031653 | 8.4325504 | 13.96044456 | 3.803272979 | 0.028828 | 1 | Up |
| lnc-CLEC2D-8:6 | 26.42141794 | 0 | 0 | -∞ | 0.0001393 | 0.4290045 | Down |
| lnc-CLEC4C-1:1 | 86.81710042 | 40.99814847 | 0.472235865 | -1.082420482 | 0.0206743 | 1 | Down |
| lnc-CMC1-1:3 | 37.47147891 | 84.14486059 | 2.245570846 | 1.167082239 | 0.0089413 | 1 | Up |
| lnc-CMC1-4:1 | 5.489433233 | 0 | 0 | -∞ | 0.0186544 | 1 | Down |
| lnc-CMC1-8:1 | 20.87688089 | 44.83694899 | 2.147684284 | 1.102781928 | 0.0383127 | 1 | Up |
| lnc-CNIH3-2:5 | 3.94246315 | 0 | 0 | -∞ | 0.0174411 | 1 | Down |
| lnc-CNNM4-1:2 | 0 | 10.79851909 | +∞ | +∞ | 0.0115834 | 1 | Up |
| lnc-CNST-1:4 | 0 | 3.536024575 | +∞ | +∞ | 0.0352445 | 1 | Up |
| lnc-CNTNAP3B-7:1 | 3.697252018 | 0 | 0 | -∞ | 0.0391271 | 1 | Down |
| lnc-COG6-4:1 | 1.61808727 | 8.190613981 | 5.061911142 | 2.339682182 | 0.0436255 | 1 | Up |
| lnc-COL28A1-1:9 | 9.171779206 | 1.028640635 | 0.112152791 | -3.156462573 | 0.0101625 | 1 | Down |
| lnc-COL4A1-5:1 | 20.716762 | 7.148627448 | 0.345064902 | -1.535060356 | 0.0330604 | 1 | Down |
| lnc-COLQ-2:1 | 11.2266551 | 0.439236244 | 0.039124409 | -4.675787237 | 0.0005124 | 0.9351433 | Down |
| lnc-CPSF2-3:1 | 81.02108636 | 229.4800453 | 2.832349646 | 1.501999373 | 0.0149384 | 1 | Up |
| lnc-CREBBP-1:1 | 3.692016059 | 14.96907592 | 4.054444966 | 2.01950443 | 0.0143091 | 1 | Up |
| lnc-CRK-3:4 | 0 | 3.518731936 | +∞ | +∞ | 0.0336259 | 1 | Up |
| lnc-CRYBA4-1:32 | 9.539311343 | 24.59434035 | 2.578209209 | 1.366369336 | 0.0330112 | 1 | Up |
| lnc-CSDC2-1:1 | 12.61706708 | 1.103539984 | 0.087464066 | -3.515165776 | 0.0016809 | 1 | Down |
| lnc-CSE1L-3:1 | 5.53961976 | 0 | 0 | -∞ | 0.0308637 | 1 | Down |
| lnc-CSNK1D-1:1 | 14.957652 | 44.90234054 | 3.001964515 | 1.585906924 | 0.0136843 | 1 | Up |
| lnc-CTAGE5-1:4 | 5.781605299 | 0 | 0 | -∞ | 0.0443556 | 1 | Down |
| lnc-CYP2E1-11:1 | 2.380379338 | 13.3824817 | 5.621995402 | 2.491082274 | 0.0154432 | 1 | Up |
| lnc-DCAF6-1:2 | 2.23913676 | 13.61108626 | 6.078720382 | 2.603767657 | 0.0287744 | 1 | Up |
| lnc-DDR2-5:1 | 8.368161163 | 1.738372061 | 0.207736446 | -2.267173744 | 0.0448677 | 1 | Down |
| lnc-DEFB128-1:1 | 6.939529722 | 20.71134003 | 2.984545186 | 1.577511096 | 0.0438549 | 1 | Up |
| lnc-DEPDC4-2:3 | 7.408521579 | 0.692499134 | 0.093473324 | -3.419301499 | 0.0227136 | 1 | Down |
| lnc-DNAH10OS-8:1 | 39.49765298 | 14.80338189 | 0.374791431 | -1.415840126 | 0.012903 | 1 | Down |
| lnc-DNAH9-1:1 | 0.805375537 | 16.97879518 | 21.08183625 | 4.397928627 | 0.0309265 | 1 | Up |
| lnc-DNAJC14-2:1 | 1.220008353 | 7.88657709 | 6.464363191 | 2.692508256 | 0.0317346 | 1 | Up |
| lnc-DNAL4-3:4 | 37.03425554 | 82.64544562 | 2.231594626 | 1.158074982 | 0.011736 | 1 | Up |
| lnc-DOCK3-1:3 | 3.246476731 | 22.24762163 | 6.852851097 | 2.77670434 | 0.0013858 | 1 | Up |
| lnc-DPY19L1-1:1 | 19.53966848 | 0.410521801 | 0.02100966 | -5.57280334 | 0.0161535 | 1 | Down |
| lnc-DYDC1-1:1 | 0.980519811 | 10.1157466 | 10.31671823 | 3.366912214 | 0.008978 | 1 | Up |
| lnc-EFNA4-1:6 | 23.87083955 | 7.128061845 | 0.2986096 | -1.743667549 | 0.0059385 | 1 | Down |
| lnc-EGLN1-1:2 | 16.80541904 | 1.511605043 | 0.089947477 | -3.474773375 | 0.0469027 | 1 | Down |
| lnc-EGLN1-1:3 | 5.339066214 | 23.81467293 | 4.460456562 | 2.157191388 | 0.0028037 | 1 | Up |
| lnc-EID2-1:1 | 0 | 3.84267956 | +∞ | +∞ | 0.0258963 | 1 | Up |
| lnc-EID2B-1:1 | 4.541618173 | 27.16674822 | 5.981733202 | 2.580563565 | 0.0012607 | 1 | Up |
| lnc-EID3-1:3 | 80.97400404 | 5.753537928 | 0.071054136 | -3.814937552 | 0.0114913 | 1 | Down |
| lnc-EIF5B-4:1 | 22.58051412 | 53.49154061 | 2.368924832 | 1.244232421 | 0.0473661 | 1 | Up |
| lnc-ELF2-2:2 | 0 | 5.238435885 | +∞ | +∞ | 0.011983 | 1 | Up |
| lnc-EPG5-1:3 | 8.124075107 | 1.313476565 | 0.161677058 | -2.628813118 | 0.0183982 | 1 | Down |
| lnc-ESR2-3:3 | 6.510017014 | 0.563954667 | 0.086628755 | -3.529010212 | 0.0220436 | 1 | Down |
| lnc-EXOSC2-3:1 | 1.071303725 | 17.50073972 | 16.33592725 | 4.029976441 | 0.0274759 | 1 | Up |
| lnc-EXTL3-6:4 | 0.808550416 | 6.414616298 | 7.93347721 | 2.987953332 | 0.039926 | 1 | Up |
| lnc-EYA2-2:1 | 4.071648005 | 15.33821278 | 3.767077301 | 1.913445638 | 0.02064 | 1 | Up |
| lnc-F13A1-2:3 | 15.61079158 | 0 | 0 | -∞ | 5.85E-05 | 0.2588119 | Down |
| lnc-FAIM-3:1 | 4.053001936 | 23.10234701 | 5.700058222 | 2.510976655 | 0.0160899 | 1 | Up |
| lnc-FAM106B-1:3 | 0.427041803 | 6.239308129 | 14.61053248 | 3.868936853 | 0.0431099 | 1 | Up |
| lnc-FAM106B-1:5 | 0 | 4.59479218 | +∞ | +∞ | 0.0258867 | 1 | Up |
| lnc-FAM108B1-1:1 | 2.460389058 | 25.72680415 | 10.45639675 | 3.386313883 | 0.0217478 | 1 | Up |
| lnc-FAM108B1-3:1 | 43.87006705 | 14.19241839 | 0.323510296 | -1.628116466 | 0.0080308 | 1 | Down |
| lnc-FAM153C-1:8 | 0.414864785 | 12.68478205 | 30.575702 | 4.934313717 | 0.0058787 | 1 | Up |
| lnc-FAM200B-1:16 | 7.193911131 | 0 | 0 | -∞ | 0.0005076 | 0.9351433 | Down |
| lnc-FAM32A-2:1 | 0.427041803 | 15.23640397 | 35.67895197 | 5.157001333 | 0.0005915 | 0.9351433 | Up |
| lnc-FAM3C-2:1 | 1.043250472 | 9.263329981 | 8.879296226 | 3.150445333 | 0.024648 | 1 | Up |
| lnc-FAM46A-3:1 | 31.32034849 | 72.772699 | 2.323495827 | 1.216297054 | 0.0302521 | 1 | Up |
| lnc-FAM53B-1:1 | 10.98410935 | 2.635926388 | 0.239976343 | -2.059035907 | 0.0231397 | 1 | Down |
| lnc-FAM53B-1:2 | 7.304324485 | 19.81024144 | 2.712125054 | 1.439423701 | 0.0386375 | 1 | Up |
| lnc-FAM82A2-1:1 | 30.19867152 | 72.13491545 | 2.388678436 | 1.256212651 | 0.0109145 | 1 | Up |
| lnc-FAM86B2-7:1 | 8.332113394 | 22.73494167 | 2.728592446 | 1.448156924 | 0.0349389 | 1 | Up |
| lnc-FAM96A-1:1 | 1.810429016 | 9.085618216 | 5.018489062 | 2.327253071 | 0.0445145 | 1 | Up |
| lnc-FBXL2-4:1 | 0 | 22.13722064 | +∞ | +∞ | 0.0259162 | 1 | Up |
| lnc-FBXL3-7:1 | 0.616948513 | 11.9331965 | 19.34228908 | 4.273686637 | 0.000611 | 0.9351433 | Up |
| lnc-FBXO44-1:1 | 0.201343884 | 6.645620035 | 33.00631684 | 5.044670253 | 0.0051913 | 1 | Up |
| lnc-FCRL1-1:3 | 0 | 5.847288966 | +∞ | +∞ | 0.0429418 | 1 | Up |
| lnc-FGA-3:1 | 23.81816942 | 6.944832613 | 0.291577094 | -1.77805071 | 0.0205057 | 1 | Down |
| lnc-FGFR1OP-3:7 | 0.604031653 | 8.451500792 | 13.99181774 | 3.806511497 | 0.0094713 | 1 | Up |
| lnc-FLRT2-2:6 | 3.164134805 | 0 | 0 | -∞ | 0.0372892 | 1 | Down |
| lnc-FMO2-1:1 | 0 | 3.989122066 | +∞ | +∞ | 0.0352751 | 1 | Up |
| lnc-FOXN2-2:1 | 18.35252002 | 0 | 0 | -∞ | 0.0014612 | 1 | Down |
| lnc-FURIN-1:1 | 12.99773767 | 3.037313524 | 0.233680168 | -2.097392794 | 0.0292255 | 1 | Down |
| lnc-GAS6-3:2 | 11.63917171 | 1.753463257 | 0.150651893 | -2.730709289 | 0.0480816 | 1 | Down |
| lnc-GATS-3:3 | 95.49422659 | 43.94547081 | 0.460189819 | -1.11969903 | 0.0119971 | 1 | Down |
| lnc-GFAP-4:1 | 12.78238744 | 2.184063966 | 0.170865105 | -2.549070307 | 0.0076624 | 1 | Down |
| lnc-GGCT-1:12 | 8.13963273 | 22.79413114 | 2.800388162 | 1.485626813 | 0.0290491 | 1 | Up |
| lnc-GGCT-1:5 | 18.82458364 | 5.379933806 | 0.285792977 | -1.806957629 | 0.0480381 | 1 | Down |
| lnc-GGNBP1-5:1 | 4.219330325 | 0 | 0 | -∞ | 0.0121252 | 1 | Down |
| lnc-GLT6D1-2:1 | 0.201343884 | 4.849386616 | 24.08509519 | 4.590068721 | 0.0246632 | 1 | Up |
| lnc-GNA13-2:10 | 49.49017448 | 12.45167101 | 0.25159885 | -1.990802764 | 0.0047666 | 1 | Down |
| lnc-GOLGA6L6-12:1 | 39.26234532 | 7.750547353 | 0.197404085 | -2.34077625 | 0.0015845 | 1 | Down |
| lnc-GOLPH3-2:1 | 3.299003312 | 0 | 0 | -∞ | 0.037625 | 1 | Down |
| lnc-GPR149-5:1 | 19.63835248 | 52.80352501 | 2.688796072 | 1.42696034 | 0.0363545 | 1 | Up |
| lnc-GPR37-2:7 | 4.482607914 | 18.43191958 | 4.111874145 | 2.039796108 | 0.0100221 | 1 | Up |
| lnc-GPR37-5:1 | 10.35653032 | 2.077444055 | 0.200592669 | -2.317659216 | 0.0486306 | 1 | Down |
| lnc-GRAP-2:1 | 6.845246186 | 29.17803599 | 4.262525437 | 2.091708443 | 0.017713 | 1 | Up |
| lnc-GS1-211B7.1.1-6:1 | 33.5473333 | 14.82198218 | 0.441822962 | -1.178459695 | 0.0353169 | 1 | Down |
| lnc-GSDMC-9:1 | 8.313883768 | 1.925817634 | 0.231638749 | -2.110051485 | 0.043775 | 1 | Down |
| lnc-H2AFB2-1:2 | 0.214260745 | 8.107132159 | 37.83769237 | 5.2417522 | 0.0165497 | 1 | Up |
| lnc-H2AFB3-1:1 | 0.214260745 | 8.107132159 | 37.83769237 | 5.2417522 | 0.0165497 | 1 | Up |
| lnc-HDAC11-2:1 | 7.620758613 | 0.740885537 | 0.097219394 | -3.362612042 | 0.0136834 | 1 | Down |
| lnc-HERPUD2-1:1 | 0.407409295 | 5.227317683 | 12.83062942 | 3.68152004 | 0.0375604 | 1 | Up |
| lnc-HES5-1:7 | 18.60762051 | 48.65177143 | 2.614615415 | 1.386598755 | 0.0093425 | 1 | Up |
| lnc-HFE2-1:1 | 4.957582133 | 24.66305399 | 4.974815006 | 2.314642878 | 0.0010379 | 1 | Up |
| lnc-HIST1H2AI-1:3 | 1.413156903 | 18.53018379 | 13.11261599 | 3.712883629 | 0.0021167 | 1 | Up |
| lnc-HIVEP3-1:1 | 6.073971574 | 47.05946993 | 7.747726402 | 2.953773009 | 2.43E-05 | 0.1380816 | Up |
| lnc-HMGB2-8:1 | 25.9557711 | 55.13649895 | 2.124248158 | 1.086952314 | 0.022198 | 1 | Up |
| lnc-HMMR-2:1 | 13.67668491 | 3.88264618 | 0.28388796 | -1.816606431 | 0.034223 | 1 | Down |
| lnc-HN1L-2:1 | 10.59835284 | 2.30667532 | 0.2176447 | -2.199953209 | 0.0363866 | 1 | Down |
| lnc-HPS6-1:1 | 14.47475044 | 34.23527626 | 2.365172126 | 1.24194518 | 0.0328674 | 1 | Up |
| lnc-HTR2C-3:2 | 9.297503521 | 0 | 0 | -∞ | 0.0461122 | 1 | Down |
| lnc-HTR2C-4:1 | 15.45005075 | 4.386038917 | 0.283885081 | -1.816621059 | 0.0384006 | 1 | Down |
| lnc-HTR2C-5:1 | 0.394492434 | 5.934593136 | 15.04361713 | 3.91107959 | 0.031545 | 1 | Up |
| lnc-HUNK-2:1 | 0.594222711 | 8.156427254 | 13.72621258 | 3.778861698 | 0.0145234 | 1 | Up |
| lnc-HYOU1-1:3 | 12.94704394 | 1.639493611 | 0.126630729 | -2.981300556 | 0.0252649 | 1 | Down |
| lnc-HYOU1-1:4 | 17.3656911 | 36.09711735 | 2.078645597 | 1.055643804 | 0.0473525 | 1 | Up |
| lnc-IFI44-8:1 | 10.26634125 | 30.61692243 | 2.982262295 | 1.576407151 | 0.0134541 | 1 | Up |
| lnc-IFITM1-3:2 | 59.25838923 | 29.34958916 | 0.495281589 | -1.013679103 | 0.0286688 | 1 | Down |
| lnc-IFNK-7:1 | 3.919193665 | 18.00487688 | 4.594025818 | 2.199758965 | 0.0054755 | 1 | Up |
| lnc-IGFBP7-1:2 | 34.01993629 | 8.438876383 | 0.248056796 | -2.011257614 | 0.0255105 | 1 | Down |
| lnc-IGLL5-1:1 | 74.91159538 | 25.70809903 | 0.343179169 | -1.542966112 | 0.0011416 | 1 | Down |
| lnc-IGLL5-1:3 | 0.201343884 | 10.0943823 | 50.13503313 | 5.647747171 | 0.0290502 | 1 | Up |
| lnc-IKZF1-7:1 | 6.365174617 | 17.81571301 | 2.79893547 | 1.484878226 | 0.0463404 | 1 | Up |
| lnc-IL17C-3:1 | 2.754243139 | 13.36608468 | 4.852906591 | 2.278849091 | 0.0175392 | 1 | Up |
| lnc-IL1R1-1:1 | 4.991328446 | 0.546325129 | 0.109454855 | -3.191592152 | 0.0425877 | 1 | Down |
| lnc-IL22RA2-1:1 | 3.213994322 | 15.34601754 | 4.774749426 | 2.255425024 | 0.0248488 | 1 | Up |
| lnc-ING2-5:2 | 24.38648951 | 10.33110846 | 0.423640658 | -1.239087039 | 0.0447273 | 1 | Down |
| lnc-IQCG-8:1 | 11.24290562 | 34.26673371 | 3.047853896 | 1.607793747 | 0.0465887 | 1 | Up |
| lnc-IRF9-2:1 | 57.91285462 | 17.81242032 | 0.307572825 | -1.701000051 | 0.0009863 | 1 | Down |
| lnc-ITGA2-1:2 | 6.940741631 | 0.878245187 | 0.126534776 | -2.982394156 | 0.032408 | 1 | Down |
| lnc-ITGA2-1:6 | 0 | 17.24878403 | +∞ | +∞ | 0.0005439 | 0.9351433 | Up |
| lnc-ITGAL-6:1 | 0.841099785 | 6.42342875 | 7.636940191 | 2.932994725 | 0.0337754 | 1 | Up |
| lnc-JMJD7-PLA2G4B-2:3 | 18.93434842 | 1.93685724 | 0.102293314 | -3.289216249 | 0.0121512 | 1 | Down |
| lnc-JPH2-1:1 | 43.76986769 | 111.1849295 | 2.540216259 | 1.344951325 | 0.0207279 | 1 | Up |
| lnc-KCTD17-5:1 | 25.13007364 | 6.655024968 | 0.264823138 | -1.916898915 | 0.00485 | 1 | Down |
| lnc-KDM4C-1:3 | 1.384821186 | 8.256748023 | 5.962320701 | 2.575873977 | 0.0399464 | 1 | Up |
| lnc-KHDRBS2-3:1 | 4.333780705 | 0 | 0 | -∞ | 0.0113498 | 1 | Down |
| lnc-KIAA0226-4:1 | 0 | 22.83687168 | +∞ | +∞ | 0.0441149 | 1 | Up |
| lnc-KIAA1429-4:1 | 13.04353857 | 0 | 0 | -∞ | 0.0231443 | 1 | Down |
| lnc-KIAA1609-3:2 | 22.17349192 | 1.563486286 | 0.070511505 | -3.825997516 | 0.0029852 | 1 | Down |
| lnc-KIAA1737-1:4 | 14.27178652 | 1.925241737 | 0.13489844 | -2.89005443 | 0.0035567 | 1 | Down |
| lnc-KLB-2:1 | 247.1107924 | 122.5376957 | 0.495881602 | -1.011932393 | 0.0390639 | 1 | Down |
| lnc-KRTAP10-12-3:1 | 1.867959588 | 11.2062233 | 5.999178663 | 2.584764997 | 0.0116129 | 1 | Up |
| lnc-LAX1-3:2 | 19.6827318 | 48.15503197 | 2.446562421 | 1.290756092 | 0.0197916 | 1 | Up |
| lnc-LDOC1L-4:1 | 0 | 4.554064956 | +∞ | +∞ | 0.0236108 | 1 | Up |
| lnc-LEPROTL1-4:7 | 4.772592769 | 23.50248076 | 4.924468083 | 2.2999679 | 0.0241738 | 1 | Up |
| lnc-LETM2-4:1 | 0.58683418 | 6.442358367 | 10.97815803 | 3.456564107 | 0.0219369 | 1 | Up |
| lnc-LGALS9B-11:3 | 0 | 5.206096626 | +∞ | +∞ | 0.0344286 | 1 | Up |
| lnc-LGSN-3:1 | 8.391200301 | 26.54128162 | 3.162989878 | 1.661288939 | 0.0112234 | 1 | Up |
| lnc-LHFPL3-4:1 | 15.19084964 | 52.66760424 | 3.467061125 | 1.793713272 | 0.0063698 | 1 | Up |
| lnc-LILRB4-1:2 | 0 | 6.448703979 | +∞ | +∞ | 0.0061311 | 1 | Up |
| lnc-LIMA1-1:7 | 57.30252351 | 18.73364234 | 0.326925259 | -1.612967247 | 0.0499444 | 1 | Down |
| lnc-LPCAT1-3:10 | 0.854823449 | 10.96243674 | 12.82421154 | 3.680798223 | 0.0050784 | 1 | Up |
| lnc-LRFN5-1:7 | 9.38195835 | 0.140988667 | 0.015027637 | -6.056237994 | 0.0311079 | 1 | Down |
| lnc-LRRC9-5:1 | 11.73117367 | 41.00511417 | 3.49539742 | 1.805456497 | 0.0077609 | 1 | Up |
| lnc-LRRFIP2-1:1 | 7.904923047 | 24.72704906 | 3.128056897 | 1.645266755 | 0.0107934 | 1 | Up |
| lnc-MAK-3:1 | 0 | 5.017817048 | +∞ | +∞ | 0.0079006 | 1 | Up |
| lnc-MB-7:1 | 3.394065999 | 0 | 0 | -∞ | 0.0326368 | 1 | Down |
| lnc-MBLAC1-2:2 | 10.52531505 | 2.33856611 | 0.222184904 | -2.170167298 | 0.0445906 | 1 | Down |
| lnc-MDK-4:2 | 25.77399411 | 55.65634638 | 2.15939936 | 1.11063008 | 0.0285746 | 1 | Up |
| lnc-MDM1-1:1 | 30.33570444 | 64.53861136 | 2.127480227 | 1.089145724 | 0.0401157 | 1 | Up |
| lnc-MDM1-1:10 | 17.46398278 | 59.07237695 | 3.382526064 | 1.758101051 | 0.0017008 | 1 | Up |
| lnc-MDM1-1:15 | 64.23358213 | 134.6330154 | 2.095991084 | 1.06763258 | 0.0095927 | 1 | Up |
| lnc-MDM1-1:18 | 2.188583116 | 10.84298161 | 4.954338509 | 2.308692445 | 0.0265864 | 1 | Up |
| lnc-MDM1-1:2 | 2.300021846 | 12.55810922 | 5.459995626 | 2.448899796 | 0.0092471 | 1 | Up |
| lnc-MED20-5:3 | 30.94081629 | 0 | 0 | -∞ | 0.0068581 | 1 | Down |
| lnc-METAP1-3:8 | 5.670071896 | 0.274719298 | 0.048450761 | -4.367336864 | 0.0180573 | 1 | Down |
| lnc-METTL14-1:2 | 60.80142583 | 15.0897796 | 0.248181344 | -2.010533422 | 0.0077641 | 1 | Down |
| lnc-MICAL2-4:1 | 13.77468913 | 33.94117181 | 2.464024522 | 1.301016614 | 0.0210686 | 1 | Up |
| lnc-MIDN-1:1 | 4.295301309 | 0.137359649 | 0.031979049 | -4.966729173 | 0.0301711 | 1 | Down |
| lnc-MLXIP-1:1 | 24.00366951 | 49.86001553 | 2.077183054 | 1.054628361 | 0.0335805 | 1 | Up |
| lnc-MPDZ-7:1 | 0 | 4.822840362 | +∞ | +∞ | 0.0199876 | 1 | Up |
| lnc-MPHOSPH8-7:1 | 34.03058416 | 74.26425174 | 2.182279664 | 1.125835998 | 0.0117268 | 1 | Up |
| lnc-MPPE1-5:1 | 0 | 26.60312942 | +∞ | +∞ | 0.0082636 | 1 | Up |
| lnc-MRPL33-1:2 | 21.88925451 | 51.15040162 | 2.336781347 | 1.224522747 | 0.0125988 | 1 | Up |
| lnc-MRPL35-1:2 | 20.35852434 | 55.62839302 | 2.732437386 | 1.450188437 | 0.0376112 | 1 | Up |
| lnc-MRPS5-12:1 | 0 | 3.919039659 | +∞ | +∞ | 0.0444104 | 1 | Up |
| lnc-MTERFD2-5:1 | 4.21692632 | 0 | 0 | -∞ | 0.0349003 | 1 | Down |
| lnc-MTPN-1:1 | 263.7971558 | 94.39550631 | 0.357833677 | -1.482638923 | 0.0269729 | 1 | Down |
| lnc-MVD-3:2 | 1.795472319 | 11.10439621 | 6.184665779 | 2.628695634 | 0.0145973 | 1 | Up |
| lnc-MYL6B-2:2 | 40.88205315 | 17.15903501 | 0.419720481 | -1.252499231 | 0.0409624 | 1 | Down |
| lnc-MYL7-3:1 | 2.016561386 | 11.66186329 | 5.783044031 | 2.531829087 | 0.0118169 | 1 | Up |
| lnc-MYOCD-3:1 | 27.5248827 | 56.93724864 | 2.068573707 | 1.048636364 | 0.0420981 | 1 | Up |
| lnc-MZT2B-1:2 | 1.25596457 | 9.155098374 | 7.289296682 | 2.865779621 | 0.0350406 | 1 | Up |
| lnc-NAA25-4:1 | 9.630883748 | 1.852790107 | 0.192380072 | -2.377968733 | 0.035411 | 1 | Down |
| lnc-NADSYN1-1:2 | 0.42852149 | 11.86827153 | 27.69586076 | 4.791598472 | 0.0045813 | 1 | Up |
| lnc-NAGS-1:3 | 17.49966254 | 2.250803581 | 0.128619828 | -2.958815033 | 0.0018733 | 1 | Down |
| lnc-NDUFA13-1:1 | 1.406179976 | 9.501392701 | 6.756882381 | 2.756357742 | 0.0167813 | 1 | Up |
| lnc-NDUFB5-3:3 | 0 | 13.06335874 | +∞ | +∞ | 0.0105508 | 1 | Up |
| lnc-NFXL1-1:4 | 63.32932614 | 28.0908652 | 0.443568042 | -1.172772668 | 0.0177873 | 1 | Down |
| lnc-NHS-2:1 | 8.247508281 | 0 | 0 | -∞ | 0.0131298 | 1 | Down |
| lnc-NNT-2:1 | 4.933218192 | 24.60931367 | 4.988490821 | 2.31860342 | 0.0044052 | 1 | Up |
| lnc-NNT-5:1 | 4.135432065 | 14.64696845 | 3.541823012 | 1.824492121 | 0.0286393 | 1 | Up |
| lnc-NPAS1-3:1 | 0.386297099 | 17.18366179 | 44.4830205 | 5.475182848 | 0.0027665 | 1 | Up |
| lnc-NPBWR1-1:2 | 5.582629491 | 24.51580594 | 4.391444207 | 2.134695474 | 0.0182887 | 1 | Up |
| lnc-NPC1-7:3 | 0.402687768 | 9.661919948 | 23.99357693 | 4.584576343 | 0.0078817 | 1 | Up |
| lnc-NR4A2-3:1 | 0 | 3.320833432 | +∞ | +∞ | 0.0403935 | 1 | Up |
| lnc-NRG1-2:4 | 7.564257247 | 1.338453162 | 0.176944427 | -2.498631773 | 0.0474104 | 1 | Down |
| lnc-OCM-2:1 | 0 | 5.701722074 | +∞ | +∞ | 0.0438504 | 1 | Up |
| lnc-OCM-3:13 | 0.386297099 | 12.5510404 | 32.49064106 | 5.021952304 | 0.0306361 | 1 | Up |
| lnc-OR4C12-9:4 | 6.677936206 | 0 | 0 | -∞ | 0.0171326 | 1 | Down |
| lnc-OSBPL2-1:1 | 83.56939148 | 200.3582385 | 2.397507447 | 1.261535297 | 0.0011672 | 1 | Up |
| lnc-OST4-2:1 | 30.25726856 | 66.10655083 | 2.18481555 | 1.127511487 | 0.0147323 | 1 | Up |
| lnc-OXNAD1-4:1 | 0.214260745 | 30.58955441 | 142.7678896 | 7.157527725 | 0.0066127 | 1 | Up |
| lnc-PANK1-5:1 | 11.59025277 | 26.73807417 | 2.30694487 | 1.205983528 | 0.0425393 | 1 | Up |
| lnc-PARP8-2:2 | 14.99076814 | 32.37898283 | 2.1599282 | 1.110983355 | 0.0486252 | 1 | Up |
| lnc-PAWR-13:1 | 0.213520901 | 6.142741794 | 28.76880791 | 4.846433536 | 0.0459912 | 1 | Up |
| lnc-PCDH20-13:1 | 0 | 7.292240295 | +∞ | +∞ | 0.0048781 | 1 | Up |
| lnc-PDZD8-8:1 | 40.72817273 | 19.0524143 | 0.467794478 | -1.096053263 | 0.0412646 | 1 | Down |
| lnc-PEBP1-2:2 | 28.99471882 | 57.99858041 | 2.000315325 | 1.000227441 | 0.0338636 | 1 | Up |
| lnc-PEX14-2:1 | 3.463126796 | 29.29299854 | 8.458540582 | 3.080408765 | 0.0459677 | 1 | Up |
| lnc-PGPEP1L-3:1 | 26.69427247 | 60.58136738 | 2.2694519 | 1.182343911 | 0.0135051 | 1 | Up |
| lnc-PIGC-2:2 | 29.85657672 | 8.468961648 | 0.283654812 | -1.817791758 | 0.0231009 | 1 | Down |
| lnc-PION-4:1 | 0.990328752 | 7.084361532 | 7.153545244 | 2.838658408 | 0.0453178 | 1 | Up |
| lnc-PLA2G10-4:1 | 0.841906588 | 6.53158654 | 7.758089356 | 2.955701393 | 0.034314 | 1 | Up |
| lnc-PLAC9-2:1 | 0.415604629 | 12.9293191 | 31.10966095 | 4.959290766 | 0.0221708 | 1 | Up |
| lnc-PLAGL2-6:1 | 10.75847524 | 29.55343292 | 2.746990839 | 1.457852099 | 0.0216688 | 1 | Up |
| lnc-PLEKHA7-2:1 | 45.99716387 | 104.3395824 | 2.268391649 | 1.18166975 | 0.004655 | 1 | Up |
| lnc-PLEKHG2-1:1 | 15.33946429 | 55.18223053 | 3.597402718 | 1.846955674 | 0.0199927 | 1 | Up |
| lnc-PLEKHG4-1:1 | 17.15186956 | 2.414596853 | 0.140777473 | -2.828511601 | 0.0027587 | 1 | Down |
| lnc-POLE3-3:2 | 17.77125331 | 5.894457941 | 0.331684988 | -1.592114378 | 0.0412706 | 1 | Down |
| lnc-POLK-4:1 | 39.00662635 | 15.27090754 | 0.391495214 | -1.352933423 | 0.0153197 | 1 | Down |
| lnc-PPIAL4C-5:2 | 2.116773729 | 14.85047506 | 7.015617617 | 2.810570116 | 0.0022063 | 1 | Up |
| lnc-PPIAL4C-5:3 | 2.116773729 | 14.85047506 | 7.015617617 | 2.810570116 | 0.0022063 | 1 | Up |
| lnc-PPIAL4G-4:4 | 83.33162576 | 41.23580231 | 0.494839767 | -1.014966649 | 0.0258734 | 1 | Down |
| lnc-PPP2R4-3:2 | 30.95055243 | 7.609694529 | 0.245866194 | -2.024054713 | 0.0129371 | 1 | Down |
| lnc-PPP6R1-2:1 | 7.061969619 | 24.75762529 | 3.505767742 | 1.809730421 | 0.009648 | 1 | Up |
| lnc-PRKAR1A-2:1 | 0 | 419.1896169 | +∞ | +∞ | 0.0420665 | 1 | Up |
| lnc-PROC-1:2 | 8.96557215 | 0.577114942 | 0.064370119 | -3.957465064 | 0.0318659 | 1 | Down |
| lnc-PRPF4B-1:2 | 66.23569004 | 32.18449763 | 0.485908694 | -1.04124285 | 0.0248452 | 1 | Down |
| lnc-PRPF4B-1:3 | 14.90524634 | 0.40896548 | 0.027437687 | -5.187697336 | 0.0178963 | 1 | Down |
| lnc-PRPF4B-4:4 | 3.344642399 | 15.5464652 | 4.648169621 | 2.216662717 | 0.014142 | 1 | Up |
| lnc-PRPF4B-4:5 | 4.439896893 | 17.29994649 | 3.89647483 | 1.962169497 | 0.0330934 | 1 | Up |
| lnc-PRR4-1:15 | 3.534041236 | 0 | 0 | -∞ | 0.031243 | 1 | Down |
| lnc-PRRC2A-3:7 | 43.77290341 | 17.44434305 | 0.398519214 | -1.327278813 | 0.0203664 | 1 | Down |
| lnc-PSMB9-6:1 | 46.54377575 | 13.03857911 | 0.280135827 | -1.835801592 | 0.0134042 | 1 | Down |
| lnc-PSMD8-1:1 | 0 | 5.25661252 | +∞ | +∞ | 0.0064548 | 1 | Up |
| lnc-PTBP3-6:1 | 0 | 9.493789796 | +∞ | +∞ | 0.0028239 | 1 | Up |
| lnc-PTH2R-1:6 | 4.469614247 | 0.273681201 | 0.061231504 | -4.029582081 | 0.0456688 | 1 | Down |
| lnc-PTMS-1:1 | 5.491403101 | 0.552548565 | 0.100620653 | -3.313001639 | 0.0465793 | 1 | Down |
| lnc-PTS-1:10 | 8.257511263 | 1.378539334 | 0.166943682 | -2.5825666 | 0.0419884 | 1 | Down |
| lnc-PTS-1:3 | 7.032549414 | 1.062159644 | 0.151034793 | -2.727047163 | 0.0453503 | 1 | Down |
| lnc-RAB23-17:1 | 16.09685545 | 4.576662687 | 0.284320295 | -1.814411012 | 0.0158621 | 1 | Down |
| lnc-RAB28-6:1 | 34.17038003 | 69.81780209 | 2.043225801 | 1.030848648 | 0.0250327 | 1 | Up |
| lnc-RAB33B-6:1 | 10.6672326 | 2.556521446 | 0.23966117 | -2.060931913 | 0.0309416 | 1 | Down |
| lnc-RAB39B-1:1 | 7.600606824 | 0.972241424 | 0.12791629 | -2.966728098 | 0.029173 | 1 | Down |
| lnc-RAB3D-2:1 | 108.5997675 | 218.0192678 | 2.007548201 | 1.005434627 | 0.0107789 | 1 | Up |
| lnc-RABEP2-2:1 | 1.813342676 | 8.435579913 | 4.651950249 | 2.217835668 | 0.0462899 | 1 | Up |
| lnc-RBL2-1:5 | 2.529957948 | 27.42858933 | 10.84151986 | 3.438495115 | 0.0371877 | 1 | Up |
| lnc-RBM12B-1:1 | 0.386297099 | 5.83117245 | 15.09504591 | 3.91600324 | 0.0337476 | 1 | Up |
| lnc-RBM28-3:2 | 2.412092593 | 17.90631022 | 7.423558394 | 2.892110891 | 0.0018666 | 1 | Up |
| lnc-RBPMS2-3:1 | 24.9451357 | 0.453593465 | 0.018183644 | -5.781214851 | 0.0076293 | 1 | Down |
| lnc-RCOR2-3:1 | 2.987376925 | 16.25182285 | 5.440164821 | 2.443650362 | 0.0068182 | 1 | Up |
| lnc-RELL1-6:1 | 0 | 4.700277965 | +∞ | +∞ | 0.0101671 | 1 | Up |
| lnc-REP15-2:1 | 3.176491457 | 0 | 0 | -∞ | 0.0370172 | 1 | Down |
| lnc-RNASE13-4:2 | 14.26006509 | 34.42182187 | 2.413861483 | 1.271342891 | 0.0342836 | 1 | Up |
| lnc-RNF111-2:1 | 0 | 42.70675223 | +∞ | +∞ | 0.0288668 | 1 | Up |
| lnc-RNF135-1:12 | 2.415760722 | 12.5566324 | 5.197796406 | 2.377900126 | 0.014374 | 1 | Up |
| lnc-RNF182-2:1 | 1.409407189 | 12.68485026 | 9.000131657 | 3.169946106 | 0.0078257 | 1 | Up |
| lnc-RNF19A-8:4 | 91.89946786 | 21.98922292 | 0.239274758 | -2.063259886 | 2.35E-05 | 0.1380816 | Down |
| lnc-RNF19B-1:7 | 7.382699413 | 0.711361124 | 0.096355152 | -3.375494383 | 0.0123028 | 1 | Down |
| lnc-RNF214-4:1 | 5.720112668 | 0.584372977 | 0.102161096 | -3.291082193 | 0.0395046 | 1 | Down |
| lnc-RNF219-6:1 | 402.4112542 | 160.7422926 | 0.399447806 | -1.323921089 | 0.0014424 | 1 | Down |
| lnc-RNF24-4:1 | 18.94111607 | 53.85978227 | 2.843537945 | 1.507687056 | 0.0306065 | 1 | Up |
| lnc-RNF39-4:13 | 10.99478212 | 0.45322029 | 0.041221398 | -4.600462754 | 0.0034292 | 1 | Down |
| lnc-RNPEPL1-1:1 | 0 | 4.250924694 | +∞ | +∞ | 0.019048 | 1 | Up |
| lnc-RP1-239B22.1.1-1:3 | 7.399090082 | 0 | 0 | -∞ | 0.0009894 | 1 | Down |
| lnc-RP1-239B22.1.1-2:2 | 0 | 7.790275093 | +∞ | +∞ | 0.019267 | 1 | Up |
| lnc-RP11-108K14.4.1-1:2 | 54.69102448 | 26.85526973 | 0.491036143 | -1.026098876 | 0.0472646 | 1 | Down |
| lnc-RP11-1277H1.1.1-11:1 | 11.70293729 | 1.567631679 | 0.133951985 | -2.900212136 | 0.0440605 | 1 | Down |
| lnc-RP11-43D2.2.1-6:1 | 0 | 4.035471197 | +∞ | +∞ | 0.0386112 | 1 | Up |
| lnc-RP11-565P22.6.1-1:1 | 16.09547832 | 2.913602925 | 0.181019965 | -2.46577927 | 0.0497335 | 1 | Down |
| lnc-RP11-62N21.1.1-16:1 | 6.425223588 | 0.292186488 | 0.045474914 | -4.458785288 | 0.0082969 | 1 | Down |
| lnc-RP11-62N21.1.1-5:2 | 0 | 8.706894754 | +∞ | +∞ | 0.0101065 | 1 | Up |
| lnc-RP11-631B21.2.1-6:3 | 0 | 5.484330371 | +∞ | +∞ | 0.0148765 | 1 | Up |
| lnc-RP11-706O15.1.1-1:4 | 13.37860278 | 44.95900279 | 3.36051556 | 1.748682584 | 0.0040529 | 1 | Up |
| lnc-RP11-706O15.1.1-2:8 | 4.427158106 | 14.55014569 | 3.286565634 | 1.716580797 | 0.0326089 | 1 | Up |
| lnc-RPF1-1:11 | 16.50385157 | 41.68505541 | 2.525777406 | 1.336727502 | 0.020454 | 1 | Up |
| lnc-RPL10-1:1 | 7.869167648 | 1.127909334 | 0.143332737 | -2.802559942 | 0.030327 | 1 | Down |
| lnc-RPL10L-2:1 | 15.67823626 | 2.938467905 | 0.187423372 | -2.415627225 | 0.0040836 | 1 | Down |
| lnc-RPL35A-4:1 | 16.17283115 | 34.85998411 | 2.15546578 | 1.107999658 | 0.0451322 | 1 | Up |
| lnc-RPL6-1:1 | 0.201343884 | 8.225411163 | 40.85255034 | 5.35235424 | 0.0012979 | 1 | Up |
| lnc-RPLP0-3:1 | 14.54057587 | 36.43107374 | 2.505476678 | 1.325085109 | 0.0191185 | 1 | Up |
| lnc-RPRML-3:20 | 0.407409295 | 8.378278139 | 20.56476926 | 4.362102979 | 0.0182861 | 1 | Up |
| lnc-RPRML-3:8 | 0.201343884 | 5.520002367 | 27.41579358 | 4.776935329 | 0.0213537 | 1 | Up |
| lnc-RPUSD2-2:1 | 16.64254795 | 42.76441619 | 2.569583476 | 1.36153452 | 0.0131154 | 1 | Up |
| lnc-S100P-1:1 | 7.732661282 | 23.34381968 | 3.018859722 | 1.59400372 | 0.0158319 | 1 | Up |
| lnc-S1PR1-2:2 | 0.200537081 | 10.06843796 | 50.20736276 | 5.649827042 | 0.0366781 | 1 | Up |
| lnc-SAMD12-5:1 | 48.40331478 | 19.84770636 | 0.410048495 | -1.286133554 | 0.0131628 | 1 | Down |
| lnc-SAV1-1:1 | 7.136660234 | 29.26194488 | 4.100229509 | 2.035704666 | 0.0119972 | 1 | Up |
| lnc-SBDS-17:4 | 81.07992346 | 29.37541677 | 0.362301979 | -1.464735407 | 0.0014451 | 1 | Down |
| lnc-SBK2-1:1 | 0.214260745 | 11.19876989 | 52.26701645 | 5.707828903 | 0.014535 | 1 | Up |
| lnc-SBNO1-1:1 | 0 | 35.12864977 | +∞ | +∞ | 0.00033 | 0.8207759 | Up |
| lnc-SCN8A-2:9 | 0.213520901 | 4.680026927 | 21.91835506 | 4.454067625 | 0.0282141 | 1 | Up |
| lnc-SDCBP-2:5 | 0.615334907 | 8.211416086 | 13.34462908 | 3.7381873 | 0.0140112 | 1 | Up |
| lnc-SEC24C-2:1 | 12.03951716 | 2.854784071 | 0.237117821 | -2.076324001 | 0.035309 | 1 | Down |
| lnc-SERPINB6-8:3 | 1.973709888 | 10.07273537 | 5.103452861 | 2.351473667 | 0.0268651 | 1 | Up |
| lnc-SFTA2-14:1 | 21.41238906 | 58.9157168 | 2.751477971 | 1.460206778 | 0.0025801 | 1 | Up |
| lnc-SH2B1-1:1 | 0 | 14.12574384 | +∞ | +∞ | 0.0399564 | 1 | Up |
| lnc-SH3D19-11:1 | 23.2861496 | 8.927101505 | 0.38336529 | -1.383208372 | 0.0343885 | 1 | Down |
| lnc-SIGIRR-1:2 | 0.214260745 | 7.073466882 | 33.01335893 | 5.044978027 | 0.005001 | 1 | Up |
| lnc-SIRT3-1:1 | 7.807562402 | 21.53190671 | 2.75782704 | 1.463531979 | 0.0342688 | 1 | Up |
| lnc-SLC12A7-4:3 | 0.415604629 | 6.004345551 | 14.44725378 | 3.852723378 | 0.0204933 | 1 | Up |
| lnc-SLC14A1-2:1 | 1.40165277 | 10.30679667 | 7.353316662 | 2.878395114 | 0.0129997 | 1 | Up |
| lnc-SLC24A3-1:2 | 0 | 4.06441337 | +∞ | +∞ | 0.0276206 | 1 | Up |
| lnc-SLC29A1-2:1 | 82.22433522 | 174.6662441 | 2.124264594 | 1.086963477 | 0.0072966 | 1 | Up |
| lnc-SLC30A10-8:1 | 2.112267707 | 9.469241465 | 4.482974121 | 2.164456171 | 0.0388651 | 1 | Up |
| lnc-SLC38A8-10:1 | 15.51332495 | 41.81250925 | 2.695264194 | 1.430426695 | 0.0093319 | 1 | Up |
| lnc-SLC38A8-7:2 | 20.19020054 | 4.804018229 | 0.237938114 | -2.07134171 | 0.0420081 | 1 | Down |
| lnc-SLC39A4-1:1 | 3.469507644 | 16.44266223 | 4.739191815 | 2.244641054 | 0.0347284 | 1 | Up |
| lnc-SLC7A1-7:2 | 7.412664923 | 21.31330054 | 2.875254819 | 1.52368982 | 0.0266198 | 1 | Up |
| lnc-SLFN5-1:1 | 6.345347789 | 18.01469534 | 2.839039866 | 1.505403108 | 0.0488705 | 1 | Up |
| lnc-SNAP23-1:4 | 59.12552334 | 29.33584157 | 0.496162062 | -1.011116669 | 0.0275777 | 1 | Down |
| lnc-SNAPC3-5:1 | 51.80801086 | 152.1109258 | 2.9360503 | 1.553876684 | 0.027729 | 1 | Up |
| lnc-SNX11-12:1 | 26.45334478 | 59.1096164 | 2.23448554 | 1.159942709 | 0.0178407 | 1 | Up |
| lnc-SP110-8:2 | 0.587640984 | 8.819397143 | 15.00813829 | 3.907673121 | 0.004045 | 1 | Up |
| lnc-SPSB4-2:1 | 12.77133746 | 34.93026722 | 2.73505162 | 1.451568062 | 0.0352563 | 1 | Up |
| lnc-ST3GAL4-2:1 | 10.4585068 | 2.131045299 | 0.203761908 | -2.295043722 | 0.0297006 | 1 | Down |
| lnc-ST8SIA1-5:2 | 16.81706398 | 2.221271569 | 0.132084386 | -2.920468163 | 0.0067063 | 1 | Down |
| lnc-ST8SIA4-7:1 | 2.768028765 | 19.5354903 | 7.057545988 | 2.819166624 | 0.0221845 | 1 | Up |
| lnc-STX2-13:1 | 42.96861945 | 89.6287169 | 2.085911022 | 1.060677619 | 0.0119422 | 1 | Up |
| lnc-SUV420H1-2:1 | 0 | 4.746418374 | +∞ | +∞ | 0.0118077 | 1 | Up |
| lnc-SWI5-1:1 | 8.573764135 | 24.63822599 | 2.873676672 | 1.522897748 | 0.0268203 | 1 | Up |
| lnc-SYT11-1:2 | 0 | 5.359070472 | +∞ | +∞ | 0.0499849 | 1 | Up |
| lnc-SYT6-2:1 | 4.054517493 | 0.20448274 | 0.050433311 | -4.309479259 | 0.0357909 | 1 | Down |
| lnc-TAL1-3:2 | 3.02759073 | 13.16249137 | 4.347513435 | 2.120190486 | 0.0235061 | 1 | Up |
| lnc-TBC1D12-5:1 | 10.07984768 | 35.22126539 | 3.494225956 | 1.804972904 | 0.0136244 | 1 | Up |
| lnc-TBC1D20-1:1 | 7.918929175 | 22.01951215 | 2.780617387 | 1.475405243 | 0.0295421 | 1 | Up |
| lnc-TCF25-1:1 | 0 | 4.596792973 | +∞ | +∞ | 0.0229873 | 1 | Up |
| lnc-TEKT4-1:1 | 132.3074476 | 52.986361 | 0.400479051 | -1.320201319 | 0.0102791 | 1 | Down |
| lnc-TFEC-9:3 | 19.23917233 | 0.823638846 | 0.042810513 | -4.545891051 | 0.0054595 | 1 | Down |
| lnc-TGFBRAP1-1:3 | 38.66066584 | 16.41228971 | 0.424521652 | -1.236089959 | 0.0227271 | 1 | Down |
| lnc-THAP10-1:1 | 0 | 4.006756754 | +∞ | +∞ | 0.0438502 | 1 | Up |
| lnc-THAP11-1:1 | 18.50913267 | 6.150291007 | 0.332284128 | -1.589510713 | 0.0329613 | 1 | Down |
| lnc-THUMPD3-9:1 | 27.34724925 | 10.25967748 | 0.375163052 | -1.414410345 | 0.0285988 | 1 | Down |
| lnc-TMEM132D-1:6 | 0 | 4.042883756 | +∞ | +∞ | 0.0192345 | 1 | Up |
| lnc-TMEM179B-2:2 | 0 | 4.137710499 | +∞ | +∞ | 0.023853 | 1 | Up |
| lnc-TMEM50B-2:9 | 25.79311891 | 7.805548757 | 0.302621361 | -1.724414267 | 0.014132 | 1 | Down |
| lnc-TOMM20L-3:1 | 0.965742748 | 7.686735798 | 7.959403072 | 2.992660238 | 0.0328311 | 1 | Up |
| lnc-TP53RK-4:1 | 7.600675563 | 22.11851106 | 2.910071727 | 1.541054713 | 0.0301305 | 1 | Up |
| lnc-TPD52-3:12 | 0 | 6.15252082 | +∞ | +∞ | 0.0284536 | 1 | Up |
| lnc-TRAF3-1:1 | 0.801161885 | 6.50608674 | 8.120814112 | 3.021624365 | 0.0368578 | 1 | Up |
| lnc-TRHDE-2:1 | 38.38742172 | 85.23042994 | 2.220269716 | 1.150734944 | 0.0146614 | 1 | Up |
| lnc-TRIM26-2:29 | 31.33830437 | 13.51399473 | 0.43122929 | -1.213472924 | 0.0384259 | 1 | Down |
| lnc-TSEN34-1:1 | 21.42925786 | 4.722414179 | 0.220372269 | -2.181985404 | 0.0253317 | 1 | Down |
| lnc-TSTA3-4:1 | 3.187861671 | 12.18478501 | 3.822243959 | 1.934419863 | 0.0331133 | 1 | Up |
| lnc-TTC23-5:1 | 13.73940095 | 31.80635754 | 2.314974114 | 1.210996061 | 0.0360149 | 1 | Up |
| lnc-TTC24-2:1 | 4.944696143 | 15.40224428 | 3.114902075 | 1.639186809 | 0.0473799 | 1 | Up |
| lnc-TTC39A-3:1 | 51.29886666 | 17.17121647 | 0.334728964 | -1.578934704 | 0.0060594 | 1 | Down |
| lnc-TUBB2B-8:4 | 9.945418797 | 28.43529007 | 2.859134508 | 1.515578493 | 0.0261421 | 1 | Up |
| lnc-TULP2-2:1 | 0 | 22.94217122 | +∞ | +∞ | 0.0026616 | 1 | Up |
| lnc-TWIST1-1:3 | 1.900899377 | 12.87505288 | 6.773137515 | 2.759824287 | 0.0293455 | 1 | Up |
| lnc-TYMS-3:1 | 0.61614171 | 7.263209266 | 11.78821227 | 3.559273039 | 0.011668 | 1 | Up |
| lnc-U2AF1L4-1:9 | 0.19314855 | 9.626640751 | 49.84060594 | 5.639249703 | 0.0003812 | 0.8923423 | Up |
| lnc-U2AF1L4-2:1 | 59.51386299 | 27.69406967 | 0.465338129 | -1.10364869 | 0.0471723 | 1 | Down |
| lnc-UPK3A-2:2 | 0.965742748 | 16.41622883 | 16.99855252 | 4.087339997 | 0.0007695 | 1 | Up |
| lnc-USF2-1:1 | 2.14679964 | 12.17234725 | 5.669996876 | 2.50334794 | 0.0198297 | 1 | Up |
| lnc-USP33-2:2 | 36.69604299 | 14.79365366 | 0.403140297 | -1.310646096 | 0.0443438 | 1 | Down |
| lnc-USP6-2:6 | 9.754610739 | 0 | 0 | -∞ | 0.008296 | 1 | Down |
| lnc-UTP11L-6:1 | 0 | 4.962298683 | +∞ | +∞ | 0.0121773 | 1 | Up |
| lnc-VAMP3-4:1 | 4.760018833 | 0 | 0 | -∞ | 0.0336899 | 1 | Down |
| lnc-VASH2-1:1 | 6.65476927 | 22.37814141 | 3.362722357 | 1.749629668 | 0.0116561 | 1 | Up |
| lnc-VASN-1:1 | 5.775345193 | 22.38732714 | 3.876361741 | 1.954703209 | 0.0070355 | 1 | Up |
| lnc-VEZT-6:1 | 16.02102038 | 1.801744332 | 0.112461272 | -3.152499825 | 0.0011101 | 1 | Down |
| lnc-VPS37A-5:1 | 11.36710918 | 2.026556357 | 0.178282475 | -2.487763202 | 0.0131597 | 1 | Down |
| lnc-VSIG7-4:1 | 48.55324116 | 103.9738652 | 2.141440256 | 1.098581428 | 0.0320852 | 1 | Up |
| lnc-WDR19-1:1 | 0.788984868 | 6.048913281 | 7.666703798 | 2.938606443 | 0.0450868 | 1 | Up |
| lnc-WDR34-2:3 | 34.55138714 | 72.96415787 | 2.111757701 | 1.078444312 | 0.0191169 | 1 | Up |
| lnc-WDR7-6:2 | 3.096970079 | 22.39150706 | 7.230133482 | 2.854022282 | 0.0028093 | 1 | Up |
| lnc-WISP1-2:1 | 12.50375818 | 2.895439686 | 0.231565554 | -2.110507432 | 0.0343584 | 1 | Down |
| lnc-WRNIP1-28:1 | 5.367883563 | 17.40320034 | 3.242097213 | 1.69692735 | 0.0289963 | 1 | Up |
| lnc-WSB1-5:1 | 17.79833298 | 41.28868234 | 2.319806151 | 1.214004255 | 0.0251845 | 1 | Up |
| lnc-XPO4-1:2 | 3.967619269 | 0 | 0 | -∞ | 0.0296207 | 1 | Down |
| lnc-YDJC-1:2 | 10.17671814 | 0.137359649 | 0.013497441 | -6.211170316 | 0.0238116 | 1 | Down |
| lnc-YME1L1-1:1 | 10.01255737 | 0.907624552 | 0.090648624 | -3.463571063 | 0.0040137 | 1 | Down |
| lnc-ZBED4-1:2 | 1.807254137 | 9.386762886 | 5.193936312 | 2.376828324 | 0.0419605 | 1 | Up |
| lnc-ZBTB37-2:1 | 106.9820808 | 3.312567404 | 0.030963759 | -5.013275548 | 4.72E-20 | 1.88E-15 | Down |
| lnc-ZC3HC1-1:1 | 1.264452275 | 7.043005686 | 5.570005151 | 2.477678662 | 0.0456851 | 1 | Up |
| lnc-ZCCHC13-11:1 | 3.529145012 | 16.12152241 | 4.568109941 | 2.191597373 | 0.0092097 | 1 | Up |
| lnc-ZFAND4-1:1 | 0 | 10.02251454 | +∞ | +∞ | 0.019944 | 1 | Up |
| lnc-ZG16-2:1 | 27.72797209 | 61.25135359 | 2.209009494 | 1.143399619 | 0.0137505 | 1 | Up |
| lnc-ZHX3-4:4 | 0 | 3.328804044 | +∞ | +∞ | 0.0425499 | 1 | Up |
| lnc-ZMAT5-4:3 | 1.031073455 | 21.22560855 | 20.5859325 | 4.363586896 | 0.000114 | 0.412317 | Up |
| lnc-ZNF114-2:1 | 2.595693481 | 16.90932276 | 6.514375783 | 2.703626944 | 0.005452 | 1 | Up |
| lnc-ZNF192-1:1 | 1.640566478 | 9.526195456 | 5.806650072 | 2.537706095 | 0.0250879 | 1 | Up |
| lnc-ZNF212-2:2 | 0 | 32.11759985 | +∞ | +∞ | 1.14E-06 | 0.0113648 | Up |
| lnc-ZNF267-4:1 | 0.200537081 | 4.269665106 | 21.2911502 | 4.412181985 | 0.0460422 | 1 | Up |
| lnc-ZNF320-3:1 | 0.640562704 | 7.216059585 | 11.26518847 | 3.493799545 | 0.0124079 | 1 | Up |
| lnc-ZNF330-3:1 | 20.4767394 | 7.011337505 | 0.342404978 | -1.546224418 | 0.0221361 | 1 | Down |
| lnc-ZNF330-4:1 | 155.006905 | 77.25625304 | 0.498405236 | -1.004608871 | 0.0066407 | 1 | Down |
| lnc-ZNF330-5:1 | 38.06749662 | 16.6021522 | 0.436124087 | -1.197189424 | 0.01913 | 1 | Down |
| lnc-ZNF333-3:1 | 5.746356215 | 0.273681201 | 0.047626912 | -4.392079184 | 0.0159335 | 1 | Down |
| lnc-ZNF468-3:3 | 5.902018365 | 17.73137926 | 3.004290763 | 1.587024447 | 0.039268 | 1 | Up |
| lnc-ZNF570-3:1 | 1.797445196 | 13.92952966 | 7.749626912 | 2.954126857 | 0.00261 | 1 | Up |
| lnc-ZNF664-1:1 | 2.439673841 | 13.86736182 | 5.684104811 | 2.506933157 | 0.0090141 | 1 | Up |
| lnc-ZNF771-1:1 | 0.414057982 | 9.504579571 | 22.95470679 | 4.520718099 | 0.0076173 | 1 | Up |
| lnc-ZNF91-4:1 | 38.93475689 | 3.902100765 | 0.100221526 | -3.318735679 | 0.001184 | 1 | Down |
| lnc-ZNF91-4:2 | 0 | 19.81002291 | +∞ | +∞ | 0.0001401 | 0.4290045 | Up |
| lnc-ZNRF2-4:4 | 4.967025185 | 0 | 0 | -∞ | 0.0188914 | 1 | Down |
| lnc-ZRSR2-4:1 | 3.940658352 | 0 | 0 | -∞ | 0.0194088 | 1 | Down |
| lnc-ZSCAN2-5:16 | 18.06184244 | 55.63826109 | 3.080431095 | 1.623132265 | 0.0118786 | 1 | Up |

**Note:** PQDS: Pi-qi-deficiency sydrome; CSG: chronic superficial gastritis; BC: balanced constituion.

^a^ normalized mean count value;

^b^ normalized mean count value (PQDS of CSG) / normalized mean count value (BC);

^c^ *P*-value adjusted by false discovery rate (FDR).

**Table S7** Interactions between QDC-specific genes coded proteins

| **node1** | **node2** | **Homology ^a^** | **Co-expression ^b^** | **Experimentally determined interaction ^c^** | **Database annotated ^d^** | **Automated textmining ^e^** | **Combined score ^f^** |
| --- | --- | --- | --- | --- | --- | --- | --- |
| KIF2C | CCNB1 | 0 | 0.929 | 0.15 | 0.9 | 0.513 | 0.996 |
| ASPM | CCNB1 | 0 | 0.95 | 0.063 | 0 | 0.42 | 0.97 |
| HBD | HBG2 | 0.974 | 0.558 | 0 | 0.9 | 0.653 | 0.954 |
| SFN | CCNB1 | 0 | 0.059 | 0.19 | 0.9 | 0.441 | 0.951 |
| NEO1 | UNC5B | 0 | 0.096 | 0.409 | 0 | 0.9 | 0.942 |
| KIF2C | ASPM | 0 | 0.915 | 0.086 | 0 | 0.321 | 0.942 |
| GPSM1 | GRM2 | 0 | 0 | 0 | 0.9 | 0.387 | 0.936 |
| DNM1 | HIP1 | 0 | 0.115 | 0.158 | 0.9 | 0.222 | 0.934 |
| NDE1 | AHI1 | 0 | 0 | 0 | 0.9 | 0.298 | 0.926 |
| NDE1 | KIF2C | 0 | 0.062 | 0.08 | 0.9 | 0.176 | 0.919 |
| SPON1 | ADAMTS1 | 0 | 0.086 | 0 | 0.9 | 0.174 | 0.918 |
| KLRC1 | HLA-G | 0 | 0 | 0.328 | 0.6 | 0.706 | 0.914 |
| NDE1 | CCNB1 | 0 | 0.063 | 0 | 0.9 | 0.156 | 0.914 |
| MXRA8 | IGFBP3 | 0 | 0.127 | 0 | 0.9 | 0.099 | 0.914 |
| CLDN9 | CLDN22 | 0.827 | 0.06 | 0 | 0.9 | 0.607 | 0.911 |
| ITGAD | ITGA2 | 0.677 | 0 | 0 | 0.9 | 0.334 | 0.909 |
| CXCR6 | GPSM1 | 0 | 0 | 0 | 0.9 | 0 | 0.9 |
| CXCR6 | GRM2 | 0 | 0 | 0 | 0.9 | 0 | 0.9 |
| HBD | ALAS2 | 0 | 0.793 | 0 | 0 | 0.285 | 0.846 |
| KIF2C | RAD51AP1 | 0 | 0.778 | 0 | 0 | 0.252 | 0.827 |
| ASPM | RAD51AP1 | 0 | 0.827 | 0 | 0 | 0 | 0.827 |
| CCNB1 | RAD51AP1 | 0 | 0.772 | 0 | 0 | 0.195 | 0.809 |
| GZMK | KLRB1 | 0 | 0.722 | 0 | 0 | 0.253 | 0.783 |
| CLK2 | YTHDC1 | 0 | 0.097 | 0.731 | 0 | 0.083 | 0.757 |
| CXCR6 | GZMK | 0 | 0.632 | 0 | 0 | 0.326 | 0.742 |
| HBG2 | ALAS2 | 0 | 0.542 | 0 | 0 | 0.441 | 0.733 |
| KCNC3 | CACNA1A | 0 | 0.065 | 0.069 | 0 | 0.677 | 0.694 |
| SLC43A2 | SLC7A8 | 0 | 0.061 | 0 | 0 | 0.671 | 0.678 |
| DNM1 | PDE9A | 0 | 0.131 | 0 | 0 | 0.64 | 0.673 |
| AR | ELF3 | 0 | 0 | 0.397 | 0 | 0.471 | 0.668 |
| CXCR6 | KLRB1 | 0 | 0.32 | 0 | 0 | 0.529 | 0.666 |
| SPON1 | KCNC3 | 0 | 0.064 | 0 | 0 | 0.645 | 0.653 |
| DDR2 | ITGA2 | 0 | 0.05 | 0 | 0 | 0.647 | 0.65 |
| CLDN24 | CLDN9 | 0.835 | 0.06 | 0 | 0.6 | 0.528 | 0.64 |
| NDE1 | ASPM | 0 | 0.064 | 0 | 0 | 0.607 | 0.617 |
| GRIK4 | SHANK1 | 0 | 0.109 | 0.127 | 0.36 | 0.322 | 0.617 |
| CLDN24 | CLDN22 | 0.982 | 0 | 0 | 0.6 | 0 | 0.6 |
| KLRC1 | PVRL2 | 0 | 0 | 0 | 0 | 0.587 | 0.587 |
| GRIK4 | GRM2 | 0 | 0.106 | 0.06 | 0 | 0.546 | 0.585 |
| REC8 | CCNB1 | 0 | 0.063 | 0.194 | 0.36 | 0.217 | 0.57 |
| VARS2 | CNIH2 | 0 | 0 | 0 | 0 | 0.56 | 0.56 |
| DDX39B | SLC7A9 | 0 | 0 | 0.552 | 0 | 0 | 0.552 |
| PUF60 | DDX39B | 0 | 0.316 | 0 | 0 | 0.351 | 0.538 |
| KCNC3 | KCNT1 | 0 | 0.142 | 0 | 0 | 0.455 | 0.513 |
| IGFBP3 | AR | 0 | 0 | 0.107 | 0 | 0.453 | 0.49 |
| CNIH2 | SHANK1 | 0 | 0.123 | 0 | 0.36 | 0.159 | 0.487 |
| CXCR6 | KLRG1 | 0 | 0.061 | 0 | 0 | 0.473 | 0.483 |
| KLRG1 | GZMK | 0 | 0.135 | 0 | 0 | 0.417 | 0.474 |
| KCNT1 | CACNA1A | 0 | 0.231 | 0 | 0 | 0.338 | 0.469 |
| TRPC3 | RASA4 | 0 | 0 | 0 | 0 | 0.468 | 0.468 |
| ITGA2 | KLRB1 | 0 | 0 | 0 | 0 | 0.467 | 0.467 |
| GRIK4 | CNIH2 | 0 | 0.13 | 0.084 | 0.36 | 0.075 | 0.465 |
| PDZD2 | FAT4 | 0 | 0 | 0.421 | 0 | 0.105 | 0.459 |
| CATSPERG | CACNA1A | 0 | 0.065 | 0.121 | 0.36 | 0.092 | 0.458 |
| DDR2 | PDGFRA | 0.585 | 0.312 | 0 | 0 | 0.527 | 0.457 |
| KLRG1 | ITGA2 | 0 | 0 | 0 | 0 | 0.456 | 0.456 |
| KLRC1 | CXCR6 | 0 | 0.095 | 0 | 0 | 0.413 | 0.447 |
| MOK | CCNB1 | 0 | 0.049 | 0.408 | 0 | 0.097 | 0.447 |
| DDX39B | TAF4 | 0 | 0.433 | 0 | 0 | 0.057 | 0.443 |
| PPP1R10 | HIST1H3J | 0 | 0 | 0 | 0 | 0.442 | 0.443 |
| VARS2 | PPP1R10 | 0 | 0.063 | 0 | 0 | 0.419 | 0.433 |
| CSF2RA | GTPBP6 | 0 | 0 | 0 | 0 | 0.421 | 0.42 |
| PDGFRA | CADM4 | 0 | 0.055 | 0.412 | 0 | 0 | 0.42 |
| REC8 | ATF6B | 0 | 0.421 | 0 | 0 | 0 | 0.42 |
| ZMYND8 | SHANK1 | 0 | 0.061 | 0 | 0 | 0.405 | 0.418 |
| VARS2 | ATF6B | 0 | 0.062 | 0 | 0 | 0.404 | 0.417 |
| AR | CCNB1 | 0 | 0 | 0.05 | 0 | 0.408 | 0.413 |
| AHI1 | CNOT3 | 0 | 0.063 | 0 | 0 | 0.397 | 0.41 |
| ATF6B | PBX2 | 0 | 0.061 | 0 | 0 | 0.398 | 0.41 |
| KLRC1 | GZMK | 0 | 0.055 | 0 | 0 | 0.4 | 0.409 |
| OLR1 | TLR3 | 0 | 0 | 0.06 | 0 | 0.398 | 0.409 |
| KCNT1 | SHANK1 | 0 | 0.227 | 0 | 0 | 0.264 | 0.407 |
| FAM127B | NUDT11 | 0 | 0 | 0 | 0 | 0.405 | 0.405 |

**Note:** QDC: qi deficiency constitution.

**^a^** Cooccurrence, Gene families whose occurrence patterns across genomes show similarities;

**^b^** Coexpression, Proteins whose genes are observed to be correlated in expression, across a large number of experiments;

**^c^** Experiments, Co-purification, co-crystallization, Yeast2Hybrid, Genetic Interactions, etc ... as imported from primary sources;

**^d^** Database, Known metabolic pathways, protein complexes, signal transduction pathways, etc ... from curated databases;

**^e^** Textmining, Automated, unsupervised textmining - searching for proteins that are frequently mentioned together;

^f^ The value determine the thickness of edge between nudes in a created interaction network cartoon, indicating the interaction strength of all the support data.

**Table S8 The QDC-specific differential genes regulated by QDC-specific differential *trans*-acting lncRNAs order by genes**

| **Targets ^a^** | **Count** | ***Trans*-acting lncRNAs ^a^** |
| --- | --- | --- |
| CNOT3 | 114 | DANT1:2,FTX:1,GAS6-AS2:1,LBX2-AS1:2,LINC00654:4,LINC00847:14,LINC00887:5,LINC01426:3,lnc-ABCC6-2:1,lnc-ABHD2-4:1,lnc-AC006455.1-10:1,lnc-AC091132.2-7:1,lnc-ACR-2:5,lnc-ADA-2:1,lnc-AMZ2-3:1,lnc-ANKRD30B-7:2,lnc-APBA2-5:8,lnc-B3GNT9-1:1,lnc-BCL9L-1:1,lnc-C10orf90-2:2,lnc-C11orf94-1:1,lnc-C14orf183-1:13,lnc-C14orf79-3:1,lnc-C15orf39-1:1,lnc-C17orf109-3:1,lnc-C1orf138-1:1,lnc-C20orf24-3:1,lnc-C2orf89-2:2,lnc-CAMK1-1:2,lnc-CARKD-4:1,lnc-CCT5-2:1,lnc-CEP170-9:2,lnc-CLCN1-2:1,lnc-CLDN23-3:1,lnc-CLIC3-2:1,lnc-COX19-2:2,lnc-CRYBA4-1:55,lnc-ELF2-1:1,lnc-EXOC2-8:4,lnc-FAM160A1-8:1,lnc-FTSJ1-2:1,lnc-GTPBP1-1:1,lnc-HEATR2-3:1,lnc-HTRA2-1:1,lnc-IFT52-1:2,lnc-IRAK1-1:1,lnc-KIAA0355-5:1,lnc-KLC2-2:1,lnc-KLF14-1:1,lnc-KLHL5-2:1,lnc-LAT-1:9,lnc-MAPK6-4:2,lnc-MOGAT2-3:1,lnc-MTIF2-1:1,lnc-NKAIN3-1:2,lnc-NRARP-3:1,lnc-NT5C-3:3,lnc-OLFML3-5:1,lnc-PCBP3-3:9,lnc-PIN4-1:1,lnc-POFUT2-5:1,lnc-POTEF-8:2,lnc-PRAGMIN.1-3:2,lnc-PRDM11-9:2,lnc-PTGER1-1:2,lnc-PTPN12-1:1,lnc-PUF60-1:1,lnc-RABGEF1-1:1,lnc-RAD52-2:5,lnc-RAP1GAP2-3:1,lnc-RBAK-2:10,lnc-RBPMS2-1:2,lnc-RCN1-2:1,lnc-ROPN1L-3:1,lnc-RP3-369A17.5.1-3:1,lnc-RPL23A-2:1,lnc-RUNDC3A-4:2,lnc-SAMD8-1:1,lnc-SBDS-4:1,lnc-SEC11A-1:3,lnc-SEC11A-2:1,lnc-SH2B2-7:1,lnc-SIX5-1:1,lnc-SLC9A7-1:2,lnc-SNURF-1:35,lnc-SP6-1:1,lnc-TAF13-2:1,lnc-TAOK3-1:11,lnc-TNNT1-1:1,lnc-TTR-3:1,lnc-TYSND1-1:7,lnc-UGCG-1:3,lnc-WDR73-7:2,lnc-ZNF587-1:1,lnc-ZSWIM7-3:1,PART1:10,PCBP1-AS1:181,PRKCQ-AS1:16,PSMB8-AS1:1,PSMB8-AS1:3,PSMB8-AS1:5,SMIM2-AS1:11,SNHG4:3,ST3GAL5-AS1:4,TCL6:1,TCONS_00000037,TCONS_00046326,TCONS_00049141,TCONS_00052323,TCONS_00052658,TCONS_00052860,THUMPD3-AS1:27,TRAF3IP2-AS1:24,ZNF528-AS1:3 |
| SYT15 | 91 | CHKB-AS1:14,DANT1:2,FTX:1,GAS6-AS2:1,INHBA-AS1:2,LBX2-AS1:2,LINC00847:14,LINC00887:5,LINC00893:4,LINC01299:2,LINC01426:3,LINC01550:2,lnc-ABCC6-2:1,lnc-ABHD2-4:1,lnc-AC006455.1-10:1,lnc-AC069257.9.1-5:5,lnc-AC091132.2-7:1,lnc-ADA-2:1,lnc-AMZ2-3:1,lnc-APBA2-5:8,lnc-BCL9L-1:1,lnc-C11orf94-1:1,lnc-C14orf79-3:1,lnc-C3orf62-2:1,lnc-CCT5-2:1,lnc-CEP170-9:2,lnc-CLCN1-2:1,lnc-CLDN23-3:1,lnc-CLIC3-2:1,lnc-COX19-2:2,lnc-CRYBA4-1:55,lnc-DDX52-3:1,lnc-ELF2-1:1,lnc-EXOC2-8:4,lnc-EXT1-1:1,lnc-FAM160A1-8:1,lnc-FRYL-3:3,lnc-FTSJ1-2:1,lnc-GTPBP1-1:1,lnc-HEATR2-3:1,lnc-HSPG2-2:1,lnc-HTRA2-1:1,lnc-IRAK1-1:1,lnc-KIAA0355-5:1,lnc-KLF13-2:4,lnc-KLHL5-2:1,lnc-LIPI-9:1,lnc-MAPK6-4:2,lnc-MOGAT2-3:1,lnc-MTR-4:1,lnc-NKAIN3-1:2,lnc-NRARP-3:1,lnc-NT5C-3:3,lnc-OLFML3-5:1,lnc-OPRD1-4:1,lnc-PAIP2-1:1,lnc-PCBP3-3:9,lnc-PRAGMIN.1-3:2,lnc-PTGER1-1:2,lnc-PTPN12-1:1,lnc-PUF60-1:1,lnc-RABGEF1-1:1,lnc-RAD52-2:5,lnc-RBAK-2:10,lnc-RCN1-2:1,lnc-RPL23A-2:1,lnc-RUNDC3A-4:2,lnc-SAMD8-1:1,lnc-SBDS-4:1,lnc-SEC11A-1:3,lnc-SEC11A-2:1,lnc-SIX5-1:1,lnc-TAF13-2:1,lnc-TAOK3-1:11,lnc-TOR3A-1:1,lnc-TTR-3:1,lnc-TYSND1-1:7,lnc-UGCG-1:3,lnc-WDR73-7:2,lnc-ZKSCAN3-1:1,PART1:10,PSMB8-AS1:1,PSMB8-AS1:5,SLC25A25-AS1:15,SMIM2-AS1:11,SNHG4:3,ST3GAL5-AS1:4,TCONS_00049141,TCONS_00052860,UBA6-AS1:1,ZNF528-AS1:3 |
| SHANK1 | 90 | CHKB-AS1:14,DANT1:2,FTX:1,GAS6-AS2:1,LBX2-AS1:2,LINC00847:14,LINC00861:6,LINC00887:5,LINC00893:4,LINC01299:2,LINC01426:3,lnc-ABCC6-2:1,lnc-ABHD2-4:1,lnc-AC006455.1-10:1,lnc-AC091132.2-7:1,lnc-AC107021.1-7:1,lnc-ACR-2:5,lnc-ADA-2:1,lnc-AMZ2-3:1,lnc-ANKRD30B-7:2,lnc-APBA2-5:8,lnc-BCL9L-1:1,lnc-C14orf79-3:1,lnc-C20orf24-3:1,lnc-C2orf89-2:2,lnc-CARKD-4:1,lnc-CLCN1-2:1,lnc-CLDN23-3:1,lnc-CLIC3-2:1,lnc-CRYBA4-1:34,lnc-ELF2-1:1,lnc-EXT1-1:1,lnc-FAM160A1-8:1,lnc-FRYL-3:3,lnc-FXYD4-4:2,lnc-HEATR2-3:1,lnc-HSPG2-2:1,lnc-HTRA2-1:1,lnc-IRAK1-1:1,lnc-KLF13-2:4,lnc-KLF14-1:1,lnc-KLHL5-2:1,lnc-LIPI-9:1,lnc-MAPK6-4:2,lnc-MOGAT2-3:1,lnc-MTR-4:1,lnc-NDUFB8-7:1,lnc-NRARP-3:1,lnc-NT5C-3:3,lnc-OLFML3-5:1,lnc-OPRD1-4:1,lnc-PAIP2-1:1,lnc-PCBP3-3:9,lnc-PIN4-1:1,lnc-PKD2L1-5:1,lnc-POTEF-8:2,lnc-PRAGMIN.1-3:2,lnc-PRDM11-9:2,lnc-PTPN12-1:1,lnc-PUF60-1:1,lnc-RAD52-2:5,lnc-RBAK-2:10,lnc-RBPMS2-1:2,lnc-RCN1-2:1,lnc-ROPN1L-3:1,lnc-RPL23A-2:1,lnc-RUNDC3A-4:2,lnc-SBDS-4:1,lnc-SEC11A-2:1,lnc-SIX5-1:1,lnc-TACR2-6:1,lnc-TAOK3-1:11,lnc-TOR3A-1:1,lnc-TYSND1-1:7,lnc-UGCG-1:3,lnc-WDR73-7:2,lnc-ZNF827-11:1,MIR4435-2HG:9,PART1:10,PCF11-AS1:2,PSMB8-AS1:1,PSMB8-AS1:3,PSMB8-AS1:5,SMIM2-AS1:11,SNHG4:3,ST3GAL5-AS1:4,TCONS_00052658,TCONS_00052860,UBA6-AS1:1,ZNF528-AS1:3 |
| KCNT1 | 83 | CHKB-AS1:14,DANT1:2,FTX:1,GAS6-AS2:1,LBX2-AS1:2,LINC00861:6,LINC00887:5,lnc-ABCC6-2:1,lnc-ABHD2-4:1,lnc-AC006455.1-10:1,lnc-AC091132.2-7:1,lnc-AC107021.1-7:1,lnc-ACR-2:5,lnc-AMZ2-3:1,lnc-ANKRD30B-7:2,lnc-APBA2-5:8,lnc-BCL9L-1:1,lnc-C11orf94-1:1,lnc-C14orf79-3:1,lnc-C17orf109-3:1,lnc-C3orf62-2:1,lnc-CAMK1-1:2,lnc-CARKD-4:1,lnc-CEP170-9:2,lnc-CLCN1-2:1,lnc-CLDN23-3:1,lnc-CLIC3-2:1,lnc-COX19-2:2,lnc-CRYBA4-1:55,lnc-CSNK1D-2:9,lnc-DDX52-3:1,lnc-DPY19L3-1:1,lnc-EFNB2-6:1,lnc-EXT1-1:1,lnc-FAM160A1-8:1,lnc-FRYL-3:3,lnc-GRID2-1:1,lnc-HEATR2-3:1,lnc-HTRA2-1:1,lnc-IFT52-1:2,lnc-KIAA0355-5:1,lnc-LAT-1:9,lnc-MOGAT2-3:1,lnc-MTR-4:1,lnc-NRARP-3:1,lnc-NT5C-3:3,lnc-PAIP2-1:1,lnc-PCBP3-3:9,lnc-PRAGMIN.1-3:2,lnc-PRDM11-9:2,lnc-PTPN12-1:1,lnc-PUF60-1:1,lnc-RABGEF1-1:1,lnc-RBAK-2:10,lnc-RCN1-2:1,lnc-ROPN1L-3:1,lnc-RPL23A-2:1,lnc-RUNDC3A-4:2,lnc-SAG-7:2,lnc-SAMD8-1:1,lnc-SBDS-4:1,lnc-SEC11A-1:3,lnc-SEC11A-2:1,lnc-SIX5-1:1,lnc-TAF13-2:1,lnc-TOR3A-1:1,lnc-TTR-3:1,lnc-TYSND1-1:7,lnc-UGCG-1:3,lnc-WDR73-7:2,lnc-ZNF827-11:1,PART1:10,PSMB8-AS1:1,PSMB8-AS1:3,PSMB8-AS1:5,SMIM2-AS1:11,SNHG4:3,ST3GAL5-AS1:4,TCONS_00049141,TCONS_00052860,TRAF3IP2-AS1:24,UBA6-AS1:1,ZNF528-AS1:3 |
| LENG8 | 78 | CHKB-AS1:14,DANT1:2,FTX:1,GAS6-AS2:1,LINC00847:14,LINC00887:5,LINC00893:4,LINC01299:2,LINC01426:3,LINC01550:2,lnc-ABCC6-2:1,lnc-ABHD2-4:1,lnc-AC091132.2-7:1,lnc-AC107021.1-7:1,lnc-ACR-2:5,lnc-ADA-2:1,lnc-AKAP12-1:2,lnc-AMZ2-3:1,lnc-APBA2-5:8,lnc-BCL9L-1:1,lnc-C14orf79-3:1,lnc-C20orf24-3:1,lnc-C3orf62-2:1,lnc-CARKD-4:1,lnc-CLCN1-2:1,lnc-CLDN23-3:1,lnc-CLIC3-2:1,lnc-EFNB2-6:1,lnc-EXOC2-8:4,lnc-EXT1-1:1,lnc-FAM160A1-8:1,lnc-FRYL-3:3,lnc-HEATR2-3:1,lnc-HSPG2-2:1,lnc-HTRA2-1:1,lnc-KLF13-2:4,lnc-LIPI-9:1,lnc-MAPK6-4:2,lnc-MOGAT2-3:1,lnc-MTR-4:1,lnc-NRARP-3:1,lnc-NT5C-3:3,lnc-OLFML3-5:1,lnc-OPRD1-4:1,lnc-PAIP2-1:1,lnc-PCBP3-3:9,lnc-PIN4-1:1,lnc-PKD2L1-5:1,lnc-POTEF-8:2,lnc-PRAGMIN.1-3:2,lnc-PRDM11-9:2,lnc-PTPN12-1:1,lnc-PUF60-1:1,lnc-RAD52-2:5,lnc-RBAK-2:10,lnc-RBPMS2-1:2,lnc-RCN1-2:1,lnc-ROPN1L-3:1,lnc-RPL23A-2:1,lnc-RUNDC3A-4:2,lnc-SEC11A-1:3,lnc-SEC11A-2:1,lnc-SIX5-1:1,lnc-TAOK3-1:11,lnc-TOR3A-1:1,lnc-TYSND1-1:7,lnc-UGCG-1:3,lnc-WDR73-7:2,lnc-ZKSCAN3-1:1,lnc-ZNF827-11:1,PART1:10,PSMB8-AS1:1,PSMB8-AS1:5,SMIM2-AS1:11,ST3GAL5-AS1:4,TCONS_00049141,TCONS_00052860,ZNF528-AS1:3 |
| SPEG | 75 | CHKB-AS1:14,DANT1:2,LBX2-AS1:2,LINC00649:8,LINC00654:4,LINC00893:4,LINC01426:3,lnc-AC006455.1-10:1,lnc-AMZ2-3:1,lnc-B3GNT9-1:1,lnc-C10orf90-2:2,lnc-C11orf94-1:1,lnc-C14orf183-1:13,lnc-C14orf79-3:1,lnc-C17orf109-3:1,lnc-C17orf77-1:6,lnc-C20orf24-3:1,lnc-C3orf62-2:1,lnc-CAMK1-1:2,lnc-CDH9-3:1,lnc-CLDN23-3:1,lnc-CLDN9-1:1,lnc-CLIC3-2:1,lnc-COX19-2:2,lnc-CRYBA4-1:34,lnc-CRYBA4-1:55,lnc-EXOC2-8:4,lnc-EXT1-1:1,lnc-GNA14-3:1,lnc-HEATR2-3:1,lnc-KLC2-2:1,lnc-LAT-1:9,lnc-MAPK6-4:2,lnc-MOGAT2-3:1,lnc-MTIF2-1:1,lnc-MTR-4:1,lnc-NKAIN3-1:2,lnc-NRARP-3:1,lnc-OLFML3-5:1,lnc-PAIP2-1:1,lnc-PCBP3-3:9,lnc-PRAGMIN.1-3:2,lnc-PTGER1-1:2,lnc-PUF60-1:1,lnc-RABGEF1-1:1,lnc-RAP1GAP2-3:1,lnc-RCN1-2:1,lnc-ROPN1L-3:1,lnc-RPL23A-2:1,lnc-RUNDC3A-4:2,lnc-SBDS-4:1,lnc-SEC11A-1:3,lnc-SEC11A-2:1,lnc-SERPINB6-1:7,lnc-SIX5-1:1,lnc-SLC9A7-1:2,lnc-TAF13-2:1,lnc-TOR3A-1:1,lnc-TRPM7-1:1,lnc-TSSC4-5:2,lnc-TYSND1-1:7,lnc-UGCG-1:3,lnc-ZSCAN10-3:17,lnc-ZSWIM7-3:1,PCF11-AS1:2,SMIM2-AS1:11,SNAI3-AS1:16,SNHG4:3,TCL6:1,TCONS_00000037,TCONS_00025364,TCONS_00046326,TCONS_00052860,TRAF3IP2-AS1:24,ZNF528-AS1:3 |
| CACNA1A | 75 | APTR:11,CHKB-AS1:14,FAM201A:1,LBX2-AS1:2,LINC00649:8,LINC00861:6,LINC00893:4,LINC01426:3,lnc-ABHD2-4:1,lnc-AC006455.1-10:1,lnc-AC107021.1-7:1,lnc-ACSBG2-2:1,lnc-AP000769.1-1:2,lnc-BRI3-2:3,lnc-C10orf90-2:2,lnc-C11orf94-1:1,lnc-C14orf79-3:1,lnc-C16orf72-7:1,lnc-C17orf109-3:1,lnc-C17orf77-1:6,lnc-C1orf138-1:1,lnc-C1orf186-3:1,lnc-CAMK1-1:2,lnc-CLDN23-3:1,lnc-CLDN9-1:1,lnc-CLIC3-2:1,lnc-COX19-2:2,lnc-CRYBA4-1:34,lnc-CRYBA4-1:55,lnc-CSNK1D-2:9,lnc-DPY19L3-1:1,lnc-EFNB2-6:1,lnc-FAM156B-1:1,lnc-GAS8-1:4,lnc-GRID2-1:1,lnc-HEATR2-3:1,lnc-LAT-1:9,lnc-LONRF2-3:1,lnc-MAP3K3-2:2,lnc-MTA2-4:1,lnc-MTIF2-1:1,lnc-OLFML3-5:1,lnc-PAIP2-1:1,lnc-POFUT2-4:1,lnc-PRAGMIN.1-3:2,lnc-PRKRIR-1:5,lnc-PTPN12-1:1,lnc-PUF60-1:1,lnc-RBAK-2:10,lnc-ROPN1L-10:5,lnc-RP3-377D14.1.1-7:10,lnc-RUNDC3A-4:2,lnc-SAG-7:2,lnc-SATB1-8:5,lnc-SBDS-4:1,lnc-SEC11A-2:1,lnc-SH3BGR-2:1,lnc-SIX5-1:1,lnc-SLC9A7-1:2,lnc-SNAPC5-3:2,lnc-TAF13-2:1,lnc-TAGLN2-1:1,lnc-TMEM71-3:6,lnc-TNFRSF1A-3:1,lnc-TSSC4-5:2,lnc-ZNF587-1:1,NDUFA6-AS1:5,PCBP1-AS1:169,PCF11-AS1:2,SNAI3-AS1:16,SNHG4:3,TCONS_00052860,TRAF3IP2-AS1:24,UBA6-AS1:1,ZNF528-AS1:3 |
| SLC9A1 | 72 | APTR:11,CARD8-AS1:4,FAM201A:1,FAM74A6:1,HOXA-AS2:2,LINC00476:3,LINC00649:8,LINC00894:20,lnc-AIF1-2:1,lnc-AL669831.1-3:42,lnc-APH1A-1:6,lnc-ARL13B-1:5,lnc-BCL2L2-PABPN1-1:2,lnc-BRI3-2:3,lnc-C10orf90-2:2,lnc-C11orf94-1:1,lnc-C15orf39-1:1,lnc-C16orf72-7:1,lnc-C17orf109-3:1,lnc-C17orf62-6:6,lnc-C1orf138-1:1,lnc-C1orf186-3:1,lnc-C4orf36-2:5,lnc-CAMK1-1:2,lnc-CLDN9-1:1,lnc-COX19-2:2,lnc-CRYBA4-1:55,lnc-CTU2-2:1,lnc-DOC2B-3:2,lnc-DPY19L3-1:1,lnc-FAM200B-1:13,lnc-FLRT2-2:10,lnc-FTSJ1-2:1,lnc-HTRA4-2:1,lnc-IL32-1:3,lnc-KLF14-1:6,lnc-L3MBTL4-4:1,lnc-LAG3-1:1,lnc-METRNL-7:4,lnc-MTA2-4:1,lnc-NCDN-2:1,lnc-NDRG1-1:4,lnc-OR2AT4-1:1,lnc-PCID2-1:2,lnc-PHLDA3-2:1,lnc-PLCG2-5:1,lnc-PLEKHG5-3:1,lnc-POFUT2-4:1,lnc-POFUT2-5:1,lnc-PRKRIR-1:5,lnc-RIPK1-1:2,lnc-SATB1-8:5,lnc-SH3BGR-2:1,lnc-SLC12A8-1:1,lnc-SLC26A11-1:1,lnc-SLC7A6OS-4:2,lnc-SLC9A7-1:2,lnc-STAB1-3:1,lnc-SYNDIG1L-2:3,lnc-TACC2-3:3,lnc-TAF13-2:1,lnc-TMEM120B-4:3,lnc-TNFRSF1A-3:1,lnc-TNNT1-1:1,lnc-VTI1A-1:3,lnc-WDR38-1:1,lnc-ZNF587-1:1,LOH12CR2:1,RGPD4-AS1:6,TCONS_00044060,THUMPD3-AS1:5,TRAF3IP2-AS1:24 |
| OBSCN | 69 | DANT1:2,GAS6-AS2:1,LINC00654:4,LINC00893:4,LINC01426:3,lnc-AC006455.1-10:1,lnc-AC107021.1-7:1,lnc-ADA-2:1,lnc-AMZ2-3:1,lnc-B3GNT9-1:1,lnc-BCL9L-1:1,lnc-C14orf183-1:13,lnc-C14orf79-3:1,lnc-C17orf109-3:1,lnc-C1orf138-1:1,lnc-C3orf62-2:1,lnc-CCT5-2:1,lnc-CRYBA4-1:34,lnc-CRYBA4-1:55,lnc-DDX52-3:1,lnc-EXOC2-8:4,lnc-EXT1-1:1,lnc-KIAA0355-5:1,lnc-KLC2-2:1,lnc-KLHL5-2:1,lnc-LIPI-9:1,lnc-LPCAT1-3:2,lnc-MTIF2-1:1,lnc-NKAIN3-1:2,lnc-PAIP2-1:1,lnc-PCBP3-3:9,lnc-PRAGMIN.1-3:2,lnc-PTGER1-1:2,lnc-PTPN12-1:1,lnc-PUF60-1:1,lnc-RABGEF1-1:1,lnc-RCN1-2:1,lnc-RP3-369A17.5.1-3:1,lnc-RP3-377D14.1.1-7:10,lnc-RPL23A-2:1,lnc-RUNDC3A-4:2,lnc-SAMD8-1:1,lnc-SEC11A-1:3,lnc-SEC11A-2:1,lnc-SERPINB6-1:7,lnc-SFPQ-2:4,lnc-SH2B2-7:1,lnc-SP6-1:1,lnc-TAF13-2:1,lnc-TMOD3-3:7,lnc-TYSND1-1:7,lnc-UGCG-1:3,lnc-ZNF587-1:1,NEAT1:14,PCBP1-AS1:169,PCBP1-AS1:181,PCF11-AS1:2,PSMB8-AS1:1,PSMB8-AS1:3,SLC25A25-AS1:15,SNHG4:3,TCL6:1,TCONS_00000037,TCONS_00025364,TCONS_00031339,TCONS_00046326,TCONS_00049141,XIST:20,XIST:6 |
| TAF4 | 68 | DANT1:2,FTX:1,GAS6-AS2:1,LBX2-AS1:2,LINC00847:14,LINC00887:5,LINC01299:2,lnc-ABCC6-2:1,lnc-AC091132.2-7:1,lnc-AC107021.1-7:1,lnc-ACR-2:5,lnc-ADA-2:1,lnc-AMZ2-3:1,lnc-ANKRD30B-7:2,lnc-APBA2-5:8,lnc-BCL9L-1:1,lnc-C14orf79-3:1,lnc-C20orf24-3:1,lnc-C3orf62-2:1,lnc-CARKD-4:1,lnc-CLCN1-2:1,lnc-CLDN23-3:1,lnc-CLIC3-2:1,lnc-EFNB2-6:1,lnc-EXOC2-8:4,lnc-EXT1-1:1,lnc-FAM160A1-8:1,lnc-FRYL-3:3,lnc-GTPBP1-1:1,lnc-HEATR2-3:1,lnc-HTRA2-1:1,lnc-KLF13-2:4,lnc-KLF14-1:1,lnc-KLHL5-2:1,lnc-LIPI-9:1,lnc-MAPK6-4:2,lnc-MOGAT2-3:1,lnc-NRARP-3:1,lnc-NT5C-3:3,lnc-OLFML3-5:1,lnc-PAIP2-1:1,lnc-PCBP3-3:9,lnc-PIN4-1:1,lnc-POTEF-8:2,lnc-PRDM11-9:2,lnc-PTPN12-1:1,lnc-PUF60-1:1,lnc-RBAK-2:10,lnc-RBPMS2-1:2,lnc-RCN1-2:1,lnc-ROPN1L-3:1,lnc-RUNDC3A-4:2,lnc-SEC11A-2:1,lnc-SIX5-1:1,lnc-TAOK3-1:11,lnc-TOR3A-1:1,lnc-TYSND1-1:7,lnc-WDR73-7:2,lnc-ZKSCAN3-1:1,PART1:10,PCF11-AS1:2,PSMB8-AS1:1,PSMB8-AS1:5,SNHG4:3,ST3GAL5-AS1:4,TCONS_00049141,TCONS_00052658,TCONS_00052860 |
| TMC8 | 67 | DANT1:2,FTX:1,GAS6-AS2:1,LINC00847:14,LINC00887:5,LINC01299:2,lnc-ABCC6-2:1,lnc-AC091132.2-7:1,lnc-ACR-2:5,lnc-ADA-2:1,lnc-AMZ2-3:1,lnc-ANKRD30B-7:2,lnc-APBA2-5:8,lnc-BCL9L-1:1,lnc-C14orf79-3:1,lnc-C20orf24-3:1,lnc-C3orf62-2:1,lnc-CARKD-4:1,lnc-CCT5-2:1,lnc-CLCN1-2:1,lnc-CLDN23-3:1,lnc-CRYBA4-1:34,lnc-DDX52-3:1,lnc-EFNB2-6:1,lnc-EXOC2-8:4,lnc-EXT1-1:1,lnc-FAM160A1-8:1,lnc-FRYL-3:3,lnc-HSPG2-2:1,lnc-HTRA2-1:1,lnc-KIAA0355-5:1,lnc-KLF13-2:4,lnc-KLHL5-2:1,lnc-LIPI-9:1,lnc-MAPK6-4:2,lnc-MOGAT2-3:1,lnc-NRARP-3:1,lnc-NT5C-3:3,lnc-PAIP2-1:1,lnc-PCBP3-3:9,lnc-POTEF-8:2,lnc-PRDM11-9:2,lnc-PTGER1-1:2,lnc-PTPN12-1:1,lnc-RAD52-2:5,lnc-RBAK-2:10,lnc-RBPMS2-1:2,lnc-ROPN1L-3:1,lnc-SEC11A-1:3,lnc-SEC11A-2:1,lnc-SP6-1:1,lnc-TACR2-6:1,lnc-TAOK3-1:11,lnc-TOR3A-1:1,lnc-TYSND1-1:7,lnc-UGCG-1:3,lnc-WDR73-7:2,lnc-ZKSCAN3-1:1,lnc-ZNF827-11:1,PART1:10,PSMB8-AS1:1,PSMB8-AS1:5,ST3GAL5-AS1:4,TCONS_00031339,TCONS_00049141,TCONS_00052658,TCONS_00052860 |
| GRIK4 | 66 | DANT1:2,GAS6-AS2:1,LBX2-AS1:2,LINC00654:4,LINC00847:14,LINC00887:5,LINC00893:4,lnc-ABCC6-2:1,lnc-ABHD2-4:1,lnc-AC091132.2-7:1,lnc-ACR-2:5,lnc-ADA-2:1,lnc-AMZ2-3:1,lnc-ANKRD30B-7:2,lnc-APBA2-5:8,lnc-C11orf94-1:1,lnc-C14orf79-3:1,lnc-C20orf24-3:1,lnc-CARKD-4:1,lnc-CEP170-9:2,lnc-CLCN1-2:1,lnc-CLDN23-3:1,lnc-CLIC3-2:1,lnc-COX19-2:2,lnc-CRYBA4-1:34,lnc-EXOC2-8:4,lnc-EXT1-1:1,lnc-FAM160A1-8:1,lnc-FRYL-3:3,lnc-FXYD4-4:2,lnc-GTPBP1-1:1,lnc-HEATR2-3:1,lnc-HSPG2-2:1,lnc-HTRA2-1:1,lnc-KLF13-2:4,lnc-MAPK6-4:2,lnc-MOGAT2-3:1,lnc-NRARP-3:1,lnc-NT5C-3:3,lnc-PCBP3-3:9,lnc-POTEF-8:2,lnc-PRAGMIN.1-3:2,lnc-PRDM11-9:2,lnc-PTGER1-1:2,lnc-PUF60-1:1,lnc-QRICH2-1:1,lnc-RBAK-2:10,lnc-ROPN1L-3:1,lnc-RPL23A-2:1,lnc-RUNDC3A-4:2,lnc-SBDS-4:1,lnc-SEC11A-2:1,lnc-SIX5-1:1,lnc-TAOK3-1:11,lnc-TOR3A-1:1,lnc-TTR-3:1,lnc-TYSND1-1:7,lnc-WDR73-7:2,PART1:10,PSMB8-AS1:1,PSMB8-AS1:5,SNHG4:3,ST3GAL5-AS1:4,TCONS_00052860,UBA6-AS1:1,ZNF528-AS1:3 |
| FOSB | 66 | DANT1:2,INHBA-AS1:2,LINC00649:8,LINC00654:4,LINC00893:4,lnc-AC006455.1-10:1,lnc-AC008686.1-6:1,lnc-AIF1-2:1,lnc-AL669831.1-3:42,lnc-B3GNT9-1:1,lnc-BRI3-2:3,lnc-C10orf90-2:2,lnc-C11orf94-1:1,lnc-C14orf183-1:13,lnc-C15orf39-1:1,lnc-C17orf109-3:1,lnc-C1orf132-1:5,lnc-C1orf138-1:1,lnc-CAMK1-1:2,lnc-CCT5-2:1,lnc-CLDN9-1:1,lnc-COX19-2:2,lnc-CRYBA4-1:55,lnc-DDX52-3:1,lnc-EXOC2-8:4,lnc-IL32-1:3,lnc-KIAA0355-5:1,lnc-LAT-1:9,lnc-LPCAT1-3:2,lnc-LRCH4-2:1,lnc-MARCH7-1:2,lnc-MTA2-4:1,lnc-NKAIN3-1:2,lnc-PCBP3-3:9,lnc-POFUT2-5:1,lnc-PRAGMIN.1-3:2,lnc-PRKCZ-1:1,lnc-RABGEF1-1:1,lnc-RPL23A-2:1,lnc-RUNDC3A-4:2,lnc-SAMD8-1:1,lnc-SBDS-4:1,lnc-SEC11A-2:1,lnc-SERPINB6-1:7,lnc-SFPQ-2:4,lnc-SH2B2-7:1,lnc-SH3BGR-2:1,lnc-SLC12A8-1:1,lnc-SNURF-1:35,lnc-SP6-1:1,lnc-TACC2-3:3,lnc-TAF13-2:1,lnc-TAGLN2-1:1,lnc-TNNT1-1:1,lnc-ZSCAN10-3:17,PCBP1-AS1:198,SNAI3-AS1:16,TCL6:1,TCONS_00000037,TCONS_00031339,TCONS_00044060,TCONS_00046326,TCONS_00052323,TRAF3IP2-AS1:24,XIST:6,ZNF528-AS1:3 |
| SLC7A8 | 63 | BISPR:14,FAM201A:1,LBX2-AS1:2,LINC00861:6,LINC00893:4,LINC01426:3,lnc-ABHD2-4:1,lnc-AC006455.1-10:1,lnc-AMZ2-3:1,lnc-BCL2L2-PABPN1-1:2,lnc-C11orf94-1:1,lnc-C14orf79-3:1,lnc-C15orf39-1:1,lnc-C17orf109-3:1,lnc-C1orf138-1:1,lnc-C1orf186-3:1,lnc-CAMK1-1:2,lnc-CEP170-9:2,lnc-CLDN23-3:1,lnc-CLIC3-2:1,lnc-COX19-2:2,lnc-CRYBA4-1:55,lnc-CSNK1D-2:9,lnc-DDX52-3:1,lnc-DPY19L3-1:1,lnc-FTSJ1-2:1,lnc-GNA14-3:1,lnc-GRID2-1:1,lnc-HEATR2-3:1,lnc-KIAA0355-5:1,lnc-LAT-1:9,lnc-MAPK6-4:2,lnc-MOGAT2-3:1,lnc-OLFML3-5:1,lnc-PAIP2-1:1,lnc-PRAGMIN.1-3:2,lnc-PRKCZ-1:1,lnc-PUF60-1:1,lnc-RBAK-2:10,lnc-RCN1-2:1,lnc-RIPK1-1:2,lnc-ROPN1L-3:1,lnc-SBDS-4:1,lnc-SEC11A-1:3,lnc-SEC11A-2:1,lnc-SH3BGR-2:1,lnc-SIX5-1:1,lnc-TAF13-2:1,lnc-TAOK3-1:11,lnc-TRPM7-1:1,lnc-TRUB2-7:1,lnc-TTR-3:1,lnc-TYSND1-1:7,lnc-ZNF587-1:1,LOH12CR2:1,PCBP1-AS1:169,PCF11-AS1:2,PSMB8-AS1:1,PSMB8-AS1:3,SMIM2-AS1:11,TCONS_00031339,TCONS_00052860,ZNF528-AS1:3 |
| LOC105376684 | 63 | APTR:11,BISPR:14,CARD8-AS1:4,FAM201A:1,FAM74A6:1,GMDS-AS1:11,HOXA-AS2:2,LINC00476:3,LINC00649:8,LINC00894:20,LINC01347:8,lnc-AL669831.1-3:42,lnc-AP000769.1-1:2,lnc-ARL13B-1:5,lnc-BCL2L2-PABPN1-1:2,lnc-BRI3-2:3,lnc-C16orf72-7:1,lnc-C17orf62-6:6,lnc-C4orf36-2:5,lnc-CADM4-2:1,lnc-CAMK1-1:2,lnc-CLDN9-1:1,lnc-COX19-2:2,lnc-CTU2-2:1,lnc-DOC2B-3:2,lnc-ETFA-3:1,lnc-FAM200B-1:13,lnc-FTSJ1-2:1,lnc-GPR152-1:7,lnc-HTRA4-2:1,lnc-IL32-1:3,lnc-INTS2-4:2,lnc-LAG3-1:1,lnc-LGALS3BP-3:1,lnc-METRNL-7:4,lnc-NCDN-2:1,lnc-PCID2-1:2,lnc-PHLDA3-2:1,lnc-PKLR-7:1,lnc-PLCG2-5:1,lnc-PLEKHG5-3:1,lnc-POFUT2-4:1,lnc-POFUT2-5:1,lnc-RIPK1-1:2,lnc-SATB1-8:5,lnc-SH3BGR-2:1,lnc-SLC12A8-1:1,lnc-SLC7A6OS-4:2,lnc-STAB1-3:1,lnc-SYNDIG1L-2:3,lnc-TACC2-3:3,lnc-TMEM120B-4:3,lnc-TNFRSF1A-3:1,lnc-TNNT1-1:1,lnc-UBL5-5:1,lnc-VTI1A-1:3,lnc-ZBED5-1:2,lnc-ZFAND5-3:1,LOH12CR2:1,PCBP1-AS1:72,RGPD4-AS1:6,TCONS_00044060,THUMPD3-AS1:5 |
| AR | 59 | CHKB-AS1:14,DANT1:2,GAS6-AS2:1,LINC00847:14,LINC00887:5,LINC00893:4,LINC01299:2,lnc-ABCC6-2:1,lnc-ABHD2-4:1,lnc-AC006455.1-10:1,lnc-AC091132.2-7:1,lnc-ACR-2:5,lnc-ADA-2:1,lnc-AKAP7-1:1,lnc-AMZ2-3:1,lnc-ANKRD30B-7:2,lnc-APBA2-5:8,lnc-C14orf79-3:1,lnc-C20orf24-3:1,lnc-C2orf89-2:2,lnc-CLCN1-2:1,lnc-DDX52-3:1,lnc-EXOC2-8:4,lnc-FAM160A1-8:1,lnc-FRYL-3:3,lnc-HTRA2-1:1,lnc-KIAA0355-5:1,lnc-KLF13-2:4,lnc-LPCAT1-3:2,lnc-MOGAT2-3:1,lnc-NRARP-3:1,lnc-NT5C-3:3,lnc-PAIP2-1:1,lnc-PCBP3-3:9,lnc-PIN4-1:1,lnc-PKD2L1-5:1,lnc-PRAGMIN.1-3:2,lnc-PRDM11-9:2,lnc-PTPN12-1:1,lnc-PUF60-1:1,lnc-RBAK-2:10,lnc-ROPN1L-3:1,lnc-RP3-369A17.5.1-3:1,lnc-RPL23A-2:1,lnc-RUNDC3A-4:2,lnc-SEC11A-2:1,lnc-SIX5-1:1,lnc-TACR2-6:1,lnc-TYSND1-1:7,lnc-WDR73-7:2,PART1:10,PCF11-AS1:2,PSMB8-AS1:1,PSMB8-AS1:3,PSMB8-AS1:5,ST3GAL5-AS1:4,TCONS_00025364,TCONS_00049141,TCONS_00052860 |
| SLX1B | 55 | APTR:11,FAM201A:1,LBX2-AS1:2,LINC00649:8,LINC00654:4,LINC00894:20,lnc-AL669831.1-3:42,lnc-AP000769.1-1:2,lnc-C10orf90-2:2,lnc-C11orf94-1:1,lnc-C14orf79-3:1,lnc-C15orf39-1:1,lnc-C16orf72-7:1,lnc-C17orf109-3:1,lnc-C1orf138-1:1,lnc-C1orf186-3:1,lnc-C4orf36-2:5,lnc-CAMK1-1:2,lnc-CLDN23-3:1,lnc-CLDN9-1:1,lnc-CLIC3-2:1,lnc-COX19-2:2,lnc-CRYBA4-1:55,lnc-CSNK1D-2:9,lnc-DOC2B-3:2,lnc-DPY19L3-1:1,lnc-GPR152-1:7,lnc-HEATR2-3:1,lnc-LONRF2-3:1,lnc-LRRC56-3:4,lnc-MTA2-4:1,lnc-POFUT2-5:1,lnc-PRAGMIN.1-3:2,lnc-PUF60-1:1,lnc-RBAK-2:10,lnc-RCN1-2:1,lnc-RIPK1-1:2,lnc-RP11-389E17.1.1-1:1,lnc-SAMD8-1:1,lnc-SBDS-4:1,lnc-SEC11A-2:1,lnc-SLC12A8-1:1,lnc-TACC2-3:3,lnc-TAF13-2:1,lnc-TMEM120B-4:3,lnc-TNNT1-1:1,lnc-TTR-3:1,lnc-TYSND1-1:7,lnc-VTI1A-1:3,NDUFA6-AS1:5,RGPD4-AS1:6,SNAI3-AS1:16,TCONS_00044060,TRAF3IP2-AS1:24,ZNF528-AS1:3 |
| CLDN9 | 55 | FAM201A:1,LBX2-AS1:2,LINC00649:8,LINC00654:4,LINC01426:3,lnc-AC008686.1-6:1,lnc-AL669831.1-3:42,lnc-APH1A-1:6,lnc-BRI3-2:3,lnc-C10orf90-2:2,lnc-C11orf94-1:1,lnc-C14orf183-1:13,lnc-C15orf39-1:1,lnc-C17orf109-3:1,lnc-C1orf132-1:5,lnc-C1orf138-1:1,lnc-C1orf186-3:1,lnc-CAMK1-1:2,lnc-CLDN9-1:1,lnc-COX19-2:2,lnc-CRYBA4-1:55,lnc-DOC2B-3:2,lnc-ETFA-3:1,lnc-GPR152-1:7,lnc-HEATR2-3:1,lnc-IL32-1:3,lnc-LAG3-1:1,lnc-LGALS3BP-3:1,lnc-LRCH4-2:1,lnc-METRNL-7:4,lnc-MTA2-4:1,lnc-MTIF2-1:1,lnc-POFUT2-5:1,lnc-RABGEF1-1:1,lnc-RCN1-2:1,lnc-RIPK1-1:2,lnc-SATB1-8:5,lnc-SH2B2-7:1,lnc-SH3BGR-2:1,lnc-SLC12A8-1:1,lnc-SNURF-1:35,lnc-TACC2-3:3,lnc-TAF13-2:1,lnc-TAGLN2-1:1,lnc-TNFRSF1A-3:1,lnc-TNNT1-1:1,lnc-WDR38-1:1,NEAT1:14,PCBP1-AS1:181,TCONS_00000037,TCONS_00044060,TCONS_00046326,TCONS_00052323,TRAF3IP2-AS1:24,ZNF528-AS1:3 |
| VSTM1 | 54 | CHKB-AS1:14,GAS6-AS2:1,LBX2-AS1:2,LINC00847:14,LINC00887:5,LINC01299:2,LINC01426:3,lnc-ABCC6-2:1,lnc-ABHD2-4:1,lnc-AC006455.1-10:1,lnc-AC091132.2-7:1,lnc-ADA-2:1,lnc-AMZ2-3:1,lnc-BCL9L-1:1,lnc-C14orf79-3:1,lnc-CCT5-2:1,lnc-CRYBA4-1:55,lnc-EXOC2-8:4,lnc-EXT1-1:1,lnc-HEATR2-3:1,lnc-HSPG2-2:1,lnc-IRAK1-1:1,lnc-KIAA0355-5:1,lnc-KLF13-2:4,lnc-KLHL5-2:1,lnc-LIPI-9:1,lnc-MOGAT2-3:1,lnc-MTR-4:1,lnc-NKAIN3-1:2,lnc-OLFML3-5:1,lnc-OPRD1-4:1,lnc-PRAGMIN.1-3:2,lnc-PTPN12-1:1,lnc-PUF60-1:1,lnc-RABGEF1-1:1,lnc-RAD52-2:5,lnc-RCN1-2:1,lnc-SBDS-4:1,lnc-SCAPER-7:1,lnc-SEC11A-1:3,lnc-SEC11A-2:1,lnc-SIX5-1:1,lnc-TAOK3-1:11,lnc-TOR3A-1:1,lnc-TTR-3:1,lnc-TYSND1-1:7,lnc-UGCG-1:3,lnc-ZKSCAN3-1:1,PART1:10,PSMB8-AS1:1,PSMB8-AS1:5,SMIM2-AS1:11,ST3GAL5-AS1:4,TCONS_00052860 |
| MYADM | 54 | DANT1:2,GAS6-AS2:1,LBX2-AS1:2,LINC00654:4,LINC00893:4,LINC01426:3,lnc-AC006455.1-10:1,lnc-AC107021.1-7:1,lnc-AMZ2-3:1,lnc-B3GNT9-1:1,lnc-BCL9L-1:1,lnc-C14orf79-3:1,lnc-C1orf132-1:5,lnc-C3orf62-2:1,lnc-CCT5-2:1,lnc-CLDN23-3:1,lnc-CRYBA4-1:55,lnc-ELF2-1:1,lnc-EXOC2-8:4,lnc-EXT1-1:1,lnc-FTSJ1-2:1,lnc-GAS8-1:4,lnc-HEATR2-3:1,lnc-KLHL5-2:1,lnc-LIPI-9:1,lnc-MAPK6-4:2,lnc-MOGAT2-3:1,lnc-MTIF2-1:1,lnc-NKAIN3-1:2,lnc-OLFML3-5:1,lnc-OPRD1-4:1,lnc-PCBP3-3:9,lnc-RABGEF1-1:1,lnc-RCN1-2:1,lnc-ROPN1L-3:1,lnc-RP3-369A17.5.1-3:1,lnc-RPL23A-2:1,lnc-RUNDC3A-4:2,lnc-SBDS-4:1,lnc-SEC11A-1:3,lnc-SEC11A-2:1,lnc-SFPQ-2:4,lnc-SIX5-1:1,lnc-TAF13-2:1,lnc-TMOD3-3:7,lnc-TOR3A-1:1,lnc-TYSND1-1:7,lnc-UGCG-1:3,SNHG4:3,TCL6:1,TCONS_00031339,TCONS_00046326,TCONS_00052860,ZNF528-AS1:3 |
| NYX | 53 | LINC00476:3,LINC00649:8,LINC00654:4,lnc-AIF1-2:1,lnc-AL669831.1-3:42,lnc-B3GNT9-1:1,lnc-BRI3-2:3,lnc-C10orf90-2:2,lnc-C11orf94-1:1,lnc-C14orf183-1:13,lnc-C15orf39-1:1,lnc-C17orf109-3:1,lnc-C17orf77-1:6,lnc-C1orf138-1:1,lnc-CAMK1-1:2,lnc-CLDN9-1:1,lnc-COX19-2:2,lnc-CRYBA4-1:55,lnc-FPR2-1:10,lnc-IL32-1:3,lnc-KCND3-3:1,lnc-KLC2-2:1,lnc-LAT-1:9,lnc-MARCH7-1:2,lnc-MTA2-4:1,lnc-PAIP2-1:1,lnc-PCBP3-3:9,lnc-POFUT2-5:1,lnc-PRAGMIN.1-3:2,lnc-PRKRIR-1:5,lnc-PTGER1-1:2,lnc-PUF60-1:1,lnc-RP11-389E17.1.1-1:1,lnc-RPL23A-2:1,lnc-RUNDC3A-4:2,lnc-SAMD8-1:1,lnc-SEC11A-2:1,lnc-SERPINB6-1:7,lnc-SH2B2-7:1,lnc-TAF13-2:1,lnc-TNNT1-1:1,lnc-ZNF587-1:1,PCBP1-AS1:181,PCBP1-AS1:198,SNAI3-AS1:16,TCL6:1,TCONS_00000037,TCONS_00046326,TCONS_00052323,TRAF3IP2-AS1:24,UBA6-AS1:1,XIST:6,ZNF528-AS1:3 |
| TEAD2 | 50 | CHKB-AS1:14,FAM201A:1,LBX2-AS1:2,LINC00654:4,LINC00894:20,lnc-AC006455.1-10:1,lnc-AC022098.1-1:5,lnc-B3GNT9-1:1,lnc-BRI3-2:3,lnc-C11orf94-1:1,lnc-C14orf79-3:1,lnc-C17orf109-3:1,lnc-CAMK1-1:2,lnc-CEP170-9:2,lnc-CLDN23-3:1,lnc-CLDN9-1:1,lnc-CLIC3-2:1,lnc-COX19-2:2,lnc-CRYBA4-1:34,lnc-CRYBA4-1:55,lnc-GTPBP1-1:1,lnc-HEATR2-3:1,lnc-IRF2-2:3,lnc-LAT-1:9,lnc-MOGAT2-3:1,lnc-MTIF2-1:1,lnc-NAT1-6:1,lnc-OLFML3-5:1,lnc-PKLR-7:1,lnc-POFUT2-4:1,lnc-PRAGMIN.1-3:2,lnc-PRKRIR-1:5,lnc-PUF60-1:1,lnc-RCN1-2:1,lnc-RPL23A-2:1,lnc-RUNDC3A-4:2,lnc-SBDS-4:1,lnc-SIX5-1:1,lnc-SLC9A7-1:2,lnc-TAF13-2:1,lnc-TSSC4-5:2,lnc-TTR-3:1,lnc-TYSND1-1:7,lnc-ZSCAN10-3:17,PCF11-AS1:2,SMIM2-AS1:11,SNAI3-AS1:16,TCONS_00025364,TRAF3IP2-AS1:24,ZNF528-AS1:3 |
| RCN3 | 49 | FAM201A:1,FAM74A6:1,GMDS-AS1:11,HOXA-AS2:2,LINC00649:8,LINC00654:4,LINC01347:8,lnc-ABT1-5:1,lnc-AC008686.1-6:1,lnc-AL669831.1-3:42,lnc-AP000769.1-1:2,lnc-BRI3-2:3,lnc-C10orf90-2:2,lnc-C11orf94-1:1,lnc-C15orf39-1:1,lnc-C17orf109-3:1,lnc-C1orf138-1:1,lnc-C4orf36-2:5,lnc-CAMK1-1:2,lnc-CLDN9-1:1,lnc-COX19-2:2,lnc-CRYBA4-1:55,lnc-DOC2B-3:2,lnc-FAM200B-1:13,lnc-GPR152-1:7,lnc-IL32-1:3,lnc-LRCH4-2:1,lnc-METRNL-7:4,lnc-MTA2-4:1,lnc-PCID2-1:2,lnc-PLCG2-5:1,lnc-PLEKHG5-3:1,lnc-POFUT2-4:1,lnc-POFUT2-5:1,lnc-SH3BGR-2:1,lnc-SLC12A8-1:1,lnc-TACC2-3:3,lnc-TAF13-2:1,lnc-TAGLN2-1:1,lnc-TNFRSF1A-3:1,lnc-TNNT1-1:1,lnc-UBAC1-2:3,lnc-ZSCAN10-3:17,NDUFA6-AS1:5,SNAI3-AS1:16,TCONS_00000037,TCONS_00044060,TCONS_00052323,TRAF3IP2-AS1:24 |
| KCNK7 | 48 | CARD8-AS1:4,FAM201A:1,GMDS-AS1:11,HOXA-AS2:2,LINC00476:3,LINC00649:8,LINC00894:20,lnc-AP000769.1-1:2,lnc-BCL2L2-PABPN1-1:2,lnc-BRI3-2:3,lnc-C11orf94-1:1,lnc-C16orf72-7:1,lnc-C17orf62-6:6,lnc-C1orf186-3:1,lnc-CADM4-2:1,lnc-CLDN9-1:1,lnc-COX19-2:2,lnc-CRYBA4-1:55,lnc-CSNK1D-2:9,lnc-CTU2-2:1,lnc-DOC2B-3:2,lnc-ETFA-3:1,lnc-FAM200B-1:13,lnc-GPR152-1:7,lnc-GRID2-1:1,lnc-LAG3-1:1,lnc-LGALS3BP-3:1,lnc-METRNL-7:4,lnc-MTA2-4:1,lnc-PCID2-1:2,lnc-PHLDA3-2:1,lnc-PLCG2-5:1,lnc-PLEKHG5-3:1,lnc-POFUT2-4:1,lnc-POFUT2-5:1,lnc-RIPK1-1:2,lnc-SATB1-8:5,lnc-SH3BGR-2:1,lnc-SLC7A6OS-4:2,lnc-STAB1-3:1,lnc-TAF13-2:1,lnc-TNFRSF1A-3:1,lnc-VTI1A-1:3,lnc-ZBED5-1:2,LOH12CR2:1,NDUFA6-AS1:5,RGPD4-AS1:6,TRAF3IP2-AS1:24 |
| KLHL33 | 47 | CHKB-AS1:14,DANT1:2,GAS6-AS2:1,LINC00887:5,LINC00893:4,LINC01299:2,lnc-ABCC6-2:1,lnc-ABHD2-4:1,lnc-AC006455.1-10:1,lnc-AC091132.2-7:1,lnc-AMZ2-3:1,lnc-C14orf79-3:1,lnc-C20orf24-3:1,lnc-C3orf62-2:1,lnc-CARKD-4:1,lnc-CLIC3-2:1,lnc-HTRA2-1:1,lnc-KLF13-2:4,lnc-LAT-1:9,lnc-LIPI-9:1,lnc-MAPK6-4:2,lnc-MOGAT2-3:1,lnc-PAIP2-1:1,lnc-PCBP3-3:9,lnc-PRAGMIN.1-3:2,lnc-PRDM11-9:2,lnc-PUF60-1:1,lnc-RBAK-2:10,lnc-RCN1-2:1,lnc-ROPN1L-3:1,lnc-RPL23A-2:1,lnc-RUNDC3A-4:2,lnc-SAG-7:2,lnc-SBDS-4:1,lnc-SEC11A-2:1,lnc-SIX5-1:1,lnc-TYSND1-1:7,lnc-WDR73-7:2,lnc-ZNF827-11:1,PART1:10,PSMB8-AS1:1,PSMB8-AS1:5,SLC25A25-AS1:15,TCONS_00025364,TCONS_00049141,TCONS_00052860,ZNF528-AS1:3 |
| PVRL2 | 45 | FTX:1,GAS6-AS2:1,LINC00847:14,LINC00887:5,lnc-ABCC6-2:1,lnc-ABHD2-4:1,lnc-AC091132.2-7:1,lnc-ADA-2:1,lnc-AMZ2-3:1,lnc-ANKRD30B-7:2,lnc-APBA2-5:8,lnc-BCL9L-1:1,lnc-C14orf79-3:1,lnc-C20orf24-3:1,lnc-CARKD-4:1,lnc-CLCN1-2:1,lnc-CLIC3-2:1,lnc-EXT1-1:1,lnc-FAM160A1-8:1,lnc-FRYL-3:3,lnc-HTRA2-1:1,lnc-KIAA0355-5:1,lnc-KLF14-1:1,lnc-LIPI-9:1,lnc-PAIP2-1:1,lnc-PIN4-1:1,lnc-POTEF-8:2,lnc-PRDM11-9:2,lnc-PTPN12-1:1,lnc-RBAK-2:10,lnc-RBPMS2-1:2,lnc-ROPN1L-3:1,lnc-SEC11A-2:1,lnc-SIX5-1:1,lnc-TAOK3-1:11,lnc-TOR3A-1:1,lnc-TYSND1-1:7,lnc-WDR73-7:2,lnc-ZNF827-11:1,PART1:10,PSMB8-AS1:1,PSMB8-AS1:3,PSMB8-AS1:5,ST3GAL5-AS1:4,TCONS_00052860 |
| HSD17B13 | 43 | INHBA-AS1:2,LINC00476:3,LINC00649:8,LINC00654:4,LINC01347:8,LINC01426:3,lnc-ABT1-5:1,lnc-AL669831.1-3:42,lnc-AP000769.1-1:2,lnc-ARL13B-1:5,lnc-C10orf90-2:2,lnc-C11orf94-1:1,lnc-C4orf36-2:5,lnc-CADM4-2:1,lnc-CEP170-9:2,lnc-CLDN9-1:1,lnc-FTSJ1-2:1,lnc-HEATR2-3:1,lnc-IRAK1-1:1,lnc-MTIF2-1:1,lnc-NCDN-2:1,lnc-OLFML3-5:1,lnc-OPRD1-4:1,lnc-PKLR-7:1,lnc-PRKRIR-1:5,lnc-RAP1GAP2-3:1,lnc-RCN1-2:1,lnc-RIPK1-1:2,lnc-SLC12A8-1:1,lnc-SLC7A6OS-4:2,lnc-TACC2-3:3,lnc-TAF13-2:1,lnc-TMEM120B-4:3,lnc-TMOD3-3:7,lnc-TNFRSF1A-3:1,lnc-TTR-3:1,lnc-VTI1A-1:3,lnc-ZSCAN10-3:17,LOH12CR2:1,RGPD4-AS1:6,SMIM2-AS1:11,THUMPD3-AS1:5,UGDH-AS1:6 |
| GTPBP6 | 43 | CHKB-AS1:14,DANT1:2,FTX:1,GAS6-AS2:1,LBX2-AS1:2,LINC00847:14,LINC00887:5,lnc-ABCC6-2:1,lnc-AC091132.2-7:1,lnc-ACR-2:5,lnc-AMZ2-3:1,lnc-APBA2-5:8,lnc-BCL9L-1:1,lnc-C14orf79-3:1,lnc-CARKD-4:1,lnc-CLCN1-2:1,lnc-CLIC3-2:1,lnc-EXT1-1:1,lnc-FRYL-3:3,lnc-HEATR2-3:1,lnc-HTRA2-1:1,lnc-KLF13-2:4,lnc-LRRC56-3:4,lnc-MOGAT2-3:1,lnc-NRARP-3:1,lnc-NT5C-3:3,lnc-PCBP3-3:9,lnc-PIN4-1:1,lnc-PRAGMIN.1-3:2,lnc-PUF60-1:1,lnc-RBAK-2:10,lnc-RUNDC3A-4:2,lnc-SBDS-4:1,lnc-SEC11A-2:1,lnc-SIX5-1:1,lnc-TYSND1-1:7,lnc-WDR73-7:2,lnc-ZNF827-11:1,ST3GAL5-AS1:4,TCONS_00049141,TCONS_00052658,TCONS_00052860,ZNF528-AS1:3 |
| DNM1 | 41 | FTX:1,GAS6-AS2:1,LINC00847:14,LINC00887:5,lnc-ABCC6-2:1,lnc-AC091132.2-7:1,lnc-ACR-2:5,lnc-AMZ2-3:1,lnc-ANKRD30B-7:2,lnc-APBA2-5:8,lnc-BCL9L-1:1,lnc-C14orf79-3:1,lnc-C20orf24-3:1,lnc-CARKD-4:1,lnc-CLCN1-2:1,lnc-CLIC3-2:1,lnc-FAM160A1-8:1,lnc-FRYL-3:3,lnc-HTRA2-1:1,lnc-KLF13-2:4,lnc-KLF14-1:1,lnc-LIPI-9:1,lnc-NRARP-3:1,lnc-NT5C-3:3,lnc-PAIP2-1:1,lnc-PIN4-1:1,lnc-PRDM11-9:2,lnc-RAD52-2:5,lnc-RBAK-2:10,lnc-ROPN1L-3:1,lnc-SIX5-1:1,lnc-TOR3A-1:1,lnc-TYSND1-1:7,lnc-WDR73-7:2,PART1:10,PCF11-AS1:2,PSMB8-AS1:1,PSMB8-AS1:5,ST3GAL5-AS1:4,TCONS_00049141,TCONS_00052860 |
| CLK2 | 39 | FAM201A:1,GMDS-AS1:11,HOXA-AS2:2,LINC00476:3,lnc-AC008686.1-6:1,lnc-AL669831.1-3:42,lnc-AP000769.1-1:2,lnc-BRI3-2:3,lnc-C10orf90-2:2,lnc-C17orf109-3:1,lnc-CAMK1-1:2,lnc-CLDN9-1:1,lnc-COX19-2:2,lnc-CRYBA4-1:55,lnc-CTU2-2:1,lnc-DFNB59-2:14,lnc-DOC2B-3:2,lnc-FAM156B-1:1,lnc-FPR2-1:10,lnc-HAX1-4:1,lnc-INTS2-4:2,lnc-LAG3-1:1,lnc-LAT-1:9,lnc-LRCH4-2:1,lnc-MARCH7-1:2,lnc-MTA2-4:1,lnc-MTIF2-1:1,lnc-NDRG1-1:4,lnc-PCID2-1:2,lnc-PLCG2-5:1,lnc-PLEKHG5-3:1,lnc-POFUT2-5:1,lnc-TNFRSF1A-3:1,lnc-TNNT1-1:1,PCBP1-AS1:72,RGPD4-AS1:6,SNAI3-AS1:16,TCONS_00044060,TRAF3IP2-AS1:24 |
| ZMYND10 | 37 | GAS6-AS2:1,LBX2-AS1:2,LINC00887:5,LINC01299:2,lnc-ABCC6-2:1,lnc-AC091132.2-7:1,lnc-AC107021.1-7:1,lnc-ANKRD30B-7:2,lnc-APBA2-5:8,lnc-C11orf94-1:1,lnc-C20orf24-3:1,lnc-CARKD-4:1,lnc-CEP170-9:2,lnc-CLIC3-2:1,lnc-COX19-2:2,lnc-FRYL-3:3,lnc-HEATR2-3:1,lnc-KLF13-2:4,lnc-MOGAT2-3:1,lnc-NT5C-3:3,lnc-PAIP2-1:1,lnc-PCBP3-3:9,lnc-PRAGMIN.1-3:2,lnc-PRDM11-9:2,lnc-PTGER1-1:2,lnc-PUF60-1:1,lnc-RBAK-2:10,lnc-RCN1-2:1,lnc-RUNDC3A-4:2,lnc-SEC11A-2:1,lnc-SIX5-1:1,lnc-TAOK3-1:11,lnc-WDR73-7:2,TCONS_00052860,THUMPD3-AS1:5,UBA6-AS1:1,ZNF528-AS1:3 |
| PDZD2 | 37 | CHKB-AS1:14,LINC00847:14,LINC00887:5,lnc-ABCC6-2:1,lnc-ABHD2-4:1,lnc-AC069257.9.1-5:5,lnc-AC091132.2-7:1,lnc-ACR-2:5,lnc-ADA-2:1,lnc-AMZ2-3:1,lnc-APBA2-5:8,lnc-C14orf79-3:1,lnc-CARKD-4:1,lnc-FAM160A1-8:1,lnc-FRYL-3:3,lnc-HTRA2-1:1,lnc-KLF13-2:4,lnc-KLF14-1:1,lnc-MAP3K3-2:2,lnc-MAPK6-4:2,lnc-NRARP-3:1,lnc-NT5C-3:3,lnc-PIN4-1:1,lnc-PRDM11-9:2,lnc-PUF60-1:1,lnc-RBAK-2:10,lnc-SIX5-1:1,lnc-TYSND1-1:7,lnc-WDR73-7:2,lnc-ZNF827-11:1,PART1:10,PCF11-AS1:2,PSMB8-AS1:1,PSMB8-AS1:5,ST3GAL5-AS1:4,TCONS_00052860,UBA6-AS1:1 |
| OPLAH | 35 | FAM201A:1,GMDS-AS1:11,HOXA-AS2:2,LINC00476:3,LINC00894:20,lnc-ABT1-5:1,lnc-AP006621.5.1-2:2,lnc-APH1A-1:6,lnc-BRI3-2:3,lnc-C17orf62-6:6,lnc-C4orf36-2:5,lnc-CLDN9-1:1,lnc-CTU2-2:1,lnc-DOC2B-3:2,lnc-FAM200B-1:13,lnc-LAG3-1:1,lnc-MDH1B-8:2,lnc-METRNL-7:4,lnc-PCID2-1:2,lnc-PHLDA3-2:1,lnc-PKLR-7:1,lnc-PLCG2-5:1,lnc-PLEKHG5-3:1,lnc-POFUT2-5:1,lnc-RAB3D-2:2,lnc-RP11-389E17.1.1-1:1,lnc-SATB1-8:5,lnc-SLC12A8-1:1,lnc-SNURF-1:35,lnc-STAB1-3:1,lnc-SYNDIG1L-2:3,lnc-TNFRSF1A-3:1,lnc-UBAC1-2:3,RGPD4-AS1:6,TCONS_00044060 |
| LOC101927789 | 35 | BISPR:14,CARD8-AS1:4,FAM74A6:1,GMDS-AS1:11,HOXA-AS2:2,LINC00476:3,lnc-AL669831.1-3:42,lnc-AP000769.1-1:2,lnc-C16orf72-7:1,lnc-C17orf62-6:6,lnc-C4orf36-2:5,lnc-CADM4-2:1,lnc-CLDN9-1:1,lnc-DFNB59-2:14,lnc-DOC2B-3:2,lnc-IL32-1:3,lnc-INTS2-4:2,lnc-LAG3-1:1,lnc-METRNL-7:4,lnc-MTA2-4:1,lnc-PCID2-1:2,lnc-PHLDA3-2:1,lnc-PKLR-7:1,lnc-PLCG2-5:1,lnc-PLEKHG5-3:1,lnc-POFUT2-5:1,lnc-RP11-389E17.1.1-1:1,lnc-SLC12A8-1:1,lnc-TACC2-3:3,lnc-TNFRSF1A-3:1,lnc-UBAC1-2:3,lnc-UBL5-5:1,lnc-ZFAND5-3:1,RGPD4-AS1:6,TCONS_00044060 |
| MOK | 34 | LBX2-AS1:2,LINC00654:4,LINC00887:5,lnc-ABCC6-2:1,lnc-ABHD2-4:1,lnc-AC091132.2-7:1,lnc-C11orf94-1:1,lnc-C14orf79-3:1,lnc-CARKD-4:1,lnc-CLIC3-2:1,lnc-COX19-2:2,lnc-CRYBA4-1:55,lnc-CSNK1D-2:9,lnc-FRYL-3:3,lnc-GTPBP1-1:1,lnc-HEATR2-3:1,lnc-HTRA2-1:1,lnc-KLF13-2:4,lnc-MOGAT2-3:1,lnc-NT5C-3:3,lnc-PCBP3-3:9,lnc-PRAGMIN.1-3:2,lnc-RBAK-2:10,lnc-RBPMS2-1:2,lnc-RUNDC3A-4:2,lnc-SBDS-4:1,lnc-SEC11A-2:1,lnc-SIX5-1:1,lnc-TAF13-2:1,PSMB8-AS1:1,PSMB8-AS1:3,PSMB8-AS1:5,SNHG4:3,TCONS_00052860 |
| MGC57346-CRHR1 | 34 | CHKB-AS1:14,DANT1:2,LINC00887:5,LINC00893:4,lnc-ABCC6-2:1,lnc-ABHD2-4:1,lnc-AC091132.2-7:1,lnc-AC107021.1-7:1,lnc-ACP1-15:1,lnc-APBA2-5:8,lnc-C14orf79-3:1,lnc-C20orf24-3:1,lnc-CLCN1-2:1,lnc-CLIC3-2:1,lnc-DDX52-3:1,lnc-FRYL-3:3,lnc-HEATR2-3:1,lnc-HTRA2-1:1,lnc-KLF13-2:4,lnc-LIPI-9:1,lnc-MOGAT2-3:1,lnc-PCBP3-3:9,lnc-PTPN12-1:1,lnc-PUF60-1:1,lnc-RBAK-2:10,lnc-RCN1-2:1,lnc-RUNDC3A-4:2,lnc-SIX5-1:1,lnc-TAOK3-1:11,lnc-TYSND1-1:7,lnc-ZNF827-11:1,PSMB8-AS1:5,TCONS_00049141,TCONS_00052860 |
| YTHDC1 | 33 | lnc-ABHD2-4:1,lnc-AIF1-2:1,lnc-C15orf39-1:1,lnc-C16orf72-7:1,lnc-CCT5-2:1,lnc-DDX52-3:1,lnc-EXOC2-8:4,lnc-IFT52-1:2,lnc-KBTBD4-3:1,lnc-KIAA0355-5:1,lnc-KLHL5-2:1,lnc-LPCAT1-3:2,lnc-NKAIN3-1:2,lnc-PTPN12-1:1,lnc-RABGEF1-1:1,lnc-RP3-369A17.5.1-3:1,lnc-SAMD8-1:1,lnc-SEC11A-1:3,lnc-SEC11A-2:1,lnc-SH2B2-7:1,lnc-SH3BGR-2:1,lnc-SLC26A11-1:1,lnc-SP6-1:1,lnc-TNNT1-1:1,lnc-TRUB2-7:1,lnc-WDR38-1:1,lnc-ZNF587-1:1,PCBP1-AS1:169,PSMB8-AS1:1,PSMB8-AS1:3,TCONS_00000037,TCONS_00031339,THUMPD3-AS1:27 |
| TMEM119 | 32 | BISPR:14,CARD8-AS1:4,FAM201A:1,FAM74A6:1,GMDS-AS1:11,HOXA-AS2:2,LINC00476:3,lnc-AP000769.1-1:2,lnc-ARL13B-1:5,lnc-C16orf72-7:1,lnc-C17orf62-6:6,lnc-C4orf36-2:5,lnc-CADM4-2:1,lnc-DFNB59-2:14,lnc-DOC2B-3:2,lnc-FAM200B-1:13,lnc-HTRA4-2:1,lnc-INTS2-4:2,lnc-LAG3-1:1,lnc-METRNL-7:4,lnc-NDRG1-1:4,lnc-PCID2-1:2,lnc-PHLDA3-2:1,lnc-PLEKHG5-3:1,lnc-SATB1-8:5,lnc-STAB1-3:1,lnc-SYNDIG1L-2:3,lnc-TNFRSF1A-3:1,lnc-UBL5-5:1,lnc-ZBED5-1:2,lnc-ZFAND5-3:1,RGPD4-AS1:6 |
| NEO1 | 27 | A2M-AS1:3,BZRAP1-AS1:19,BZRAP1-AS1:20,BZRAP1-AS1:21,LINC00888:6,LINC00987:3,lnc-AAGAB-3:2,lnc-ATF7IP2-3:4,lnc-C12orf39-2:4,lnc-CLK3-1:1,lnc-ERICH1-19:8,lnc-FRYL-3:4,lnc-GBP5-2:6,lnc-GET4-1:2,lnc-GNLY-2:3,lnc-LILRB5-1:1,lnc-MARCH7-1:1,lnc-OR4F16-5:16,lnc-RNF150-2:2,lnc-SLC17A9-5:1,lnc-TBC1D28-1:1,lnc-VPREB1-7:11,lnc-XRCC1-2:1,lnc-YPEL5-5:1,TCONS_00049092,TCONS_00049095,ZNF674-AS1:3 |
| TSSK6 | 26 | LINC00476:3,LINC00649:8,LINC00654:4,LINC00894:20,lnc-AL669831.1-3:42,lnc-B3GNT9-1:1,lnc-BRI3-2:3,lnc-C10orf90-2:2,lnc-C4orf36-2:5,lnc-CAMK1-1:2,lnc-CLDN9-1:1,lnc-COX19-2:2,lnc-CRYBA4-1:55,lnc-FPR2-1:10,lnc-LAT-1:9,lnc-METRNL-7:4,lnc-MTA2-4:1,lnc-POFUT2-5:1,lnc-RP11-389E17.1.1-1:1,lnc-RUNDC3A-4:2,lnc-SATB1-8:5,lnc-SERPINB6-1:7,lnc-TSSC4-5:2,lnc-UBL5-5:1,RGPD4-AS1:6,SNAI3-AS1:16 |
| TMEM88 | 26 | FAM201A:1,GMDS-AS1:11,lnc-AC008686.1-6:1,lnc-AP000769.1-1:2,lnc-C17orf62-6:6,lnc-CLDN9-1:1,lnc-DOC2B-3:2,lnc-FAM156B-1:1,lnc-FAM200B-1:13,lnc-FLRT2-2:10,lnc-GPR152-1:7,lnc-LAG3-1:1,lnc-LRCH4-2:1,lnc-METRNL-7:4,lnc-MTA2-4:1,lnc-PCID2-1:2,lnc-PLCG2-5:1,lnc-PLEKHG5-3:1,lnc-POFUT2-4:1,lnc-POFUT2-5:1,lnc-SLC12A8-1:1,lnc-TACC2-3:3,lnc-TNFRSF1A-3:1,RGPD4-AS1:6,STXBP5-AS1:13,TCONS_00044060 |
| SLC43A2 | 26 | LINC00476:3,LINC00894:20,lnc-BCL2L2-PABPN1-1:2,lnc-C16orf72-7:1,lnc-C4orf36-2:5,lnc-C5orf25-3:3,lnc-CADM4-2:1,lnc-DOC2B-3:2,lnc-DPY19L3-1:1,lnc-GRID2-1:1,lnc-GSR-3:1,lnc-L3MBTL4-4:1,lnc-NCDN-2:1,lnc-NMRAL1-2:1,lnc-OR2AT4-1:1,lnc-PKLR-7:1,lnc-PRKRIR-1:5,lnc-RIPK1-1:2,lnc-SLC7A6OS-4:2,lnc-TNFRSF1A-3:1,lnc-VTI1A-1:3,lnc-WDR38-1:1,LOH12CR2:1,RGPD4-AS1:6,THUMPD3-AS1:5,TTC21B-AS1:3 |
| LOC105369154 | 26 | GMDS-AS1:11,HOXA-AS2:2,lnc-AP006621.5.1-2:2,lnc-BRI3-2:3,lnc-C17orf62-6:6,lnc-CLDN9-1:1,lnc-CTU2-2:1,lnc-DFNB59-2:14,lnc-FAM200B-1:13,lnc-HAX1-4:1,lnc-INTS2-4:2,lnc-KLF14-1:6,lnc-LAG3-1:1,lnc-METRNL-7:4,lnc-MTA2-4:1,lnc-PCID2-1:2,lnc-PHLDA3-2:1,lnc-PLCG2-5:1,lnc-PLEKHG5-3:1,lnc-RP11-389E17.1.1-1:1,lnc-SLC12A8-1:1,lnc-STAB1-3:1,lnc-SYNDIG1L-2:3,RGPD4-AS1:6,STXBP5-AS1:13,TCONS_00044060 |
| RASA4 | 25 | CARD8-AS1:4,HOXA-AS2:2,LINC00476:3,lnc-ARL13B-1:5,lnc-C17orf62-6:6,lnc-C4orf36-2:5,lnc-CTU2-2:1,lnc-DFNB59-2:14,lnc-DOC2B-3:2,lnc-FAM200B-1:13,lnc-FLRT2-2:10,lnc-FPR2-1:10,lnc-HTRA4-2:1,lnc-INTS2-4:2,lnc-KLF14-1:6,lnc-LAG3-1:1,lnc-NDRG1-1:4,lnc-PCID2-1:2,lnc-PHLDA3-2:1,lnc-PLCG2-5:1,lnc-PLEKHG5-3:1,lnc-RAB3D-2:2,lnc-SYNDIG1L-2:3,lnc-ZFAND5-3:1,RGPD4-AS1:6 |
| ZSWIM5 | 24 | A2M-AS1:3,BZRAP1-AS1:19,BZRAP1-AS1:20,DICER1-AS1:2,lnc-ALKBH4-8:1,lnc-BCL11B-1:2,lnc-CLK3-1:1,lnc-COG6-7:1,lnc-DNAJC8-1:1,lnc-ERICH1-19:8,lnc-FBXO3-3:2,lnc-GBP5-2:6,lnc-GNLY-2:3,lnc-KLHL9-1:1,lnc-MMP23B-5:2,lnc-SLC17A9-5:1,lnc-VPREB1-7:11,lnc-YPEL5-5:1,lnc-ZNF22-3:2,lnc-ZNF737-3:5,TCONS_00018893,TCONS_00049082,TCONS_00049096,TCONS_00052889 |
| TRPC3 | 24 | GAS6-AS2:1,LINC00847:14,LINC00887:5,lnc-ABCC6-2:1,lnc-AC091132.2-7:1,lnc-ACR-2:5,lnc-APBA2-5:8,lnc-C14orf79-3:1,lnc-C20orf24-3:1,lnc-C2orf89-2:2,lnc-CARKD-4:1,lnc-CLCN1-2:1,lnc-FAM160A1-8:1,lnc-FRYL-3:3,lnc-HTRA2-1:1,lnc-KLF13-2:4,lnc-MOGAT2-3:1,lnc-POTEF-8:2,lnc-PRDM11-9:2,lnc-SIX5-1:1,lnc-TLR1-1:1,lnc-TYSND1-1:7,ST3GAL5-AS1:4,TCONS_00052860 |
| ACTL7B | 24 | LBX2-AS1:2,LINC00847:14,lnc-ABCC6-2:1,lnc-ABHD2-4:1,lnc-AC091132.2-7:1,lnc-AC107021.1-7:1,lnc-ACR-2:5,lnc-AMZ2-3:1,lnc-CLCN1-2:1,lnc-FAM160A1-8:1,lnc-FRYL-3:3,lnc-HTRA2-1:1,lnc-MOGAT2-3:1,lnc-NRARP-3:1,lnc-NT5C-3:3,lnc-OLFML3-5:1,lnc-SBDS-4:1,lnc-SEC11A-2:1,lnc-SIX5-1:1,lnc-TYSND1-1:7,lnc-WDR73-7:2,lnc-ZNF827-11:1,ST3GAL5-AS1:4,TCONS_00052860 |
| UNC5B | 23 | LINC00278:1,LINC00278:4,LINC00869:34,lnc-CLLU1.1-1:10,lnc-COG6-7:1,lnc-DNAJC8-1:1,lnc-DUOXA1-1:1,lnc-ERICH1-19:8,lnc-FAM160A1-6:3,lnc-GOLGA8IP-2:1,lnc-HSFY2-13:1,lnc-KDM5D-4:1,lnc-LST1-1:5,lnc-MICA-9:1,lnc-MMP23B-5:2,lnc-PXDC1-14:4,lnc-RP11-712L6.5.1-2:3,lnc-TBL1Y-6:2,MIR22HG:3,TTTY14:13,TTTY15:2,TTTY15:5,TTTY15:6 |
| ITGAD | 23 | LBX2-AS1:2,LINC00887:5,lnc-ABCC6-2:1,lnc-AC091132.2-7:1,lnc-ACR-2:5,lnc-APBA2-5:8,lnc-C14orf79-3:1,lnc-CLCN1-2:1,lnc-FRYL-3:3,lnc-HTRA2-1:1,lnc-LIPI-9:1,lnc-MOGAT2-3:1,lnc-RBPMS2-1:2,lnc-SBDS-4:1,lnc-SEC11A-2:1,lnc-SIX5-1:1,lnc-TYSND1-1:7,lnc-WDR73-7:2,PSMB8-AS1:1,PSMB8-AS1:5,ST3GAL5-AS1:4,TCONS_00052658,TCONS_00052860 |
| KIAA1731NL | 22 | FAM74A6:1,HOXA-AS2:2,lnc-AL669831.1-3:42,lnc-C10orf90-2:2,lnc-C1orf138-1:1,lnc-CADM4-2:1,lnc-CLDN9-1:1,lnc-DOC2B-3:2,lnc-FAM200B-1:13,lnc-GPR152-1:7,lnc-IL32-1:3,lnc-LRCH4-2:1,lnc-NMRAL1-2:1,lnc-POFUT2-4:1,lnc-POFUT2-5:1,lnc-SATB1-8:5,lnc-SYNDIG1L-2:3,lnc-TNFRSF1A-3:1,lnc-TNNT1-1:1,PCBP1-AS1:181,TCONS_00000037,TCONS_00044060 |
| ELF3 | 22 | FAM201A:1,GMDS-AS1:11,LINC00649:8,lnc-ACSBG2-2:1,lnc-AL669831.1-3:42,lnc-APH1A-1:6,lnc-CLDN9-1:1,lnc-DOC2B-3:2,lnc-FPR2-1:10,lnc-METRNL-7:4,lnc-MTA2-4:1,lnc-PCID2-1:2,lnc-PLCG2-5:1,lnc-PLEKHG5-3:1,lnc-POFUT2-4:1,lnc-RP11-389E17.1.1-1:1,lnc-SATB1-8:5,lnc-SLC12A8-1:1,lnc-SYNDIG1L-2:3,lnc-TAGLN2-1:1,SNAI3-AS1:16,TCONS_00044060 |
| LOC102724378 | 21 | lnc-ABT1-5:1,lnc-AL669831.1-3:42,lnc-C10orf90-2:2,lnc-EXOC2-8:4,lnc-HAX1-4:1,lnc-IL32-1:3,lnc-KBTBD4-3:1,lnc-KCND3-3:1,lnc-KLC2-2:1,lnc-PTGER1-1:2,lnc-RP3-369A17.5.1-3:1,lnc-SAMD8-1:1,lnc-SERPINB6-1:7,lnc-SH2B2-7:1,lnc-ZNF587-1:1,SLC25A25-AS1:15,STXBP5-AS1:13,TCONS_00000037,TCONS_00046326,TCONS_00052323,XIST:6 |
| DDX39B | 21 | INHBA-AS1:2,LINC01299:2,lnc-BCL2L2-PABPN1-1:2,lnc-C10orf90-2:2,lnc-C11orf94-1:1,lnc-C17orf109-3:1,lnc-CLDN23-3:1,lnc-CLIC3-2:1,lnc-CRYBA4-1:55,lnc-HEATR2-3:1,lnc-PAIP2-1:1,lnc-PUF60-1:1,lnc-SBDS-4:1,lnc-SEC11A-2:1,lnc-SIX5-1:1,lnc-TAF13-2:1,lnc-TAOK3-1:11,lnc-TOR3A-1:1,lnc-TYSND1-1:7,SNAI3-AS1:16,TCONS_00052860 |
| SLC7A9 | 20 | LINC00887:5,lnc-ABCC6-2:1,lnc-AC091132.2-7:1,lnc-AMZ2-3:1,lnc-ANKRD30B-7:2,lnc-APBA2-5:8,lnc-C1orf186-3:1,lnc-CARKD-4:1,lnc-CLIC3-2:1,lnc-COX19-2:2,lnc-CRYBA4-1:55,lnc-HEATR2-3:1,lnc-NT5C-3:3,lnc-PCBP3-3:9,lnc-PRAGMIN.1-3:2,lnc-SBDS-4:1,lnc-TYSND1-1:7,lnc-WDR73-7:2,MIR4435-2HG:9,TCONS_00052860 |
| LTB | 20 | FAM74A6:1,HOXA-AS2:2,lnc-AP000769.1-1:2,lnc-BRI3-2:3,lnc-C4orf36-2:5,lnc-CTU2-2:1,lnc-DOC2B-3:2,lnc-FAM200B-1:13,lnc-HTRA4-2:1,lnc-KLF14-1:6,lnc-MDH1B-8:2,lnc-METRNL-7:4,lnc-MTA2-4:1,lnc-PCID2-1:2,lnc-PHLDA3-2:1,lnc-PLEKHG5-3:1,lnc-SYNDIG1L-2:3,lnc-ZBED5-1:2,PCBP1-AS1:72,TCONS_00044060 |
| CXCR6 | 20 | LINC00888:6,LINC00996:2,LINC01341:3,lnc-AAGAB-3:2,lnc-ALKBH4-8:1,lnc-AMPH-10:7,lnc-C7orf42-4:5,lnc-CCDC103-4:1,lnc-FAM27A-4:1,lnc-GMDS-14:1,lnc-GUSB-12:1,lnc-LILRB5-1:1,lnc-MFN1-1:3,lnc-PSMB9-6:4,lnc-TFPI-1:4,lnc-VPREB1-7:11,lnc-VSTM5-1:9,lnc-XRCC1-2:1,TCONS_00020149,TCONS_00049095 |
| CSF2RA | 20 | INHBA-AS1:2,lnc-AP000769.1-1:2,lnc-C11orf94-1:1,lnc-CEP170-9:2,lnc-FTSJ1-2:1,lnc-FUK-2:1,lnc-FXYD4-4:2,lnc-GAS8-1:4,lnc-HEATR2-3:1,lnc-IRAK1-1:1,lnc-OLFML3-5:1,lnc-PKLR-7:1,lnc-SLC9A7-1:2,lnc-TMEM120B-4:3,lnc-VTI1A-1:3,LOH12CR2:1,SMIM2-AS1:11,THUMPD3-AS1:5,UGDH-AS1:6,ZNF528-AS1:3 |
| PRSS21 | 19 | APTR:11,LBX2-AS1:2,lnc-ABHD2-4:1,lnc-C11orf94-1:1,lnc-C14orf79-3:1,lnc-C1orf186-3:1,lnc-COX19-2:2,lnc-CRYBA4-1:55,lnc-CSNK1D-2:9,lnc-FXYD4-4:2,lnc-MOGAT2-3:1,lnc-NRARP-3:1,lnc-PAIP2-1:1,lnc-ROPN1L-3:1,lnc-SBDS-4:1,lnc-SIX5-1:1,lnc-TYSND1-1:7,NDUFA6-AS1:5,TCONS_00052860 |
| PDE9A | 19 | CHKB-AS1:14,LINC00887:5,LINC01299:2,lnc-AC091132.2-7:1,lnc-AC107021.1-7:1,lnc-ANKRD30B-7:2,lnc-APBA2-5:8,lnc-C14orf79-3:1,lnc-CLIC3-2:1,lnc-FAM160A1-8:1,lnc-HEATR2-3:1,lnc-MOGAT2-3:1,lnc-PUF60-1:1,lnc-RBAK-2:10,lnc-RUNDC3A-4:2,lnc-SIX5-1:1,lnc-WDR73-7:2,PCF11-AS1:2,TCONS_00052860 |
| BIVM | 19 | LINC00888:6,LINC01184:24,lnc-AAGAB-3:2,lnc-ATF7IP2-7:1,lnc-C3orf79-2:1,lnc-CLK3-1:1,lnc-COG6-7:1,lnc-DNAJC8-1:1,lnc-ERICH1-19:8,lnc-LILRB5-1:1,lnc-OR4F29-3:4,lnc-PXDC1-14:4,lnc-RGMB-1:2,lnc-RP11-158I9.5.1-2:1,lnc-XRCC1-2:1,lnc-ZNF131-10:3,MIR22HG:3,PSMD5-AS1:22,ZNF674-AS1:3 |
| LOC101927853 | 17 | LBX2-AS1:2,LINC00654:4,lnc-ACR-2:5,lnc-APBA2-5:8,lnc-B3GNT9-1:1,lnc-CLIC3-2:1,lnc-EXOC2-8:4,lnc-FRYL-3:3,lnc-HEATR2-3:1,lnc-HTRA2-1:1,lnc-MOGAT2-3:1,lnc-PRAGMIN.1-3:2,lnc-RUNDC3A-4:2,lnc-SBDS-4:1,lnc-SIX5-1:1,lnc-UGCG-1:3,TCONS_00052860 |
| KLRG1 | 17 | LINC00888:6,LINC01184:20,lnc-ACTR1B-3:10,lnc-AL359853.2-2:1,lnc-ATF7IP2-7:1,lnc-C11orf54-2:1,lnc-C19orf52-1:1,lnc-GOLGA8IP-2:1,lnc-GUSB-12:1,lnc-KLHL9-1:1,lnc-KLRG1-3:2,lnc-N6AMT1-1:5,lnc-RGMB-1:2,lnc-TRIM41-1:6,lnc-ZNF131-10:3,MIF-AS1:4,TCONS_00031535 |
| IGFBP3 | 17 | BZRAP1-AS1:19,BZRAP1-AS1:20,LINC00888:6,LINC00987:3,LINC01341:3,lnc-ATF7IP2-3:4,lnc-C12orf39-2:4,lnc-ERICH1-19:8,lnc-FAM27A-4:1,lnc-GBP5-2:6,lnc-GET4-2:1,lnc-KLRG1-3:2,lnc-MARCH7-1:1,lnc-PSMB9-6:4,lnc-TH1L-1:3,TCONS_00049092,TCONS_00049095 |
| NSG1 | 16 | LINC01184:24,lnc-CALHM1-1:5,lnc-CLK3-1:1,lnc-DNAJC8-1:1,lnc-DUOXA1-1:1,lnc-ERICH1-19:8,lnc-FAM160A1-6:3,lnc-FBXO3-3:2,lnc-RGMB-1:2,lnc-RP11-158I9.5.1-2:1,lnc-RP11-712L6.5.1-2:3,lnc-STAMBPL1-1:6,lnc-VPREB1-7:11,lnc-XRCC1-2:1,MIR22HG:3,TTTY15:1 |
| LOC105377808 | 16 | FAM201A:1,GMDS-AS1:11,lnc-AL669831.1-3:42,lnc-BCL2L2-PABPN1-1:2,lnc-C17orf62-6:6,lnc-DOC2B-3:2,lnc-FPR2-1:10,lnc-L3MBTL4-4:1,lnc-MTA2-4:1,lnc-PHLDA3-2:1,lnc-POFUT2-4:1,lnc-RIPK1-1:2,lnc-SYNDIG1L-2:3,lnc-TNFRSF1A-3:1,lnc-VTI1A-1:3,LOH12CR2:1 |
| GOLGA8N | 16 | A2M-AS1:3,LINC00278:4,lnc-CALHM1-1:5,lnc-CLLU1.1-1:10,lnc-ERICH1-19:8,lnc-GOLGA8A-2:2,lnc-KLRG1-3:2,lnc-LILRB5-1:1,lnc-MICA-9:1,lnc-RGMB-1:2,lnc-RP11-408E5.4.1-12:1,lnc-RP11-712L6.5.1-2:3,lnc-TIAF1-3:1,MIF-AS1:4,TCONS_00031535,TTTY15:2 |
| NDE1 | 15 | DANT1:2,lnc-AL669831.1-3:42,lnc-C10orf90-2:2,lnc-C14orf183-1:13,lnc-EXOC2-8:4,lnc-KLC2-2:1,lnc-RP3-369A17.5.1-3:1,lnc-SERPINB6-1:7,lnc-SH2B2-7:1,SLC25A25-AS1:15,TCL6:1,TCONS_00000037,TCONS_00025364,XIST:20,XIST:6 |
| LOC102725016 | 15 | lnc-APOBEC3A-2:1,lnc-ATAD5-3:5,lnc-C12orf39-2:4,lnc-C4orf46-2:2,lnc-COMTD1-6:3,lnc-DHRS1-1:3,lnc-MRFAP1L1-1:1,TCONS_00002746,TCONS_00048629,TCONS_00049090,TCONS_00049091,TCONS_00049092,TCONS_00052885,TP53TG1:8,ZEB1-AS1:11 |
| H1FNT | 15 | FAM201A:1,GMDS-AS1:11,HOXA-AS2:2,lnc-DOC2B-3:2,lnc-INTS2-4:2,lnc-LAG3-1:1,lnc-METRNL-7:4,lnc-NDRG1-1:4,lnc-PCID2-1:2,lnc-PHLDA3-2:1,lnc-PLCG2-5:1,lnc-PLEKHG5-3:1,lnc-POFUT2-4:1,lnc-TNFRSF1A-3:1,lnc-VTI1A-1:3 |
| ARHGEF25 | 15 | LINC00869:34,lnc-CALHM1-1:5,lnc-COG6-7:1,lnc-ERICH1-19:8,lnc-FAM160A1-6:3,lnc-FBXO3-3:2,lnc-GBP5-2:6,lnc-IL22RA2-1:2,lnc-LILRB5-1:1,lnc-LST1-1:5,lnc-MMP23B-5:2,lnc-OR4F29-3:4,lnc-RP11-712L6.5.1-2:3,lnc-YPEL5-5:1,TCONS_00049096 |
| TMEM171 | 14 | BZRAP1-AS1:19,BZRAP1-AS1:20,LINC00936:1,LINC00996:2,lnc-ATF7IP2-3:4,lnc-C12orf39-2:4,lnc-FAM27A-4:1,lnc-FAM27D1.1-2:2,lnc-NBPF15-4:5,lnc-PSMB9-6:4,lnc-RBM28-2:1,PSMD5-AS1:10,TCONS_00049092,TCONS_00049095 |
| PUF60 | 14 | BZRAP1-AS1:19,BZRAP1-AS1:20,LINC00888:6,lnc-AC007952.2.1-2:1,lnc-FAM27D1.1-2:2,lnc-OR4F16-5:16,lnc-TBC1D28-1:1,lnc-TMEM75-10:1,lnc-XRCC1-2:1,PRKCQ-AS1:3,TCONS_00049091,TCONS_00049092,TCONS_00049095,ZNF674-AS1:3 |
| KIAA1324L | 14 | LINC00623:19,LINC00987:3,lnc-C12orf39-2:4,lnc-DHRS1-1:3,lnc-GET4-1:2,lnc-HAO2-2:2,lnc-KLRG1-3:2,lnc-MARCH7-1:1,lnc-MRFAP1L1-1:1,lnc-TRIM41-1:6,TCONS_00002746,TCONS_00002748,TP53TG1:8,ZEB1-AS1:11 |
| AHI1 | 14 | A2M-AS1:3,LINC00888:6,LINC01184:24,lnc-ATF7IP2-7:1,lnc-C19orf52-1:1,lnc-C3orf79-2:1,lnc-GOLGA8IP-2:1,lnc-GUSB-12:1,lnc-KLRG1-3:2,lnc-LILRB5-1:1,lnc-RGMB-1:2,lnc-ZNF131-10:3,MIF-AS1:4,TCONS_00031535 |
| UTY | 13 | lnc-CLLU1.1-1:10,lnc-CYorf15A.1-2:6,lnc-GOLGA8IP-2:1,lnc-KDM5D-4:1,lnc-RP11-408E5.4.1-12:1,lnc-RP11-712L6.5.1-2:3,lnc-TBL1Y-6:2,lnc-USP9Y-3:1,lnc-UTY-1:53,MIF-AS1:4,TTTY15:2,TTTY15:5,TTTY15:6 |
| KDM5D | 13 | lnc-CLLU1.1-1:10,lnc-GOLGA8A-2:2,lnc-GOLGA8IP-2:1,lnc-HSFY2-13:1,lnc-KDM5D-4:1,lnc-MICA-9:1,lnc-MMP23B-5:2,lnc-RP11-408E5.4.1-12:1,lnc-USP9Y-3:1,MIF-AS1:4,TTTY15:2,TTTY15:5,TTTY15:6 |
| HIP1 | 13 | BISPR:14,FAM74A6:1,lnc-AC006455.1-10:1,lnc-AC008686.1-6:1,lnc-BRI3-2:3,lnc-FAM156B-1:1,lnc-MARCH7-1:2,lnc-MTA2-4:1,lnc-NAT1-6:1,lnc-SFPQ-2:4,lnc-TAGLN2-1:1,lnc-TRPM7-1:1,NDUFA6-AS1:5 |
| FAM72D | 13 | CD27-AS1:5,DICER1-AS1:2,LINC00339:21,lnc-AL359853.2-2:1,lnc-ATL3-2:1,lnc-CDK2AP1-1:4,lnc-FAM106A-2:15,lnc-KLHL9-1:1,lnc-MMP23B-5:2,lnc-TMUB2-1:4,lnc-ZNF100-3:1,TCONS_00052887,TCONS_00052889 |
| ZFY | 12 | LINC00278:1,LINC00278:4,lnc-GOLGA8A-2:2,lnc-MICA-9:1,lnc-MMP23B-5:2,lnc-RP11-712L6.5.1-2:3,MIF-AS1:4,TTTY14:13,TTTY15:1,TTTY15:2,TTTY15:5,TTTY15:6 |
| SPDYE18 | 12 | BISPR:14,lnc-C15orf39-1:1,lnc-C16orf72-7:1,lnc-CADM4-2:1,lnc-FUK-2:1,lnc-GPR152-1:7,lnc-LRCH4-2:1,lnc-POFUT2-5:1,lnc-TACC2-3:3,lnc-TNFRSF1A-3:1,lnc-TNNT1-1:1,UGDH-AS1:6 |
| B4GAT1 | 12 | BZRAP1-AS1:19,BZRAP1-AS1:20,lnc-AAGAB-3:2,lnc-AC007952.2.1-2:1,lnc-ANKRD18B-6:4,lnc-CLK3-1:1,lnc-ERICH1-19:8,lnc-MARCH7-1:1,lnc-OR4F16-5:16,lnc-RGMB-1:2,lnc-RP11-796G6.2.1-4:2,TCONS_00049082/ NEAT1:14 |
| TARP | 11 | LINC00888:6,LINC01184:20,lnc-AL359853.2-2:1,lnc-AMPH-10:7,lnc-KLHL9-1:1,lnc-TH1L-1:3,lnc-TRIM41-1:6,lnc-ZNF100-3:1,lnc-ZNF71-4:1,TCONS_00031535,TPT1-AS1:16 |
| SUSD2 | 11 | lnc-C1orf132-1:5,lnc-C1orf138-1:1,lnc-LRCH4-2:1,lnc-SEC11A-2:1,lnc-TNNT1-1:1,lnc-TRUB2-7:1,lnc-ZSWIM7-3:1,PCBP1-AS1:198,PSMB8-AS1:3,TCL6:1,TCONS_00000037 |
| SPON1 | 11 | BZRAP1-AS1:19,BZRAP1-AS1:20,BZRAP1-AS1:21,LINC00888:6,lnc-GBP5-2:6,lnc-GET4-1:2,lnc-MARCH7-1:1,lnc-TBC1D28-1:1,lnc-TH1L-1:3,lnc-VPREB1-7:11,PSMD5-AS1:22 |
| SFN | 11 | FAM201A:1,lnc-BCL2L2-PABPN1-1:2,lnc-C16orf72-7:1,lnc-C1orf186-3:1,lnc-DOC2B-3:2,lnc-METRNL-7:4,lnc-PLEKHG5-3:1,lnc-POFUT2-4:1,lnc-SATB1-8:5,lnc-TNFRSF1A-3:1,lnc-VTI1A-1:3 |
| PDGFRA | 11 | LINC00888:6,lnc-AL359853.2-2:1,lnc-DMKN-1:9,lnc-DPH5-1:3,lnc-MRFAP1L1-1:1,lnc-YPEL5-5:1,PRKCQ-AS1:3,TCONS_00049091,TCONS_00052884,TCONS_00052887,TCONS_00052889 |
| CLDN22 | 11 | LBX2-AS1:2,lnc-C10orf90-2:2,lnc-C11orf94-1:1,lnc-C17orf109-3:1,lnc-CLIC3-2:1,lnc-CRYBA4-1:55,lnc-CSNK1D-2:9,lnc-RP11-389E17.1.1-1:1,lnc-TAF13-2:1,lnc-TTR-3:1,SNAI3-AS1:16 |
| ADAMTS1 | 11 | LINC00888:6,lnc-CLK3-1:1,lnc-LILRB5-1:1,lnc-OR4F29-3:4,lnc-RP11-158I9.5.1-2:1,lnc-STAMBPL1-1:6,lnc-TMEM75-10:1,lnc-VPREB1-7:11,lnc-XRCC1-2:1,MIR22HG:3,TCONS_00049082 |
| NTHL1 | 10 | A2M-AS1:3,lnc-ERICH1-19:8,lnc-FAM160A1-6:3,lnc-GET4-1:2,lnc-GOLGA8A-2:2,lnc-HAO2-2:2,lnc-RGMB-1:2,lnc-TMEM75-10:1,MIF-AS1:4,PSMD5-AS1:22 |
| ITGA2 | 10 | lnc-C11orf54-2:1,lnc-CYorf15A.1-2:6,lnc-GOLGA8IP-2:1,lnc-HSFY2-13:1,lnc-IL22RA2-1:2,lnc-KDM5D-4:1,lnc-RP11-408E5.4.1-12:1,lnc-USP9Y-3:1,MIF-AS1:4,TTTY15:2 |
| HLA-G | 10 | FAM201A:1,GMDS-AS1:11,lnc-DOC2B-3:2,lnc-FAM200B-1:13,lnc-METRNL-7:4,lnc-PLCG2-5:1,lnc-PLEKHG5-3:1,lnc-RP11-389E17.1.1-1:1,lnc-SATB1-8:5,SCHLAP1:3 |
| CLIC5 | 10 | A2M-AS1:3,LINC00339:21,lnc-CLLU1.1-1:10,lnc-DPH5-1:3,lnc-DUOXA1-1:1,lnc-ERICH1-19:8,lnc-MMP23B-5:2,lnc-TMUB2-1:4,lnc-YPEL5-5:1,lnc-ZNF737-3:5 |
| C14orf183 | 10 | FAM201A:1,lnc-AC022098.1-1:5,lnc-BCL2L2-PABPN1-1:2,lnc-C17orf62-6:6,lnc-FPR2-1:10,lnc-METRNL-7:4,lnc-PCID2-1:2,lnc-PLCG2-5:1,lnc-PLEKHG5-3:1,SNAI3-AS1:16 |
| USP9Y | 9 | lnc-CLLU1.1-1:10,lnc-GOLGA8IP-2:1,lnc-KDM5D-4:1,lnc-RP11-408E5.4.1-12:1,lnc-RP11-712L6.5.1-2:3,lnc-USP9Y-3:1,TTTY15:2,TTTY15:5,TTTY15:6 |
| NFE2L3 | 9 | A2M-AS1:3,LINC00278:4,LINC00987:3,lnc-C8orf31-1:1,lnc-CALHM1-1:5,lnc-ERICH1-19:8,lnc-GOLGA8A-2:2,lnc-RHOXF1-3:15,lnc-RHOXF1-3:16 |
| FAM60A | 9 | lnc-AC092329.1-1:24,lnc-C4orf46-2:2,lnc-COMTD1-6:3,lnc-MRFAP1L1-1:1,lnc-TRIM41-1:6,TCONS_00002746,TP53TG1:8,TPT1-AS1:16,ZEB1-AS1:11 |
| TIAM2 | 8 | lnc-AC008686.1-6:1,lnc-AL669831.1-3:42,lnc-KLC2-2:1,lnc-LRCH4-2:1,lnc-MARCH7-1:2,TCONS_00000037,TCONS_00044060,TCONS_00052323 |
| PTPRM | 8 | lnc-C12orf39-2:4,lnc-C4orf46-2:2,lnc-COMTD1-6:3,lnc-DHRS1-1:3,lnc-TBC1D28-1:1,TCONS_00002746,TCONS_00049092,ZEB1-AS1:11 |
| GRM2 | 8 | lnc-BCL2L2-PABPN1-1:2,lnc-C16orf72-7:1,lnc-DPY19L3-1:1,lnc-GSR-3:1,lnc-RIPK1-1:2,lnc-SLC26A11-1:1,lnc-SLC7A6OS-4:2,LOH12CR2:1 |
| CATSPERG | 8 | lnc-BCL2L2-PABPN1-1:2,lnc-DPY19L3-1:1,lnc-GSR-3:1,lnc-IFT52-1:2,lnc-L3MBTL4-4:1,lnc-OR2AT4-1:1,lnc-TTR-3:1,LOH12CR2:1 |
| NUDT11 | 7 | BZRAP1-AS1:19,lnc-CALHM1-1:5,lnc-FAM160A1-6:3,lnc-FBXO3-3:2,lnc-GNLY-2:3,lnc-STAMBPL1-1:6,TCONS_00049082 |
| ME3 | 7 | lnc-AC092329.1-1:24,lnc-GET4-1:2,lnc-KLRG1-3:2,lnc-KLRG1-5:1,TCONS_00031535,TP53TG1:8,ZEB1-AS1:11 |
| FAM127B | 7 | lnc-BCL2L2-PABPN1-1:2,lnc-CEP170-9:2,lnc-DPY19L3-1:1,lnc-MAP3K3-2:2,lnc-PRKRIR-1:5,lnc-VTI1A-1:3,LOH12CR2:1 |
| ROR2 | 6 | LINC00278:1,LINC00278:4,lnc-CALHM1-1:5,lnc-KDM5D-1:1,lnc-RP11-712L6.5.1-2:3,lnc-TRIML1-9:1 |
| RNF225 | 6 | lnc-BCL9L-1:1,lnc-CCT5-2:1,lnc-EXOC2-8:4,lnc-EXT1-1:1,lnc-LIPI-9:1,lnc-SP6-1:1 |
| PBX2 | 6 | APTR:11,lnc-AP000769.1-1:2,lnc-C1orf186-3:1,lnc-CSNK1D-2:9,lnc-STAB1-3:1,NDUFA6-AS1:5 |
| NFXL1 | 6 | lnc-GBP5-2:6,lnc-GNLY-2:3,lnc-TH1L-1:3,lnc-XRCC1-2:1,lnc-YPEL5-5:1,lnc-ZNF100-2:2 |
| KIF2C | 6 | LINC01184:24,lnc-AL901608.1-3:27,lnc-CLK3-1:1,lnc-FBXO3-3:2,MIR22HG:3,TCONS_00049082 |
| FAT4 | 6 | lnc-COG6-7:1,lnc-ERICH1-19:8,lnc-RGMB-1:2,lnc-RP11-158I9.5.1-2:1,lnc-RP11-712L6.5.1-2:3,lnc-TIAF1-3:1 |
| ECH1 | 6 | lnc-METRNL-7:4,lnc-MTA2-4:1,lnc-PCID2-1:2,lnc-PLEKHG5-3:1,lnc-SYNDIG1L-2:3,lnc-TNFRSF1A-3:1 |
| CYB5D1 | 6 | lnc-AP000769.1-1:2,lnc-FUK-2:1,lnc-GPR152-1:7,lnc-PKLR-7:1,lnc-TMEM120B-4:3,lnc-TNFRSF1A-3:1 |
| CADM4 | 6 | lnc-AC022098.1-1:5,lnc-AP000769.1-1:2,lnc-C17orf77-1:6,lnc-LONRF2-3:1,lnc-TSSC4-5:2,NDUFA6-AS1:5 |
| VARS2 | 5 | lnc-FXYD4-4:2,lnc-GTPBP1-1:1,lnc-MTR-4:1,lnc-NDUFB8-7:1,lnc-SIX5-1:1 |
| MADCAM1 | 5 | LINC00888:6,lnc-ANKRD18B-6:4,lnc-COMTD1-6:3,lnc-FAM113B-5:5,lnc-TMEM75-10:1 |
| ANKHD1-EIF4EBP3 | 5 | lnc-CLDN23-3:1,lnc-GTPBP1-1:1,lnc-HEATR2-3:1,lnc-SIX5-1:1,TCONS_00052860 |
| ZNF215 | 4 | LINC00888:6,lnc-LILRB5-1:1,lnc-PXDC1-14:4,lnc-XRCC1-2:1 |
| MXRA8 | 4 | lnc-AC008686.1-6:1,lnc-BRI3-2:3,lnc-MTA2-4:1,NDUFA6-AS1:5 |
| FLJ44635 | 4 | lnc-C4orf36-2:5,lnc-DOC2B-3:2,lnc-METRNL-7:4,lnc-MTA2-4:1 |
| FAM110B | 4 | lnc-C8orf31-1:1,lnc-CALHM1-1:5,lnc-FAM160A1-6:3,lnc-RP11-712L6.5.1-2:3 |
| CLEC18A | 4 | LBX2-AS1:2,lnc-FAM156B-1:1,lnc-SBDS-4:1,NDUFA6-AS1:5 |
| CLDN24 | 4 | LINC00920:3,lnc-CLIC3-2:1,lnc-MOGAT2-3:1,PSMB8-AS1:5 |
| TGM3 | 3 | lnc-CLDN9-1:1,lnc-MTA2-4:1,NDUFA6-AS1:5 |
| SGCD | 3 | TCONS_00002746,TCONS_00049092,ZEB1-AS1:11 |
| OTX1 | 3 | lnc-C14orf183-1:13,lnc-KCND3-3:1,TCONS_00052323 |
| OLR1 | 3 | lnc-FAM200B-1:13,lnc-LAG3-1:1,lnc-SLC12A8-1:1 |
| GSTM3 | 3 | lnc-PRKRIR-1:5,lnc-SAMD8-1:1,lnc-TTR-3:1 |
| DDR2 | 3 | DICER1-AS1:2,lnc-CLK3-1:1,lnc-EXOC3-3:1 |
| CHI3L2 | 3 | A2M-AS1:3,lnc-COMTD1-6:3,lnc-ZNF100-3:1 |
| CFAP45 | 3 | lnc-C1orf132-1:5,lnc-PCBP3-3:9,TCONS_00000037 |
| CCNB1 | 3 | lnc-AMPH-2:2,lnc-DNAJC8-1:1,lnc-RP11-712L6.5.1-2:3 |
| ATF6B | 3 | LINC00278:1,lnc-DHRS1-1:3,MIF-AS1:4 |
| ALAS2 | 3 | LINC00278:1,lnc-RGMB-1:2,MIF-AS1:4 |
| AATK | 3 | LINC00894:20,lnc-DFNB59-2:14,lnc-RP11-389E17.1.1-1:1 |
| TMSB4Y | 2 | lnc-GOLGA8IP-2:1,lnc-MMP23B-5:2 |
| KCNC3 | 2 | lnc-METTL14-2:3,lnc-NMRAL1-2:1 |
| FAM153B | 2 | LBX2-AS1:2,lnc-C1orf186-3:1 |
| DDX3Y | 2 | lnc-USP9Y-4:1,MIF-AS1:4 |
| ASPM | 2 | lnc-CALHM1-1:5,lnc-RP11-158I9.5.1-2:1 |
| ARPIN | 2 | lnc-C11orf54-2:1,lnc-FAM160A1-6:3 |
| ZNF695 | 1 | lnc-UBL5-5:1 |
| SDK2 | 1 | TCONS_00025364 |
| S100P | 1 | lnc-MMP23B-5:2 |
| REC8 | 1 | lnc-TNFRSF1A-3:1 |
| RASA4B | 1 | lnc-MARCH7-1:2 |
| RAG1 | 1 | lnc-AL901608.1-3:27 |
| RAD51AP1 | 1 | lnc-PTP4A2-3:4 |
| PPP1R10 | 1 | lnc-FSD1L-3:1 |
| MAP1LC3B2 | 1 | lnc-STAB1-3:1 |
| LOC105372343 | 1 | BISPR:14 |
| LOC101060181 | 1 | lnc-SERPINB6-1:7 |
| HIST1H3J | 1 | lnc-AAGAB-3:2 |
| HBD | 1 | lnc-MMP23B-5:2 |
| DPCD | 1 | lnc-ERICH1-19:8 |
| CFH | 1 | lnc-ERICH1-19:8 |
| APOBEC3A_B | 1 | lnc-SERPINB6-1:7 |

^a^ Under the prerequisite of coexpression (Pearson’s correlation coefficient >0.8) of a lncRNA and a gene in QDC population, the *trans*-acting lncRNAs could directly bind to genes’ mRNAs.

**Table S9 Detailed the *cis*-regulation pairs between the QDC-specific differential genes and lncRNAs in QDC population**

| **gene** | **ncrna ^a^** | **Correlation Coefficient ^b^** | ***P*-value** | **gene_chrom** | **gene_start** | **gene_end** | **gene strand** | **distance** | **ncrna_chrom** | **ncrna_start** | **ncrna_end** | **ncrna strand** |
| --- | --- | --- | --- | --- | --- | --- | --- | --- | --- | --- | --- | --- |
| B4GAT1 | NEAT1:14 | -0.937938422 | 0.001782037 | NC_000011.10 | 66345372 | 66347690 | . | 918846 | NC_000011.10 | 65424330 | 65426526 | + |
| CFAP45 | TCONS_00001685 | 0.8757092 | 0.009772458 | NC_000001.11 | 159872364 | 159900116 | . | 22338 | NC_000001.11 | 159922454 | 159937702 | - |
| CLDN22 | lnc-ING2-5:2 | -0.919970906 | 0.003331927 | NC_000004.12 | 183318067 | 183320774 | . | 346181 | NC_000004.12 | 183666955 | 183677538 | + |
| CLDN9 | lnc-CLDN9-1:1 | 0.922855739 | 0.003044559 | NC_000016.10 | 3012456 | 3014505 | . | 3335 | NC_000016.10 | 3004682 | 3009121 | + |
| CLDN9 | lnc-IL32-1:3 | 0.885793864 | 0.007953816 | NC_000016.10 | 3012456 | 3014505 | . | 38870 | NC_000016.10 | 3053375 | 3054719 | + |
| DDX3Y | lnc-USP9Y-3:1 | 0.992951083 | 7.98E-06 | NC_000024.10 | 12904108 | 12920478 | . | 43269 | NC_000024.10 | 12856812 | 12860839 | + |
| DDX3Y | lnc-USP9Y-4:1 | 0.984313374 | 5.87E-05 | NC_000024.10 | 12904108 | 12920478 | . | 740 | NC_000024.10 | 12904848 | 12909696 | + |
| DDX3Y | lnc-UTY-1:53 | 0.937836174 | 0.001789286 | NC_000024.10 | 12904108 | 12920478 | . | 472673 | NC_000024.10 | 13393151 | 13479504 | - |
| DDX3Y | TTTY15:1 | 0.97243594 | 0.000238709 | NC_000024.10 | 12904108 | 12920478 | . | 213667 | NC_000024.10 | 12662333 | 12690441 | + |
| DDX3Y | TTTY15:2 | 0.998180601 | 2.71E-07 | NC_000024.10 | 12904108 | 12920478 | . | 211875 | NC_000024.10 | 12662333 | 12692233 | + |
| DDX3Y | TTTY15:5 | 0.933176656 | 0.002138196 | NC_000024.10 | 12904108 | 12920478 | . | 211885 | NC_000024.10 | 12662366 | 12692223 | + |
| DDX3Y | TTTY15:6 | 0.935013783 | 0.001996261 | NC_000024.10 | 12904108 | 12920478 | . | 211884 | NC_000024.10 | 12662366 | 12692224 | + |
| EIF1AY | lnc-CYorf15A.1-2:6 | 0.990096378 | 1.86E-05 | NC_000024.10 | 20575711 | 20593154 | . | 985864 | NC_000024.10 | 19567828 | 19589847 | + |
| EIF1AY | lnc-CYorf15A.1-2:7 | 0.992540048 | 9.20E-06 | NC_000024.10 | 20575711 | 20593154 | . | 985292 | NC_000024.10 | 19588569 | 19590419 | + |
| EIF1AY | lnc-CYorf15A.1-3:1 | 0.999057386 | 5.24E-08 | NC_000024.10 | 20575711 | 20593154 | . | 976954 | NC_000024.10 | 19598187 | 19598757 | + |
| EIF1AY | lnc-HSFY2-13:1 | 0.992134904 | 1.05E-05 | NC_000024.10 | 20575711 | 20593154 | . | 865105 | NC_000024.10 | 19709699 | 19710606 | - |
| EIF1AY | lnc-KDM5D-1:1 | 0.949563023 | 0.0010678 | NC_000024.10 | 20575711 | 20593154 | . | 881105 | NC_000024.10 | 19691940 | 19694606 | - |
| EIF1AY | lnc-KDM5D-4:1 | 0.985577708 | 4.76E-05 | NC_000024.10 | 20575711 | 20593154 | . | 51278 | NC_000024.10 | 20519947 | 20524433 | - |
| EIF1AY | TTTY10:1 | 0.993322346 | 6.97E-06 | NC_000024.10 | 20575711 | 20593154 | . | 56483 | NC_000024.10 | 20465667 | 20519228 | - |
| ELF3 | lnc-PHLDA3-2:1 | 0.918983776 | 0.003433757 | NC_000001.11 | 202010519 | 202017188 | . | 549564 | NC_000001.11 | 201455641 | 201460955 | - |
| HSD17B13 | lnc-C4orf36-2:5 | 0.810548569 | 0.027016882 | NC_000004.12 | 87303789 | 87322906 | . | 368939 | NC_000004.12 | 86924893 | 86934850 | - |
| KCNK7 | lnc-AP000769.1-1:2 | 0.984599482 | 5.61E-05 | NC_000011.10 | 65592855 | 65595996 | . | 139207 | NC_000011.10 | 65451059 | 65453648 | + |
| KDM5D | lnc-CYorf15A.1-2:6 | 0.99819374 | 2.66E-07 | NC_000024.10 | 19705415 | 19744939 | . | 115568 | NC_000024.10 | 19567828 | 19589847 | + |
| KDM5D | lnc-CYorf15A.1-2:7 | 0.996059477 | 1.87E-06 | NC_000024.10 | 19705415 | 19744939 | . | 114996 | NC_000024.10 | 19588569 | 19590419 | + |
| KDM5D | lnc-CYorf15A.1-3:1 | 0.987976482 | 3.03E-05 | NC_000024.10 | 19705415 | 19744939 | . | 106658 | NC_000024.10 | 19598187 | 19598757 | + |
| KDM5D | lnc-HSFY2-13:1 | 0.972442649 | 0.000238564 | NC_000024.10 | 19705415 | 19744939 | . | 4284 | NC_000024.10 | 19709699 | 19710606 | - |
| KDM5D | lnc-KDM5D-1:1 | 0.977994179 | 0.000136351 | NC_000024.10 | 19705415 | 19744939 | . | 10809 | NC_000024.10 | 19691940 | 19694606 | - |
| KDM5D | lnc-KDM5D-4:1 | 0.975531938 | 0.000177515 | NC_000024.10 | 19705415 | 19744939 | . | 775008 | NC_000024.10 | 20519947 | 20524433 | - |
| KDM5D | TTTY10:1 | 0.962614642 | 0.000508691 | NC_000024.10 | 19705415 | 19744939 | . | 720728 | NC_000024.10 | 20465667 | 20519228 | - |
| KDM5D | TTTY14:13 | 0.997151655 | 8.30E-07 | NC_000024.10 | 19705415 | 19744939 | . | 661865 | NC_000024.10 | 19041345 | 19043550 | - |
| KIAA1324L | TP53TG1:8 | 0.892526027 | 0.006858639 | NC_000007.14 | 86876906 | 87059698 | . | 265651 | NC_000007.14 | 87325349 | 87345515 | - |
| KLRB1 | A2M-AS1:3 | 0.847488219 | 0.016042582 | NC_000012.12 | 9595274 | 9607901 | . | 528567 | NC_000012.12 | 9065184 | 9066707 | + |
| KLRB1 | lnc-CLEC2D-8:4 | 0.87876163 | 0.00919915 | NC_000012.12 | 9595274 | 9607901 | . | 34970 | NC_000012.12 | 9642871 | 9658414 | + |
| KLRC1 | lnc-CLEC2D-8:4 | 0.835012508 | 0.019389874 | NC_000012.12 | 10442264 | 10454685 | . | 783850 | NC_000012.12 | 9642871 | 9658414 | + |
| KLRG1 | A2M-AS1:3 | 0.980601444 | 9.96E-05 | NC_000012.12 | 8989625 | 9010747 | . | 54437 | NC_000012.12 | 9065184 | 9066707 | + |
| KLRG1 | LINC00987:3 | 0.909406506 | 0.004516341 | NC_000012.12 | 8989625 | 9010747 | . | 229255 | NC_000012.12 | 9240002 | 9243049 | + |
| KLRG1 | lnc-KLRG1-3:2 | 0.930444872 | 0.00235993 | NC_000012.12 | 8989625 | 9010747 | . | 243188 | NC_000012.12 | 9253935 | 9287255 | + |
| KLRG1 | lnc-KLRG1-5:1 | 0.940876838 | 0.001581073 | NC_000012.12 | 8989625 | 9010747 | . | 124336 | NC_000012.12 | 9135083 | 9135591 | + |
| KLRG1 | TCONS_00031535 | 0.964901949 | 0.000434957 | NC_000012.12 | 8989625 | 9010747 | . | 187196 | NC_000012.12 | 9197943 | 9288560 | - |
| LOC101927789 | lnc-AP000769.1-1:2 | 0.923595241 | 0.00297333 | NC_000011.10 | 65454421 | 65466720 | . | 773 | NC_000011.10 | 65451059 | 65453648 | + |
| LOC105372343 | BISPR:14 | 0.820991455 | 0.023586688 | NC_000019.10 | 17389069 | 17394158 | . | 11956 | NC_000019.10 | 17406114 | 17414347 | + |
| NEO1 | lnc-CELF6-1:5 | 0.826120638 | 0.021997443 | NC_000015.10 | 73051715 | 73305206 | . | 705833 | NC_000015.10 | 72343856 | 72345882 | - |
| NFXL1 | lnc-CORIN-1:1 | 0.970262143 | 0.000288253 | NC_000004.12 | 47847233 | 47914667 | . | 2894 | NC_000004.12 | 47840121 | 47844339 | - |
| NTHL1 | lnc-CLDN9-1:1 | -0.886397 | 0.007851858 | NC_000016.10 | 2039815 | 2047866 | . | 956816 | NC_000016.10 | 3004682 | 3009121 | + |
| OLR1 | lnc-CLEC2D-8:4 | -0.892666404 | 0.006836799 | NC_000012.12 | 10158300 | 10176261 | . | 499886 | NC_000012.12 | 9642871 | 9658414 | + |
| PTPRM | lnc-L3MBTL4-4:1 | -0.915430842 | 0.003815214 | NC_000018.10 | 7567316 | 8406861 | . | 594327 | NC_000018.10 | 6972707 | 6972989 | - |
| PVRL2 | lnc-SIX5-1:1 | 0.837870116 | 0.018591274 | NC_000019.10 | 44846136 | 44889228 | . | 880727 | NC_000019.10 | 45769955 | 45771365 | - |
| RPS4Y1 | LINC00278:1 | 0.985834625 | 4.55E-05 | NC_000024.10 | 2841582 | 2866956 | . | 135955 | NC_000024.10 | 3002911 | 3097217 | + |
| RPS4Y1 | LINC00278:4 | 0.951799685 | 0.000954489 | NC_000024.10 | 2841582 | 2866956 | . | 136041 | NC_000024.10 | 3002997 | 3102272 | + |
| SLC7A8 | lnc-BCL2L2-PABPN1-1:1 | -0.908494512 | 0.00462853 | NC_000014.9 | 23125295 | 23183660 | . | 123142 | NC_000014.9 | 23306802 | 23311757 | + |
| SLC7A8 | lnc-BCL2L2-PABPN1-1:2 | 0.828107165 | 0.021398711 | NC_000014.9 | 23125295 | 23183660 | . | 123142 | NC_000014.9 | 23306802 | 23326158 | + |
| SLC7A9 | lnc-DPY19L3-1:1 | 0.82700954 | 0.021728376 | NC_000019.10 | 32830511 | 32870167 | . | 402450 | NC_000019.10 | 32405748 | 32428061 | + |
| SLX1B | lnc-LAT-1:9 | 0.92887393 | 0.002493296 | NC_000016.10 | 29454501 | 29458224 | . | 464046 | NC_000016.10 | 28989576 | 28990455 | + |
| TARP | lnc-AMPH-10:7 | 0.910056231 | 0.004437393 | NC_000007.14 | 38257879 | 38305646 | . | 11393 | NC_000007.14 | 38269272 | 38271302 | - |
| TARP | lnc-AMPH-5:1 | 0.813150984 | 0.026137522 | NC_000007.14 | 38257879 | 38305646 | . | 29394 | NC_000007.14 | 38335040 | 38335514 | - |
| TEAD2 | lnc-TULP2-2:1 | 0.847566553 | 0.016022699 | NC_000019.10 | 49340595 | 49362457 | . | 464194 | NC_000019.10 | 48876111 | 48876401 | - |
| TMSB4Y | lnc-USP9Y-3:1 | 0.991062234 | 1.44E-05 | NC_000024.10 | 13703567 | 13706024 | . | 842728 | NC_000024.10 | 12856812 | 12860839 | + |
| TMSB4Y | lnc-USP9Y-4:1 | 0.98778442 | 3.15E-05 | NC_000024.10 | 13703567 | 13706024 | . | 793871 | NC_000024.10 | 12904848 | 12909696 | + |
| TMSB4Y | lnc-UTY-1:53 | 0.937306657 | 0.001827103 | NC_000024.10 | 13703567 | 13706024 | . | 224063 | NC_000024.10 | 13393151 | 13479504 | - |
| USP9Y | lnc-USP9Y-3:1 | 0.988934525 | 2.46E-05 | NC_000024.10 | 12701231 | 12860844 | . | 5 | NC_000024.10 | 12856812 | 12860839 | + |
| USP9Y | lnc-USP9Y-4:1 | 0.976295676 | 0.000164053 | NC_000024.10 | 12701231 | 12860844 | . | 44004 | NC_000024.10 | 12904848 | 12909696 | + |
| USP9Y | lnc-UTY-1:53 | 0.92829453 | 0.002543574 | NC_000024.10 | 12701231 | 12860844 | . | 532307 | NC_000024.10 | 13393151 | 13479504 | - |
| USP9Y | TTTY15:1 | 0.980726972 | 9.80E-05 | NC_000024.10 | 12701231 | 12860844 | . | 10790 | NC_000024.10 | 12662333 | 12690441 | + |
| USP9Y | TTTY15:2 | 0.999791229 | 1.21E-09 | NC_000024.10 | 12701231 | 12860844 | . | 8998 | NC_000024.10 | 12662333 | 12692233 | + |
| USP9Y | TTTY15:5 | 0.934441187 | 0.002039885 | NC_000024.10 | 12701231 | 12860844 | . | 9008 | NC_000024.10 | 12662366 | 12692223 | + |
| USP9Y | TTTY15:6 | 0.936922206 | 0.001854853 | NC_000024.10 | 12701231 | 12860844 | . | 9007 | NC_000024.10 | 12662366 | 12692224 | + |
| UTY | lnc-USP9Y-3:1 | 0.991074956 | 1.44E-05 | NC_000024.10 | 13233920 | 13480670 | . | 373081 | NC_000024.10 | 12856812 | 12860839 | + |
| UTY | lnc-USP9Y-4:1 | 0.982062358 | 8.20E-05 | NC_000024.10 | 13233920 | 13480670 | . | 324224 | NC_000024.10 | 12904848 | 12909696 | + |
| UTY | lnc-UTY-1:53 | 0.932551385 | 0.002187816 | NC_000024.10 | 13233920 | 13480670 | . | 1166 | NC_000024.10 | 13393151 | 13479504 | - |
| UTY | TTTY15:1 | 0.975042124 | 0.000186484 | NC_000024.10 | 13233920 | 13480670 | . | 543479 | NC_000024.10 | 12662333 | 12690441 | + |
| UTY | TTTY15:2 | 0.998028307 | 3.31E-07 | NC_000024.10 | 13233920 | 13480670 | . | 541687 | NC_000024.10 | 12662333 | 12692233 | + |
| UTY | TTTY15:5 | 0.927946896 | 0.002574023 | NC_000024.10 | 13233920 | 13480670 | . | 541697 | NC_000024.10 | 12662366 | 12692223 | + |
| UTY | TTTY15:6 | 0.929938785 | 0.002402425 | NC_000024.10 | 13233920 | 13480670 | . | 541696 | NC_000024.10 | 12662366 | 12692224 | + |
| ZFY | LINC00278:1 | 0.985925837 | 4.48E-05 | NC_000024.10 | 2934416 | 2982508 | . | 20403 | NC_000024.10 | 3002911 | 3097217 | + |
| ZFY | LINC00278:4 | 0.965940254 | 0.000403726 | NC_000024.10 | 2934416 | 2982508 | . | 20489 | NC_000024.10 | 3002997 | 3102272 | + |

^a^ The common differential lncRNAs appeared in both case populations (QDC and PQDS of CSG) were indicated in red font.

^b^ *Cis*-acting lncRNAs usually control their neighboring genes, so, for each coexpression pair (|Pearson’s correlation coefficient| >0.8) of gene and lncRNA, if the gene is closed (less than 100 kb) to the lncRNA in a genome, it was considered as a candidate target of the cis-acting lncRNA

**Table S10 Interactions between PQDS-specific genes coded proteins**

| **node1** | **node2** | **Homology ^a^** | **Coexpression ^b^** | **Experimentally determined interaction ^c^** | **Database annotated ^d^** | **Automated textmining ^e^** | **Combined score ^f^** |
| --- | --- | --- | --- | --- | --- | --- | --- |
| ASMTL | NR1I2 | 0.988 | 0.771 | 0.8 | 0.9 | 0.828 | 0.995 |
| C4A | C4BPA | 0 | 0.088 | 0.379 | 0.9 | 0.654 | 0.977 |
| C4A | C4BPB | 0 | 0 | 0 | 0.9 | 0.715 | 0.97 |
| C4A | SPP1 | 0 | 0.096 | 0 | 0.9 | 0.64 | 0.964 |
| C4A | VTN | 0 | 0 | 0 | 0.9 | 0.641 | 0.962 |
| C4B | C4A | 0 | 0.111 | 0.064 | 0.9 | 0.406 | 0.944 |
| C4B | C4BPA | 0.642 | 0.146 | 0 | 0.9 | 0.711 | 0.933 |
| C4B | C4BPB | 0.746 | 0.061 | 0 | 0.9 | 0.911 | 0.932 |
| C4B | VTN | 0 | 0.062 | 0.064 | 0.9 | 0.311 | 0.931 |
| C4BPA | C4BPB | 0 | 0.088 | 0 | 0.9 | 0.188 | 0.919 |
| C4BPA | VTN | 0.743 | 0 | 0 | 0.9 | 0.29 | 0.916 |
| CACNA1C | GNAO1 | 0.836 | 0.047 | 0 | 0.9 | 0.42 | 0.913 |
| CLDN7 | CRB3 | 0.543 | 0.076 | 0 | 0.9 | 0.05 | 0.904 |
| CLEC4C | PTPRB | 0 | 0.062 | 0 | 0.9 | 0 | 0.902 |
| COL10A1 | COL5A3 | 0 | 0.062 | 0 | 0 | 0.718 | 0.724 |
| COL2A1 | ADAMTS2 | 0 | 0.116 | 0 | 0.6 | 0.105 | 0.656 |
| COL2A1 | COL10A1 | 0 | 0 | 0 | 0 | 0.641 | 0.641 |
| COL2A1 | COL5A3 | 0 | 0 | 0 | 0 | 0.591 | 0.591 |
| COL5A3 | ADAMTS2 | 0 | 0 | 0 | 0 | 0.58 | 0.58 |
| CPLX2 | GNAO1 | 0 | 0.17 | 0 | 0 | 0.514 | 0.579 |
| CPXM1 | ADAM12 | 0 | 0 | 0 | 0 | 0.573 | 0.573 |
| FAM83D | CAPN8 | 0 | 0.139 | 0 | 0 | 0.513 | 0.562 |
| PLCXD1 | ASMTL | 0 | 0 | 0 | 0 | 0.562 | 0.562 |
| PRG4 | COL10A1 | 0.623 | 0 | 0.475 | 0 | 0.407 | 0.55 |
| PRG4 | COL2A1 | 0 | 0 | 0 | 0 | 0.513 | 0.513 |
| RXRB | NR1I2 | 0 | 0.265 | 0 | 0 | 0.348 | 0.501 |
| SEMA5A | ADAMTS2 | 0 | 0 | 0 | 0 | 0.483 | 0.483 |
| SPP1 | COL10A1 | 0 | 0.062 | 0 | 0 | 0.457 | 0.469 |
| SPP1 | COL2A1 | 0 | 0.12 | 0.266 | 0 | 0.194 | 0.434 |
| SPP1 | VTN | 0 | 0 | 0 | 0 | 0.429 | 0.429 |
| SYNGAP1 | GNAO1 | 0 | 0.351 | 0 | 0 | 0.129 | 0.411 |
| ZP3 | CACNA1C | 0 | 0.098 | 0 | 0 | 0.372 | 0.41 |

**Note:** PQDS: Pi-qi-deficiency syndrome.

**^a^** Cooccurrence, Gene families whose occurrence patterns across genomes show similarities;

**^b^** Coexpression, Proteins whose genes are observed to be correlated in expression, across a large number of experiments;

**^c^** Experiments, Co-purification, co-crystallization, Yeast2Hybrid, Genetic Interactions, etc ... as imported from primary sources;

**^d^** Database, Known metabolic pathways, protein complexes, signal transduction pathways, etc ... from curated databases;

**^e^** Textmining, Automated, unsupervised textmining - searching for proteins that are frequently mentioned together;

^f^ The value determine the thickness of edge between nudes in a created interaction network cartoon, indicating the interaction strength of all the support data.

**Table S11 The PQDS-specific differential genes regulated by the PQDS-specific differential lncRNAs order by genes**

| **Targets ^a^** | **Count** | **Trans-acting lncRNAs** |
| --- | --- | --- |
| GNAO1 | 52 | EMX2OS:2,LINC01032:4,lnc-AC106873.4.1-6:2,lnc-AC115989.1.1-5:1,lnc-AL136218.1-1:2,lnc-ALK-1:1,lnc-AUH-3:1,lnc-BAG1-2:9,lnc-C22orf46-2:1,lnc-C5orf49-2:1,lnc-C6orf120-3:1,lnc-C6orf201-2:2,lnc-C7orf55-1:2,lnc-CPSF2-3:1,lnc-CRK-3:4,lnc-EIF5B-4:1,lnc-GLT6D1-2:1,lnc-HPS6-1:1,lnc-JPH2-1:1,lnc-LETM2-4:1,lnc-LHFPL3-4:1,lnc-MDM1-1:1,lnc-MDM1-1:15,lnc-MDM1-1:2,lnc-MICAL2-4:1,lnc-MLXIP-1:1,lnc-MPHOSPH8-7:1,lnc-MRPL33-1:2,lnc-MZT2B-1:2,lnc-NNT-5:1,lnc-NR4A2-3:1,lnc-OSBPL2-1:1,lnc-PARP8-2:2,lnc-S100P-1:1,lnc-SFTA2-14:1,lnc-SIRT3-1:1,lnc-SLC12A7-4:3,lnc-SP110-8:2,lnc-TAL1-3:2,lnc-TBC1D12-5:1,lnc-TBC1D20-1:1,lnc-TSTA3-4:1,lnc-TYMS-3:1,lnc-U2AF1L4-1:9,lnc-WDR34-2:3,lnc-ZG16-2:1,lnc-ZNF771-1:1,MIR663AHG:14,MIR663AHG:9,MMP25-AS1:25,SIRPG-AS1:1,TCONS_00048679 |
| C4BPB | 33 | DNAJC27-AS1:8,HCG21:1,lnc-AL136218.1-1:2,lnc-ARVCF-1:9,lnc-AXDND1-1:1,lnc-C7orf55-1:2,lnc-C9orf107-1:1,lnc-CREBBP-1:1,lnc-FAM82A2-1:1,lnc-HMGB2-8:1,lnc-HYOU1-1:4,lnc-JPH2-1:1,lnc-MICAL2-4:1,lnc-MLXIP-1:1,lnc-MRPL35-1:2,lnc-MYOCD-3:1,lnc-OSBPL2-1:1,lnc-PEBP1-2:2,lnc-PLAGL2-6:1,lnc-PRPF4B-4:5,lnc-RAB28-6:1,lnc-RBM28-3:2,lnc-RNF182-2:1,lnc-RPF1-1:11,lnc-RPL6-1:1,lnc-SFTA2-14:1,lnc-SP110-8:2,lnc-TBC1D20-1:1,lnc-TSTA3-4:1,lnc-VSIG7-4:1,lnc-WDR34-2:3,MEG3:41,TCONS_00048679 |
| COL2A1 | 25 | CCDC18-AS1:37,INHBA-AS1:5,lnc-AC092329.1-1:3,lnc-ACTR2-8:1,lnc-AKT2-2:1,lnc-C1orf132-1:7,lnc-C6orf120-3:1,lnc-C6orf201-2:2,lnc-CARD11-1:1,lnc-CDK10-2:5,lnc-CREBBP-1:1,lnc-FBXO44-1:1,lnc-HTR2C-5:1,lnc-IL22RA2-1:1,lnc-IQCG-8:1,lnc-ITGA2-1:6,lnc-NNT-2:1,lnc-NPAS1-3:1,lnc-PANK1-5:1,lnc-PLAGL2-6:1,lnc-RABEP2-2:1,lnc-RPL6-1:1,lnc-TUBB2B-8:4,MMP25-AS1:25,PCAT7:5 |
| LRFN3 | 20 | lnc-AXDND1-1:1,lnc-BACE2-1:1,lnc-IFNK-7:1,lnc-JPH2-1:1,lnc-LEPROTL1-4:7,lnc-LGSN-3:1,lnc-LPCAT1-3:10,lnc-OCM-3:13,lnc-OSBPL2-1:1,lnc-OST4-2:1,lnc-PRPF4B-4:5,lnc-S100P-1:1,lnc-TSTA3-4:1,lnc-ZG16-2:1,MIR663AHG:9,ST8SIA6-AS1:3,STAU2-AS1:2,TCONS_00048664,TCONS_00048679,TCONS_00049108 |
| LILRB3 | 16 | LINC00957:3,lnc-AC012652.1.1-4:1,lnc-C5orf49-2:1,lnc-CNST-1:4,lnc-CRK-3:4,lnc-HUNK-2:1,lnc-MICAL2-4:1,lnc-MYL7-3:1,lnc-NDUFA13-1:1,lnc-NNT-2:1,lnc-PEX14-2:1,lnc-PLAC9-2:1,lnc-RNF135-1:12,lnc-SIRT3-1:1,lnc-SP110-8:2,MEG3:41 |
| BRSK1 | 12 | LINC00958:13,LINC00963:35,lnc-AC009958.1-3:1,lnc-AL901608.1-3:20,lnc-ANKRD65-4:2,lnc-EFNA4-1:6,lnc-KIAA1609-3:2,lnc-KIAA1737-1:4,lnc-OR4C12-9:4,lnc-SH3D19-11:1,lnc-THAP11-1:1,lnc-USP6-2:6 |
| DPYSL3 | 12 | lnc-AC012652.1.1-4:1,lnc-CRK-3:4,lnc-DNAH9-1:1,lnc-FAM82A2-1:1,lnc-LEPROTL1-4:7,lnc-NR4A2-3:1,lnc-OCM-3:13,lnc-PARP8-2:2,lnc-PLA2G10-4:1,lnc-S100P-1:1,lnc-TCF25-1:1,STAU2-AS1:2 |
| NAPRT | 10 | HCG21:1,lnc-AGBL1-7:1,lnc-AUH-3:1,lnc-C16orf5-1:1,lnc-EXTL3-6:4,lnc-FAIM-3:1,lnc-SFTA2-14:1,lnc-SIRT3-1:1,lnc-TRAF3-1:1,SIRPG-AS1:1 |
| COL5A3 | 9 | lnc-AL901608.1-3:20,lnc-BCL11A-6:1,lnc-EFNA4-1:6,lnc-FURIN-1:1,lnc-KIAA1609-3:2,lnc-KIAA1737-1:4,lnc-PLEKHG4-1:1,lnc-SH3D19-11:1,lnc-USP6-2:6 |
| SYNGAP1 | 9 | LINC00299:5,LINC00926:14,lnc-CNST-1:4,lnc-LILRB4-1:2,lnc-NPC1-7:3,lnc-PSMD8-1:1,lnc-RPRML-3:8,lnc-TCF25-1:1,OSER1-AS1:15 |
| PCDHGC5 | 8 | lnc-C16orf5-1:1,lnc-CDK10-2:5,lnc-KRTAP10-12-3:1,lnc-MRPL33-1:2,lnc-MZT2B-1:2,lnc-RP1-239B22.1.1-2:2,lnc-RPL6-1:1,lnc-TTC23-5:1 |
| ADAMTS2 | 7 | LINC00547:1,LINC00958:13,lnc-KIAA1609-3:2,lnc-MED20-5:3,lnc-MTERFD2-5:1,lnc-OR4C12-9:4,lnc-USP6-2:6 |
| HOXA3 | 7 | lnc-AL669831.1-3:33,lnc-ATAD5-3:12,lnc-CSDC2-1:1,lnc-DEPDC4-2:3,lnc-FAM200B-1:16,lnc-ITGA2-1:2,MIR9-3HG:31 |
| PLEKHN1 | 7 | LINC00926:14,lnc-ANGEL1-1:7,lnc-CNST-1:4,lnc-NPC1-7:3,lnc-PSMD8-1:1,MIR663AHG:28,OSER1-AS1:15 |
| TRIM73 | 7 | LINC00299:5,lnc-AC010336.2-4:2,lnc-PSMD8-1:1,lnc-TRAF3-1:1,lnc-TTC24-2:1,MEG3:41,THUMPD3-AS1:23 |
| ADAM12 | 6 | LINC00963:35,lnc-ANKRD65-4:2,lnc-ITGA2-1:2,lnc-KIAA1737-1:4,lnc-MIDN-1:1,lnc-OR4C12-9:4 |
| CLDN7 | 6 | LINC00200:1,lnc-AL901608.1-3:39,lnc-FLRT2-2:6,lnc-MIDN-1:1,lnc-PTMS-1:1,RBFADN:7 |
| DHRS2 | 5 | lnc-ACTR2-8:1,lnc-CDK10-2:5,lnc-CHMP4C-10:1,lnc-NPAS1-3:1,PCAT7:5 |
| NELFE | 5 | LINC00958:13,LINC00963:35,lnc-ANKRD65-4:2,lnc-OR4C12-9:4,lnc-USP6-2:6 |
| PTPRB | 5 | lnc-ATAD5-3:12,lnc-DDR2-5:1,lnc-ITGA2-1:2,lnc-MIDN-1:1,RBFADN:7 |
| B3GALNT2 | 4 | LINC00958:13,lnc-ANKRD65-4:2,lnc-KIAA1737-1:4,lnc-USP6-2:6 |
| CPLX2 | 4 | HCG21:1,LINC00311:2,lnc-AUH-3:1,lnc-RBM28-3:2 |
| TMEM132B | 4 | lnc-KCTD17-5:1,lnc-TFEC-9:3,TMEM161B-AS1:31,TP53TG1:7 |
| C4BPA | 3 | lnc-C7orf55-1:2,lnc-PRPF4B-4:5,TCONS_00048679 |
| CPXM1 | 3 | LINC00200:1,lnc-MIDN-1:1,RBFADN:7 |
| CRB3 | 3 | IQCH-AS1:1,lnc-AC007952.1-1:1,lnc-SCN8A-2:9 |
| GPM6B | 3 | LINC00958:13,lnc-DEPDC4-2:3,lnc-KIAA1737-1:4 |
| MEIS3 | 3 | IQCH-AS1:1,lnc-CDHR4-3:1,lnc-NADSYN1-1:2 |
| SLC47A1 | 3 | lnc-GGCT-1:5,lnc-GGNBP1-5:1,MIR9-3HG:31 |
| ZNF415 | 3 | LINC00958:13,lnc-EFNA4-1:6,lnc-OR4C12-9:4 |
| C4A | 2 | ILF3-AS1:5,lnc-CDH12-1:2 |
| C4B | 2 | LINC00857:1,lnc-FAM106B-1:3 |
| DHX16 | 2 | lnc-RNF19B-1:7,lnc-ST3GAL4-2:1 |
| FAM83D | 2 | IQCH-AS1:6,lnc-EFNA4-1:6 |
| GGACT | 2 | lnc-FAM200B-1:16,lnc-NAGS-1:3 |
| LOC100130520 | 2 | lnc-PTMS-1:1,MIR9-3HG:31 |
| LOC102724994 | 2 | LINC00963:36,lnc-AL669831.1-3:33 |
| NR1I2 | 2 | lnc-RBPMS2-3:1,TP53TG1:7 |
| PCDHGA11 | 2 | LINC00092:1,lnc-SIGIRR-1:2 |
| PCDHGA8 | 2 | lnc-SBK2-1:1,lnc-SIGIRR-1:2 |
| RAI14 | 2 | lnc-ATL3-1:9,lnc-MIDN-1:1 |
| RXRB | 2 | lnc-CDH12-1:2,lnc-RBM12B-1:1 |
| SAMD14 | 2 | SNHG11:14,THUMPD3-AS1:23 |
| SEMA5A | 2 | lnc-CDK10-2:5,lnc-RP1-239B22.1.1-2:2 |
| SORCS3 | 2 | lnc-CHMP4C-10:1,lnc-RP1-239B22.1.1-2:2 |
| VTN | 2 | LINC00963:36,MIR9-3HG:31 |
| ZP3 | 2 | lnc-MIDN-1:1,lnc-PTMS-1:1 |
| CACNA1C | 1 | lnc-RP11-706O15.1.1-1:4 |
| COL10A1 | 1 | lnc-CDHR4-3:1 |
| FAM171B | 1 | IQCH-AS1:6 |
| GAL3ST1 | 1 | lnc-LHFPL3-4:1 |
| GLB1L2 | 1 | lnc-CDHR4-3:1 |
| KCNJ14 | 1 | lnc-CMC1-1:3 |
| KIR2DL1 | 1 | lnc-MIDN-1:1 |
| PLCXD1 | 1 | lnc-POLE3-3:2 |
| PRG4 | 1 | MIR4435-2HG:11 |
| SLC35G2 | 1 | LINC00857:1 |
| SOGA3 | 1 | lnc-CDH12-1:2 |
| SPP1 | 1 | lnc-SIRT3-1:1 |

^a^ Under the prerequisite of coexpression (Pearson’s correlation coefficient >0.8) of a lncRNA and a gene in PQDS population, the *trans*-acting lncRNAs could directly bind to genes’ mRNAs.

**Table S12 Detailed the *cis*-regulation pairs between the PQDC-specific differential genes and lncRNAs in PQDS population**

| **gene** | **ncrna ^a^** | **Correlation Coefficient ^b^** | ***P*-value** | **gene_chrom** | **gene_start** | **gene_end** | **gene strand** | **distance** | **ncrna_chrom** | **ncrna_start** | **ncrna_end** | **ncrna strand** |
| --- | --- | --- | --- | --- | --- | --- | --- | --- | --- | --- | --- | --- |
| LOC100130520 | lnc-C17orf77-1:1 | 0.847092173 | 0.00099874 | NC_000017.11 | 74557246 | 74567534 | . | 32305 | NC_000017.11 | 74599839 | 74604269 | + |
| LOC100130520 | lnc-CD300C-2:1 | 0.956957772 | 3.92E-06 | NC_000017.11 | 74557246 | 74567534 | . | 1248 | NC_000017.11 | 74553728 | 74558494 | - |
| CLEC4C | lnc-CLEC4C-1:1 | 0.930646387 | 3.23E-05 | NC_000012.12 | 7729415 | 7749473 | . | 2734 | NC_000012.12 | 7725531 | 7726681 | - |

^a^ The common differential lncRNAs appeared in both case populations (QDC and PQDS of CSG) were indicated in red font.

^b^ *Cis*-acting lncRNAs usually control their neighboring genes, so, for each coexpression pair (|Pearson’s correlation coefficient| >0.8) of gene and lncRNA, if the gene is closed (less than 100 kb) to the lncRNA in a genome, it was considered as a candidate target of the cis-acting lncRNA.

**Table S13 Detailed list for binding pairs of the differential lncRNAs and the 12 common differential genes**

| **ncrna** | **ncrna_start** | **ncrna_end** | **gene_name** | **gene_start** | **gene_end** | **free_energy** | **Interaction ^a^** | **Common**  **lncRNA ^b^** |
| --- | --- | --- | --- | --- | --- | --- | --- | --- |
| DLX6-AS1:13 | 85 | 151 | ADAMTSL5 | 2678 | 2744 | -99.41 | lmBtoBDcommon | Yes |
| lnc-AKAP11-1:2 | 1836 | 1889 | ADAMTSL5 | 2639 | 2692 | -94.16 | lmBtoBDcommon | Yes |
| lnc-C12orf50-6:2 | 968 | 1019 | ADAMTSL5 | 2834 | 2887 | -80.13 | lmBtoBDcommon | Yes |
| lnc-CA10-1:1 | 84 | 133 | ADAMTSL5 | 2639 | 2688 | -80.39 | lmBtoBDcommon | Yes |
| lnc-CEP170-11:1 | 173 | 237 | ADAMTSL5 | 2682 | 2746 | -80.92 | lmBtoBDcommon | Yes |
| lnc-CGNL1-6:1 | 40 | 84 | ADAMTSL5 | 1345 | 1389 | -52.87 | lmBtoBDcommon | Yes |
| lnc-EID2B-1:1 | 376 | 426 | ADAMTSL5 | 2639 | 2689 | -88.71 | lmBtoBDcommon | Yes |
| lnc-GGCT-1:12 | 551 | 616 | ADAMTSL5 | 2678 | 2743 | -106.13 | lmBtoBDcommon | Yes |
| lnc-HIST1H2AI-1:3 | 658 | 718 | ADAMTSL5 | 2838 | 2898 | -84.01 | lmBtoBDcommon | Yes |
| lnc-KIAA0226-4:1 | 158 | 211 | ADAMTSL5 | 2697 | 2750 | -82.94 | lmBtoBDcommon | Yes |
| lnc-LRRFIP2-1:1 | 1725 | 1779 | ADAMTSL5 | 2677 | 2731 | -98.63 | lmBtoBDcommon | Yes |
| lnc-PGPEP1L-3:1 | 346 | 399 | ADAMTSL5 | 2845 | 2895 | -75.61 | lmBtoBDcommon | Yes |
| lnc-PLEKHA7-2:1 | 2469 | 2541 | ADAMTSL5 | 2678 | 2750 | -111.48 | lmBtoBDcommon | Yes |
| lnc-RPRML-3:20 | 1504 | 1560 | ADAMTSL5 | 2834 | 2890 | -83.98 | lmBtoBDcommon | Yes |
| lnc-THAP10-1:1 | 472 | 539 | ADAMTSL5 | 2683 | 2750 | -99.46 | lmBtoBDcommon | Yes |
| lnc-VASH2-1:1 | 314 | 367 | ADAMTSL5 | 2658 | 2711 | -66.8 | lmBtoBDcommon | Yes |
| lnc-ZNF114-2:1 | 251 | 318 | ADAMTSL5 | 2683 | 2750 | -93.86 | lmBtoBDcommon | Yes |
| TCONS_00048671 | 41 | 90 | ADAMTSL5 | 15 | 61 | -67.79 | lmBtoBDcommon | Yes |
| TCONS_00048674 | 706 | 758 | ADAMTSL5 | 971 | 1019 | -74.28 | lmBtoBDcommon | Yes |
| TCONS_00052318 | 48 | 86 | ADAMTSL5 | 23 | 68 | -54.11 | lmBtoBDcommon | Yes |
| TCONS_00052867 | 586 | 634 | ADAMTSL5 | 53 | 99 | -71.45 | lmBtoBDcommon | Yes |
| TCONS_00052868 | 575 | 623 | ADAMTSL5 | 53 | 99 | -71.45 | lmBtoBDcommon | Yes |
| lnc-AMZ2-5:1 | 1708 | 1776 | ADAMTSL5 | 2828 | 2896 | -94.21 | lmDtoBDcommon | Yes |
| lnc-ARL1-3:1 | 2236 | 2284 | ADAMTSL5 | 2702 | 2751 | -90.99 | lmDtoBDcommon | Yes |
| lnc-C12orf50-6:2 | 968 | 1019 | ADAMTSL5 | 2834 | 2887 | -80.13 | lmDtoBDcommon | Yes |
| lnc-CACYBP-2:1 | 1366 | 1419 | ADAMTSL5 | 2658 | 2711 | -77.9 | lmDtoBDcommon | Yes |
| lnc-CEP170-11:1 | 173 | 237 | ADAMTSL5 | 2682 | 2746 | -80.92 | lmDtoBDcommon | Yes |
| lnc-CSNK1D-1:1 | 1115 | 1165 | ADAMTSL5 | 2639 | 2689 | -71.54 | lmDtoBDcommon | Yes |
| lnc-FAM32A-2:1 | 364 | 408 | ADAMTSL5 | 1587 | 1633 | -58.87 | lmDtoBDcommon | Yes |
| lnc-LRRFIP2-1:1 | 1725 | 1779 | ADAMTSL5 | 2677 | 2731 | -98.63 | lmDtoBDcommon | Yes |
| lnc-NPBWR1-1:2 | 739 | 792 | ADAMTSL5 | 2697 | 2750 | -83.14 | lmDtoBDcommon | Yes |
| lnc-PGPEP1L-3:1 | 346 | 399 | ADAMTSL5 | 2845 | 2895 | -75.61 | lmDtoBDcommon | Yes |
| lnc-PLEKHA7-2:1 | 2469 | 2541 | ADAMTSL5 | 2678 | 2750 | -111.48 | lmDtoBDcommon | Yes |
| lnc-PLEKHG2-1:1 | 1325 | 1378 | ADAMTSL5 | 2838 | 2893 | -86.31 | lmDtoBDcommon | Yes |
| lnc-PRPF4B-4:4 | 711 | 768 | ADAMTSL5 | 2834 | 2893 | -87.26 | lmDtoBDcommon | Yes |
| lnc-SWI5-1:1 | 28 | 73 | ADAMTSL5 | 2701 | 2746 | -70.18 | lmDtoBDcommon | Yes |
| lnc-TWIST1-1:3 | 789 | 842 | ADAMTSL5 | 2658 | 2711 | -85.27 | lmDtoBDcommon | Yes |
| lnc-VASH2-1:1 | 314 | 367 | ADAMTSL5 | 2658 | 2711 | -66.8 | lmDtoBDcommon | Yes |
| lnc-ZNF212-2:2 | 529 | 597 | ADAMTSL5 | 2682 | 2750 | -104.26 | lmDtoBDcommon | Yes |
| DANT1:2 | 10896 | 10940 | ADAMTSL5 | 1694 | 1742 | -50.52 | lmBtoBDcommon | No |
| FTX:1 | 272 | 332 | ADAMTSL5 | 2838 | 2898 | -83.68 | lmBtoBDcommon | No |
| GAS6-AS2:1 | 197 | 250 | ADAMTSL5 | 2697 | 2750 | -80.72 | lmBtoBDcommon | No |
| LBX2-AS1:2 | 479 | 526 | ADAMTSL5 | 1786 | 1833 | -62.94 | lmBtoBDcommon | No |
| LINC00654:4 | 6767 | 6816 | ADAMTSL5 | 2696 | 2746 | -64.43 | lmBtoBDcommon | No |
| LINC00847:14 | 231 | 297 | ADAMTSL5 | 2680 | 2751 | -130.53 | lmBtoBDcommon | No |
| LINC00887:5 | 1237 | 1283 | ADAMTSL5 | 2838 | 2886 | -75.51 | lmBtoBDcommon | No |
| LINC01299:2 | 255 | 316 | ADAMTSL5 | 2689 | 2750 | -94.05 | lmBtoBDcommon | No |
| LINC01426:3 | 2288 | 2339 | ADAMTSL5 | 2834 | 2887 | -70.72 | lmBtoBDcommon | No |
| lnc-ABCC6-2:1 | 863 | 925 | ADAMTSL5 | 2639 | 2701 | -95 | lmBtoBDcommon | No |
| lnc-ABHD2-4:1 | 985 | 1038 | ADAMTSL5 | 2697 | 2750 | -81.04 | lmBtoBDcommon | No |
| lnc-AC069257.9.1-5:5 | 45 | 89 | ADAMTSL5 | 2685 | 2729 | -68.38 | lmBtoBDcommon | No |
| lnc-AC091132.2-7:1 | 15 | 63 | ADAMTSL5 | 141 | 185 | -58.93 | lmBtoBDcommon | No |
| lnc-AC107021.1-7:1 | 19 | 67 | ADAMTSL5 | 496 | 544 | -55.69 | lmBtoBDcommon | No |
| lnc-ACR-2:5 | 13 | 59 | ADAMTSL5 | 681 | 731 | -54.52 | lmBtoBDcommon | No |
| lnc-ADA-2:1 | 612 | 676 | ADAMTSL5 | 2836 | 2900 | -93 | lmBtoBDcommon | No |
| lnc-AMZ2-3:1 | 158 | 221 | ADAMTSL5 | 2687 | 2750 | -99 | lmBtoBDcommon | No |
| lnc-ANKRD30B-7:2 | 285 | 332 | ADAMTSL5 | 1894 | 1942 | -52.23 | lmBtoBDcommon | No |
| lnc-APBA2-5:8 | 51 | 96 | ADAMTSL5 | 36 | 77 | -58.42 | lmBtoBDcommon | No |
| lnc-BCL9L-1:1 | 1927 | 1992 | ADAMTSL5 | 2639 | 2704 | -123.01 | lmBtoBDcommon | No |
| lnc-C11orf94-1:1 | 187 | 228 | ADAMTSL5 | 1785 | 1834 | -62.77 | lmBtoBDcommon | No |
| lnc-C14orf79-3:1 | 2532 | 2584 | ADAMTSL5 | 2698 | 2750 | -73.01 | lmBtoBDcommon | No |
| lnc-C3orf62-2:1 | 80 | 133 | ADAMTSL5 | 2678 | 2731 | -85.01 | lmBtoBDcommon | No |
| lnc-CEP170-9:2 | 1215 | 1268 | ADAMTSL5 | 2639 | 2692 | -99.41 | lmBtoBDcommon | No |
| lnc-CLCN1-2:1 | 34 | 84 | ADAMTSL5 | 58 | 98 | -57.94 | lmBtoBDcommon | No |
| lnc-CLDN23-3:1 | 59 | 115 | ADAMTSL5 | 2844 | 2900 | -75.77 | lmBtoBDcommon | No |
| lnc-CLIC3-2:1 | 311 | 352 | ADAMTSL5 | 159 | 200 | -58.8 | lmBtoBDcommon | No |
| lnc-COX19-2:2 | 766 | 809 | ADAMTSL5 | 710 | 752 | -54.63 | lmBtoBDcommon | No |
| lnc-CRYBA4-1:55 | 1455 | 1502 | ADAMTSL5 | 107 | 155 | -64.81 | lmBtoBDcommon | No |
| lnc-EXOC2-8:4 | 1864 | 1932 | ADAMTSL5 | 2682 | 2750 | -100.71 | lmBtoBDcommon | No |
| lnc-EXT1-1:1 | 402 | 474 | ADAMTSL5 | 2678 | 2750 | -128.5 | lmBtoBDcommon | No |
| lnc-FAM160A1-8:1 | 120 | 163 | ADAMTSL5 | 140 | 182 | -53.71 | lmBtoBDcommon | No |
| lnc-FTSJ1-2:1 | 486 | 538 | ADAMTSL5 | 2658 | 2710 | -82.96 | lmBtoBDcommon | No |
| lnc-GTPBP1-1:1 | 253 | 295 | ADAMTSL5 | 1581 | 1626 | -52.55 | lmBtoBDcommon | No |
| lnc-HEATR2-3:1 | 3388 | 3444 | ADAMTSL5 | 2661 | 2717 | -79.53 | lmBtoBDcommon | No |
| lnc-HSPG2-2:1 | 80 | 143 | ADAMTSL5 | 2630 | 2692 | -93.25 | lmBtoBDcommon | No |
| lnc-HTRA2-1:1 | 8 | 52 | ADAMTSL5 | 39 | 84 | -61.09 | lmBtoBDcommon | No |
| lnc-KLF13-2:4 | 2066 | 2120 | ADAMTSL5 | 2831 | 2885 | -62.53 | lmBtoBDcommon | No |
| lnc-KLHL5-2:1 | 3314 | 3365 | ADAMTSL5 | 2677 | 2728 | -102.76 | lmBtoBDcommon | No |
| lnc-LIPI-9:1 | 1549 | 1602 | ADAMTSL5 | 2697 | 2750 | -92.84 | lmBtoBDcommon | No |
| lnc-LRRC56-3:4 | 761 | 802 | ADAMTSL5 | 1762 | 1806 | -52.53 | lmBtoBDcommon | No |
| lnc-MAPK6-4:2 | 144 | 208 | ADAMTSL5 | 2834 | 2895 | -98.93 | lmBtoBDcommon | No |
| lnc-MOGAT2-3:1 | 70 | 112 | ADAMTSL5 | 1586 | 1631 | -63.27 | lmBtoBDcommon | No |
| lnc-MTR-4:1 | 523 | 590 | ADAMTSL5 | 2683 | 2750 | -99.36 | lmBtoBDcommon | No |
| lnc-NKAIN3-1:2 | 414 | 467 | ADAMTSL5 | 2658 | 2711 | -82.52 | lmBtoBDcommon | No |
| lnc-NRARP-3:1 | 1107 | 1152 | ADAMTSL5 | 1555 | 1602 | -58.93 | lmBtoBDcommon | No |
| lnc-NT5C-3:3 | 165 | 199 | ADAMTSL5 | 1897 | 1934 | -55.9 | lmBtoBDcommon | No |
| lnc-OLFML3-5:1 | 458 | 527 | ADAMTSL5 | 2682 | 2750 | -103.78 | lmBtoBDcommon | No |
| lnc-PAIP2-1:1 | 1 | 42 | ADAMTSL5 | 1614 | 1665 | -55.56 | lmBtoBDcommon | No |
| lnc-PCBP3-3:9 | 656 | 707 | ADAMTSL5 | 719 | 767 | -58.25 | lmBtoBDcommon | No |
| lnc-PRAGMIN.1-3:2 | 35 | 83 | ADAMTSL5 | 134 | 180 | -68.94 | lmBtoBDcommon | No |
| lnc-PRDM11-9:2 | 484 | 528 | ADAMTSL5 | 122 | 170 | -56.17 | lmBtoBDcommon | No |
| lnc-PTGER1-1:2 | 118 | 166 | ADAMTSL5 | 75 | 123 | -58.42 | lmBtoBDcommon | No |
| lnc-PTPN12-1:1 | 1511 | 1564 | ADAMTSL5 | 2697 | 2750 | -89.14 | lmBtoBDcommon | No |
| lnc-PUF60-1:1 | 313 | 358 | ADAMTSL5 | 980 | 1025 | -62.17 | lmBtoBDcommon | No |
| lnc-RAD52-2:5 | 1089 | 1149 | ADAMTSL5 | 2837 | 2889 | -76.49 | lmBtoBDcommon | No |
| lnc-RAP1GAP2-3:1 | 652 | 705 | ADAMTSL5 | 2639 | 2693 | -83.2 | lmBtoBDcommon | No |
| lnc-RBAK-2:10 | 462 | 510 | ADAMTSL5 | 67 | 110 | -58.24 | lmBtoBDcommon | No |
| lnc-RBPMS2-1:2 | 143 | 190 | ADAMTSL5 | 565 | 614 | -50.66 | lmBtoBDcommon | No |
| lnc-RCN1-2:1 | 488 | 544 | ADAMTSL5 | 2688 | 2750 | -98.78 | lmBtoBDcommon | No |
| lnc-ROPN1L-3:1 | 1894 | 1942 | ADAMTSL5 | 1595 | 1642 | -53.32 | lmBtoBDcommon | No |
| lnc-RPL23A-2:1 | 1012 | 1057 | ADAMTSL5 | 1024 | 1071 | -54.12 | lmBtoBDcommon | No |
| lnc-RUNDC3A-4:2 | 390 | 440 | ADAMTSL5 | 45 | 96 | -55.08 | lmBtoBDcommon | No |
| lnc-SBDS-4:1 | 3776 | 3848 | ADAMTSL5 | 2820 | 2893 | -116.67 | lmBtoBDcommon | No |
| lnc-SEC11A-2:1 | 136 | 170 | ADAMTSL5 | 1789 | 1824 | -60.15 | lmBtoBDcommon | No |
| lnc-SIX5-1:1 | 1033 | 1075 | ADAMTSL5 | 530 | 576 | -66.94 | lmBtoBDcommon | No |
| lnc-TAOK3-1:11 | 1369 | 1430 | ADAMTSL5 | 2829 | 2890 | -91.42 | lmBtoBDcommon | No |
| lnc-TOR3A-1:1 | 439 | 492 | ADAMTSL5 | 2697 | 2750 | -90.76 | lmBtoBDcommon | No |
| lnc-TTR-3:1 | 2546 | 2595 | ADAMTSL5 | 2639 | 2689 | -81.58 | lmBtoBDcommon | No |
| lnc-TYSND1-1:7 | 1205 | 1258 | ADAMTSL5 | 2697 | 2750 | -82.95 | lmBtoBDcommon | No |
| lnc-UGCG-1:3 | 792 | 843 | ADAMTSL5 | 2677 | 2728 | -87.36 | lmBtoBDcommon | No |
| lnc-WDR73-7:2 | 14 | 58 | ADAMTSL5 | 173 | 213 | -55.58 | lmBtoBDcommon | No |
| lnc-ZKSCAN3-1:1 | 167 | 216 | ADAMTSL5 | 2836 | 2878 | -61.26 | lmBtoBDcommon | No |
| lnc-ZNF827-11:1 | 107 | 154 | ADAMTSL5 | 563 | 612 | -53.15 | lmBtoBDcommon | No |
| PART1:10 | 3587 | 3639 | ADAMTSL5 | 2639 | 2692 | -88.86 | lmBtoBDcommon | No |
| PSMB8-AS1:1 | 1156 | 1206 | ADAMTSL5 | 2678 | 2728 | -65.5 | lmBtoBDcommon | No |
| PSMB8-AS1:5 | 1217 | 1267 | ADAMTSL5 | 2678 | 2728 | -65.5 | lmBtoBDcommon | No |
| SMIM2-AS1:11 | 1622 | 1675 | ADAMTSL5 | 2658 | 2711 | -80.87 | lmBtoBDcommon | No |
| SNHG4:3 | 277 | 349 | ADAMTSL5 | 2678 | 2750 | -114.08 | lmBtoBDcommon | No |
| ST3GAL5-AS1:4 | 978 | 1021 | ADAMTSL5 | 2658 | 2703 | -64.97 | lmBtoBDcommon | No |
| TCONS_00049141 | 1959 | 2003 | ADAMTSL5 | 1897 | 1943 | -56.99 | lmBtoBDcommon | No |
| TCONS_00052658 | 3 | 53 | ADAMTSL5 | 83 | 130 | -55.08 | lmBtoBDcommon | No |
| TCONS_00052860 | 701 | 749 | ADAMTSL5 | 53 | 99 | -71.45 | lmBtoBDcommon | No |
| ZNF528-AS1:3 | 15 | 61 | ADAMTSL5 | 145 | 197 | -58.07 | lmBtoBDcommon | No |
| DNAJC27-AS1:8 | 3428 | 3500 | ADAMTSL5 | 2678 | 2750 | -122.48 | lmDtoBDcommon | No |
| EMX2OS:2 | 5799 | 5860 | ADAMTSL5 | 2683 | 2744 | -88.71 | lmDtoBDcommon | No |
| LINC01032:4 | 370 | 428 | ADAMTSL5 | 2692 | 2750 | -85.45 | lmDtoBDcommon | No |
| LINC01285:5 | 2221 | 2270 | ADAMTSL5 | 2639 | 2688 | -85.56 | lmDtoBDcommon | No |
| lnc-AC007390.5.1-1:1 | 23 | 74 | ADAMTSL5 | 2685 | 2736 | -62.54 | lmDtoBDcommon | No |
| lnc-AC012652.1.1-4:1 | 733 | 798 | ADAMTSL5 | 2616 | 2681 | -97.64 | lmDtoBDcommon | No |
| lnc-AC106873.4.1-6:2 | 1808 | 1876 | ADAMTSL5 | 2683 | 2751 | -107.28 | lmDtoBDcommon | No |
| lnc-AC115989.1.1-5:1 | 472 | 525 | ADAMTSL5 | 2697 | 2750 | -82.22 | lmDtoBDcommon | No |
| lnc-ALG10B-8:1 | 2022 | 2091 | ADAMTSL5 | 2639 | 2708 | -116.1 | lmDtoBDcommon | No |
| lnc-ALK-1:1 | 672 | 744 | ADAMTSL5 | 2678 | 2750 | -123.08 | lmDtoBDcommon | No |
| lnc-ANKRD30BL-3:1 | 226 | 267 | ADAMTSL5 | 1554 | 1599 | -56.7 | lmDtoBDcommon | No |
| lnc-ARVCF-1:9 | 141 | 194 | ADAMTSL5 | 2697 | 2750 | -85.83 | lmDtoBDcommon | No |
| lnc-AXDND1-1:1 | 61 | 114 | ADAMTSL5 | 2658 | 2711 | -75.22 | lmDtoBDcommon | No |
| lnc-BACE2-1:1 | 432 | 485 | ADAMTSL5 | 2639 | 2692 | -93.39 | lmDtoBDcommon | No |
| lnc-C16orf5-1:1 | 903 | 975 | ADAMTSL5 | 2678 | 2750 | -104.66 | lmDtoBDcommon | No |
| lnc-C1orf132-1:7 | 302 | 348 | ADAMTSL5 | 1874 | 1918 | -51.43 | lmDtoBDcommon | No |
| lnc-C22orf46-2:1 | 251 | 307 | ADAMTSL5 | 2697 | 2750 | -87.43 | lmDtoBDcommon | No |
| lnc-C6orf120-3:1 | 245 | 302 | ADAMTSL5 | 2645 | 2702 | -100.24 | lmDtoBDcommon | No |
| lnc-C6orf201-2:2 | 2240 | 2309 | ADAMTSL5 | 2681 | 2750 | -99.58 | lmDtoBDcommon | No |
| lnc-C7orf55-1:2 | 626 | 667 | ADAMTSL5 | 2846 | 2887 | -59.99 | lmDtoBDcommon | No |
| lnc-C9orf146-7:1 | 881 | 934 | ADAMTSL5 | 2658 | 2711 | -92.52 | lmDtoBDcommon | No |
| lnc-CARD11-1:1 | 242 | 282 | ADAMTSL5 | 1590 | 1640 | -52.99 | lmDtoBDcommon | No |
| lnc-CIDEA-2:3 | 1241 | 1294 | ADAMTSL5 | 2837 | 2890 | -76.2 | lmDtoBDcommon | No |
| lnc-CPSF2-3:1 | 1484 | 1540 | ADAMTSL5 | 2834 | 2890 | -85.57 | lmDtoBDcommon | No |
| lnc-CREBBP-1:1 | 813 | 871 | ADAMTSL5 | 2692 | 2750 | -89.59 | lmDtoBDcommon | No |
| lnc-CRK-3:4 | 2 | 54 | ADAMTSL5 | 46 | 91 | -59.46 | lmDtoBDcommon | No |
| lnc-DNAL4-3:4 | 233 | 282 | ADAMTSL5 | 1492 | 1544 | -57.28 | lmDtoBDcommon | No |
| lnc-EIF5B-4:1 | 1891 | 1941 | ADAMTSL5 | 2639 | 2689 | -89.46 | lmDtoBDcommon | No |
| lnc-FAIM-3:1 | 388 | 450 | ADAMTSL5 | 2838 | 2900 | -81.52 | lmDtoBDcommon | No |
| lnc-FAM82A2-1:1 | 1520 | 1568 | ADAMTSL5 | 2701 | 2749 | -74.78 | lmDtoBDcommon | No |
| lnc-FMO2-1:1 | 517 | 580 | ADAMTSL5 | 2815 | 2878 | -98.26 | lmDtoBDcommon | No |
| lnc-GLT6D1-2:1 | 1420 | 1475 | ADAMTSL5 | 2838 | 2893 | -84.49 | lmDtoBDcommon | No |
| lnc-HPS6-1:1 | 406 | 457 | ADAMTSL5 | 2834 | 2887 | -74.06 | lmDtoBDcommon | No |
| lnc-IFNK-7:1 | 5112 | 5180 | ADAMTSL5 | 2682 | 2750 | -100.83 | lmDtoBDcommon | No |
| lnc-IL22RA2-1:1 | 1185 | 1238 | ADAMTSL5 | 2697 | 2750 | -88.84 | lmDtoBDcommon | No |
| lnc-JPH2-1:1 | 2132 | 2191 | ADAMTSL5 | 2834 | 2893 | -94.71 | lmDtoBDcommon | No |
| lnc-KDM4C-1:3 | 366 | 419 | ADAMTSL5 | 2845 | 2895 | -80.09 | lmDtoBDcommon | No |
| lnc-LEPROTL1-4:7 | 2708 | 2761 | ADAMTSL5 | 2642 | 2692 | -64.73 | lmDtoBDcommon | No |
| lnc-LETM2-4:1 | 1619 | 1680 | ADAMTSL5 | 2815 | 2876 | -94.21 | lmDtoBDcommon | No |
| lnc-LRRC9-5:1 | 2108 | 2158 | ADAMTSL5 | 2639 | 2689 | -77.36 | lmDtoBDcommon | No |
| lnc-MLXIP-1:1 | 1021 | 1088 | ADAMTSL5 | 2683 | 2750 | -97.56 | lmDtoBDcommon | No |
| lnc-MRPL33-1:2 | 775 | 840 | ADAMTSL5 | 2834 | 2896 | -93.56 | lmDtoBDcommon | No |
| lnc-MRPL35-1:2 | 709 | 763 | ADAMTSL5 | 2677 | 2731 | -97.85 | lmDtoBDcommon | No |
| lnc-MZT2B-1:2 | 275 | 326 | ADAMTSL5 | 2834 | 2887 | -64.93 | lmDtoBDcommon | No |
| lnc-NNT-5:1 | 4835 | 4899 | ADAMTSL5 | 2649 | 2713 | -109.16 | lmDtoBDcommon | No |
| lnc-NPAS1-3:1 | 174 | 242 | ADAMTSL5 | 2682 | 2750 | -96.59 | lmDtoBDcommon | No |
| lnc-NR4A2-3:1 | 5 | 65 | ADAMTSL5 | 2838 | 2895 | -85.94 | lmDtoBDcommon | No |
| lnc-OSBPL2-1:1 | 1475 | 1543 | ADAMTSL5 | 2682 | 2750 | -100.73 | lmDtoBDcommon | No |
| lnc-OST4-2:1 | 1740 | 1793 | ADAMTSL5 | 2838 | 2893 | -83.31 | lmDtoBDcommon | No |
| lnc-PARP8-2:2 | 4505 | 4566 | ADAMTSL5 | 2839 | 2900 | -76.62 | lmDtoBDcommon | No |
| lnc-PAWR-13:1 | 682 | 734 | ADAMTSL5 | 2658 | 2710 | -84.42 | lmDtoBDcommon | No |
| lnc-PLAGL2-6:1 | 2748 | 2820 | ADAMTSL5 | 2678 | 2750 | -134.55 | lmDtoBDcommon | No |
| lnc-PRPF4B-4:5 | 546 | 603 | ADAMTSL5 | 2834 | 2893 | -87.26 | lmDtoBDcommon | No |
| lnc-RAB28-6:1 | 877 | 930 | ADAMTSL5 | 2834 | 2887 | -77.85 | lmDtoBDcommon | No |
| lnc-RABEP2-2:1 | 178 | 250 | ADAMTSL5 | 2678 | 2749 | -112.42 | lmDtoBDcommon | No |
| lnc-RNF182-2:1 | 204 | 274 | ADAMTSL5 | 2679 | 2749 | -112.64 | lmDtoBDcommon | No |
| lnc-RPF1-1:11 | 1038 | 1110 | ADAMTSL5 | 2678 | 2750 | -116.65 | lmDtoBDcommon | No |
| lnc-RPL6-1:1 | 181 | 235 | ADAMTSL5 | 2636 | 2690 | -86.99 | lmDtoBDcommon | No |
| lnc-S100P-1:1 | 3093 | 3156 | ADAMTSL5 | 2834 | 2894 | -89.36 | lmDtoBDcommon | No |
| lnc-SFTA2-14:1 | 3533 | 3586 | ADAMTSL5 | 2639 | 2692 | -87.44 | lmDtoBDcommon | No |
| lnc-SLC12A7-4:3 | 1397 | 1455 | ADAMTSL5 | 2838 | 2895 | -81.06 | lmDtoBDcommon | No |
| lnc-SLC29A1-2:1 | 3355 | 3423 | ADAMTSL5 | 2677 | 2745 | -138.39 | lmDtoBDcommon | No |
| lnc-STX2-13:1 | 660 | 732 | ADAMTSL5 | 2678 | 2750 | -122.98 | lmDtoBDcommon | No |
| lnc-TAL1-3:2 | 1115 | 1174 | ADAMTSL5 | 2839 | 2895 | -85.16 | lmDtoBDcommon | No |
| lnc-TBC1D12-5:1 | 546 | 595 | ADAMTSL5 | 2640 | 2688 | -62.47 | lmDtoBDcommon | No |
| lnc-TBC1D20-1:1 | 963 | 1016 | ADAMTSL5 | 2640 | 2692 | -76.64 | lmDtoBDcommon | No |
| lnc-TSTA3-4:1 | 338 | 387 | ADAMTSL5 | 2639 | 2688 | -74.79 | lmDtoBDcommon | No |
| lnc-TTC23-5:1 | 2871 | 2924 | ADAMTSL5 | 2696 | 2750 | -77.35 | lmDtoBDcommon | No |
| lnc-TUBB2B-8:4 | 415 | 471 | ADAMTSL5 | 2834 | 2890 | -89.57 | lmDtoBDcommon | No |
| lnc-TYMS-3:1 | 1642 | 1694 | ADAMTSL5 | 2639 | 2691 | -89.94 | lmDtoBDcommon | No |
| lnc-U2AF1L4-1:9 | 821 | 869 | ADAMTSL5 | 2845 | 2893 | -74.24 | lmDtoBDcommon | No |
| lnc-VASN-1:1 | 435 | 493 | ADAMTSL5 | 2838 | 2893 | -86.85 | lmDtoBDcommon | No |
| lnc-WDR19-1:1 | 3295 | 3357 | ADAMTSL5 | 2683 | 2745 | -93.73 | lmDtoBDcommon | No |
| lnc-WDR34-2:3 | 5342 | 5412 | ADAMTSL5 | 2680 | 2750 | -120.63 | lmDtoBDcommon | No |
| lnc-WRNIP1-28:1 | 669 | 725 | ADAMTSL5 | 2834 | 2890 | -97.51 | lmDtoBDcommon | No |
| lnc-ZC3HC1-1:1 | 33 | 86 | ADAMTSL5 | 2640 | 2692 | -81.54 | lmDtoBDcommon | No |
| lnc-ZG16-2:1 | 176 | 225 | ADAMTSL5 | 2829 | 2878 | -88.01 | lmDtoBDcommon | No |
| lnc-ZNF771-1:1 | 869 | 933 | ADAMTSL5 | 2678 | 2742 | -96.55 | lmDtoBDcommon | No |
| PRKAR2A-AS1:8 | 2102 | 2155 | ADAMTSL5 | 2697 | 2750 | -94.24 | lmDtoBDcommon | No |
| SIRPG-AS1:1 | 1200 | 1260 | ADAMTSL5 | 2838 | 2895 | -88.04 | lmDtoBDcommon | No |
| TCONS_00048679 | 27 | 67 | ADAMTSL5 | 45 | 89 | -61.88 | lmDtoBDcommon | No |
| ZEB1-AS1:13 | 93 | 160 | ADAMTSL5 | 2683 | 2750 | -99.62 | lmDtoBDcommon | No |
| DLX6-AS1:13 | 102 | 150 | COL26A1 | 1733 | 1776 | -53.11 | lmBtoBDcommon | Yes |
| LINC00299:3 | 1 | 45 | COL26A1 | 2779 | 2824 | -50.05 | lmBtoBDcommon | Yes |
| lnc-AKAP11-1:2 | 1785 | 1832 | COL26A1 | 2581 | 2624 | -52.9 | lmBtoBDcommon | Yes |
| lnc-CGNL1-6:1 | 39 | 83 | COL26A1 | 904 | 944 | -57.35 | lmBtoBDcommon | Yes |
| lnc-HIST1H2AI-1:3 | 1141 | 1183 | COL26A1 | 440 | 487 | -58.29 | lmBtoBDcommon | Yes |
| lnc-KIAA0226-4:1 | 156 | 204 | COL26A1 | 1733 | 1776 | -51.94 | lmBtoBDcommon | Yes |
| lnc-LRRFIP2-1:1 | 2000 | 2044 | COL26A1 | 2739 | 2778 | -53.62 | lmBtoBDcommon | Yes |
| lnc-PGPEP1L-3:1 | 3794 | 3841 | COL26A1 | 2581 | 2624 | -52.97 | lmBtoBDcommon | Yes |
| lnc-RPRML-3:20 | 1458 | 1510 | COL26A1 | 808 | 857 | -60.44 | lmBtoBDcommon | Yes |
| lnc-TULP2-2:1 | 142 | 189 | COL26A1 | 2149 | 2196 | -56.24 | lmBtoBDcommon | Yes |
| lnc-ZNF114-2:1 | 802 | 843 | COL26A1 | 2593 | 2636 | -52.25 | lmBtoBDcommon | Yes |
| TCONS_00052318 | 29 | 75 | COL26A1 | 115 | 154 | -57.22 | lmBtoBDcommon | Yes |
| TCONS_00052868 | 558 | 605 | COL26A1 | 234 | 281 | -58.74 | lmBtoBDcommon | Yes |
| lnc-CACYBP-2:1 | 1503 | 1550 | COL26A1 | 2581 | 2624 | -53.7 | lmDtoBDcommon | Yes |
| lnc-CSNK1D-1:1 | 204 | 244 | COL26A1 | 796 | 845 | -65.42 | lmDtoBDcommon | Yes |
| lnc-FAM32A-2:1 | 115 | 161 | COL26A1 | 2145 | 2190 | -59.22 | lmDtoBDcommon | Yes |
| lnc-LRRFIP2-1:1 | 2000 | 2044 | COL26A1 | 2739 | 2778 | -53.62 | lmDtoBDcommon | Yes |
| lnc-PLEKHA7-2:1 | 1551 | 1597 | COL26A1 | 2138 | 2188 | -53.23 | lmDtoBDcommon | Yes |
| lnc-RPRML-3:20 | 1458 | 1510 | COL26A1 | 808 | 857 | -60.44 | lmDtoBDcommon | Yes |
| lnc-ZFAND4-1:1 | 26 | 72 | COL26A1 | 236 | 279 | -63.24 | lmDtoBDcommon | Yes |
| CHKB-AS1:14 | 2688 | 2735 | COL26A1 | 2581 | 2624 | -59.4 | lmBtoBDcommon | No |
| DANT1:2 | 12002 | 12047 | COL26A1 | 1150 | 1199 | -54.13 | lmBtoBDcommon | No |
| GAS6-AS2:1 | 195 | 243 | COL26A1 | 1733 | 1776 | -54.25 | lmBtoBDcommon | No |
| LINC00847:14 | 174 | 219 | COL26A1 | 909 | 946 | -52.31 | lmBtoBDcommon | No |
| LINC00887:5 | 916 | 956 | COL26A1 | 1712 | 1760 | -53.66 | lmBtoBDcommon | No |
| LINC00893:4 | 487 | 534 | COL26A1 | 2935 | 2982 | -53.3 | lmBtoBDcommon | No |
| LINC00920:3 | 217 | 260 | COL26A1 | 224 | 270 | -50.38 | lmBtoBDcommon | No |
| LINC01299:2 | 2224 | 2264 | COL26A1 | 2593 | 2636 | -50.32 | lmBtoBDcommon | No |
| lnc-ABCC6-2:1 | 849 | 897 | COL26A1 | 334 | 381 | -57.78 | lmBtoBDcommon | No |
| lnc-ABHD2-4:1 | 397 | 445 | COL26A1 | 2117 | 2163 | -67.76 | lmBtoBDcommon | No |
| lnc-AC091132.2-7:1 | 8 | 58 | COL26A1 | 945 | 992 | -60.53 | lmBtoBDcommon | No |
| lnc-ADA-2:1 | 471 | 519 | COL26A1 | 1733 | 1776 | -51.26 | lmBtoBDcommon | No |
| lnc-AMZ2-3:1 | 1783 | 1825 | COL26A1 | 902 | 937 | -54.31 | lmBtoBDcommon | No |
| lnc-ANKRD30B-7:2 | 285 | 330 | COL26A1 | 2078 | 2121 | -51.89 | lmBtoBDcommon | No |
| lnc-APBA2-5:8 | 93 | 136 | COL26A1 | 2708 | 2759 | -65.6 | lmBtoBDcommon | No |
| lnc-BCL9L-1:1 | 880 | 927 | COL26A1 | 2581 | 2624 | -54.32 | lmBtoBDcommon | No |
| lnc-C14orf79-3:1 | 797 | 842 | COL26A1 | 897 | 940 | -56.49 | lmBtoBDcommon | No |
| lnc-C3orf62-2:1 | 68 | 115 | COL26A1 | 2581 | 2624 | -53.03 | lmBtoBDcommon | No |
| lnc-CARKD-4:1 | 103 | 146 | COL26A1 | 902 | 947 | -54.14 | lmBtoBDcommon | No |
| lnc-CLCN1-2:1 | 173 | 216 | COL26A1 | 1765 | 1805 | -61.13 | lmBtoBDcommon | No |
| lnc-CLIC3-2:1 | 319 | 365 | COL26A1 | 2097 | 2144 | -57.34 | lmBtoBDcommon | No |
| lnc-DDX52-3:1 | 828 | 879 | COL26A1 | 965 | 1012 | -62.05 | lmBtoBDcommon | No |
| lnc-EXOC2-8:4 | 1724 | 1770 | COL26A1 | 1112 | 1158 | -51.3 | lmBtoBDcommon | No |
| lnc-FRYL-3:3 | 119 | 166 | COL26A1 | 145 | 195 | -59.34 | lmBtoBDcommon | No |
| lnc-HEATR2-3:1 | 1351 | 1397 | COL26A1 | 963 | 1006 | -61 | lmBtoBDcommon | No |
| lnc-HSPG2-2:1 | 29 | 75 | COL26A1 | 2579 | 2624 | -52.36 | lmBtoBDcommon | No |
| lnc-HTRA2-1:1 | 425 | 471 | COL26A1 | 124 | 170 | -55.29 | lmBtoBDcommon | No |
| lnc-KLF13-2:4 | 1509 | 1558 | COL26A1 | 980 | 1024 | -57.55 | lmBtoBDcommon | No |
| lnc-KLHL5-2:1 | 1824 | 1871 | COL26A1 | 2581 | 2624 | -54.7 | lmBtoBDcommon | No |
| lnc-MAPK6-4:2 | 326 | 372 | COL26A1 | 2866 | 2915 | -50.84 | lmBtoBDcommon | No |
| lnc-MOGAT2-3:1 | 97 | 144 | COL26A1 | 801 | 847 | -69.92 | lmBtoBDcommon | No |
| lnc-NT5C-3:3 | 229 | 271 | COL26A1 | 2119 | 2159 | -52.18 | lmBtoBDcommon | No |
| lnc-PAIP2-1:1 | 40 | 88 | COL26A1 | 987 | 1029 | -53.73 | lmBtoBDcommon | No |
| lnc-PCBP3-3:9 | 180 | 230 | COL26A1 | 2171 | 2221 | -53.85 | lmBtoBDcommon | No |
| lnc-POTEF-8:2 | 2036 | 2084 | COL26A1 | 2361 | 2410 | -50.05 | lmBtoBDcommon | No |
| lnc-PRDM11-9:2 | 225 | 264 | COL26A1 | 331 | 375 | -54.06 | lmBtoBDcommon | No |
| lnc-PUF60-1:1 | 333 | 373 | COL26A1 | 1566 | 1604 | -52.68 | lmBtoBDcommon | No |
| lnc-RBAK-2:10 | 465 | 506 | COL26A1 | 1137 | 1185 | -52.76 | lmBtoBDcommon | No |
| lnc-RBPMS2-1:2 | 141 | 189 | COL26A1 | 1106 | 1151 | -59.26 | lmBtoBDcommon | No |
| lnc-RCN1-2:1 | 489 | 537 | COL26A1 | 1733 | 1776 | -50.33 | lmBtoBDcommon | No |
| lnc-RUNDC3A-4:2 | 94 | 135 | COL26A1 | 211 | 254 | -51.56 | lmBtoBDcommon | No |
| lnc-SEC11A-1:3 | 1230 | 1271 | COL26A1 | 2593 | 2636 | -50.81 | lmBtoBDcommon | No |
| lnc-SEC11A-2:1 | 89 | 145 | COL26A1 | 979 | 1032 | -62.45 | lmBtoBDcommon | No |
| lnc-SIX5-1:1 | 653 | 699 | COL26A1 | 901 | 947 | -60.63 | lmBtoBDcommon | No |
| lnc-TAOK3-1:11 | 2645 | 2693 | COL26A1 | 1733 | 1776 | -53.45 | lmBtoBDcommon | No |
| lnc-TOR3A-1:1 | 2354 | 2409 | COL26A1 | 1896 | 1947 | -54.71 | lmBtoBDcommon | No |
| lnc-TYSND1-1:7 | 1505 | 1554 | COL26A1 | 833 | 873 | -63.56 | lmBtoBDcommon | No |
| lnc-WDR73-7:2 | 26 | 70 | COL26A1 | 2613 | 2658 | -55.2 | lmBtoBDcommon | No |
| lnc-ZNF827-11:1 | 12 | 58 | COL26A1 | 810 | 857 | -56.02 | lmBtoBDcommon | No |
| PART1:10 | 866 | 905 | COL26A1 | 1733 | 1774 | -53.31 | lmBtoBDcommon | No |
| PCF11-AS1:2 | 19 | 65 | COL26A1 | 966 | 1009 | -57 | lmBtoBDcommon | No |
| PSMB8-AS1:1 | 601 | 646 | COL26A1 | 1653 | 1699 | -50.89 | lmBtoBDcommon | No |
| PSMB8-AS1:5 | 662 | 707 | COL26A1 | 1653 | 1699 | -50.89 | lmBtoBDcommon | No |
| ST3GAL5-AS1:4 | 1534 | 1587 | COL26A1 | 2130 | 2180 | -64.69 | lmBtoBDcommon | No |
| TCONS_00052860 | 684 | 731 | COL26A1 | 234 | 281 | -58.74 | lmBtoBDcommon | No |
| DNAJC27-AS1:8 | 3703 | 3745 | COL26A1 | 41 | 86 | -55.09 | lmDtoBDcommon | No |
| EMX2OS:2 | 4616 | 4662 | COL26A1 | 904 | 949 | -63.82 | lmDtoBDcommon | No |
| lnc-AC115989.1.1-5:1 | 506 | 547 | COL26A1 | 2625 | 2669 | -55.44 | lmDtoBDcommon | No |
| lnc-C16orf5-1:1 | 426 | 474 | COL26A1 | 1733 | 1776 | -53.07 | lmDtoBDcommon | No |
| lnc-C22orf46-2:1 | 353 | 398 | COL26A1 | 2593 | 2639 | -56.8 | lmDtoBDcommon | No |
| lnc-C7orf55-1:2 | 279 | 327 | COL26A1 | 967 | 1018 | -50 | lmDtoBDcommon | No |
| lnc-CIDEA-2:3 | 1389 | 1436 | COL26A1 | 2581 | 2624 | -53.71 | lmDtoBDcommon | No |
| lnc-CPSF2-3:1 | 1698 | 1743 | COL26A1 | 672 | 721 | -52.07 | lmDtoBDcommon | No |
| lnc-CRK-3:4 | 22 | 68 | COL26A1 | 905 | 949 | -57.85 | lmDtoBDcommon | No |
| lnc-FAIM-3:1 | 246 | 294 | COL26A1 | 1733 | 1776 | -50.63 | lmDtoBDcommon | No |
| lnc-GLT6D1-2:1 | 723 | 764 | COL26A1 | 176 | 220 | -52.43 | lmDtoBDcommon | No |
| lnc-HPS6-1:1 | 939 | 979 | COL26A1 | 1730 | 1769 | -57.99 | lmDtoBDcommon | No |
| lnc-LETM2-4:1 | 2143 | 2194 | COL26A1 | 896 | 945 | -61.78 | lmDtoBDcommon | No |
| lnc-MDM1-1:1 | 31 | 74 | COL26A1 | 1452 | 1496 | -52.81 | lmDtoBDcommon | No |
| lnc-MDM1-1:15 | 1935 | 1981 | COL26A1 | 2115 | 2157 | -51.48 | lmDtoBDcommon | No |
| lnc-MZT2B-1:2 | 648 | 692 | COL26A1 | 2101 | 2149 | -59.22 | lmDtoBDcommon | No |
| lnc-OST4-2:1 | 477 | 519 | COL26A1 | 807 | 851 | -56.89 | lmDtoBDcommon | No |
| lnc-PARP8-2:2 | 1184 | 1231 | COL26A1 | 258 | 305 | -50.65 | lmDtoBDcommon | No |
| lnc-PLA2G10-4:1 | 29 | 76 | COL26A1 | 2581 | 2624 | -54 | lmDtoBDcommon | No |
| lnc-RP11-62N21.1.1-5:2 | 20 | 61 | COL26A1 | 102 | 150 | -55.02 | lmDtoBDcommon | No |
| lnc-SLC12A7-4:3 | 565 | 607 | COL26A1 | 923 | 972 | -60.82 | lmDtoBDcommon | No |
| lnc-TTC23-5:1 | 56 | 103 | COL26A1 | 2126 | 2169 | -62.85 | lmDtoBDcommon | No |
| lnc-U2AF1L4-1:9 | 1631 | 1680 | COL26A1 | 1734 | 1781 | -54.39 | lmDtoBDcommon | No |
| lnc-VASN-1:1 | 84 | 131 | COL26A1 | 2366 | 2416 | -60.43 | lmDtoBDcommon | No |
| lnc-ZNF771-1:1 | 1839 | 1886 | COL26A1 | 2581 | 2624 | -51.7 | lmDtoBDcommon | No |
| SIRPG-AS1:1 | 1544 | 1589 | COL26A1 | 1731 | 1781 | -58.09 | lmDtoBDcommon | No |
| LINC01237:6 | 20 | 66 | COL27A1 | 3831 | 3869 | -54.33 | lmBtoBDcommon | Yes |
| lnc-AMZ2-5:1 | 2473 | 2520 | COL27A1 | 6952 | 6996 | -61.91 | lmBtoBDcommon | Yes |
| lnc-ARL1-3:1 | 3205 | 3245 | COL27A1 | 705 | 752 | -50.25 | lmBtoBDcommon | Yes |
| lnc-C12orf50-6:2 | 382 | 430 | COL27A1 | 1433 | 1477 | -51.6 | lmBtoBDcommon | Yes |
| lnc-CSNK1D-1:1 | 174 | 212 | COL27A1 | 5687 | 5727 | -63.44 | lmBtoBDcommon | Yes |
| lnc-EGLN1-1:3 | 287 | 330 | COL27A1 | 4662 | 4702 | -50.01 | lmBtoBDcommon | Yes |
| lnc-EID2B-1:1 | 107 | 151 | COL27A1 | 3975 | 4020 | -53.04 | lmBtoBDcommon | Yes |
| lnc-FBXL2-4:1 | 88 | 129 | COL27A1 | 533 | 579 | -55.02 | lmBtoBDcommon | Yes |
| lnc-GGCT-1:12 | 501 | 549 | COL27A1 | 519 | 560 | -55.07 | lmBtoBDcommon | Yes |
| lnc-HES5-1:7 | 43 | 82 | COL27A1 | 6029 | 6075 | -62.49 | lmBtoBDcommon | Yes |
| lnc-HIST1H2AI-1:3 | 896 | 943 | COL27A1 | 9503 | 9547 | -55.3 | lmBtoBDcommon | Yes |
| lnc-HIVEP3-1:1 | 2 | 44 | COL27A1 | 629 | 676 | -65.49 | lmBtoBDcommon | Yes |
| lnc-LRRFIP2-1:1 | 2186 | 2234 | COL27A1 | 2252 | 2299 | -50.96 | lmBtoBDcommon | Yes |
| lnc-MDK-4:2 | 377 | 420 | COL27A1 | 5778 | 5818 | -71.13 | lmBtoBDcommon | Yes |
| lnc-MPPE1-5:1 | 70 | 121 | COL27A1 | 499 | 549 | -53.18 | lmBtoBDcommon | Yes |
| lnc-NPBWR1-1:2 | 664 | 708 | COL27A1 | 529 | 572 | -52.34 | lmBtoBDcommon | Yes |
| lnc-PGPEP1L-3:1 | 1039 | 1084 | COL27A1 | 8892 | 8935 | -58.29 | lmBtoBDcommon | Yes |
| lnc-PLEKHA7-2:1 | 1536 | 1582 | COL27A1 | 537 | 583 | -59.56 | lmBtoBDcommon | Yes |
| lnc-RP11-706O15.1.1-2:8 | 235 | 276 | COL27A1 | 538 | 575 | -56.39 | lmBtoBDcommon | Yes |
| lnc-RPRML-3:20 | 1832 | 1881 | COL27A1 | 4232 | 4279 | -58.19 | lmBtoBDcommon | Yes |
| lnc-RPUSD2-2:1 | 2028 | 2076 | COL27A1 | 274 | 317 | -54.22 | lmBtoBDcommon | Yes |
| lnc-TULP2-2:1 | 149 | 191 | COL27A1 | 3976 | 4020 | -50.56 | lmBtoBDcommon | Yes |
| lnc-VASH2-1:1 | 254 | 293 | COL27A1 | 429 | 472 | -52.36 | lmBtoBDcommon | Yes |
| lnc-WDR7-6:2 | 371 | 410 | COL27A1 | 8024 | 8063 | -55.65 | lmBtoBDcommon | Yes |
| lnc-ZNF91-4:2 | 538 | 583 | COL27A1 | 519 | 566 | -53 | lmBtoBDcommon | Yes |
| TCL6:19 | 2517 | 2565 | COL27A1 | 5768 | 5817 | -71 | lmBtoBDcommon | Yes |
| TCONS_00001685 | 1707 | 1754 | COL27A1 | 4308 | 4355 | -60.73 | lmBtoBDcommon | Yes |
| TCONS_00048671 | 21 | 74 | COL27A1 | 430 | 478 | -71.37 | lmBtoBDcommon | Yes |
| TCONS_00052862 | 645 | 694 | COL27A1 | 1892 | 1940 | -77.63 | lmBtoBDcommon | Yes |
| TCONS_00052867 | 590 | 639 | COL27A1 | 1892 | 1940 | -77.63 | lmBtoBDcommon | Yes |
| lnc-EGLN1-1:3 | 287 | 330 | COL27A1 | 4662 | 4702 | -50.01 | lmDtoBDcommon | Yes |
| APTR:11 | 33 | 78 | COL27A1 | 238 | 283 | -54.56 | lmBtoBDcommon | No |
| CHKB-AS1:14 | 49 | 95 | COL27A1 | 4721 | 4763 | -52 | lmBtoBDcommon | No |
| DANT1:2 | 1611 | 1658 | COL27A1 | 622 | 665 | -60.59 | lmBtoBDcommon | No |
| FAM201A:1 | 129 | 170 | COL27A1 | 3503 | 3549 | -62.52 | lmBtoBDcommon | No |
| LBX2-AS1:2 | 716 | 761 | COL27A1 | 3767 | 3808 | -63.75 | lmBtoBDcommon | No |
| LINC00476:3 | 508 | 553 | COL27A1 | 499 | 546 | -54.82 | lmBtoBDcommon | No |
| LINC00649:8 | 26 | 76 | COL27A1 | 3567 | 3620 | -58.63 | lmBtoBDcommon | No |
| LINC00654:4 | 224 | 273 | COL27A1 | 5805 | 5853 | -62.72 | lmBtoBDcommon | No |
| LINC00893:4 | 63 | 104 | COL27A1 | 3597 | 3645 | -50.46 | lmBtoBDcommon | No |
| LINC01426:3 | 101 | 147 | COL27A1 | 2285 | 2332 | -57.04 | lmBtoBDcommon | No |
| lnc-ABCC6-2:1 | 24 | 63 | COL27A1 | 1153 | 1198 | -57.37 | lmBtoBDcommon | No |
| lnc-ABHD2-4:1 | 756 | 804 | COL27A1 | 5684 | 5735 | -64.97 | lmBtoBDcommon | No |
| lnc-AC006455.1-10:1 | 563 | 609 | COL27A1 | 3949 | 3997 | -51.04 | lmBtoBDcommon | No |
| lnc-ACSBG2-2:1 | 280 | 325 | COL27A1 | 628 | 672 | -50.76 | lmBtoBDcommon | No |
| lnc-AIF1-2:1 | 602 | 645 | COL27A1 | 540 | 588 | -59.25 | lmBtoBDcommon | No |
| lnc-AL669831.1-3:42 | 14 | 62 | COL27A1 | 537 | 574 | -59.6 | lmBtoBDcommon | No |
| lnc-AMZ2-3:1 | 96 | 137 | COL27A1 | 529 | 575 | -50.9 | lmBtoBDcommon | No |
| lnc-APH1A-1:6 | 206 | 247 | COL27A1 | 1933 | 1974 | -52.91 | lmBtoBDcommon | No |
| lnc-B3GNT9-1:1 | 249 | 293 | COL27A1 | 653 | 696 | -66.19 | lmBtoBDcommon | No |
| lnc-BCL2L2-PABPN1-1:2 | 306 | 353 | COL27A1 | 2658 | 2702 | -61.8 | lmBtoBDcommon | No |
| lnc-BRI3-2:3 | 95 | 144 | COL27A1 | 6040 | 6087 | -58.27 | lmBtoBDcommon | No |
| lnc-C10orf90-2:2 | 196 | 238 | COL27A1 | 479 | 523 | -65.3 | lmBtoBDcommon | No |
| lnc-C11orf94-1:1 | 276 | 317 | COL27A1 | 5799 | 5841 | -60.45 | lmBtoBDcommon | No |
| lnc-C14orf79-3:1 | 787 | 829 | COL27A1 | 2764 | 2809 | -57.82 | lmBtoBDcommon | No |
| lnc-C15orf39-1:1 | 1271 | 1315 | COL27A1 | 472 | 515 | -58.44 | lmBtoBDcommon | No |
| lnc-C16orf72-7:1 | 767 | 813 | COL27A1 | 3483 | 3532 | -57.62 | lmBtoBDcommon | No |
| lnc-C17orf109-3:1 | 54 | 92 | COL27A1 | 6346 | 6384 | -63.09 | lmBtoBDcommon | No |
| lnc-C17orf77-1:6 | 294 | 342 | COL27A1 | 8023 | 8067 | -50.68 | lmBtoBDcommon | No |
| lnc-C1orf138-1:1 | 137 | 182 | COL27A1 | 5777 | 5827 | -70.1 | lmBtoBDcommon | No |
| lnc-C1orf186-3:1 | 465 | 510 | COL27A1 | 1929 | 1974 | -67.07 | lmBtoBDcommon | No |
| lnc-C20orf24-3:1 | 245 | 294 | COL27A1 | 5183 | 5230 | -50.36 | lmBtoBDcommon | No |
| lnc-C3orf62-2:1 | 83 | 128 | COL27A1 | 4017 | 4064 | -50.82 | lmBtoBDcommon | No |
| lnc-CAMK1-1:2 | 428 | 470 | COL27A1 | 4090 | 4133 | -58.11 | lmBtoBDcommon | No |
| lnc-CEP170-9:2 | 3359 | 3426 | COL27A1 | 8689 | 8762 | -59.21 | lmBtoBDcommon | No |
| lnc-CLDN23-3:1 | 576 | 630 | COL27A1 | 648 | 699 | -64.85 | lmBtoBDcommon | No |
| lnc-CLDN9-1:1 | 2 | 50 | COL27A1 | 654 | 697 | -66.66 | lmBtoBDcommon | No |
| lnc-CLIC3-2:1 | 288 | 333 | COL27A1 | 6091 | 6132 | -58.3 | lmBtoBDcommon | No |
| lnc-COX19-2:2 | 733 | 771 | COL27A1 | 2038 | 2080 | -55.59 | lmBtoBDcommon | No |
| lnc-CRYBA4-1:55 | 4843 | 4889 | COL27A1 | 5777 | 5825 | -67.95 | lmBtoBDcommon | No |
| lnc-CSNK1D-2:9 | 35 | 72 | COL27A1 | 864 | 910 | -50.97 | lmBtoBDcommon | No |
| lnc-DDX52-3:1 | 829 | 876 | COL27A1 | 1928 | 1964 | -52.33 | lmBtoBDcommon | No |
| lnc-DPY19L3-1:1 | 63 | 110 | COL27A1 | 483 | 528 | -71.32 | lmBtoBDcommon | No |
| lnc-GRID2-1:1 | 413 | 463 | COL27A1 | 3025 | 3074 | -58.1 | lmBtoBDcommon | No |
| lnc-GTPBP1-1:1 | 100 | 143 | COL27A1 | 4558 | 4603 | -55.69 | lmBtoBDcommon | No |
| lnc-HEATR2-3:1 | 2280 | 2328 | COL27A1 | 426 | 475 | -72.23 | lmBtoBDcommon | No |
| lnc-IFT52-1:2 | 53 | 98 | COL27A1 | 2770 | 2820 | -63.49 | lmBtoBDcommon | No |
| lnc-KIAA0355-5:1 | 922 | 964 | COL27A1 | 3625 | 3664 | -50.89 | lmBtoBDcommon | No |
| lnc-KLF14-1:1 | 1214 | 1265 | COL27A1 | 7255 | 7308 | -56.34 | lmBtoBDcommon | No |
| lnc-LAT-1:9 | 398 | 445 | COL27A1 | 3956 | 4004 | -55.88 | lmBtoBDcommon | No |
| lnc-LIPI-9:1 | 463 | 512 | COL27A1 | 538 | 582 | -53.57 | lmBtoBDcommon | No |
| lnc-LONRF2-3:1 | 192 | 236 | COL27A1 | 553 | 601 | -50.38 | lmBtoBDcommon | No |
| lnc-MOGAT2-3:1 | 122 | 169 | COL27A1 | 494 | 541 | -65.45 | lmBtoBDcommon | No |
| lnc-NRARP-3:1 | 1438 | 1482 | COL27A1 | 478 | 528 | -60.33 | lmBtoBDcommon | No |
| lnc-OLFML3-5:1 | 2993 | 3042 | COL27A1 | 3634 | 3684 | -54.12 | lmBtoBDcommon | No |
| lnc-PAIP2-1:1 | 130 | 172 | COL27A1 | 7399 | 7448 | -52.08 | lmBtoBDcommon | No |
| lnc-PCBP3-3:9 | 322 | 371 | COL27A1 | 2344 | 2391 | -56.8 | lmBtoBDcommon | No |
| lnc-POFUT2-4:1 | 360 | 405 | COL27A1 | 454 | 502 | -56.95 | lmBtoBDcommon | No |
| lnc-POFUT2-5:1 | 1720 | 1768 | COL27A1 | 1924 | 1974 | -71.8 | lmBtoBDcommon | No |
| lnc-PRAGMIN.1-3:2 | 36 | 84 | COL27A1 | 5778 | 5826 | -68.08 | lmBtoBDcommon | No |
| lnc-PRKRIR-1:5 | 54 | 103 | COL27A1 | 4671 | 4714 | -50.91 | lmBtoBDcommon | No |
| lnc-PTGER1-1:2 | 488 | 540 | COL27A1 | 4058 | 4111 | -61.92 | lmBtoBDcommon | No |
| lnc-PTPN12-1:1 | 1531 | 1571 | COL27A1 | 3519 | 3563 | -54.04 | lmBtoBDcommon | No |
| lnc-PUF60-1:1 | 318 | 352 | COL27A1 | 5675 | 5721 | -63.98 | lmBtoBDcommon | No |
| lnc-RABGEF1-1:1 | 1649 | 1695 | COL27A1 | 5157 | 5205 | -56.05 | lmBtoBDcommon | No |
| lnc-RBAK-2:10 | 450 | 496 | COL27A1 | 3570 | 3618 | -58.01 | lmBtoBDcommon | No |
| lnc-RCN1-2:1 | 1000 | 1040 | COL27A1 | 494 | 542 | -55.77 | lmBtoBDcommon | No |
| lnc-RIPK1-1:2 | 2360 | 2396 | COL27A1 | 433 | 472 | -53.45 | lmBtoBDcommon | No |
| lnc-ROPN1L-3:1 | 1921 | 1960 | COL27A1 | 540 | 582 | -54.06 | lmBtoBDcommon | No |
| lnc-RPL23A-2:1 | 1043 | 1089 | COL27A1 | 2406 | 2454 | -57.62 | lmBtoBDcommon | No |
| lnc-RUNDC3A-4:2 | 145 | 188 | COL27A1 | 2173 | 2220 | -57.16 | lmBtoBDcommon | No |
| lnc-SAG-7:2 | 113 | 157 | COL27A1 | 1041 | 1084 | -51.07 | lmBtoBDcommon | No |
| lnc-SAMD8-1:1 | 969 | 1015 | COL27A1 | 1876 | 1919 | -55.33 | lmBtoBDcommon | No |
| lnc-SATB1-8:5 | 56 | 100 | COL27A1 | 5118 | 5158 | -50.81 | lmBtoBDcommon | No |
| lnc-SBDS-4:1 | 2662 | 2711 | COL27A1 | 3515 | 3564 | -66.98 | lmBtoBDcommon | No |
| lnc-SEC11A-2:1 | 115 | 163 | COL27A1 | 5806 | 5854 | -67.73 | lmBtoBDcommon | No |
| lnc-SH3BGR-2:1 | 2354 | 2395 | COL27A1 | 444 | 478 | -52.56 | lmBtoBDcommon | No |
| lnc-SIX5-1:1 | 1095 | 1150 | COL27A1 | 646 | 702 | -68.03 | lmBtoBDcommon | No |
| lnc-SLC12A8-1:1 | 203 | 246 | COL27A1 | 2799 | 2837 | -54.1 | lmBtoBDcommon | No |
| lnc-SLC26A11-1:1 | 569 | 614 | COL27A1 | 2775 | 2821 | -56.14 | lmBtoBDcommon | No |
| lnc-SLC9A7-1:2 | 349 | 395 | COL27A1 | 2166 | 2211 | -50.49 | lmBtoBDcommon | No |
| lnc-SNURF-1:35 | 2132 | 2181 | COL27A1 | 4672 | 4717 | -51.17 | lmBtoBDcommon | No |
| lnc-TACC2-3:3 | 582 | 622 | COL27A1 | 4244 | 4287 | -56.98 | lmBtoBDcommon | No |
| lnc-TAF13-2:1 | 256 | 296 | COL27A1 | 438 | 484 | -59.82 | lmBtoBDcommon | No |
| lnc-TAOK3-1:11 | 817 | 850 | COL27A1 | 1918 | 1955 | -54.82 | lmBtoBDcommon | No |
| lnc-TLR1-1:1 | 459 | 506 | COL27A1 | 5625 | 5666 | -50.53 | lmBtoBDcommon | No |
| lnc-TMEM120B-4:3 | 32 | 72 | COL27A1 | 6349 | 6393 | -58.08 | lmBtoBDcommon | No |
| lnc-TNNT1-1:1 | 1284 | 1337 | COL27A1 | 6273 | 6328 | -77.45 | lmBtoBDcommon | No |
| lnc-TOR3A-1:1 | 473 | 521 | COL27A1 | 1492 | 1531 | -59.97 | lmBtoBDcommon | No |
| lnc-TSSC4-5:2 | 15 | 49 | COL27A1 | 545 | 581 | -51.57 | lmBtoBDcommon | No |
| lnc-TTR-3:1 | 50 | 97 | COL27A1 | 440 | 487 | -62.77 | lmBtoBDcommon | No |
| lnc-TYSND1-1:7 | 378 | 425 | COL27A1 | 5792 | 5834 | -59.94 | lmBtoBDcommon | No |
| lnc-VTI1A-1:3 | 1209 | 1255 | COL27A1 | 524 | 573 | -64.76 | lmBtoBDcommon | No |
| lnc-ZNF587-1:1 | 664 | 709 | COL27A1 | 6894 | 6941 | -51.82 | lmBtoBDcommon | No |
| lnc-ZSCAN10-3:17 | 537 | 579 | COL27A1 | 492 | 532 | -53.7 | lmBtoBDcommon | No |
| lnc-ZSWIM7-3:1 | 576 | 616 | COL27A1 | 1900 | 1940 | -50.16 | lmBtoBDcommon | No |
| LOH12CR2:1 | 30 | 74 | COL27A1 | 3419 | 3466 | -54.61 | lmBtoBDcommon | No |
| MIR4435-2HG:9 | 591 | 639 | COL27A1 | 4355 | 4405 | -52.35 | lmBtoBDcommon | No |
| PSMB8-AS1:1 | 1177 | 1223 | COL27A1 | 5669 | 5713 | -56.94 | lmBtoBDcommon | No |
| PSMB8-AS1:5 | 1238 | 1284 | COL27A1 | 5669 | 5713 | -56.94 | lmBtoBDcommon | No |
| SLC25A25-AS1:15 | 10 | 56 | COL27A1 | 3978 | 4020 | -52.26 | lmBtoBDcommon | No |
| SNAI3-AS1:16 | 1278 | 1318 | COL27A1 | 3969 | 4013 | -66.98 | lmBtoBDcommon | No |
| SNHG4:3 | 9 | 52 | COL27A1 | 7057 | 7103 | -53.21 | lmBtoBDcommon | No |
| TCL6:1 | 677 | 718 | COL27A1 | 3055 | 3092 | -53.29 | lmBtoBDcommon | No |
| TCONS_00000037 | 2084 | 2130 | COL27A1 | 5541 | 5592 | -68.24 | lmBtoBDcommon | No |
| TCONS_00025364 | 957 | 1019 | COL27A1 | 7888 | 7950 | -60.38 | lmBtoBDcommon | No |
| TCONS_00031339 | 450 | 493 | COL27A1 | 1004 | 1046 | -50.35 | lmBtoBDcommon | No |
| TCONS_00046326 | 858 | 908 | COL27A1 | 7906 | 7954 | -57.72 | lmBtoBDcommon | No |
| TCONS_00052860 | 764 | 813 | COL27A1 | 655 | 697 | -85.9 | lmBtoBDcommon | No |
| THUMPD3-AS1:5 | 30 | 74 | COL27A1 | 541 | 586 | -53.13 | lmBtoBDcommon | No |
| TRAF3IP2-AS1:24 | 322 | 365 | COL27A1 | 1453 | 1498 | -58.95 | lmBtoBDcommon | No |
| ZNF528-AS1:3 | 743 | 789 | COL27A1 | 1492 | 1537 | -58.27 | lmBtoBDcommon | No |
| CCDC18-AS1:37 | 127 | 169 | COL27A1 | 3975 | 4021 | -51.78 | lmDtoBDcommon | No |
| lnc-AAMP-1:2 | 114 | 159 | COL27A1 | 3961 | 4009 | -61.51 | lmDtoBDcommon | No |
| lnc-AKT2-2:1 | 26 | 75 | COL27A1 | 540 | 586 | -50.04 | lmDtoBDcommon | No |
| lnc-CDHR4-3:1 | 3 | 44 | COL27A1 | 1933 | 1979 | -66.95 | lmDtoBDcommon | No |
| lnc-EYA2-2:1 | 46 | 91 | COL27A1 | 4672 | 4714 | -50.39 | lmDtoBDcommon | No |
| lnc-HFE2-1:1 | 257 | 299 | COL27A1 | 7906 | 7955 | -60.16 | lmDtoBDcommon | No |
| lnc-MPDZ-7:1 | 74 | 125 | COL27A1 | 8078 | 8128 | -54.73 | lmDtoBDcommon | No |
| lnc-RBM28-3:2 | 1783 | 1828 | COL27A1 | 903 | 951 | -56.98 | lmDtoBDcommon | No |
| lnc-TBC1D28-1:1 | 1372 | 1417 | CORIN | 173 | 213 | -50.72 | lmBtoBDcommon | No |
| lnc-ATL3-1:9 | 1024 | 1071 | CORIN | 180 | 221 | -55.23 | lmDtoBDcommon | No |
| lnc-AMZ2-5:1 | 1710 | 1762 | LOC105371430 | 1 | 53 | -105.46 | lmBtoBDcommon | Yes |
| lnc-ARL1-3:1 | 3169 | 3216 | LOC105371430 | 19 | 66 | -87.91 | lmBtoBDcommon | Yes |
| lnc-EID2B-1:1 | 1344 | 1396 | LOC105371430 | 1 | 53 | -95.26 | lmBtoBDcommon | Yes |
| lnc-RP11-706O15.1.1-2:8 | 32 | 84 | LOC105371430 | 1 | 53 | -66.07 | lmBtoBDcommon | Yes |
| lnc-TWIST1-1:3 | 624 | 674 | LOC105371430 | 3 | 53 | -91.51 | lmBtoBDcommon | Yes |
| TCL6:19 | 842 | 894 | LOC105371430 | 1 | 53 | -90.32 | lmBtoBDcommon | Yes |
| TCONS_00001685 | 1323 | 1366 | LOC105371430 | 361 | 397 | -53.89 | lmBtoBDcommon | Yes |
| lnc-AMZ2-5:1 | 1710 | 1762 | LOC105371430 | 1 | 53 | -105.46 | lmDtoBDcommon | Yes |
| lnc-RP11-706O15.1.1-2:8 | 32 | 84 | LOC105371430 | 1 | 53 | -66.07 | lmDtoBDcommon | Yes |
| DANT1:2 | 9946 | 9997 | LOC105371430 | 2946 | 2992 | -55 | lmBtoBDcommon | No |
| LINC00893:4 | 483 | 530 | LOC105371430 | 3076 | 3121 | -50.95 | lmBtoBDcommon | No |
| lnc-AC006455.1-10:1 | 236 | 281 | LOC105371430 | 28 | 72 | -53.24 | lmBtoBDcommon | No |
| lnc-C10orf90-2:2 | 3748 | 3801 | LOC105371430 | 3 | 55 | -92.59 | lmBtoBDcommon | No |
| lnc-KLC2-2:1 | 1751 | 1809 | LOC105371430 | 17 | 72 | -96.41 | lmBtoBDcommon | No |
| lnc-LPCAT1-3:2 | 551 | 597 | LOC105371430 | 1 | 47 | -64.12 | lmBtoBDcommon | No |
| lnc-PRAGMIN.1-3:2 | 229 | 270 | LOC105371430 | 3680 | 3732 | -52.05 | lmBtoBDcommon | No |
| lnc-RP3-369A17.5.1-3:1 | 1054 | 1116 | LOC105371430 | 17 | 76 | -91.27 | lmBtoBDcommon | No |
| lnc-SAMD8-1:1 | 1119 | 1168 | LOC105371430 | 17 | 66 | -81.91 | lmBtoBDcommon | No |
| lnc-SEC11A-1:3 | 1201 | 1259 | LOC105371430 | 17 | 75 | -95.35 | lmBtoBDcommon | No |
| lnc-SERPINB6-1:7 | 1382 | 1429 | LOC105371430 | 1 | 53 | -71.59 | lmBtoBDcommon | No |
| lnc-ZSCAN10-3:17 | 1143 | 1195 | LOC105371430 | 1 | 53 | -73.54 | lmBtoBDcommon | No |
| SLC25A25-AS1:15 | 55 | 105 | LOC105371430 | 3 | 53 | -84.89 | lmBtoBDcommon | No |
| TCONS_00000037 | 6484 | 6529 | LOC105371430 | 3 | 48 | -66.04 | lmBtoBDcommon | No |
| XIST:6 | 3189 | 3241 | LOC105371430 | 1 | 53 | -94.86 | lmBtoBDcommon | No |
| HCG21:1 | 2878 | 2927 | LOC105371430 | 4 | 53 | -85.34 | lmDtoBDcommon | No |
| lnc-AL136218.1-1:2 | 411 | 463 | LOC105371430 | 1 | 53 | -97.26 | lmDtoBDcommon | No |
| lnc-AUH-3:1 | 1978 | 2025 | LOC105371430 | 228 | 275 | -50.17 | lmDtoBDcommon | No |
| lnc-HMGB2-8:1 | 2491 | 2541 | LOC105371430 | 3 | 53 | -99.91 | lmDtoBDcommon | No |
| lnc-MPHOSPH8-7:1 | 1085 | 1133 | LOC105371430 | 17 | 65 | -66.7 | lmDtoBDcommon | No |
| lnc-LRRFIP2-1:1 | 2037 | 2080 | LOC105376526 | 624 | 678 | -54.82 | lmBtoBDcommon | Yes |
| TCONS_00048674 | 591 | 642 | LOC105376526 | 816 | 862 | -59 | lmDtoBDcommon | Yes |
| lnc-ABCC6-2:1 | 707 | 755 | LOC105376526 | 2290 | 2337 | -52.44 | lmBtoBDcommon | No |
| lnc-AC091132.2-7:1 | 219 | 264 | LOC105376526 | 1256 | 1293 | -51.45 | lmBtoBDcommon | No |
| lnc-FAM160A1-8:1 | 226 | 272 | LOC105376526 | 2273 | 2317 | -54.33 | lmBtoBDcommon | No |
| lnc-RBAK-2:10 | 798 | 845 | LOC105376526 | 1216 | 1262 | -58.4 | lmBtoBDcommon | No |
| lnc-SIX5-1:1 | 312 | 354 | LOC105376526 | 1339 | 1379 | -50.28 | lmBtoBDcommon | No |
| TCONS_00052860 | 771 | 815 | LOC105376526 | 3589 | 3626 | -50.61 | lmBtoBDcommon | No |
| lnc-CDHR4-3:1 | 317 | 360 | LOC105376526 | 1308 | 1353 | -50.66 | lmDtoBDcommon | No |
| lnc-HTR2C-5:1 | 5024 | 5082 | LOC105376526 | 2339 | 2397 | -53.3 | lmDtoBDcommon | No |
| lnc-KRTAP10-12-3:1 | 89 | 147 | LOC105376526 | 2339 | 2397 | -56.6 | lmDtoBDcommon | No |
| lnc-AMZ2-5:1 | 598 | 641 | LOC390937 | 1224 | 1267 | -53.44 | lmBtoBDcommon | Yes |
| lnc-CGNL1-6:1 | 61 | 106 | LOC390937 | 950 | 994 | -52.91 | lmBtoBDcommon | Yes |
| lnc-DYDC1-1:1 | 461 | 502 | LOC390937 | 1691 | 1736 | -51.96 | lmBtoBDcommon | Yes |
| lnc-EID2B-1:1 | 112 | 151 | LOC390937 | 1127 | 1173 | -50.2 | lmBtoBDcommon | Yes |
| lnc-HIST1H2AI-1:3 | 499 | 545 | LOC390937 | 1238 | 1287 | -51.34 | lmBtoBDcommon | Yes |
| lnc-KIAA0226-4:1 | 139 | 185 | LOC390937 | 1238 | 1287 | -51.34 | lmBtoBDcommon | Yes |
| lnc-LRRFIP2-1:1 | 1837 | 1883 | LOC390937 | 482 | 526 | -52.55 | lmBtoBDcommon | Yes |
| lnc-MDK-4:2 | 46 | 93 | LOC390937 | 1564 | 1613 | -66.66 | lmBtoBDcommon | Yes |
| lnc-PGPEP1L-3:1 | 1035 | 1082 | LOC390937 | 1582 | 1625 | -57.2 | lmBtoBDcommon | Yes |
| lnc-RPRML-3:20 | 64 | 107 | LOC390937 | 946 | 992 | -55.43 | lmBtoBDcommon | Yes |
| lnc-RPUSD2-2:1 | 65 | 116 | LOC390937 | 1340 | 1386 | -55.8 | lmBtoBDcommon | Yes |
| lnc-VASH2-1:1 | 146 | 193 | LOC390937 | 1113 | 1162 | -51.56 | lmBtoBDcommon | Yes |
| TCONS_00048671 | 22 | 67 | LOC390937 | 935 | 984 | -55.26 | lmBtoBDcommon | Yes |
| TCONS_00052318 | 262 | 306 | LOC390937 | 1190 | 1232 | -52.89 | lmBtoBDcommon | Yes |
| TCONS_00052868 | 578 | 625 | LOC390937 | 1576 | 1623 | -71.83 | lmBtoBDcommon | Yes |
| lnc-FAM96A-1:1 | 52 | 99 | LOC390937 | 1372 | 1410 | -50.74 | lmDtoBDcommon | Yes |
| lnc-HIVEP3-1:1 | 1 | 45 | LOC390937 | 964 | 1007 | -52.47 | lmDtoBDcommon | Yes |
| CHKB-AS1:14 | 2555 | 2600 | LOC390937 | 1113 | 1162 | -53.31 | lmBtoBDcommon | No |
| DANT1:2 | 1597 | 1641 | LOC390937 | 56 | 98 | -53.67 | lmBtoBDcommon | No |
| FTX:1 | 71 | 115 | LOC390937 | 1153 | 1200 | -58.03 | lmBtoBDcommon | No |
| GAS6-AS2:1 | 178 | 224 | LOC390937 | 1238 | 1287 | -51.34 | lmBtoBDcommon | No |
| LINC00847:14 | 598 | 648 | LOC390937 | 834 | 885 | -56.75 | lmBtoBDcommon | No |
| LINC00887:5 | 2148 | 2195 | LOC390937 | 908 | 953 | -55.83 | lmBtoBDcommon | No |
| LINC00920:3 | 16 | 75 | LOC390937 | 1554 | 1605 | -55.1 | lmBtoBDcommon | No |
| LINC01299:2 | 1610 | 1649 | LOC390937 | 1587 | 1632 | -53.22 | lmBtoBDcommon | No |
| lnc-ABCC6-2:1 | 209 | 256 | LOC390937 | 920 | 965 | -55.78 | lmBtoBDcommon | No |
| lnc-ABHD2-4:1 | 450 | 493 | LOC390937 | 1501 | 1543 | -58.97 | lmBtoBDcommon | No |
| lnc-AC091132.2-7:1 | 216 | 260 | LOC390937 | 880 | 924 | -59.54 | lmBtoBDcommon | No |
| lnc-ACR-2:5 | 1 | 45 | LOC390937 | 980 | 1024 | -50.41 | lmBtoBDcommon | No |
| lnc-AMZ2-3:1 | 542 | 585 | LOC390937 | 1396 | 1440 | -56.87 | lmBtoBDcommon | No |
| lnc-ANKRD30B-7:2 | 529 | 568 | LOC390937 | 1585 | 1631 | -55.33 | lmBtoBDcommon | No |
| lnc-APBA2-5:8 | 57 | 103 | LOC390937 | 931 | 975 | -67.31 | lmBtoBDcommon | No |
| lnc-BCL9L-1:1 | 1859 | 1904 | LOC390937 | 1560 | 1601 | -54.03 | lmBtoBDcommon | No |
| lnc-C20orf24-3:1 | 434 | 484 | LOC390937 | 1435 | 1479 | -50.78 | lmBtoBDcommon | No |
| lnc-CARKD-4:1 | 27 | 74 | LOC390937 | 1583 | 1626 | -58.42 | lmBtoBDcommon | No |
| lnc-CLCN1-2:1 | 154 | 200 | LOC390937 | 1704 | 1753 | -64.15 | lmBtoBDcommon | No |
| lnc-CLIC3-2:1 | 138 | 180 | LOC390937 | 1414 | 1460 | -59.69 | lmBtoBDcommon | No |
| lnc-DDX52-3:1 | 833 | 879 | LOC390937 | 1206 | 1253 | -52.17 | lmBtoBDcommon | No |
| lnc-EXOC2-8:4 | 1603 | 1642 | LOC390937 | 1584 | 1626 | -53.38 | lmBtoBDcommon | No |
| lnc-FAM160A1-8:1 | 89 | 134 | LOC390937 | 1398 | 1446 | -50.05 | lmBtoBDcommon | No |
| lnc-FRYL-3:3 | 121 | 169 | LOC390937 | 1127 | 1176 | -60.34 | lmBtoBDcommon | No |
| lnc-HTRA2-1:1 | 22 | 71 | LOC390937 | 1432 | 1476 | -65.33 | lmBtoBDcommon | No |
| lnc-IFT52-1:2 | 2639 | 2684 | LOC390937 | 1400 | 1440 | -52.36 | lmBtoBDcommon | No |
| lnc-KIAA0355-5:1 | 561 | 608 | LOC390937 | 964 | 1010 | -54.79 | lmBtoBDcommon | No |
| lnc-KLF13-2:4 | 205 | 242 | LOC390937 | 1058 | 1102 | -56.16 | lmBtoBDcommon | No |
| lnc-MAPK6-4:2 | 349 | 394 | LOC390937 | 1217 | 1259 | -51.17 | lmBtoBDcommon | No |
| lnc-MOGAT2-3:1 | 115 | 162 | LOC390937 | 616 | 652 | -57.32 | lmBtoBDcommon | No |
| lnc-NT5C-3:3 | 178 | 221 | LOC390937 | 1554 | 1597 | -51.56 | lmBtoBDcommon | No |
| lnc-PAIP2-1:1 | 61 | 102 | LOC390937 | 1587 | 1636 | -54.33 | lmBtoBDcommon | No |
| lnc-PCBP3-3:9 | 311 | 354 | LOC390937 | 130 | 166 | -50.68 | lmBtoBDcommon | No |
| lnc-POTEF-8:2 | 2050 | 2095 | LOC390937 | 1225 | 1274 | -52.34 | lmBtoBDcommon | No |
| lnc-PRAGMIN.1-3:2 | 212 | 258 | LOC390937 | 947 | 994 | -62.6 | lmBtoBDcommon | No |
| lnc-PRDM11-9:2 | 507 | 553 | LOC390937 | 1591 | 1635 | -54.07 | lmBtoBDcommon | No |
| lnc-PTGER1-1:2 | 527 | 574 | LOC390937 | 864 | 912 | -59.87 | lmBtoBDcommon | No |
| lnc-PUF60-1:1 | 332 | 378 | LOC390937 | 985 | 1032 | -59.97 | lmBtoBDcommon | No |
| lnc-RABGEF1-1:1 | 1499 | 1548 | LOC390937 | 1558 | 1607 | -55.6 | lmBtoBDcommon | No |
| lnc-RAD52-2:5 | 739 | 786 | LOC390937 | 917 | 955 | -56.33 | lmBtoBDcommon | No |
| lnc-RBAK-2:10 | 534 | 590 | LOC390937 | 1553 | 1607 | -63.51 | lmBtoBDcommon | No |
| lnc-RPL23A-2:1 | 407 | 449 | LOC390937 | 987 | 1034 | -56.34 | lmBtoBDcommon | No |
| lnc-RUNDC3A-4:2 | 327 | 367 | LOC390937 | 261 | 309 | -51.4 | lmBtoBDcommon | No |
| lnc-SAMD8-1:1 | 2054 | 2101 | LOC390937 | 1113 | 1162 | -51.23 | lmBtoBDcommon | No |
| lnc-SEC11A-2:1 | 15 | 67 | LOC390937 | 1568 | 1615 | -67.49 | lmBtoBDcommon | No |
| lnc-SERPINB6-1:7 | 835 | 884 | LOC390937 | 1579 | 1625 | -59.2 | lmBtoBDcommon | No |
| lnc-SIX5-1:1 | 1034 | 1077 | LOC390937 | 951 | 995 | -65.75 | lmBtoBDcommon | No |
| lnc-TAOK3-1:11 | 2624 | 2674 | LOC390937 | 1239 | 1287 | -54.91 | lmBtoBDcommon | No |
| lnc-TOR3A-1:1 | 3797 | 3838 | LOC390937 | 836 | 880 | -51.9 | lmBtoBDcommon | No |
| lnc-TTR-3:1 | 43 | 88 | LOC390937 | 1421 | 1465 | -53.54 | lmBtoBDcommon | No |
| lnc-TYSND1-1:7 | 41 | 90 | LOC390937 | 971 | 1020 | -63.93 | lmBtoBDcommon | No |
| lnc-WDR73-7:2 | 144 | 190 | LOC390937 | 1573 | 1612 | -58.5 | lmBtoBDcommon | No |
| lnc-ZKSCAN3-1:1 | 145 | 189 | LOC390937 | 908 | 956 | -52.76 | lmBtoBDcommon | No |
| PART1:10 | 4653 | 4704 | LOC390937 | 499 | 545 | -51.64 | lmBtoBDcommon | No |
| PCF11-AS1:2 | 121 | 165 | LOC390937 | 1584 | 1629 | -52.84 | lmBtoBDcommon | No |
| PSMB8-AS1:1 | 412 | 453 | LOC390937 | 1585 | 1632 | -51.07 | lmBtoBDcommon | No |
| PSMB8-AS1:5 | 473 | 514 | LOC390937 | 1585 | 1632 | -51.07 | lmBtoBDcommon | No |
| SLC25A25-AS1:15 | 54 | 104 | LOC390937 | 1396 | 1444 | -50.63 | lmBtoBDcommon | No |
| ST3GAL5-AS1:4 | 1250 | 1295 | LOC390937 | 1563 | 1613 | -50.95 | lmBtoBDcommon | No |
| TCONS_00025364 | 867 | 914 | LOC390937 | 962 | 1006 | -50.97 | lmBtoBDcommon | No |
| TCONS_00049141 | 2481 | 2526 | LOC390937 | 1216 | 1255 | -53.57 | lmBtoBDcommon | No |
| TCONS_00052860 | 704 | 751 | LOC390937 | 1576 | 1623 | -71.83 | lmBtoBDcommon | No |
| ZNF528-AS1:3 | 697 | 745 | LOC390937 | 1223 | 1264 | -52.64 | lmBtoBDcommon | No |
| CCDC18-AS1:37 | 206 | 248 | LOC390937 | 1560 | 1607 | -52.62 | lmDtoBDcommon | No |
| LINC00944:13 | 502 | 551 | LOC390937 | 1006 | 1060 | -52.65 | lmDtoBDcommon | No |
| lnc-CDHR4-3:1 | 55 | 103 | LOC390937 | 1563 | 1605 | -55.19 | lmDtoBDcommon | No |
| lnc-HTR2C-5:1 | 5014 | 5063 | LOC390937 | 1095 | 1142 | -54.35 | lmDtoBDcommon | No |
| lnc-RP11-706O15.1.1-1:4 | 1485 | 1537 | LOC390937 | 981 | 1029 | -62.97 | lmDtoBDcommon | No |
| BZRAP1-AS1:19 | 1003 | 1052 | MATN2 | 5 | 51 | -56.46 | lmBtoBDcommon | No |
| BZRAP1-AS1:20 | 1003 | 1052 | MATN2 | 5 | 51 | -56.46 | lmBtoBDcommon | No |
| LINC00987:3 | 401 | 448 | MATN2 | 56 | 101 | -57.28 | lmBtoBDcommon | No |
| lnc-C12orf39-2:4 | 255 | 305 | MATN2 | 30 | 77 | -50.89 | lmBtoBDcommon | No |
| lnc-FSD1L-3:1 | 118 | 161 | MATN2 | 195 | 239 | -56.87 | lmBtoBDcommon | No |
| lnc-PSMB9-6:4 | 617 | 655 | MATN2 | 4 | 42 | -55.78 | lmBtoBDcommon | No |
| TCONS_00049092 | 349 | 390 | MATN2 | 145 | 188 | -60.14 | lmBtoBDcommon | No |
| TCONS_00049095 | 296 | 337 | MATN2 | 145 | 188 | -60.14 | lmBtoBDcommon | No |
| lnc-BRD1-7:1 | 12 | 61 | MATN2 | 184 | 228 | -50.87 | lmDtoBDcommon | No |
| lnc-EFNA4-1:6 | 414 | 458 | MATN2 | 190 | 234 | -52.18 | lmDtoBDcommon | No |
| lnc-RP11-1277H1.1.1-11:1 | 166 | 206 | MATN2 | 2424 | 2469 | -52.97 | lmDtoBDcommon | No |
| LINC01237:6 | 54 | 98 | MSH5 | 322 | 371 | -57.13 | lmBtoBDcommon | Yes |
| lnc-AMZ2-5:1 | 1725 | 1766 | MSH5 | 369 | 413 | -61.1 | lmBtoBDcommon | Yes |
| lnc-C12orf50-6:2 | 976 | 1015 | MSH5 | 369 | 413 | -51.74 | lmBtoBDcommon | Yes |
| lnc-CGNL1-6:1 | 204 | 250 | MSH5 | 1370 | 1412 | -50.01 | lmBtoBDcommon | Yes |
| lnc-GGCT-1:12 | 1075 | 1123 | MSH5 | 345 | 394 | -56.91 | lmBtoBDcommon | Yes |
| lnc-HIST1H2AI-1:3 | 658 | 696 | MSH5 | 370 | 413 | -50.29 | lmBtoBDcommon | Yes |
| lnc-MDK-4:2 | 315 | 359 | MSH5 | 340 | 390 | -59.8 | lmBtoBDcommon | Yes |
| lnc-PGPEP1L-3:1 | 2520 | 2567 | MSH5 | 344 | 385 | -53.45 | lmBtoBDcommon | Yes |
| lnc-PLEKHA7-2:1 | 2851 | 2900 | MSH5 | 365 | 413 | -56.07 | lmBtoBDcommon | Yes |
| lnc-RPUSD2-2:1 | 1696 | 1736 | MSH5 | 370 | 416 | -54.85 | lmBtoBDcommon | Yes |
| lnc-ZNF114-2:1 | 810 | 851 | MSH5 | 369 | 413 | -52.4 | lmBtoBDcommon | Yes |
| TCONS_00048671 | 6 | 48 | MSH5 | 301 | 347 | -50.46 | lmBtoBDcommon | Yes |
| TCONS_00048674 | 872 | 918 | MSH5 | 136 | 189 | -63.32 | lmBtoBDcommon | Yes |
| TCONS_00052318 | 162 | 209 | MSH5 | 509 | 557 | -57.18 | lmBtoBDcommon | Yes |
| TCONS_00052868 | 286 | 331 | MSH5 | 346 | 386 | -60.24 | lmBtoBDcommon | Yes |
| lnc-ZNF114-2:1 | 810 | 851 | MSH5 | 369 | 413 | -52.4 | lmDtoBDcommon | Yes |
| TCONS_00048671 | 6 | 48 | MSH5 | 301 | 347 | -50.46 | lmDtoBDcommon | Yes |
| GAS6-AS2:1 | 126 | 169 | MSH5 | 1849 | 1892 | -51.48 | lmBtoBDcommon | No |
| LBX2-AS1:2 | 1062 | 1107 | MSH5 | 346 | 391 | -57.84 | lmBtoBDcommon | No |
| LINC00654:4 | 4238 | 4288 | MSH5 | 358 | 409 | -58.06 | lmBtoBDcommon | No |
| LINC00847:14 | 157 | 197 | MSH5 | 362 | 401 | -53.59 | lmBtoBDcommon | No |
| LINC00887:5 | 1237 | 1275 | MSH5 | 370 | 413 | -58.23 | lmBtoBDcommon | No |
| LINC01299:2 | 1925 | 1973 | MSH5 | 2373 | 2419 | -52.14 | lmBtoBDcommon | No |
| LINC01426:3 | 95 | 142 | MSH5 | 2839 | 2891 | -56.69 | lmBtoBDcommon | No |
| lnc-ABCC6-2:1 | 236 | 284 | MSH5 | 321 | 369 | -55.86 | lmBtoBDcommon | No |
| lnc-ABHD2-4:1 | 464 | 502 | MSH5 | 1910 | 1955 | -53.28 | lmBtoBDcommon | No |
| lnc-AC091132.2-7:1 | 12 | 58 | MSH5 | 345 | 384 | -50.22 | lmBtoBDcommon | No |
| lnc-AMZ2-3:1 | 864 | 904 | MSH5 | 369 | 412 | -53.8 | lmBtoBDcommon | No |
| lnc-ANKRD30B-7:2 | 283 | 331 | MSH5 | 342 | 392 | -55.88 | lmBtoBDcommon | No |
| lnc-APBA2-5:8 | 104 | 154 | MSH5 | 324 | 371 | -56.07 | lmBtoBDcommon | No |
| lnc-C11orf94-1:1 | 172 | 222 | MSH5 | 303 | 351 | -50.66 | lmBtoBDcommon | No |
| lnc-C14orf79-3:1 | 1044 | 1091 | MSH5 | 362 | 404 | -58.62 | lmBtoBDcommon | No |
| lnc-C17orf109-3:1 | 24 | 70 | MSH5 | 351 | 397 | -52.1 | lmBtoBDcommon | No |
| lnc-C20orf24-3:1 | 374 | 417 | MSH5 | 339 | 390 | -54.43 | lmBtoBDcommon | No |
| lnc-C3orf62-2:1 | 67 | 107 | MSH5 | 369 | 412 | -54.97 | lmBtoBDcommon | No |
| lnc-CARKD-4:1 | 47 | 94 | MSH5 | 353 | 392 | -50.34 | lmBtoBDcommon | No |
| lnc-CEP170-9:2 | 3341 | 3388 | MSH5 | 386 | 423 | -61.05 | lmBtoBDcommon | No |
| lnc-CLCN1-2:1 | 62 | 100 | MSH5 | 2416 | 2461 | -51.3 | lmBtoBDcommon | No |
| lnc-CLIC3-2:1 | 151 | 195 | MSH5 | 341 | 382 | -58.15 | lmBtoBDcommon | No |
| lnc-COX19-2:2 | 1469 | 1513 | MSH5 | 368 | 413 | -52.97 | lmBtoBDcommon | No |
| lnc-CRYBA4-1:55 | 2315 | 2360 | MSH5 | 376 | 425 | -56.86 | lmBtoBDcommon | No |
| lnc-FAM160A1-8:1 | 120 | 164 | MSH5 | 360 | 402 | -54.03 | lmBtoBDcommon | No |
| lnc-FRYL-3:3 | 103 | 151 | MSH5 | 2482 | 2528 | -50.65 | lmBtoBDcommon | No |
| lnc-FTSJ1-2:1 | 470 | 515 | MSH5 | 855 | 901 | -52.87 | lmBtoBDcommon | No |
| lnc-GRID2-1:1 | 406 | 455 | MSH5 | 380 | 427 | -54.91 | lmBtoBDcommon | No |
| lnc-GTPBP1-1:1 | 119 | 166 | MSH5 | 319 | 365 | -59.08 | lmBtoBDcommon | No |
| lnc-HEATR2-3:1 | 1351 | 1397 | MSH5 | 344 | 387 | -56.36 | lmBtoBDcommon | No |
| lnc-HTRA2-1:1 | 511 | 559 | MSH5 | 1152 | 1200 | -58.52 | lmBtoBDcommon | No |
| lnc-IFT52-1:2 | 52 | 87 | MSH5 | 353 | 393 | -57.11 | lmBtoBDcommon | No |
| lnc-KIAA0355-5:1 | 560 | 599 | MSH5 | 370 | 412 | -51 | lmBtoBDcommon | No |
| lnc-KLHL5-2:1 | 3297 | 3338 | MSH5 | 368 | 412 | -53.83 | lmBtoBDcommon | No |
| lnc-LRRC56-3:4 | 618 | 667 | MSH5 | 4 | 48 | -50.95 | lmBtoBDcommon | No |
| lnc-MOGAT2-3:1 | 118 | 168 | MSH5 | 2370 | 2419 | -54.28 | lmBtoBDcommon | No |
| lnc-NT5C-3:3 | 171 | 211 | MSH5 | 344 | 387 | -59.66 | lmBtoBDcommon | No |
| lnc-OLFML3-5:1 | 925 | 970 | MSH5 | 2594 | 2635 | -51.33 | lmBtoBDcommon | No |
| lnc-PCBP3-3:9 | 314 | 358 | MSH5 | 1909 | 1958 | -60.7 | lmBtoBDcommon | No |
| lnc-PRAGMIN.1-3:2 | 55 | 100 | MSH5 | 379 | 422 | -55.91 | lmBtoBDcommon | No |
| lnc-PRDM11-9:2 | 121 | 167 | MSH5 | 345 | 389 | -54.79 | lmBtoBDcommon | No |
| lnc-PTGER1-1:2 | 129 | 172 | MSH5 | 354 | 398 | -52.65 | lmBtoBDcommon | No |
| lnc-PUF60-1:1 | 329 | 374 | MSH5 | 343 | 386 | -54.49 | lmBtoBDcommon | No |
| lnc-RABGEF1-1:1 | 527 | 573 | MSH5 | 855 | 902 | -54.98 | lmBtoBDcommon | No |
| lnc-RBAK-2:10 | 597 | 642 | MSH5 | 1366 | 1412 | -50.61 | lmBtoBDcommon | No |
| lnc-RBPMS2-1:2 | 214 | 250 | MSH5 | 1367 | 1408 | -55.61 | lmBtoBDcommon | No |
| lnc-RCN1-2:1 | 631 | 669 | MSH5 | 370 | 413 | -52.47 | lmBtoBDcommon | No |
| lnc-RUNDC3A-4:2 | 137 | 185 | MSH5 | 2462 | 2517 | -53.12 | lmBtoBDcommon | No |
| lnc-SAMD8-1:1 | 991 | 1038 | MSH5 | 338 | 377 | -58.98 | lmBtoBDcommon | No |
| lnc-SBDS-4:1 | 2098 | 2144 | MSH5 | 2594 | 2639 | -54.39 | lmBtoBDcommon | No |
| lnc-SEC11A-2:1 | 24 | 74 | MSH5 | 345 | 392 | -61.07 | lmBtoBDcommon | No |
| lnc-SIX5-1:1 | 358 | 407 | MSH5 | 1364 | 1413 | -64.11 | lmBtoBDcommon | No |
| lnc-SLC9A7-1:2 | 1386 | 1422 | MSH5 | 2804 | 2845 | -51.2 | lmBtoBDcommon | No |
| lnc-TAF13-2:1 | 1427 | 1473 | MSH5 | 1371 | 1413 | -57.19 | lmBtoBDcommon | No |
| lnc-TAOK3-1:11 | 706 | 747 | MSH5 | 2597 | 2636 | -53.72 | lmBtoBDcommon | No |
| lnc-TOR3A-1:1 | 381 | 424 | MSH5 | 2383 | 2430 | -50.58 | lmBtoBDcommon | No |
| lnc-TYSND1-1:7 | 936 | 978 | MSH5 | 358 | 406 | -55.33 | lmBtoBDcommon | No |
| lnc-WDR73-7:2 | 16 | 61 | MSH5 | 335 | 377 | -54.87 | lmBtoBDcommon | No |
| lnc-ZCCHC9-2:5 | 381 | 423 | MSH5 | 2806 | 2853 | -52.63 | lmBtoBDcommon | No |
| PART1:10 | 875 | 906 | MSH5 | 386 | 423 | -52.49 | lmBtoBDcommon | No |
| SMIM2-AS1:11 | 1606 | 1652 | MSH5 | 855 | 902 | -55.98 | lmBtoBDcommon | No |
| ST3GAL5-AS1:4 | 1527 | 1571 | MSH5 | 337 | 385 | -52.9 | lmBtoBDcommon | No |
| TCONS_00052860 | 412 | 457 | MSH5 | 346 | 386 | -60.24 | lmBtoBDcommon | No |
| THUMPD3-AS1:5 | 972 | 1016 | MSH5 | 2845 | 2890 | -50.87 | lmBtoBDcommon | No |
| ZNF528-AS1:3 | 738 | 778 | MSH5 | 349 | 394 | -56.39 | lmBtoBDcommon | No |
| LINC00311:2 | 24 | 65 | MSH5 | 353 | 393 | -57.05 | lmDtoBDcommon | No |
| lnc-AL136218.1-1:2 | 426 | 467 | MSH5 | 369 | 413 | -52.4 | lmDtoBDcommon | No |
| lnc-AUH-3:1 | 80 | 125 | MSH5 | 355 | 399 | -51.27 | lmDtoBDcommon | No |
| lnc-BAG1-2:12 | 49 | 87 | MSH5 | 370 | 413 | -52.77 | lmDtoBDcommon | No |
| lnc-C16orf5-1:1 | 1342 | 1388 | MSH5 | 855 | 902 | -52.37 | lmDtoBDcommon | No |
| lnc-S1PR1-2:2 | 8388 | 8434 | MSH5 | 369 | 421 | -62.86 | lmDtoBDcommon | No |
| lnc-SFTA2-14:1 | 2641 | 2683 | MSH5 | 2846 | 2885 | -52.45 | lmDtoBDcommon | No |
| lnc-WDR19-1:1 | 2060 | 2099 | MSH5 | 369 | 412 | -52.41 | lmDtoBDcommon | No |
| lnc-RPRML-3:20 | 1457 | 1503 | OR1J2 | 644 | 688 | -51.71 | lmBtoBDcommon | Yes |
| lnc-THAP10-1:1 | 486 | 532 | OR1J2 | 783 | 831 | -52.59 | lmBtoBDcommon | Yes |
| TCONS_00052318 | 271 | 318 | OR1J2 | 735 | 782 | -53.36 | lmBtoBDcommon | Yes |
| TCONS_00052868 | 564 | 609 | OR1J2 | 174 | 220 | -51.7 | lmBtoBDcommon | Yes |
| LINC01299:2 | 399 | 444 | OR1J2 | 783 | 829 | -51.6 | lmBtoBDcommon | No |
| lnc-ABCC6-2:1 | 183 | 229 | OR1J2 | 727 | 775 | -51.7 | lmBtoBDcommon | No |
| lnc-CARKD-4:1 | 390 | 431 | OR1J2 | 812 | 855 | -52.84 | lmBtoBDcommon | No |
| lnc-FRYL-3:3 | 119 | 159 | OR1J2 | 821 | 861 | -51.91 | lmBtoBDcommon | No |
| lnc-FXYD4-4:2 | 67 | 114 | OR1J2 | 2377 | 2423 | -50.1 | lmBtoBDcommon | No |
| lnc-HEATR2-3:1 | 1428 | 1471 | OR1J2 | 809 | 852 | -55.23 | lmBtoBDcommon | No |
| lnc-LIPI-9:1 | 467 | 513 | OR1J2 | 783 | 830 | -54.41 | lmBtoBDcommon | No |
| lnc-MOGAT2-3:1 | 120 | 159 | OR1J2 | 827 | 862 | -57.26 | lmBtoBDcommon | No |
| lnc-ROPN1L-3:1 | 1898 | 1944 | OR1J2 | 828 | 868 | -52.6 | lmBtoBDcommon | No |
| lnc-SIX5-1:1 | 597 | 643 | OR1J2 | 817 | 862 | -53.01 | lmBtoBDcommon | No |
| lnc-TYSND1-1:7 | 1205 | 1251 | OR1J2 | 783 | 830 | -55.72 | lmBtoBDcommon | No |
| lnc-WDR73-7:2 | 72 | 120 | OR1J2 | 896 | 948 | -51.59 | lmBtoBDcommon | No |
| lnc-ZNF827-11:1 | 69 | 119 | OR1J2 | 1589 | 1627 | -50.43 | lmBtoBDcommon | No |
| PART1:10 | 3851 | 3891 | OR1J2 | 829 | 874 | -55.39 | lmBtoBDcommon | No |
| TCONS_00052860 | 1020 | 1062 | OR1J2 | 772 | 818 | -53.52 | lmBtoBDcommon | No |
| TCONS_00049092 | 103 | 150 | SLC4A10 | 2703 | 2749 | -50.1 | lmBtoBDcommon | No |
| TCONS_00049095 | 101 | 148 | SLC4A10 | 2703 | 2749 | -50.1 | lmBtoBDcommon | No |
| LINC01237:6 | 3 | 43 | ZFP57 | 16 | 58 | -51.15 | lmBtoBDcommon | Yes |
| lnc-AMZ2-5:1 | 369 | 413 | ZFP57 | 77 | 118 | -53.04 | lmBtoBDcommon | Yes |
| lnc-HES5-1:7 | 2080 | 2119 | ZFP57 | 301 | 342 | -54.94 | lmBtoBDcommon | Yes |
| lnc-PLEKHG2-1:1 | 252 | 305 | ZFP57 | 1062 | 1110 | -54.02 | lmBtoBDcommon | Yes |
| lnc-WDR7-6:2 | 325 | 374 | ZFP57 | 572 | 620 | -50.41 | lmBtoBDcommon | Yes |
| TCL6:19 | 2719 | 2763 | ZFP57 | 156 | 201 | -50.73 | lmBtoBDcommon | Yes |
| TCONS_00001685 | 2047 | 2093 | ZFP57 | 135 | 186 | -51.09 | lmBtoBDcommon | Yes |
| TCONS_00052862 | 630 | 675 | ZFP57 | 357 | 404 | -60.28 | lmBtoBDcommon | Yes |
| FAM201A:1 | 118 | 163 | ZFP57 | 1119 | 1160 | -50.35 | lmBtoBDcommon | No |
| LBX2-AS1:2 | 1290 | 1338 | ZFP57 | 690 | 737 | -52.04 | lmBtoBDcommon | No |
| LINC00654:4 | 5039 | 5091 | ZFP57 | 1308 | 1355 | -53.26 | lmBtoBDcommon | No |
| lnc-AL669831.1-3:42 | 2169 | 2220 | ZFP57 | 26 | 76 | -51.2 | lmBtoBDcommon | No |
| lnc-C10orf90-2:2 | 333 | 377 | ZFP57 | 86 | 132 | -50.42 | lmBtoBDcommon | No |
| lnc-C11orf94-1:1 | 191 | 238 | ZFP57 | 91 | 132 | -50.95 | lmBtoBDcommon | No |
| lnc-C14orf79-3:1 | 1059 | 1108 | ZFP57 | 687 | 738 | -50.37 | lmBtoBDcommon | No |
| lnc-C15orf39-1:1 | 1292 | 1339 | ZFP57 | 131 | 176 | -57.85 | lmBtoBDcommon | No |
| lnc-C17orf109-3:1 | 34 | 77 | ZFP57 | 894 | 941 | -54.73 | lmBtoBDcommon | No |
| lnc-C1orf138-1:1 | 452 | 500 | ZFP57 | 1609 | 1654 | -54.08 | lmBtoBDcommon | No |
| lnc-CAMK1-1:2 | 430 | 471 | ZFP57 | 1634 | 1676 | -54.9 | lmBtoBDcommon | No |
| lnc-CEP170-9:2 | 3223 | 3268 | ZFP57 | 1121 | 1159 | -50.6 | lmBtoBDcommon | No |
| lnc-CLIC3-2:1 | 308 | 353 | ZFP57 | 6 | 47 | -53.64 | lmBtoBDcommon | No |
| lnc-CRYBA4-1:55 | 1438 | 1485 | ZFP57 | 131 | 176 | -55.01 | lmBtoBDcommon | No |
| lnc-DOC2B-3:2 | 1397 | 1443 | ZFP57 | 698 | 743 | -52.42 | lmBtoBDcommon | No |
| lnc-FPR2-1:10 | 563 | 606 | ZFP57 | 297 | 335 | -55.38 | lmBtoBDcommon | No |
| lnc-GPR152-1:7 | 141 | 191 | ZFP57 | 144 | 194 | -52.24 | lmBtoBDcommon | No |
| lnc-GRID2-1:1 | 404 | 451 | ZFP57 | 1716 | 1769 | -55.48 | lmBtoBDcommon | No |
| lnc-HEATR2-3:1 | 1448 | 1484 | ZFP57 | 920 | 964 | -57.71 | lmBtoBDcommon | No |
| lnc-LONRF2-3:1 | 179 | 218 | ZFP57 | 152 | 195 | -50.14 | lmBtoBDcommon | No |
| lnc-LRCH4-2:1 | 146 | 183 | ZFP57 | 20 | 68 | -54.88 | lmBtoBDcommon | No |
| lnc-MTA2-4:1 | 195 | 242 | ZFP57 | 170 | 216 | -50.2 | lmBtoBDcommon | No |
| lnc-POFUT2-5:1 | 2203 | 2251 | ZFP57 | 20 | 57 | -53.08 | lmBtoBDcommon | No |
| lnc-PRAGMIN.1-3:2 | 210 | 255 | ZFP57 | 159 | 208 | -55.8 | lmBtoBDcommon | No |
| lnc-RAP1GAP2-3:1 | 782 | 825 | ZFP57 | 368 | 413 | -57.22 | lmBtoBDcommon | No |
| lnc-SLC26A11-1:1 | 534 | 581 | ZFP57 | 128 | 163 | -52.59 | lmBtoBDcommon | No |
| lnc-TNFRSF1A-3:1 | 147 | 191 | ZFP57 | 1432 | 1477 | -50.49 | lmBtoBDcommon | No |
| lnc-TNNT1-1:1 | 1583 | 1634 | ZFP57 | 371 | 423 | -54.85 | lmBtoBDcommon | No |
| lnc-TTR-3:1 | 153 | 195 | ZFP57 | 4 | 45 | -50.04 | lmBtoBDcommon | No |
| lnc-TYSND1-1:7 | 1590 | 1633 | ZFP57 | 149 | 183 | -53.76 | lmBtoBDcommon | No |
| lnc-VTI1A-1:3 | 1197 | 1239 | ZFP57 | 158 | 203 | -58.02 | lmBtoBDcommon | No |
| lnc-ZBED5-1:2 | 671 | 717 | ZFP57 | 1941 | 1984 | -52.98 | lmBtoBDcommon | No |
| lnc-ZNF587-1:1 | 171 | 217 | ZFP57 | 688 | 732 | -54.3 | lmBtoBDcommon | No |
| SNAI3-AS1:16 | 1208 | 1255 | ZFP57 | 24 | 73 | -61.07 | lmBtoBDcommon | No |
| TCONS_00000037 | 3086 | 3134 | ZFP57 | 674 | 711 | -56.43 | lmBtoBDcommon | No |
| TCONS_00044060 | 446 | 492 | ZFP57 | 1631 | 1683 | -52.04 | lmBtoBDcommon | No |
| TCONS_00052860 | 690 | 735 | ZFP57 | 145 | 192 | -60.28 | lmBtoBDcommon | No |
| TRAF3IP2-AS1:24 | 189 | 228 | ZFP57 | 1895 | 1944 | -52.38 | lmBtoBDcommon | No |
| lnc-MRPS5-12:1 | 2100 | 2147 | ZFP57 | 1292 | 1333 | -52.56 | lmDtoBDcommon | No |
| SNHG11:14 | 190 | 236 | ZFP57 | 10 | 51 | -50.61 | lmDtoBDcommon | No |

^a^ The possible lncRNA-mRNA interactions were evaluated by the software “RIsearch” v2.0 , setting parameters as hybridization sites length >20 nt, binding free energy < -50, under the prerequisite of coexpression (Pearson’s correlation coefficient >0.8) of a lncRNA and a gene in each case population. lmBtoBDcommon, means the interaction was observed in QDC population; lmDtoBDcommon, denotes the interaction was observed in PQDS population.

^b^ “Yes” or “No” denotes whether the corresponding lncRNA of a possible binding pair belongs to the common lncRNAs set.

**Table S14** The possible lncRNA-mRNA binding among the common differential lncRNAs and genes.

| **Bound mRNAs ^a^** | **lncRNAs count** | **List for *trans*-acting lncRNAs ^a^** |
| --- | --- | --- |
| **ADAMTSL5** | 58 | CDC42-IT1:1; DLX6-AS1:13; GAS5:31; GAS5:41; LINC00152:10; LINC01237:6; lnc-AC233264.2-1:1; lnc-AKAP11-1:2; lnc-AMZ2-5:1; lnc-ARHGAP27-2:1; lnc-ARL1-3:1; **lnc-C12orf50-6:2**; lnc-CA10-1:1; lnc-CACYBP-2:1; lnc-CELF6-1:5; **lnc-CEP170-11:1**; lnc-CSNK1D-1:1; lnc-EGLN1-1:3; lnc-FAM32A-2:1; lnc-FBXL2-4:1; lnc-GGCT-1:12; lnc-HES5-1:7; lnc-HIST1H2AI-1:3; lnc-HIVEP3-1:1; lnc-KIAA0226-4:1; lnc-KLB-2:1; **lnc-LRRFIP2-1:1**; lnc-MDK-4:2; lnc-NPBWR1-1:2; **lnc-PGPEP1L-3:1**; **lnc-PLEKHA7-2:1**; lnc-PLEKHG2-1:1; lnc-PRPF4B-4:4; lnc-RAB23-17:1; lnc-RP11-108K14.4.1-1:2; lnc-RP11-706O15.1.1-2:8; lnc-RPRML-3:20; lnc-SWI5-1:1; lnc-THAP10-1:1; lnc-TWIST1-1:3; **lnc-VASH2-1:1**; lnc-ZFAND4-1:1; lnc-ZNF114-2:1; lnc-ZNF212-2:2; lnc-ZNF91-4:2; PAN3-AS1:5; PITPNA-AS1:2; PSMD5-AS1:3; TCL6:19; TCONS_00048609; TCONS_00048671; TCONS_00048674; TCONS_00048675; TCONS_00049093; TCONS_00049110; TCONS_00052318; TCONS_00052862; TCONS_00052867; |
| **COL26A1** | 48 | CDC42-IT1:1; DLX6-AS1:13; LINC00299:3; LINC01237:6; lnc-AC233264.2-1:1; lnc-AKAP11-1:2; lnc-AMZ2-5:1; lnc-ARHGAP27-2:1; lnc-ARL1-3:1; lnc-C12orf50-6:2; lnc-CACYBP-2:1; lnc-CELF6-1:5; lnc-CSNK1D-1:1; lnc-FAM32A-2:1; lnc-FAM96A-1:1; lnc-GGCT-1:12; lnc-HES5-1:7; lnc-HIST1H2AI-1:3; lnc-HIVEP3-1:1; lnc-HYOU1-1:3; lnc-KIAA0226-4:1; **lnc-LRRFIP2-1:1**; lnc-MDK-4:2; lnc-NPBWR1-1:2; lnc-PGPEP1L-3:1; lnc-PLEKHA7-2:1; lnc-PLEKHG2-1:1; lnc-RNF19A-8:4; lnc-RP11-108K14.4.1-1:2; lnc-RPL10L-2:1; **lnc-RPRML-3:20**; lnc-WDR7-6:2; lnc-ZFAND4-1:1; lnc-ZNF114-2:1; lnc-ZNF212-2:2; PAN3-AS1:5; PITPNA-AS1:2; PSMD5-AS1:3; TCL6:19; TCONS_00048609; TCONS_00048671; TCONS_00048674; TCONS_00048675; TCONS_00049093; TCONS_00049110; TCONS_00052318; TCONS_00052862; TCONS_00052867; |
| **COL27A1** | 57 | CDC42-IT1:1; DLX6-AS1:13; LINC00152:10; LINC00299:3; LINC01237:6; lnc-AC103810.1-1:2; lnc-AC233264.2-1:1; lnc-AMZ2-5:1; lnc-ARAF-1:1; lnc-ARHGAP27-2:1; lnc-ARL1-3:1; lnc-C12orf50-6:2; lnc-CELF6-1:5; lnc-CEP170-11:1; lnc-CSNK1D-1:1; **lnc-EGLN1-1:3**; lnc-FAM32A-2:1; lnc-FAM96A-1:1; lnc-FBXL2-4:1; lnc-GGCT-1:12; lnc-HES5-1:7; lnc-HIST1H2AI-1:3; lnc-HIVEP3-1:1; lnc-IGFBP7-1:2; lnc-KIAA0226-4:1; lnc-LRRFIP2-1:1; lnc-MDK-4:2; lnc-MPPE1-5:1; lnc-NPBWR1-1:2; lnc-PGPEP1L-3:1; lnc-PLEKHA7-2:1; lnc-PLEKHG2-1:1; lnc-PRPF4B-4:4; lnc-RP11-108K14.4.1-1:2; lnc-RP11-706O15.1.1-2:8; lnc-RPL10L-2:1; lnc-RPRML-3:20; lnc-THAP10-1:1; lnc-VASH2-1:1; lnc-WDR7-6:2; lnc-ZFAND4-1:1; lnc-ZMAT5-4:3; lnc-ZNF212-2:2; lnc-ZNF91-4:2; PAN3-AS1:5; PITPNA-AS1:2; PSMD5-AS1:3; TCL6:19; TCONS_00048609; TCONS_00048671; TCONS_00048674; TCONS_00048675; TCONS_00049093; TCONS_00049110; TCONS_00052318; TCONS_00052862; TCONS_00052867; |
| CORIN | 8 | lnc-ARHGAP27-2:1; lnc-CSNK1D-1:1; lnc-HES5-1:7; lnc-PGPEP1L-3:1; TCONS_00048674; TCONS_00048675; TCONS_00049093; TCONS_00049110; |
| **LOC105371430** | 37 | CDC42-IT1:1; DLX6-AS1:13; lnc-AC103810.1-1:2; lnc-AKAP11-1:2; **lnc-AMZ2-5:1**; lnc-ARHGAP27-2:1; lnc-ARL1-3:1; lnc-C12orf50-6:2; lnc-CACYBP-2:1; lnc-CEP170-11:1; lnc-CSNK1D-1:1; lnc-EGLN1-1:3; lnc-GGCT-1:12; lnc-HES5-1:7; lnc-HIST1H2AI-1:3; lnc-KIAA0226-4:1; lnc-LRRFIP2-1:1; lnc-NPBWR1-1:2; lnc-PGPEP1L-3:1; lnc-PLEKHA7-2:1; lnc-PLEKHG2-1:1; lnc-PRPF4B-4:4; **lnc-RP11-706O15.1.1-2:8**; lnc-RPRML-3:20; lnc-SWI5-1:1; lnc-THAP10-1:1; lnc-TWIST1-1:3; lnc-VASH2-1:1; lnc-ZNF114-2:1; lnc-ZNF212-2:2; lnc-ZNF91-4:2; TCL6:19; TCONS_00048609; TCONS_00048674; TCONS_00048675; TCONS_00052862; TCONS_00052867; |
| LOC105376526 | 5 | lnc-LRRFIP2-1:1; lnc-PLEKHA7-2:1; lnc-TWIST1-1:3; TCONS_00048674; TCONS_00048675; |
| LOC390937 | 39 | lnc-AC233264.2-1:1; lnc-AMZ2-5:1; lnc-ARHGAP27-2:1; lnc-CELF6-1:5; lnc-CSNK1D-1:1; lnc-DYDC1-1:1; lnc-FAM32A-2:1; lnc-FAM96A-1:1; lnc-FBXL2-4:1; lnc-HES5-1:7; lnc-HIST1H2AI-1:3; lnc-HIVEP3-1:1; lnc-KIAA0226-4:1; lnc-KLB-2:1; lnc-LRRFIP2-1:1; lnc-MDK-4:2; lnc-NPBWR1-1:2; lnc-PGPEP1L-3:1; lnc-PLEKHA7-2:1; lnc-PLEKHG2-1:1; lnc-RP11-108K14.4.1-1:2; lnc-RPL10L-2:1; lnc-RPRML-3:20; lnc-VASH2-1:1; lnc-ZFAND4-1:1; lnc-ZNF212-2:2; PAN3-AS1:5; PITPNA-AS1:2; PSMD5-AS1:3; TCL6:19; TCONS_00048609; TCONS_00048671; TCONS_00048674; TCONS_00048675; TCONS_00049093; TCONS_00049110; TCONS_00052318; TCONS_00052862; TCONS_00052867; |
| MATN2 | 21 | DLX6-AS1:13; lnc-AMZ2-5:1; lnc-CELF6-1:5; lnc-HES5-1:7; lnc-HIST1H2AI-1:3; lnc-HIVEP3-1:1; lnc-KIAA0226-4:1; lnc-NPBWR1-1:2; lnc-PLEKHG2-1:1; lnc-RPL10L-2:1; lnc-ZFAND4-1:1; PAN3-AS1:5; TCONS_00048609; TCONS_00048671; TCONS_00048674; TCONS_00048675; TCONS_00049093; TCONS_00049110; TCONS_00052318; TCONS_00052862; TCONS_00052867; |
| **MSH5** | 27 | LINC01237:6; lnc-AMZ2-5:1; lnc-C12orf50-6:2; lnc-CSNK1D-1:1; lnc-GGCT-1:12; lnc-HES5-1:7; lnc-HIST1H2AI-1:3; lnc-HIVEP3-1:1; lnc-MDK-4:2; lnc-PGPEP1L-3:1; lnc-PLEKHA7-2:1; lnc-PLEKHG2-1:1; lnc-ZFAND4-1:1; **lnc-ZNF114-2:1**; lnc-ZNF212-2:2; lnc-ZNF91-4:2; PAN3-AS1:5; TCL6:19; TCONS_00048609; **TCONS_00048671**; TCONS_00048674; TCONS_00048675; TCONS_00049093; TCONS_00049110; TCONS_00052318; TCONS_00052862; TCONS_00052867; |
| SLC4A10 | 6 | lnc-HES5-1:7; TCONS_00048609; TCONS_00048674; TCONS_00048675; TCONS_00049093; TCONS_00049110; |
| ZFP57 | 17 | LINC01237:6; lnc-AMZ2-5:1; lnc-FAM32A-2:1; lnc-HES5-1:7; lnc-LRRFIP2-1:1; lnc-PLEKHG2-1:1; lnc-RP11-108K14.4.1-1:2; lnc-RPL10L-2:1; lnc-WDR7-6:2; TCL6:19; TCONS_00048609; TCONS_00048674; TCONS_00048675; TCONS_00049093; TCONS_00049110; TCONS_00052862; TCONS_00052867; |

^a^ “TCONS_” prefixed lncRNAs denote the novel lncRNAs discovered in this work. The possible lncRNA-mRNA interactions were evaluated by the software “RIsearch” v2.0 , setting parameters as hybridization sites length >20 nt, binding free energy < -50.

**Table S15** The lncRNA-protein interactions between the common differential lncRNAs and genes-coded proteins

| **Bound proteins ^a^** | **lncRNAs count** | **lncRNAs ^a^** | **Binding probability control parameters ^b^** | |
| --- | --- | --- | --- | --- |
|  |  |  | **RF** | **SVM** |
| **ADAMTSL5** | 6 | lnc-C12orf50-6:2 | 0.7 | 0.872 |
|  |  | lnc-EID2B-1:1 | 0.7 | 0.904 |
|  |  | lnc-LRRFIP2-1:1 | 0.75 | 0.928 |
|  |  | lnc-PGPEP1L-3:1 | 0.8 | 0.858 |
|  |  | lnc-PLEKHA7-2:1 | 0.75 | 0.738 |
|  |  | lnc-VASH2-1:1 | 0.75 | 0.849 |
| **COL26A1** | 3 | lnc-LRRFIP2-1:1 | 0.75 | 0.973 |
|  |  | lnc-RPRML-3:20 | 0.8 | 0.919 |
|  |  | lnc-VASH2-1:1 | 0.7 | 0.931 |
| **COL27A1** | 1 | EDNRB-AS1:2 | 0.85 | 0.984 |
| **LOC390937** | 2 | lnc-CEP170-11:1 | 0.8 | 0.981 |
|  |  | lnc-ZNF114-2:1 | 0.95 | 0.978 |
| **MATN2** | 2 | lnc-RAB23-17:1 | 0.8 | 0.824 |
|  |  | lnc-BTK-1:2 | 0.7 | 0.879 |
| **MSH5** | 1 | lnc-ZNF114-2:1 | 0.8 | 0.982 |

^a^ The possible lnRNA-protein interactions were evaluated by the RPISeq tool , under the prerequisite of coexpression (Pearson’s correlation coefficient >0.8) of a lncRNA and a gene in both case populations.

^b^ The binding probability control parameters were used, SVM (support vector machine) >0.5 and RF (random forest) >0.5. So, the predictions with probabilities >0.5 were considered “positive”, indicating that the corresponding lncRNA and protein are likely to interact with each other.

**Table S16** The 17 biological process (GO) terms enriched with the common targets and the predicted functional partners.

| **Term ID** | **Term description** | **Matched ^a^** | **Total ^b^** | **FDR** | **Matched proteins (labels) ^c^** |
| --- | --- | --- | --- | --- | --- |
| GO:0007131 | reciprocal meiotic recombination | 5 | 48 | 8.14E-08 | MLH1, MLH3, MSH4, MSH5, RAD51 |
| GO:0070192 | chromosome organization involved in meiotic cell cycle | 5 | 67 | 1.31E-07 | MLH1, MLH3, MSH4, MSH5, RAD51 |
| GO:0006298 | mismatch repair | 4 | 26 | 4.26E-07 | MLH1, MLH3, MSH4, MSH5 |
| GO:0007129 | synapsis | 4 | 46 | 2.03E-06 | MLH1, MLH3, MSH4, MSH5 |
| GO:0006259 | DNA metabolic process | 6 | 773 | 0.00018 | MLH1, MLH3, MSH4, MSH5, RAD51, ZFP57 |
| GO:0003433 | chondrocyte development involved in endochondral bone morphogenesis | 2 | 6 | 0.00033 | COL27A1, SERPINH1 |
| GO:0006281 | DNA repair | 5 | 491 | 0.00033 | MLH1, MLH3, MSH4, MSH5, RAD51 |
| GO:0051026 | chiasma assembly | 2 | 8 | 0.00049 | MSH4, MSH5 |
| GO:0007292 | female gamete generation | 3 | 126 | 0.0018 | MLH1, MLH3, MSH4 |
| GO:0022414 | reproductive process | 6 | 1350 | 0.0025 | CORIN, MLH1, MLH3, MSH4, MSH5, RAD51 |
| GO:0007143 | female meiotic nuclear division | 2 | 28 | 0.0032 | MLH1, MLH3 |
| GO:0033554 | cellular response to stress | 6 | 1553 | 0.0046 | MATN2, MLH1, MLH3, MSH4, MSH5, RAD51 |
| GO:0007140 | male meiotic nuclear division | 2 | 46 | 0.0073 | MLH1, MLH3 |
| GO:0000018 | regulation of DNA recombination | 2 | 76 | 0.0172 | MLH1, RAD51 |
| GO:0048468 | cell development | 5 | 1493 | 0.0274 | COL27A1, MATN2, MLH1, SERPINH1, SLC4A10 |
| GO:0006950 | response to stress | 7 | 3267 | 0.0327 | MATN2, MLH1, MLH3, MSH4, MSH5, RAD51, SERPINH1 |
| GO:0044703 | multi-organism reproductive process | 4 | 923 | 0.0332 | CORIN, MLH1, MLH3, MSH4 |

**Note**: FDR, false discovery rate.

^a^ The number of proteins matched with the corresponding GO term.

^b^ Total number of proteins belong to the corresponding GO term.

^c^ List of proteins matched with the corresponding GO term. Blue font indicates the common target genes.

**Table S17** The 11 cellular component (GO) terms enriched with the common targets and the predicted functional partners.

| **Term ID** | **Term description** | **Matched ^a^** | **Total ^b^** | **FDR** | **Matched proteins (labels) ^c^** |
| --- | --- | --- | --- | --- | --- |
| GO:0000795 | synaptonemal complex | 5 | 41 | 7.91E-09 | MLH1, MLH3, MSH4, MSH5, RAD51 |
| GO:0005712 | chiasma | 2 | 2 | 5.32E-05 | MLH1, MLH3 |
| GO:0005713 | recombination nodule | 2 | 2 | 5.32E-05 | MLH1, MSH4 |
| GO:0032300 | mismatch repair complex | 2 | 7 | 0.00019 | MLH1, MLH3 |
| GO:0001673 | male germ cell nucleus | 2 | 15 | 0.0006 | MLH1, MLH3 |
| GO:0044420 | extracellular matrix component | 2 | 59 | 0.0053 | ADAMTSL5, COL27A1 |
| GO:0031012 | extracellular matrix | 3 | 283 | 0.0054 | ADAMTSL5, COL27A1, MATN2 |
| GO:0005788 | endoplasmic reticulum lumen | 3 | 299 | 0.0059 | COL26A1, COL27A1, SERPINH1 |
| GO:0005581 | collagen trimer | 2 | 88 | 0.0092 | COL26A1, COL27A1 |
| GO:0070013 | intracellular organelle lumen | 9 | 5162 | 0.0092 | COL26A1, COL27A1, CORIN, MLH1, MLH3, MSH4, MSH5, RAD51, SERPINH1 |
| GO:0062023 | collagen-containing extracellular matrix | 2 | 144 | 0.0202 | COL27A1, MATN2 |

**Note**: FDR, false discovery rate.

^a^ The number of proteins matched with the corresponding GO term.

^b^ Total number of proteins belong to the corresponding GO term.

^c^ List of proteins matched with the corresponding GO term. Blue font indicates the common target genes.

**Table S18** The 11 molecular function (GO) terms enriched with the common targets and the predicted functional partners.

| **Term ID** | **Term description** | **Matched ^a^** | **Total ^b^** | **FDR** | **Matched proteins (labels) ^c^** |
| --- | --- | --- | --- | --- | --- |
| GO:0030983 | mismatched DNA binding | 4 | 12 | 2.10E-08 | MLH1, MLH3, MSH4, MSH5 |
| GO:0016887 | ATPase activity | 5 | 392 | 0.00019 | MLH1, MLH3, MSH4, MSH5, RAD51 |
| GO:0008094 | DNA-dependent ATPase activity | 3 | 66 | 0.00039 | MSH4, MSH5, RAD51 |
| GO:0003690 | double-stranded DNA binding | 5 | 828 | 0.0025 | MLH1, MLH3, MSH4, MSH5, RAD51 |
| GO:0097367 | carbohydrate derivative binding | 6 | 2163 | 0.0156 | ADAMTSL5, MLH1, MLH3, MSH4, MSH5, RAD51 |
| GO:0003697 | single-stranded DNA binding | 2 | 99 | 0.0163 | MLH3, RAD51 |
| GO:0005524 | ATP binding | 5 | 1462 | 0.0163 | MLH1, MLH3, MSH4, MSH5, RAD51 |
| GO:0003677 | DNA binding | 6 | 2457 | 0.0199 | MLH1, MLH3, MSH4, MSH5, RAD51, ZFP57 |
| GO:0016787 | hydrolase activity | 6 | 2448 | 0.0199 | CORIN, MLH1, MLH3, MSH4, MSH5, RAD51 |
| GO:0043167 | ion binding | 9 | 6066 | 0.0257 | ADAMTSL5, COL27A1, MATN2, MLH1, MLH3, MSH4, MSH5, RAD51, ZFP57 |
| GO:0043168 | anion binding | 6 | 2696 | 0.0257 | ADAMTSL5, MLH1, MLH3, MSH4, MSH5, RAD51 |

**Note**: FDR, false discovery rate.

^a^ The number of proteins matched with the corresponding GO term.

^b^ Total number of proteins belong to the corresponding GO term.

^c^ List of proteins matched with the corresponding GO term. Blue font indicates the common target genes.

**Table S19** **Reactome pathways significantly enriched with the common targets and the predicted functional partners.**

| **Term ID** | **Term description** | **Matched ^a^** | **Total ^b^** | **FDR** | **Matched proteins (labels) ^c^** |
| --- | --- | --- | --- | --- | --- |
| HSA-912446 | Meiotic recombination | 4 | 55 | 2.24E-06 | MLH1, MLH3, MSH4, MSH5 |
| HSA-1650814 | Collagen biosynthesis and modifying enzymes | 3 | 67 | 0.00013 | COL26A1, COL27A1, SERPINH1 |
| HSA-8948216 | Collagen chain trimerization | 2 | 44 | 0.003 | COL26A1, COL27A1 |

**Note**: FDR, false discovery rate.

^a^ The number of proteins matched with the corresponding pathway

^b^ Total number of proteins belong to the corresponding pathway.

^c^ List of proteins matched with the corresponding pathway. Blue font indicates the common target genes.

**Table S20 KEGG pathways significantly enriched with the common targets and the predicted functional partners.**

| **Term ID** | **Term description** | **Matched ^a^** | **Total ^b^** | **FDR** | **Matched proteins (labels) ^c^** |
| --- | --- | --- | --- | --- | --- |
| hsa03430 | Mismatch repair | 2 | 23 | 0.00097 | MLH1, MLH3 |
| hsa03460 | Fanconi anemia pathway | 2 | 51 | 0.00020 | MLH1, RAD51 |

**Note**: FDR, false discovery rate.

^a^ The number of proteins matched with the corresponding pathway

^b^ Total number of proteins belong to the corresponding pathway.

^c^ List of proteins matched with the corresponding pathway. Blue font indicates the common target genes.

**Table S21** The RNA binding proteins (RBPs) and binding motifs of lnc-FAM32A-2:1 and lnc-MDK-4:2

-

| **LncRNA** | **Score** | **Relative Score** | **RBP Name ^a^** | **Start** | **End** | **Matching sequence** | **Matrix ID ^a^** | **Download PWM ^a^** | **Download PFM ^a^** |
| --- | --- | --- | --- | --- | --- | --- | --- | --- | --- |
| lnc-FAM32A-2:1 | 8.9484945 | 100% | [NONO](http://rbpdb.ccbr.utoronto.ca/proteins.php?PME_sys_operation=PME_op_View&PME_sys_rec=1486) | 379 | 383 | AGGGA | [488_9001221](http://rbpdb.ccbr.utoronto.ca/experiments.php?exp_id=488) | [Download PWM](http://rbpdb.ccbr.utoronto.ca/PWMDir/488_9001221.pwm) | [Download PFM](http://rbpdb.ccbr.utoronto.ca/PFMDir/488_9001221.pfm) |
| lnc-FAM32A-2:1 | 8.883996 | 87% | [SNRPA](http://rbpdb.ccbr.utoronto.ca/proteins.php?PME_sys_operation=PME_op_View&PME_sys_rec=1274) | 227 | 233 | UGGAGAU | [949_10094314](http://rbpdb.ccbr.utoronto.ca/experiments.php?exp_id=949) | [Download PWM](http://rbpdb.ccbr.utoronto.ca/PWMDir/949_10094314.pwm) | [Download PFM](http://rbpdb.ccbr.utoronto.ca/PFMDir/949_10094314.pfm) |
| lnc-FAM32A-2:1 | 8.7178165 | 100% | [PABPC1](http://rbpdb.ccbr.utoronto.ca/proteins.php?PME_sys_operation=PME_op_View&PME_sys_rec=1262) | 566 | 570 | AAAAA | [24_7908267](http://rbpdb.ccbr.utoronto.ca/experiments.php?exp_id=24) | [Download PWM](http://rbpdb.ccbr.utoronto.ca/PWMDir/24_7908267.pwm) | [Download PFM](http://rbpdb.ccbr.utoronto.ca/PFMDir/24_7908267.pfm) |
| lnc-FAM32A-2:1 | 7.96303354 | 94% | [PABPC1](http://rbpdb.ccbr.utoronto.ca/proteins.php?PME_sys_operation=PME_op_View&PME_sys_rec=1262) | 7 | 13 | ACAAAUC | [950_7908267](http://rbpdb.ccbr.utoronto.ca/experiments.php?exp_id=950) | [Download PWM](http://rbpdb.ccbr.utoronto.ca/PWMDir/950_7908267.pwm) | [Download PFM](http://rbpdb.ccbr.utoronto.ca/PFMDir/950_7908267.pfm) |
| lnc-FAM32A-2:1 | 7.945691304 | 84% | [ybx2-a](http://rbpdb.ccbr.utoronto.ca/proteins.php?PME_sys_operation=PME_op_View&PME_sys_rec=2571) | 168 | 173 | AAGAUC | [114_7499328](http://rbpdb.ccbr.utoronto.ca/experiments.php?exp_id=114) | [Download PWM](http://rbpdb.ccbr.utoronto.ca/PWMDir/114_7499328.pwm) | [Download PFM](http://rbpdb.ccbr.utoronto.ca/PFMDir/114_7499328.pfm) |
| lnc-FAM32A-2:1 | 7.89298258 | 84% | [ybx2-a](http://rbpdb.ccbr.utoronto.ca/proteins.php?PME_sys_operation=PME_op_View&PME_sys_rec=2571) | 270 | 275 | AACACC | [115_7499328](http://rbpdb.ccbr.utoronto.ca/experiments.php?exp_id=115) | [Download PWM](http://rbpdb.ccbr.utoronto.ca/PWMDir/115_7499328.pwm) | [Download PFM](http://rbpdb.ccbr.utoronto.ca/PFMDir/115_7499328.pfm) |
| lnc-FAM32A-2:1 | 7.3693752 | 100% | [FUS](http://rbpdb.ccbr.utoronto.ca/proteins.php?PME_sys_operation=PME_op_View&PME_sys_rec=1289) | 125 | 128 | GGUG | [637_11098054](http://rbpdb.ccbr.utoronto.ca/experiments.php?exp_id=637) | [Download PWM](http://rbpdb.ccbr.utoronto.ca/PWMDir/637_11098054.pwm) | [Download PFM](http://rbpdb.ccbr.utoronto.ca/PFMDir/637_11098054.pfm) |
| lnc-FAM32A-2:1 | 7.3693752 | 100% | [FUS](http://rbpdb.ccbr.utoronto.ca/proteins.php?PME_sys_operation=PME_op_View&PME_sys_rec=1289) | 206 | 209 | GGUG | [637_11098054](http://rbpdb.ccbr.utoronto.ca/experiments.php?exp_id=637) | [Download PWM](http://rbpdb.ccbr.utoronto.ca/PWMDir/637_11098054.pwm) | [Download PFM](http://rbpdb.ccbr.utoronto.ca/PFMDir/637_11098054.pfm) |
| lnc-FAM32A-2:1 | 7.3693752 | 100% | [FUS](http://rbpdb.ccbr.utoronto.ca/proteins.php?PME_sys_operation=PME_op_View&PME_sys_rec=1289) | 293 | 296 | GGUG | [637_11098054](http://rbpdb.ccbr.utoronto.ca/experiments.php?exp_id=637) | [Download PWM](http://rbpdb.ccbr.utoronto.ca/PWMDir/637_11098054.pwm) | [Download PFM](http://rbpdb.ccbr.utoronto.ca/PFMDir/637_11098054.pfm) |
| lnc-FAM32A-2:1 | 7.3693752 | 100% | [FUS](http://rbpdb.ccbr.utoronto.ca/proteins.php?PME_sys_operation=PME_op_View&PME_sys_rec=1289) | 155 | 158 | GGUG | [637_11098054](http://rbpdb.ccbr.utoronto.ca/experiments.php?exp_id=637) | [Download PWM](http://rbpdb.ccbr.utoronto.ca/PWMDir/637_11098054.pwm) | [Download PFM](http://rbpdb.ccbr.utoronto.ca/PFMDir/637_11098054.pfm) |
| lnc-FAM32A-2:1 | 7.2294196 | 100% | [Pum2](http://rbpdb.ccbr.utoronto.ca/proteins.php?PME_sys_operation=PME_op_View&PME_sys_rec=1800) | 523 | 526 | UGUA | [329_11780640](http://rbpdb.ccbr.utoronto.ca/experiments.php?exp_id=329) | [Download PWM](http://rbpdb.ccbr.utoronto.ca/PWMDir/329_11780640.pwm) | [Download PFM](http://rbpdb.ccbr.utoronto.ca/PFMDir/329_11780640.pfm) |
| lnc-FAM32A-2:1 | 6.93292615 | 94% | [ACO1](http://rbpdb.ccbr.utoronto.ca/proteins.php?PME_sys_operation=PME_op_View&PME_sys_rec=2664) | 249 | 254 | CAGUGA | [1213_8021254](http://rbpdb.ccbr.utoronto.ca/experiments.php?exp_id=1213) | [Download PWM](http://rbpdb.ccbr.utoronto.ca/PWMDir/1213_8021254.pwm) | [Download PFM](http://rbpdb.ccbr.utoronto.ca/PFMDir/1213_8021254.pfm) |
| lnc-FAM32A-2:1 | 6.6279899 | 100% | [MBNL1](http://rbpdb.ccbr.utoronto.ca/proteins.php?PME_sys_operation=PME_op_View&PME_sys_rec=1507) | 406 | 409 | UGCU | [669_20071745](http://rbpdb.ccbr.utoronto.ca/experiments.php?exp_id=669) | [Download PWM](http://rbpdb.ccbr.utoronto.ca/PWMDir/669_20071745.pwm) | [Download PFM](http://rbpdb.ccbr.utoronto.ca/PFMDir/669_20071745.pfm) |
| lnc-FAM32A-2:1 | 6.4668404 | 100% | [EIF4B](http://rbpdb.ccbr.utoronto.ca/proteins.php?PME_sys_operation=PME_op_View&PME_sys_rec=1251) | 501 | 504 | GGAA | [352_8846295](http://rbpdb.ccbr.utoronto.ca/experiments.php?exp_id=352) | [Download PWM](http://rbpdb.ccbr.utoronto.ca/PWMDir/352_8846295.pwm) | [Download PFM](http://rbpdb.ccbr.utoronto.ca/PFMDir/352_8846295.pfm) |
| lnc-FAM32A-2:1 | 6.6279899 | 100% | [MBNL1](http://rbpdb.ccbr.utoronto.ca/proteins.php?PME_sys_operation=PME_op_View&PME_sys_rec=1507) | 139 | 142 | UGCU | [669_20071745](http://rbpdb.ccbr.utoronto.ca/experiments.php?exp_id=669) | [Download PWM](http://rbpdb.ccbr.utoronto.ca/PWMDir/669_20071745.pwm) | [Download PFM](http://rbpdb.ccbr.utoronto.ca/PFMDir/669_20071745.pfm) |
| lnc-FAM32A-2:1 | 6.6279899 | 100% | [MBNL1](http://rbpdb.ccbr.utoronto.ca/proteins.php?PME_sys_operation=PME_op_View&PME_sys_rec=1507) | 127 | 130 | UGCU | [669_20071745](http://rbpdb.ccbr.utoronto.ca/experiments.php?exp_id=669) | [Download PWM](http://rbpdb.ccbr.utoronto.ca/PWMDir/669_20071745.pwm) | [Download PFM](http://rbpdb.ccbr.utoronto.ca/PFMDir/669_20071745.pfm) |
| lnc-FAM32A-2:1 | 6.6279899 | 100% | [MBNL1](http://rbpdb.ccbr.utoronto.ca/proteins.php?PME_sys_operation=PME_op_View&PME_sys_rec=1507) | 544 | 547 | UGCU | [669_20071745](http://rbpdb.ccbr.utoronto.ca/experiments.php?exp_id=669) | [Download PWM](http://rbpdb.ccbr.utoronto.ca/PWMDir/669_20071745.pwm) | [Download PFM](http://rbpdb.ccbr.utoronto.ca/PFMDir/669_20071745.pfm) |
| lnc-FAM32A-2:1 | 6.6279899 | 100% | [MBNL1](http://rbpdb.ccbr.utoronto.ca/proteins.php?PME_sys_operation=PME_op_View&PME_sys_rec=1507) | 36 | 39 | UGCU | [669_20071745](http://rbpdb.ccbr.utoronto.ca/experiments.php?exp_id=669) | [Download PWM](http://rbpdb.ccbr.utoronto.ca/PWMDir/669_20071745.pwm) | [Download PFM](http://rbpdb.ccbr.utoronto.ca/PFMDir/669_20071745.pfm) |
| lnc-FAM32A-2:1 | 6.33985 | 100% | [KHSRP](http://rbpdb.ccbr.utoronto.ca/proteins.php?PME_sys_operation=PME_op_View&PME_sys_rec=2661) | 1 | 4 | GUCC | [1186_17893325](http://rbpdb.ccbr.utoronto.ca/experiments.php?exp_id=1186) | [Download PWM](http://rbpdb.ccbr.utoronto.ca/PWMDir/1186_17893325.pwm) | [Download PFM](http://rbpdb.ccbr.utoronto.ca/PFMDir/1186_17893325.pfm) |
| lnc-FAM32A-2:1 | 6.4668404 | 100% | [EIF4B](http://rbpdb.ccbr.utoronto.ca/proteins.php?PME_sys_operation=PME_op_View&PME_sys_rec=1251) | 87 | 90 | GGAA | [352_8846295](http://rbpdb.ccbr.utoronto.ca/experiments.php?exp_id=352) | [Download PWM](http://rbpdb.ccbr.utoronto.ca/PWMDir/352_8846295.pwm) | [Download PFM](http://rbpdb.ccbr.utoronto.ca/PFMDir/352_8846295.pfm) |
| lnc-FAM32A-2:1 | 6.33890598 | 100% | [YBX1](http://rbpdb.ccbr.utoronto.ca/proteins.php?PME_sys_operation=PME_op_View&PME_sys_rec=1257) | 120 | 125 | CCUGCG | [1177_19561594](http://rbpdb.ccbr.utoronto.ca/experiments.php?exp_id=1177) | [Download PWM](http://rbpdb.ccbr.utoronto.ca/PWMDir/1177_19561594.pwm) | [Download PFM](http://rbpdb.ccbr.utoronto.ca/PFMDir/1177_19561594.pfm) |
| lnc-FAM32A-2:1 | 5.65374333 | 88% | [Vts1](http://rbpdb.ccbr.utoronto.ca/proteins.php?PME_sys_operation=PME_op_View&PME_sys_rec=2586) | 452 | 458 | GCUGGUC | [1176_19561594](http://rbpdb.ccbr.utoronto.ca/experiments.php?exp_id=1176) | [Download PWM](http://rbpdb.ccbr.utoronto.ca/PWMDir/1176_19561594.pwm) | [Download PFM](http://rbpdb.ccbr.utoronto.ca/PFMDir/1176_19561594.pfm) |
| lnc-FAM32A-2:1 | 6.17832025 | 93% | [MBNL1](http://rbpdb.ccbr.utoronto.ca/proteins.php?PME_sys_operation=PME_op_View&PME_sys_rec=1507) | 159 | 162 | CGCU | [669_20071745](http://rbpdb.ccbr.utoronto.ca/experiments.php?exp_id=669) | [Download PWM](http://rbpdb.ccbr.utoronto.ca/PWMDir/669_20071745.pwm) | [Download PFM](http://rbpdb.ccbr.utoronto.ca/PFMDir/669_20071745.pfm) |
| lnc-FAM32A-2:1 | 5.830231484 | 82% | [SFRS9](http://rbpdb.ccbr.utoronto.ca/proteins.php?PME_sys_operation=PME_op_View&PME_sys_rec=1358) | 299 | 303 | AGCAC | [797_17548433](http://rbpdb.ccbr.utoronto.ca/experiments.php?exp_id=797) | [Download PWM](http://rbpdb.ccbr.utoronto.ca/PWMDir/797_17548433.pwm) | [Download PFM](http://rbpdb.ccbr.utoronto.ca/PFMDir/797_17548433.pfm) |
| lnc-FAM32A-2:1 | 5.2682554 | 100% | [RBMX](http://rbpdb.ccbr.utoronto.ca/proteins.php?PME_sys_operation=PME_op_View&PME_sys_rec=1487) | 65 | 68 | CCAG | [922_19282290](http://rbpdb.ccbr.utoronto.ca/experiments.php?exp_id=922) | [Download PWM](http://rbpdb.ccbr.utoronto.ca/PWMDir/922_19282290.pwm) | [Download PFM](http://rbpdb.ccbr.utoronto.ca/PFMDir/922_19282290.pfm) |
| lnc-FAM32A-2:1 | 4.62028767 | 100% | [SFRS1](http://rbpdb.ccbr.utoronto.ca/proteins.php?PME_sys_operation=PME_op_View&PME_sys_rec=1448) | 86 | 89 | AGGA | [1173_19561594](http://rbpdb.ccbr.utoronto.ca/experiments.php?exp_id=1173) | [Download PWM](http://rbpdb.ccbr.utoronto.ca/PWMDir/1173_19561594.pwm) | [Download PFM](http://rbpdb.ccbr.utoronto.ca/PFMDir/1173_19561594.pfm) |
| lnc-FAM32A-2:1 | 5.306199555 | 82% | [Vts1](http://rbpdb.ccbr.utoronto.ca/proteins.php?PME_sys_operation=PME_op_View&PME_sys_rec=2586) | 113 | 119 | GCGGGAG | [1176_19561594](http://rbpdb.ccbr.utoronto.ca/experiments.php?exp_id=1176) | [Download PWM](http://rbpdb.ccbr.utoronto.ca/PWMDir/1176_19561594.pwm) | [Download PFM](http://rbpdb.ccbr.utoronto.ca/PFMDir/1176_19561594.pfm) |
| lnc-FAM32A-2:1 | 4.40359056 | 100% | [ELAVL1](http://rbpdb.ccbr.utoronto.ca/proteins.php?PME_sys_operation=PME_op_View&PME_sys_rec=1258) | 493 | 496 | GUUU | [1170_19561594](http://rbpdb.ccbr.utoronto.ca/experiments.php?exp_id=1170) | [Download PWM](http://rbpdb.ccbr.utoronto.ca/PWMDir/1170_19561594.pwm) | [Download PFM](http://rbpdb.ccbr.utoronto.ca/PFMDir/1170_19561594.pfm) |
| lnc-FAM32A-2:1 | 5.2682554 | 100% | [RBMX](http://rbpdb.ccbr.utoronto.ca/proteins.php?PME_sys_operation=PME_op_View&PME_sys_rec=1487) | 236 | 239 | CCAG | [922_19282290](http://rbpdb.ccbr.utoronto.ca/experiments.php?exp_id=922) | [Download PWM](http://rbpdb.ccbr.utoronto.ca/PWMDir/922_19282290.pwm) | [Download PFM](http://rbpdb.ccbr.utoronto.ca/PFMDir/922_19282290.pfm) |
| lnc-FAM32A-2:1 | 5.2682554 | 100% | [RBMX](http://rbpdb.ccbr.utoronto.ca/proteins.php?PME_sys_operation=PME_op_View&PME_sys_rec=1487) | 152 | 155 | CCAG | [922_19282290](http://rbpdb.ccbr.utoronto.ca/experiments.php?exp_id=922) | [Download PWM](http://rbpdb.ccbr.utoronto.ca/PWMDir/922_19282290.pwm) | [Download PFM](http://rbpdb.ccbr.utoronto.ca/PFMDir/922_19282290.pfm) |
| lnc-FAM32A-2:1 | 5.2682554 | 100% | [RBMX](http://rbpdb.ccbr.utoronto.ca/proteins.php?PME_sys_operation=PME_op_View&PME_sys_rec=1487) | 557 | 560 | CCAG | [922_19282290](http://rbpdb.ccbr.utoronto.ca/experiments.php?exp_id=922) | [Download PWM](http://rbpdb.ccbr.utoronto.ca/PWMDir/922_19282290.pwm) | [Download PFM](http://rbpdb.ccbr.utoronto.ca/PFMDir/922_19282290.pfm) |
| lnc-FAM32A-2:1 | 5.2682554 | 100% | [RBMX](http://rbpdb.ccbr.utoronto.ca/proteins.php?PME_sys_operation=PME_op_View&PME_sys_rec=1487) | 396 | 399 | CCAG | [922_19282290](http://rbpdb.ccbr.utoronto.ca/experiments.php?exp_id=922) | [Download PWM](http://rbpdb.ccbr.utoronto.ca/PWMDir/922_19282290.pwm) | [Download PFM](http://rbpdb.ccbr.utoronto.ca/PFMDir/922_19282290.pfm) |
| lnc-FAM32A-2:1 | 5.2682554 | 100% | [RBMX](http://rbpdb.ccbr.utoronto.ca/proteins.php?PME_sys_operation=PME_op_View&PME_sys_rec=1487) | 443 | 446 | CCAG | [922_19282290](http://rbpdb.ccbr.utoronto.ca/experiments.php?exp_id=922) | [Download PWM](http://rbpdb.ccbr.utoronto.ca/PWMDir/922_19282290.pwm) | [Download PFM](http://rbpdb.ccbr.utoronto.ca/PFMDir/922_19282290.pfm) |
| lnc-FAM32A-2:1 | 5.2682554 | 100% | [RBMX](http://rbpdb.ccbr.utoronto.ca/proteins.php?PME_sys_operation=PME_op_View&PME_sys_rec=1487) | 328 | 331 | CCAG | [922_19282290](http://rbpdb.ccbr.utoronto.ca/experiments.php?exp_id=922) | [Download PWM](http://rbpdb.ccbr.utoronto.ca/PWMDir/922_19282290.pwm) | [Download PFM](http://rbpdb.ccbr.utoronto.ca/PFMDir/922_19282290.pfm) |
| lnc-FAM32A-2:1 | 5.2682554 | 100% | [RBMX](http://rbpdb.ccbr.utoronto.ca/proteins.php?PME_sys_operation=PME_op_View&PME_sys_rec=1487) | 303 | 306 | CCAG | [922_19282290](http://rbpdb.ccbr.utoronto.ca/experiments.php?exp_id=922) | [Download PWM](http://rbpdb.ccbr.utoronto.ca/PWMDir/922_19282290.pwm) | [Download PFM](http://rbpdb.ccbr.utoronto.ca/PFMDir/922_19282290.pfm) |
| lnc-FAM32A-2:1 | 5.2682554 | 100% | [RBMX](http://rbpdb.ccbr.utoronto.ca/proteins.php?PME_sys_operation=PME_op_View&PME_sys_rec=1487) | 377 | 380 | CCAG | [922_19282290](http://rbpdb.ccbr.utoronto.ca/experiments.php?exp_id=922) | [Download PWM](http://rbpdb.ccbr.utoronto.ca/PWMDir/922_19282290.pwm) | [Download PFM](http://rbpdb.ccbr.utoronto.ca/PFMDir/922_19282290.pfm) |
| lnc-FAM32A-2:1 | 5.2682554 | 100% | [RBMX](http://rbpdb.ccbr.utoronto.ca/proteins.php?PME_sys_operation=PME_op_View&PME_sys_rec=1487) | 191 | 194 | CCAG | [922_19282290](http://rbpdb.ccbr.utoronto.ca/experiments.php?exp_id=922) | [Download PWM](http://rbpdb.ccbr.utoronto.ca/PWMDir/922_19282290.pwm) | [Download PFM](http://rbpdb.ccbr.utoronto.ca/PFMDir/922_19282290.pfm) |
| lnc-FAM32A-2:1 | 5.2682554 | 100% | [RBMX](http://rbpdb.ccbr.utoronto.ca/proteins.php?PME_sys_operation=PME_op_View&PME_sys_rec=1487) | 248 | 251 | CCAG | [922_19282290](http://rbpdb.ccbr.utoronto.ca/experiments.php?exp_id=922) | [Download PWM](http://rbpdb.ccbr.utoronto.ca/PWMDir/922_19282290.pwm) | [Download PFM](http://rbpdb.ccbr.utoronto.ca/PFMDir/922_19282290.pfm) |
| lnc-FAM32A-2:1 | 5.2682554 | 100% | [RBMX](http://rbpdb.ccbr.utoronto.ca/proteins.php?PME_sys_operation=PME_op_View&PME_sys_rec=1487) | 3 | 6 | CCAG | [922_19282290](http://rbpdb.ccbr.utoronto.ca/experiments.php?exp_id=922) | [Download PWM](http://rbpdb.ccbr.utoronto.ca/PWMDir/922_19282290.pwm) | [Download PFM](http://rbpdb.ccbr.utoronto.ca/PFMDir/922_19282290.pfm) |
| lnc-FAM32A-2:1 | 5.2682554 | 100% | [RBMX](http://rbpdb.ccbr.utoronto.ca/proteins.php?PME_sys_operation=PME_op_View&PME_sys_rec=1487) | 81 | 84 | CCAG | [922_19282290](http://rbpdb.ccbr.utoronto.ca/experiments.php?exp_id=922) | [Download PWM](http://rbpdb.ccbr.utoronto.ca/PWMDir/922_19282290.pwm) | [Download PFM](http://rbpdb.ccbr.utoronto.ca/PFMDir/922_19282290.pfm) |
| lnc-FAM32A-2:1 | 5.2682554 | 100% | [RBMX](http://rbpdb.ccbr.utoronto.ca/proteins.php?PME_sys_operation=PME_op_View&PME_sys_rec=1487) | 366 | 369 | CCAG | [922_19282290](http://rbpdb.ccbr.utoronto.ca/experiments.php?exp_id=922) | [Download PWM](http://rbpdb.ccbr.utoronto.ca/PWMDir/922_19282290.pwm) | [Download PFM](http://rbpdb.ccbr.utoronto.ca/PFMDir/922_19282290.pfm) |
| lnc-FAM32A-2:1 | 5.2682554 | 100% | [RBMX](http://rbpdb.ccbr.utoronto.ca/proteins.php?PME_sys_operation=PME_op_View&PME_sys_rec=1487) | 71 | 74 | CCAG | [922_19282290](http://rbpdb.ccbr.utoronto.ca/experiments.php?exp_id=922) | [Download PWM](http://rbpdb.ccbr.utoronto.ca/PWMDir/922_19282290.pwm) | [Download PFM](http://rbpdb.ccbr.utoronto.ca/PFMDir/922_19282290.pfm) |
| lnc-FAM32A-2:1 | 5.256645851 | 81% | [EIF4B](http://rbpdb.ccbr.utoronto.ca/proteins.php?PME_sys_operation=PME_op_View&PME_sys_rec=1251) | 102 | 105 | GGAC | [352_8846295](http://rbpdb.ccbr.utoronto.ca/experiments.php?exp_id=352) | [Download PWM](http://rbpdb.ccbr.utoronto.ca/PWMDir/352_8846295.pwm) | [Download PFM](http://rbpdb.ccbr.utoronto.ca/PFMDir/352_8846295.pfm) |
| lnc-FAM32A-2:1 | 5.256645851 | 81% | [EIF4B](http://rbpdb.ccbr.utoronto.ca/proteins.php?PME_sys_operation=PME_op_View&PME_sys_rec=1251) | 381 | 384 | GGAC | [352_8846295](http://rbpdb.ccbr.utoronto.ca/experiments.php?exp_id=352) | [Download PWM](http://rbpdb.ccbr.utoronto.ca/PWMDir/352_8846295.pwm) | [Download PFM](http://rbpdb.ccbr.utoronto.ca/PFMDir/352_8846295.pfm) |
| lnc-FAM32A-2:1 | 5.256645851 | 81% | [EIF4B](http://rbpdb.ccbr.utoronto.ca/proteins.php?PME_sys_operation=PME_op_View&PME_sys_rec=1251) | 308 | 311 | GGAC | [352_8846295](http://rbpdb.ccbr.utoronto.ca/experiments.php?exp_id=352) | [Download PWM](http://rbpdb.ccbr.utoronto.ca/PWMDir/352_8846295.pwm) | [Download PFM](http://rbpdb.ccbr.utoronto.ca/PFMDir/352_8846295.pfm) |
| lnc-FAM32A-2:1 | 5.256645851 | 81% | [EIF4B](http://rbpdb.ccbr.utoronto.ca/proteins.php?PME_sys_operation=PME_op_View&PME_sys_rec=1251) | 244 | 247 | GGAC | [352_8846295](http://rbpdb.ccbr.utoronto.ca/experiments.php?exp_id=352) | [Download PWM](http://rbpdb.ccbr.utoronto.ca/PWMDir/352_8846295.pwm) | [Download PFM](http://rbpdb.ccbr.utoronto.ca/PFMDir/352_8846295.pfm) |
| lnc-FAM32A-2:1 | 4.99861593 | 94% | [RBMX](http://rbpdb.ccbr.utoronto.ca/proteins.php?PME_sys_operation=PME_op_View&PME_sys_rec=1487) | 401 | 404 | CCAU | [922_19282290](http://rbpdb.ccbr.utoronto.ca/experiments.php?exp_id=922) | [Download PWM](http://rbpdb.ccbr.utoronto.ca/PWMDir/922_19282290.pwm) | [Download PFM](http://rbpdb.ccbr.utoronto.ca/PFMDir/922_19282290.pfm) |
| lnc-FAM32A-2:1 | 4.6667232 | 88% | [RBMX](http://rbpdb.ccbr.utoronto.ca/proteins.php?PME_sys_operation=PME_op_View&PME_sys_rec=1487) | 325 | 328 | CCAC | [922_19282290](http://rbpdb.ccbr.utoronto.ca/experiments.php?exp_id=922) | [Download PWM](http://rbpdb.ccbr.utoronto.ca/PWMDir/922_19282290.pwm) | [Download PFM](http://rbpdb.ccbr.utoronto.ca/PFMDir/922_19282290.pfm) |
| lnc-FAM32A-2:1 | 4.6667232 | 88% | [RBMX](http://rbpdb.ccbr.utoronto.ca/proteins.php?PME_sys_operation=PME_op_View&PME_sys_rec=1487) | 358 | 361 | CCAC | [922_19282290](http://rbpdb.ccbr.utoronto.ca/experiments.php?exp_id=922) | [Download PWM](http://rbpdb.ccbr.utoronto.ca/PWMDir/922_19282290.pwm) | [Download PFM](http://rbpdb.ccbr.utoronto.ca/PFMDir/922_19282290.pfm) |
| lnc-FAM32A-2:1 | 4.62028767 | 100% | [SFRS1](http://rbpdb.ccbr.utoronto.ca/proteins.php?PME_sys_operation=PME_op_View&PME_sys_rec=1448) | 504 | 507 | AGGA | [1173_19561594](http://rbpdb.ccbr.utoronto.ca/experiments.php?exp_id=1173) | [Download PWM](http://rbpdb.ccbr.utoronto.ca/PWMDir/1173_19561594.pwm) | [Download PFM](http://rbpdb.ccbr.utoronto.ca/PFMDir/1173_19561594.pfm) |
| lnc-FAM32A-2:1 | 4.40359056 | 100% | [ELAVL1](http://rbpdb.ccbr.utoronto.ca/proteins.php?PME_sys_operation=PME_op_View&PME_sys_rec=1258) | 472 | 475 | GUUU | [1170_19561594](http://rbpdb.ccbr.utoronto.ca/experiments.php?exp_id=1170) | [Download PWM](http://rbpdb.ccbr.utoronto.ca/PWMDir/1170_19561594.pwm) | [Download PFM](http://rbpdb.ccbr.utoronto.ca/PFMDir/1170_19561594.pfm) |
| lnc-FAM32A-2:1 | 4.40271173 | 83% | [RBMX](http://rbpdb.ccbr.utoronto.ca/proteins.php?PME_sys_operation=PME_op_View&PME_sys_rec=1487) | 275 | 278 | CCCG | [922_19282290](http://rbpdb.ccbr.utoronto.ca/experiments.php?exp_id=922) | [Download PWM](http://rbpdb.ccbr.utoronto.ca/PWMDir/922_19282290.pwm) | [Download PFM](http://rbpdb.ccbr.utoronto.ca/PFMDir/922_19282290.pfm) |
| lnc-FAM32A-2:1 | 4.316830439 | 84% | [SFRS13A](http://rbpdb.ccbr.utoronto.ca/proteins.php?PME_sys_operation=PME_op_View&PME_sys_rec=1649) | 89 | 95 | AAAGAUU | [1169_19561594](http://rbpdb.ccbr.utoronto.ca/experiments.php?exp_id=1169) | [Download PWM](http://rbpdb.ccbr.utoronto.ca/PWMDir/1169_19561594.pwm) | [Download PFM](http://rbpdb.ccbr.utoronto.ca/PFMDir/1169_19561594.pfm) |
| lnc-FAM32A-2:1 | 4.1881759 | 90% | [SFRS1](http://rbpdb.ccbr.utoronto.ca/proteins.php?PME_sys_operation=PME_op_View&PME_sys_rec=1448) | 227 | 230 | UGGA | [1173_19561594](http://rbpdb.ccbr.utoronto.ca/experiments.php?exp_id=1173) | [Download PWM](http://rbpdb.ccbr.utoronto.ca/PWMDir/1173_19561594.pwm) | [Download PFM](http://rbpdb.ccbr.utoronto.ca/PFMDir/1173_19561594.pfm) |
| lnc-FAM32A-2:1 | 4.1881759 | 90% | [SFRS1](http://rbpdb.ccbr.utoronto.ca/proteins.php?PME_sys_operation=PME_op_View&PME_sys_rec=1448) | 333 | 336 | UGGA | [1173_19561594](http://rbpdb.ccbr.utoronto.ca/experiments.php?exp_id=1173) | [Download PWM](http://rbpdb.ccbr.utoronto.ca/PWMDir/1173_19561594.pwm) | [Download PFM](http://rbpdb.ccbr.utoronto.ca/PFMDir/1173_19561594.pfm) |
| lnc-FAM32A-2:1 | 3.82636396 | 86% | [ELAVL1](http://rbpdb.ccbr.utoronto.ca/proteins.php?PME_sys_operation=PME_op_View&PME_sys_rec=1258) | 514 | 517 | AUUU | [1170_19561594](http://rbpdb.ccbr.utoronto.ca/experiments.php?exp_id=1170) | [Download PWM](http://rbpdb.ccbr.utoronto.ca/PWMDir/1170_19561594.pwm) | [Download PFM](http://rbpdb.ccbr.utoronto.ca/PFMDir/1170_19561594.pfm) |
| lnc-MDK-4:2 | 7.3693752 | 100% | [FUS](http://rbpdb.ccbr.utoronto.ca/proteins.php?PME_sys_operation=PME_op_View&PME_sys_rec=1289) | 163 | 166 | GGUG | [637_11098054](http://rbpdb.ccbr.utoronto.ca/experiments.php?exp_id=637) | [Download PWM](http://rbpdb.ccbr.utoronto.ca/PWMDir/637_11098054.pwm) | [Download PFM](http://rbpdb.ccbr.utoronto.ca/PFMDir/637_11098054.pfm) |
| lnc-MDK-4:2 | 7.3693752 | 100% | [FUS](http://rbpdb.ccbr.utoronto.ca/proteins.php?PME_sys_operation=PME_op_View&PME_sys_rec=1289) | 159 | 162 | GGUG | [637_11098054](http://rbpdb.ccbr.utoronto.ca/experiments.php?exp_id=637) | [Download PWM](http://rbpdb.ccbr.utoronto.ca/PWMDir/637_11098054.pwm) | [Download PFM](http://rbpdb.ccbr.utoronto.ca/PFMDir/637_11098054.pfm) |
| lnc-MDK-4:2 | 7.2294196 | 100% | [Pum2](http://rbpdb.ccbr.utoronto.ca/proteins.php?PME_sys_operation=PME_op_View&PME_sys_rec=1800) | 547 | 550 | UGUA | [329_11780640](http://rbpdb.ccbr.utoronto.ca/experiments.php?exp_id=329) | [Download PWM](http://rbpdb.ccbr.utoronto.ca/PWMDir/329_11780640.pwm) | [Download PFM](http://rbpdb.ccbr.utoronto.ca/PFMDir/329_11780640.pfm) |
| lnc-MDK-4:2 | 7.2294196 | 100% | [Pum2](http://rbpdb.ccbr.utoronto.ca/proteins.php?PME_sys_operation=PME_op_View&PME_sys_rec=1800) | 147 | 150 | UGUA | [329_11780640](http://rbpdb.ccbr.utoronto.ca/experiments.php?exp_id=329) | [Download PWM](http://rbpdb.ccbr.utoronto.ca/PWMDir/329_11780640.pwm) | [Download PFM](http://rbpdb.ccbr.utoronto.ca/PFMDir/329_11780640.pfm) |
| lnc-MDK-4:2 | 6.63255189 | 93% | [SFRS9](http://rbpdb.ccbr.utoronto.ca/proteins.php?PME_sys_operation=PME_op_View&PME_sys_rec=1358) | 215 | 219 | AGGAG | [797_17548433](http://rbpdb.ccbr.utoronto.ca/experiments.php?exp_id=797) | [Download PWM](http://rbpdb.ccbr.utoronto.ca/PWMDir/797_17548433.pwm) | [Download PFM](http://rbpdb.ccbr.utoronto.ca/PFMDir/797_17548433.pfm) |
| lnc-MDK-4:2 | 6.6279899 | 100% | [MBNL1](http://rbpdb.ccbr.utoronto.ca/proteins.php?PME_sys_operation=PME_op_View&PME_sys_rec=1507) | 524 | 527 | UGCU | [669_20071745](http://rbpdb.ccbr.utoronto.ca/experiments.php?exp_id=669) | [Download PWM](http://rbpdb.ccbr.utoronto.ca/PWMDir/669_20071745.pwm) | [Download PFM](http://rbpdb.ccbr.utoronto.ca/PFMDir/669_20071745.pfm) |
| lnc-MDK-4:2 | 6.6279899 | 100% | [MBNL1](http://rbpdb.ccbr.utoronto.ca/proteins.php?PME_sys_operation=PME_op_View&PME_sys_rec=1507) | 558 | 561 | UGCU | [669_20071745](http://rbpdb.ccbr.utoronto.ca/experiments.php?exp_id=669) | [Download PWM](http://rbpdb.ccbr.utoronto.ca/PWMDir/669_20071745.pwm) | [Download PFM](http://rbpdb.ccbr.utoronto.ca/PFMDir/669_20071745.pfm) |
| lnc-MDK-4:2 | 6.6279899 | 100% | [MBNL1](http://rbpdb.ccbr.utoronto.ca/proteins.php?PME_sys_operation=PME_op_View&PME_sys_rec=1507) | 347 | 350 | UGCU | [669_20071745](http://rbpdb.ccbr.utoronto.ca/experiments.php?exp_id=669) | [Download PWM](http://rbpdb.ccbr.utoronto.ca/PWMDir/669_20071745.pwm) | [Download PFM](http://rbpdb.ccbr.utoronto.ca/PFMDir/669_20071745.pfm) |
| lnc-MDK-4:2 | 6.6279899 | 100% | [MBNL1](http://rbpdb.ccbr.utoronto.ca/proteins.php?PME_sys_operation=PME_op_View&PME_sys_rec=1507) | 127 | 130 | UGCU | [669_20071745](http://rbpdb.ccbr.utoronto.ca/experiments.php?exp_id=669) | [Download PWM](http://rbpdb.ccbr.utoronto.ca/PWMDir/669_20071745.pwm) | [Download PFM](http://rbpdb.ccbr.utoronto.ca/PFMDir/669_20071745.pfm) |
| lnc-MDK-4:2 | 6.6279899 | 100% | [MBNL1](http://rbpdb.ccbr.utoronto.ca/proteins.php?PME_sys_operation=PME_op_View&PME_sys_rec=1507) | 12 | 15 | UGCU | [669_20071745](http://rbpdb.ccbr.utoronto.ca/experiments.php?exp_id=669) | [Download PWM](http://rbpdb.ccbr.utoronto.ca/PWMDir/669_20071745.pwm) | [Download PFM](http://rbpdb.ccbr.utoronto.ca/PFMDir/669_20071745.pfm) |
| lnc-MDK-4:2 | 6.6279899 | 100% | [MBNL1](http://rbpdb.ccbr.utoronto.ca/proteins.php?PME_sys_operation=PME_op_View&PME_sys_rec=1507) | 271 | 274 | UGCU | [669_20071745](http://rbpdb.ccbr.utoronto.ca/experiments.php?exp_id=669) | [Download PWM](http://rbpdb.ccbr.utoronto.ca/PWMDir/669_20071745.pwm) | [Download PFM](http://rbpdb.ccbr.utoronto.ca/PFMDir/669_20071745.pfm) |
| lnc-MDK-4:2 | 6.6279899 | 100% | [MBNL1](http://rbpdb.ccbr.utoronto.ca/proteins.php?PME_sys_operation=PME_op_View&PME_sys_rec=1507) | 134 | 137 | UGCU | [669_20071745](http://rbpdb.ccbr.utoronto.ca/experiments.php?exp_id=669) | [Download PWM](http://rbpdb.ccbr.utoronto.ca/PWMDir/669_20071745.pwm) | [Download PFM](http://rbpdb.ccbr.utoronto.ca/PFMDir/669_20071745.pfm) |
| lnc-MDK-4:2 | 6.4668404 | 100% | [EIF4B](http://rbpdb.ccbr.utoronto.ca/proteins.php?PME_sys_operation=PME_op_View&PME_sys_rec=1251) | 248 | 251 | GGAA | [352_8846295](http://rbpdb.ccbr.utoronto.ca/experiments.php?exp_id=352) | [Download PWM](http://rbpdb.ccbr.utoronto.ca/PWMDir/352_8846295.pwm) | [Download PFM](http://rbpdb.ccbr.utoronto.ca/PFMDir/352_8846295.pfm) |
| lnc-MDK-4:2 | 6.4668404 | 100% | [EIF4B](http://rbpdb.ccbr.utoronto.ca/proteins.php?PME_sys_operation=PME_op_View&PME_sys_rec=1251) | 357 | 360 | GGAA | [352_8846295](http://rbpdb.ccbr.utoronto.ca/experiments.php?exp_id=352) | [Download PWM](http://rbpdb.ccbr.utoronto.ca/PWMDir/352_8846295.pwm) | [Download PFM](http://rbpdb.ccbr.utoronto.ca/PFMDir/352_8846295.pfm) |
| lnc-MDK-4:2 | 6.33985 | 100% | [KHSRP](http://rbpdb.ccbr.utoronto.ca/proteins.php?PME_sys_operation=PME_op_View&PME_sys_rec=2661) | 256 | 259 | GUCC | [1186_17893325](http://rbpdb.ccbr.utoronto.ca/experiments.php?exp_id=1186) | [Download PWM](http://rbpdb.ccbr.utoronto.ca/PWMDir/1186_17893325.pwm) | [Download PFM](http://rbpdb.ccbr.utoronto.ca/PFMDir/1186_17893325.pfm) |
| lnc-MDK-4:2 | 6.33985 | 100% | [KHSRP](http://rbpdb.ccbr.utoronto.ca/proteins.php?PME_sys_operation=PME_op_View&PME_sys_rec=2661) | 511 | 514 | GUCC | [1186_17893325](http://rbpdb.ccbr.utoronto.ca/experiments.php?exp_id=1186) | [Download PWM](http://rbpdb.ccbr.utoronto.ca/PWMDir/1186_17893325.pwm) | [Download PFM](http://rbpdb.ccbr.utoronto.ca/PFMDir/1186_17893325.pfm) |
| lnc-MDK-4:2 | 6.33890598 | 100% | [YBX1](http://rbpdb.ccbr.utoronto.ca/proteins.php?PME_sys_operation=PME_op_View&PME_sys_rec=1257) | 77 | 82 | CCUGCG | [1177_19561594](http://rbpdb.ccbr.utoronto.ca/experiments.php?exp_id=1177) | [Download PWM](http://rbpdb.ccbr.utoronto.ca/PWMDir/1177_19561594.pwm) | [Download PFM](http://rbpdb.ccbr.utoronto.ca/PFMDir/1177_19561594.pfm) |
| lnc-MDK-4:2 | 5.830231484 | 82% | [SFRS9](http://rbpdb.ccbr.utoronto.ca/proteins.php?PME_sys_operation=PME_op_View&PME_sys_rec=1358) | 486 | 490 | AGCAC | [797_17548433](http://rbpdb.ccbr.utoronto.ca/experiments.php?exp_id=797) | [Download PWM](http://rbpdb.ccbr.utoronto.ca/PWMDir/797_17548433.pwm) | [Download PFM](http://rbpdb.ccbr.utoronto.ca/PFMDir/797_17548433.pfm) |
| lnc-MDK-4:2 | 5.830231484 | 82% | [SFRS9](http://rbpdb.ccbr.utoronto.ca/proteins.php?PME_sys_operation=PME_op_View&PME_sys_rec=1358) | 384 | 388 | AGCAC | [797_17548433](http://rbpdb.ccbr.utoronto.ca/experiments.php?exp_id=797) | [Download PWM](http://rbpdb.ccbr.utoronto.ca/PWMDir/797_17548433.pwm) | [Download PFM](http://rbpdb.ccbr.utoronto.ca/PFMDir/797_17548433.pfm) |
| lnc-MDK-4:2 | 5.2682554 | 100% | [RBMX](http://rbpdb.ccbr.utoronto.ca/proteins.php?PME_sys_operation=PME_op_View&PME_sys_rec=1487) | 332 | 335 | CCAG | [922_19282290](http://rbpdb.ccbr.utoronto.ca/experiments.php?exp_id=922) | [Download PWM](http://rbpdb.ccbr.utoronto.ca/PWMDir/922_19282290.pwm) | [Download PFM](http://rbpdb.ccbr.utoronto.ca/PFMDir/922_19282290.pfm) |
| lnc-MDK-4:2 | 5.2682554 | 100% | [RBMX](http://rbpdb.ccbr.utoronto.ca/proteins.php?PME_sys_operation=PME_op_View&PME_sys_rec=1487) | 382 | 385 | CCAG | [922_19282290](http://rbpdb.ccbr.utoronto.ca/experiments.php?exp_id=922) | [Download PWM](http://rbpdb.ccbr.utoronto.ca/PWMDir/922_19282290.pwm) | [Download PFM](http://rbpdb.ccbr.utoronto.ca/PFMDir/922_19282290.pfm) |
| lnc-MDK-4:2 | 5.2682554 | 100% | [RBMX](http://rbpdb.ccbr.utoronto.ca/proteins.php?PME_sys_operation=PME_op_View&PME_sys_rec=1487) | 28 | 31 | CCAG | [922_19282290](http://rbpdb.ccbr.utoronto.ca/experiments.php?exp_id=922) | [Download PWM](http://rbpdb.ccbr.utoronto.ca/PWMDir/922_19282290.pwm) | [Download PFM](http://rbpdb.ccbr.utoronto.ca/PFMDir/922_19282290.pfm) |
| lnc-MDK-4:2 | 5.2682554 | 100% | [RBMX](http://rbpdb.ccbr.utoronto.ca/proteins.php?PME_sys_operation=PME_op_View&PME_sys_rec=1487) | 115 | 118 | CCAG | [922_19282290](http://rbpdb.ccbr.utoronto.ca/experiments.php?exp_id=922) | [Download PWM](http://rbpdb.ccbr.utoronto.ca/PWMDir/922_19282290.pwm) | [Download PFM](http://rbpdb.ccbr.utoronto.ca/PFMDir/922_19282290.pfm) |
| lnc-MDK-4:2 | 5.2682554 | 100% | [RBMX](http://rbpdb.ccbr.utoronto.ca/proteins.php?PME_sys_operation=PME_op_View&PME_sys_rec=1487) | 92 | 95 | CCAG | [922_19282290](http://rbpdb.ccbr.utoronto.ca/experiments.php?exp_id=922) | [Download PWM](http://rbpdb.ccbr.utoronto.ca/PWMDir/922_19282290.pwm) | [Download PFM](http://rbpdb.ccbr.utoronto.ca/PFMDir/922_19282290.pfm) |
| lnc-MDK-4:2 | 5.2682554 | 100% | [RBMX](http://rbpdb.ccbr.utoronto.ca/proteins.php?PME_sys_operation=PME_op_View&PME_sys_rec=1487) | 45 | 48 | CCAG | [922_19282290](http://rbpdb.ccbr.utoronto.ca/experiments.php?exp_id=922) | [Download PWM](http://rbpdb.ccbr.utoronto.ca/PWMDir/922_19282290.pwm) | [Download PFM](http://rbpdb.ccbr.utoronto.ca/PFMDir/922_19282290.pfm) |
| lnc-MDK-4:2 | 5.2682554 | 100% | [RBMX](http://rbpdb.ccbr.utoronto.ca/proteins.php?PME_sys_operation=PME_op_View&PME_sys_rec=1487) | 552 | 555 | CCAG | [922_19282290](http://rbpdb.ccbr.utoronto.ca/experiments.php?exp_id=922) | [Download PWM](http://rbpdb.ccbr.utoronto.ca/PWMDir/922_19282290.pwm) | [Download PFM](http://rbpdb.ccbr.utoronto.ca/PFMDir/922_19282290.pfm) |
| lnc-MDK-4:2 | 5.2682554 | 100% | [RBMX](http://rbpdb.ccbr.utoronto.ca/proteins.php?PME_sys_operation=PME_op_View&PME_sys_rec=1487) | 229 | 232 | CCAG | [922_19282290](http://rbpdb.ccbr.utoronto.ca/experiments.php?exp_id=922) | [Download PWM](http://rbpdb.ccbr.utoronto.ca/PWMDir/922_19282290.pwm) | [Download PFM](http://rbpdb.ccbr.utoronto.ca/PFMDir/922_19282290.pfm) |
| lnc-MDK-4:2 | 5.2682554 | 100% | [RBMX](http://rbpdb.ccbr.utoronto.ca/proteins.php?PME_sys_operation=PME_op_View&PME_sys_rec=1487) | 278 | 281 | CCAG | [922_19282290](http://rbpdb.ccbr.utoronto.ca/experiments.php?exp_id=922) | [Download PWM](http://rbpdb.ccbr.utoronto.ca/PWMDir/922_19282290.pwm) | [Download PFM](http://rbpdb.ccbr.utoronto.ca/PFMDir/922_19282290.pfm) |
| lnc-MDK-4:2 | 5.2682554 | 100% | [RBMX](http://rbpdb.ccbr.utoronto.ca/proteins.php?PME_sys_operation=PME_op_View&PME_sys_rec=1487) | 67 | 70 | CCAG | [922_19282290](http://rbpdb.ccbr.utoronto.ca/experiments.php?exp_id=922) | [Download PWM](http://rbpdb.ccbr.utoronto.ca/PWMDir/922_19282290.pwm) | [Download PFM](http://rbpdb.ccbr.utoronto.ca/PFMDir/922_19282290.pfm) |
| lnc-MDK-4:2 | 5.256645851 | 81% | [EIF4B](http://rbpdb.ccbr.utoronto.ca/proteins.php?PME_sys_operation=PME_op_View&PME_sys_rec=1251) | 62 | 65 | GGAC | [352_8846295](http://rbpdb.ccbr.utoronto.ca/experiments.php?exp_id=352) | [Download PWM](http://rbpdb.ccbr.utoronto.ca/PWMDir/352_8846295.pwm) | [Download PFM](http://rbpdb.ccbr.utoronto.ca/PFMDir/352_8846295.pfm) |
| lnc-MDK-4:2 | 5.256645851 | 81% | [EIF4B](http://rbpdb.ccbr.utoronto.ca/proteins.php?PME_sys_operation=PME_op_View&PME_sys_rec=1251) | 457 | 460 | GGAC | [352_8846295](http://rbpdb.ccbr.utoronto.ca/experiments.php?exp_id=352) | [Download PWM](http://rbpdb.ccbr.utoronto.ca/PWMDir/352_8846295.pwm) | [Download PFM](http://rbpdb.ccbr.utoronto.ca/PFMDir/352_8846295.pfm) |
| lnc-MDK-4:2 | 5.256645851 | 81% | [EIF4B](http://rbpdb.ccbr.utoronto.ca/proteins.php?PME_sys_operation=PME_op_View&PME_sys_rec=1251) | 188 | 191 | GGAC | [352_8846295](http://rbpdb.ccbr.utoronto.ca/experiments.php?exp_id=352) | [Download PWM](http://rbpdb.ccbr.utoronto.ca/PWMDir/352_8846295.pwm) | [Download PFM](http://rbpdb.ccbr.utoronto.ca/PFMDir/352_8846295.pfm) |
| lnc-MDK-4:2 | 5.256645851 | 81% | [EIF4B](http://rbpdb.ccbr.utoronto.ca/proteins.php?PME_sys_operation=PME_op_View&PME_sys_rec=1251) | 7 | 10 | GGAC | [352_8846295](http://rbpdb.ccbr.utoronto.ca/experiments.php?exp_id=352) | [Download PWM](http://rbpdb.ccbr.utoronto.ca/PWMDir/352_8846295.pwm) | [Download PFM](http://rbpdb.ccbr.utoronto.ca/PFMDir/352_8846295.pfm) |
| lnc-MDK-4:2 | 5.256645851 | 81% | [EIF4B](http://rbpdb.ccbr.utoronto.ca/proteins.php?PME_sys_operation=PME_op_View&PME_sys_rec=1251) | 517 | 520 | GGAC | [352_8846295](http://rbpdb.ccbr.utoronto.ca/experiments.php?exp_id=352) | [Download PWM](http://rbpdb.ccbr.utoronto.ca/PWMDir/352_8846295.pwm) | [Download PFM](http://rbpdb.ccbr.utoronto.ca/PFMDir/352_8846295.pfm) |
| lnc-MDK-4:2 | 4.99861593 | 94% | [RBMX](http://rbpdb.ccbr.utoronto.ca/proteins.php?PME_sys_operation=PME_op_View&PME_sys_rec=1487) | 328 | 331 | CCAU | [922_19282290](http://rbpdb.ccbr.utoronto.ca/experiments.php?exp_id=922) | [Download PWM](http://rbpdb.ccbr.utoronto.ca/PWMDir/922_19282290.pwm) | [Download PFM](http://rbpdb.ccbr.utoronto.ca/PFMDir/922_19282290.pfm) |
| lnc-MDK-4:2 | 4.99861593 | 94% | [RBMX](http://rbpdb.ccbr.utoronto.ca/proteins.php?PME_sys_operation=PME_op_View&PME_sys_rec=1487) | 202 | 205 | CCAU | [922_19282290](http://rbpdb.ccbr.utoronto.ca/experiments.php?exp_id=922) | [Download PWM](http://rbpdb.ccbr.utoronto.ca/PWMDir/922_19282290.pwm) | [Download PFM](http://rbpdb.ccbr.utoronto.ca/PFMDir/922_19282290.pfm) |
| lnc-MDK-4:2 | 4.99861593 | 94% | [RBMX](http://rbpdb.ccbr.utoronto.ca/proteins.php?PME_sys_operation=PME_op_View&PME_sys_rec=1487) | 363 | 366 | CCAU | [922_19282290](http://rbpdb.ccbr.utoronto.ca/experiments.php?exp_id=922) | [Download PWM](http://rbpdb.ccbr.utoronto.ca/PWMDir/922_19282290.pwm) | [Download PFM](http://rbpdb.ccbr.utoronto.ca/PFMDir/922_19282290.pfm) |
| lnc-MDK-4:2 | 7.08652094 | 100% | [SFRS9](http://rbpdb.ccbr.utoronto.ca/proteins.php?PME_sys_operation=PME_op_View&PME_sys_rec=1358) | 6 | 10 | AGGAC | [797_17548433](http://rbpdb.ccbr.utoronto.ca/experiments.php?exp_id=797) | [Download PWM](http://rbpdb.ccbr.utoronto.ca/PWMDir/797_17548433.pwm) | [Download PFM](http://rbpdb.ccbr.utoronto.ca/PFMDir/797_17548433.pfm) |
| lnc-MDK-4:2 | 4.687504219 | 91% | [SFRS13A](http://rbpdb.ccbr.utoronto.ca/proteins.php?PME_sys_operation=PME_op_View&PME_sys_rec=1649) | 251 | 257 | AAAGGGU | [1169_19561594](http://rbpdb.ccbr.utoronto.ca/experiments.php?exp_id=1169) | [Download PWM](http://rbpdb.ccbr.utoronto.ca/PWMDir/1169_19561594.pwm) | [Download PFM](http://rbpdb.ccbr.utoronto.ca/PFMDir/1169_19561594.pfm) |
| lnc-MDK-4:2 | 4.6667232 | 88% | [RBMX](http://rbpdb.ccbr.utoronto.ca/proteins.php?PME_sys_operation=PME_op_View&PME_sys_rec=1487) | 342 | 345 | CCAC | [922_19282290](http://rbpdb.ccbr.utoronto.ca/experiments.php?exp_id=922) | [Download PWM](http://rbpdb.ccbr.utoronto.ca/PWMDir/922_19282290.pwm) | [Download PFM](http://rbpdb.ccbr.utoronto.ca/PFMDir/922_19282290.pfm) |
| lnc-MDK-4:2 | 4.6667232 | 88% | [RBMX](http://rbpdb.ccbr.utoronto.ca/proteins.php?PME_sys_operation=PME_op_View&PME_sys_rec=1487) | 74 | 77 | CCAC | [922_19282290](http://rbpdb.ccbr.utoronto.ca/experiments.php?exp_id=922) | [Download PWM](http://rbpdb.ccbr.utoronto.ca/PWMDir/922_19282290.pwm) | [Download PFM](http://rbpdb.ccbr.utoronto.ca/PFMDir/922_19282290.pfm) |
| lnc-MDK-4:2 | 4.6667232 | 88% | [RBMX](http://rbpdb.ccbr.utoronto.ca/proteins.php?PME_sys_operation=PME_op_View&PME_sys_rec=1487) | 199 | 202 | CCAC | [922_19282290](http://rbpdb.ccbr.utoronto.ca/experiments.php?exp_id=922) | [Download PWM](http://rbpdb.ccbr.utoronto.ca/PWMDir/922_19282290.pwm) | [Download PFM](http://rbpdb.ccbr.utoronto.ca/PFMDir/922_19282290.pfm) |
| lnc-MDK-4:2 | 4.6667232 | 88% | [RBMX](http://rbpdb.ccbr.utoronto.ca/proteins.php?PME_sys_operation=PME_op_View&PME_sys_rec=1487) | 405 | 408 | CCAC | [922_19282290](http://rbpdb.ccbr.utoronto.ca/experiments.php?exp_id=922) | [Download PWM](http://rbpdb.ccbr.utoronto.ca/PWMDir/922_19282290.pwm) | [Download PFM](http://rbpdb.ccbr.utoronto.ca/PFMDir/922_19282290.pfm) |
| lnc-MDK-4:2 | 5.7148754 | 91% | [YTHDC1](http://rbpdb.ccbr.utoronto.ca/proteins.php?PME_sys_operation=PME_op_View&PME_sys_rec=1283) | 448 | 453 | GAGUAC | [969_20167602](http://rbpdb.ccbr.utoronto.ca/experiments.php?exp_id=969) | [Download PWM](http://rbpdb.ccbr.utoronto.ca/PWMDir/969_20167602.pwm) | [Download PFM](http://rbpdb.ccbr.utoronto.ca/PFMDir/969_20167602.pfm) |
| lnc-MDK-4:2 | 4.62028767 | 100% | [SFRS1](http://rbpdb.ccbr.utoronto.ca/proteins.php?PME_sys_operation=PME_op_View&PME_sys_rec=1448) | 356 | 359 | AGGA | [1173_19561594](http://rbpdb.ccbr.utoronto.ca/experiments.php?exp_id=1173) | [Download PWM](http://rbpdb.ccbr.utoronto.ca/PWMDir/1173_19561594.pwm) | [Download PFM](http://rbpdb.ccbr.utoronto.ca/PFMDir/1173_19561594.pfm) |
| lnc-MDK-4:2 | 4.62028767 | 100% | [SFRS1](http://rbpdb.ccbr.utoronto.ca/proteins.php?PME_sys_operation=PME_op_View&PME_sys_rec=1448) | 554 | 557 | AGGA | [1173_19561594](http://rbpdb.ccbr.utoronto.ca/experiments.php?exp_id=1173) | [Download PWM](http://rbpdb.ccbr.utoronto.ca/PWMDir/1173_19561594.pwm) | [Download PFM](http://rbpdb.ccbr.utoronto.ca/PFMDir/1173_19561594.pfm) |
| lnc-MDK-4:2 | 4.62028767 | 100% | [SFRS1](http://rbpdb.ccbr.utoronto.ca/proteins.php?PME_sys_operation=PME_op_View&PME_sys_rec=1448) | 6 | 9 | AGGA | [1173_19561594](http://rbpdb.ccbr.utoronto.ca/experiments.php?exp_id=1173) | [Download PWM](http://rbpdb.ccbr.utoronto.ca/PWMDir/1173_19561594.pwm) | [Download PFM](http://rbpdb.ccbr.utoronto.ca/PFMDir/1173_19561594.pfm) |
| lnc-MDK-4:2 | 4.62028767 | 100% | [SFRS1](http://rbpdb.ccbr.utoronto.ca/proteins.php?PME_sys_operation=PME_op_View&PME_sys_rec=1448) | 215 | 218 | AGGA | [1173_19561594](http://rbpdb.ccbr.utoronto.ca/experiments.php?exp_id=1173) | [Download PWM](http://rbpdb.ccbr.utoronto.ca/PWMDir/1173_19561594.pwm) | [Download PFM](http://rbpdb.ccbr.utoronto.ca/PFMDir/1173_19561594.pfm) |
| lnc-MDK-4:2 | 4.751281996 | 92% | [SFRS13A](http://rbpdb.ccbr.utoronto.ca/proteins.php?PME_sys_operation=PME_op_View&PME_sys_rec=1649) | 105 | 111 | AAAGGGG | [1169_19561594](http://rbpdb.ccbr.utoronto.ca/experiments.php?exp_id=1169) | [Download PWM](http://rbpdb.ccbr.utoronto.ca/PWMDir/1169_19561594.pwm) | [Download PFM](http://rbpdb.ccbr.utoronto.ca/PFMDir/1169_19561594.pfm) |
| lnc-MDK-4:2 | 4.40271173 | 83% | [RBMX](http://rbpdb.ccbr.utoronto.ca/proteins.php?PME_sys_operation=PME_op_View&PME_sys_rec=1487) | 32 | 35 | CCCG | [922_19282290](http://rbpdb.ccbr.utoronto.ca/experiments.php?exp_id=922) | [Download PWM](http://rbpdb.ccbr.utoronto.ca/PWMDir/922_19282290.pwm) | [Download PFM](http://rbpdb.ccbr.utoronto.ca/PFMDir/922_19282290.pfm) |
| lnc-MDK-4:2 | 4.40271173 | 83% | [RBMX](http://rbpdb.ccbr.utoronto.ca/proteins.php?PME_sys_operation=PME_op_View&PME_sys_rec=1487) | 352 | 355 | CCCG | [922_19282290](http://rbpdb.ccbr.utoronto.ca/experiments.php?exp_id=922) | [Download PWM](http://rbpdb.ccbr.utoronto.ca/PWMDir/922_19282290.pwm) | [Download PFM](http://rbpdb.ccbr.utoronto.ca/PFMDir/922_19282290.pfm) |
| lnc-MDK-4:2 | 4.40271173 | 83% | [RBMX](http://rbpdb.ccbr.utoronto.ca/proteins.php?PME_sys_operation=PME_op_View&PME_sys_rec=1487) | 418 | 421 | CCCG | [922_19282290](http://rbpdb.ccbr.utoronto.ca/experiments.php?exp_id=922) | [Download PWM](http://rbpdb.ccbr.utoronto.ca/PWMDir/922_19282290.pwm) | [Download PFM](http://rbpdb.ccbr.utoronto.ca/PFMDir/922_19282290.pfm) |
| lnc-MDK-4:2 | 4.40271173 | 83% | [RBMX](http://rbpdb.ccbr.utoronto.ca/proteins.php?PME_sys_operation=PME_op_View&PME_sys_rec=1487) | 261 | 264 | CCCG | [922_19282290](http://rbpdb.ccbr.utoronto.ca/experiments.php?exp_id=922) | [Download PWM](http://rbpdb.ccbr.utoronto.ca/PWMDir/922_19282290.pwm) | [Download PFM](http://rbpdb.ccbr.utoronto.ca/PFMDir/922_19282290.pfm) |
| lnc-MDK-4:2 | 4.40271173 | 83% | [RBMX](http://rbpdb.ccbr.utoronto.ca/proteins.php?PME_sys_operation=PME_op_View&PME_sys_rec=1487) | 51 | 54 | CCCG | [922_19282290](http://rbpdb.ccbr.utoronto.ca/experiments.php?exp_id=922) | [Download PWM](http://rbpdb.ccbr.utoronto.ca/PWMDir/922_19282290.pwm) | [Download PFM](http://rbpdb.ccbr.utoronto.ca/PFMDir/922_19282290.pfm) |
| lnc-MDK-4:2 | 4.40271173 | 83% | [RBMX](http://rbpdb.ccbr.utoronto.ca/proteins.php?PME_sys_operation=PME_op_View&PME_sys_rec=1487) | 191 | 194 | CCCG | [922_19282290](http://rbpdb.ccbr.utoronto.ca/experiments.php?exp_id=922) | [Download PWM](http://rbpdb.ccbr.utoronto.ca/PWMDir/922_19282290.pwm) | [Download PFM](http://rbpdb.ccbr.utoronto.ca/PFMDir/922_19282290.pfm) |
| lnc-MDK-4:2 | 4.365802746 | 85% | [SFRS13A](http://rbpdb.ccbr.utoronto.ca/proteins.php?PME_sys_operation=PME_op_View&PME_sys_rec=1649) | 20 | 26 | AGAGGCC | [1169_19561594](http://rbpdb.ccbr.utoronto.ca/experiments.php?exp_id=1169) | [Download PWM](http://rbpdb.ccbr.utoronto.ca/PWMDir/1169_19561594.pwm) | [Download PFM](http://rbpdb.ccbr.utoronto.ca/PFMDir/1169_19561594.pfm) |
| lnc-MDK-4:2 | 4.1881759 | 90% | [SFRS1](http://rbpdb.ccbr.utoronto.ca/proteins.php?PME_sys_operation=PME_op_View&PME_sys_rec=1448) | 461 | 464 | UGGA | [1173_19561594](http://rbpdb.ccbr.utoronto.ca/experiments.php?exp_id=1173) | [Download PWM](http://rbpdb.ccbr.utoronto.ca/PWMDir/1173_19561594.pwm) | [Download PFM](http://rbpdb.ccbr.utoronto.ca/PFMDir/1173_19561594.pfm) |
| lnc-MDK-4:2 | 4.1881759 | 90% | [SFRS1](http://rbpdb.ccbr.utoronto.ca/proteins.php?PME_sys_operation=PME_op_View&PME_sys_rec=1448) | 247 | 250 | UGGA | [1173_19561594](http://rbpdb.ccbr.utoronto.ca/experiments.php?exp_id=1173) | [Download PWM](http://rbpdb.ccbr.utoronto.ca/PWMDir/1173_19561594.pwm) | [Download PFM](http://rbpdb.ccbr.utoronto.ca/PFMDir/1173_19561594.pfm) |
| lnc-MDK-4:2 | 4.1881759 | 90% | [SFRS1](http://rbpdb.ccbr.utoronto.ca/proteins.php?PME_sys_operation=PME_op_View&PME_sys_rec=1448) | 40 | 43 | UGGA | [1173_19561594](http://rbpdb.ccbr.utoronto.ca/experiments.php?exp_id=1173) | [Download PWM](http://rbpdb.ccbr.utoronto.ca/PWMDir/1173_19561594.pwm) | [Download PFM](http://rbpdb.ccbr.utoronto.ca/PFMDir/1173_19561594.pfm) |
| lnc-MDK-4:2 | 4.1881759 | 90% | [SFRS1](http://rbpdb.ccbr.utoronto.ca/proteins.php?PME_sys_operation=PME_op_View&PME_sys_rec=1448) | 177 | 180 | UGGA | [1173_19561594](http://rbpdb.ccbr.utoronto.ca/experiments.php?exp_id=1173) | [Download PWM](http://rbpdb.ccbr.utoronto.ca/PWMDir/1173_19561594.pwm) | [Download PFM](http://rbpdb.ccbr.utoronto.ca/PFMDir/1173_19561594.pfm) |
| lnc-MDK-4:2 | 4.1881759 | 90% | [SFRS1](http://rbpdb.ccbr.utoronto.ca/proteins.php?PME_sys_operation=PME_op_View&PME_sys_rec=1448) | 456 | 459 | UGGA | [1173_19561594](http://rbpdb.ccbr.utoronto.ca/experiments.php?exp_id=1173) | [Download PWM](http://rbpdb.ccbr.utoronto.ca/PWMDir/1173_19561594.pwm) | [Download PFM](http://rbpdb.ccbr.utoronto.ca/PFMDir/1173_19561594.pfm) |
| lnc-MDK-4:2 | 4.142143128 | 89% | [KHDRBS3](http://rbpdb.ccbr.utoronto.ca/proteins.php?PME_sys_operation=PME_op_View&PME_sys_rec=1423) | 296 | 301 | ACUAAA | [1174_19561594](http://rbpdb.ccbr.utoronto.ca/experiments.php?exp_id=1174) | [Download PWM](http://rbpdb.ccbr.utoronto.ca/PWMDir/1174_19561594.pwm) | [Download PFM](http://rbpdb.ccbr.utoronto.ca/PFMDir/1174_19561594.pfm) |

^a^ Click to detail them in the open corresponding web page (RBPDB site: http://rbpdb.ccbr.utoronto.ca/index.php) .

**Supplemental Figures**


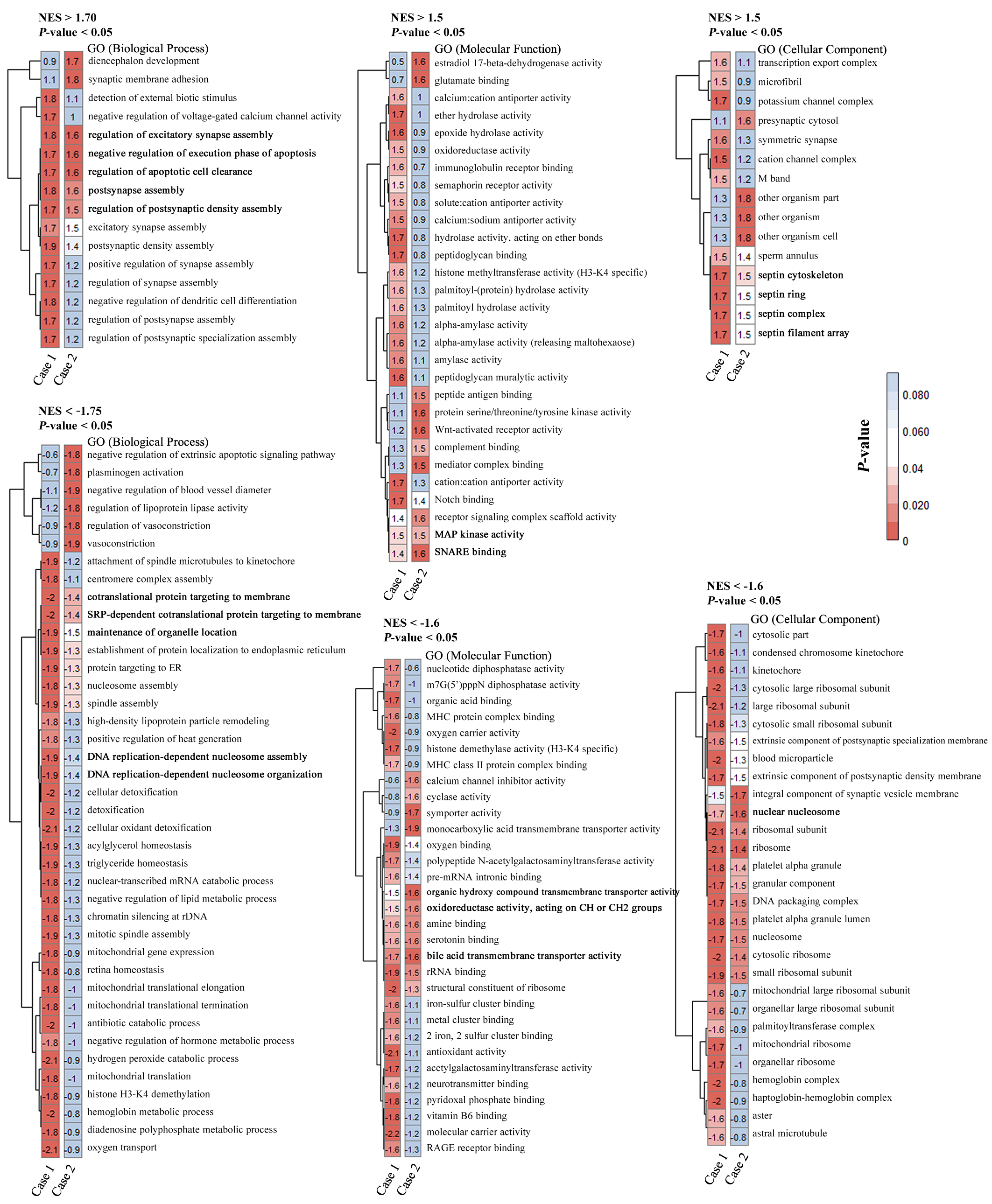


**Figure S2** Comparison of GO function enrichments identified by GSEA method in two case population.

**Note**: GSEA, gene set enrichment analyses; NES, normalized enrichment score; Case 1, qi deficiency constitution; Case 2, Pi-qi-deficiency syndrome of chronic superficial gastritis.


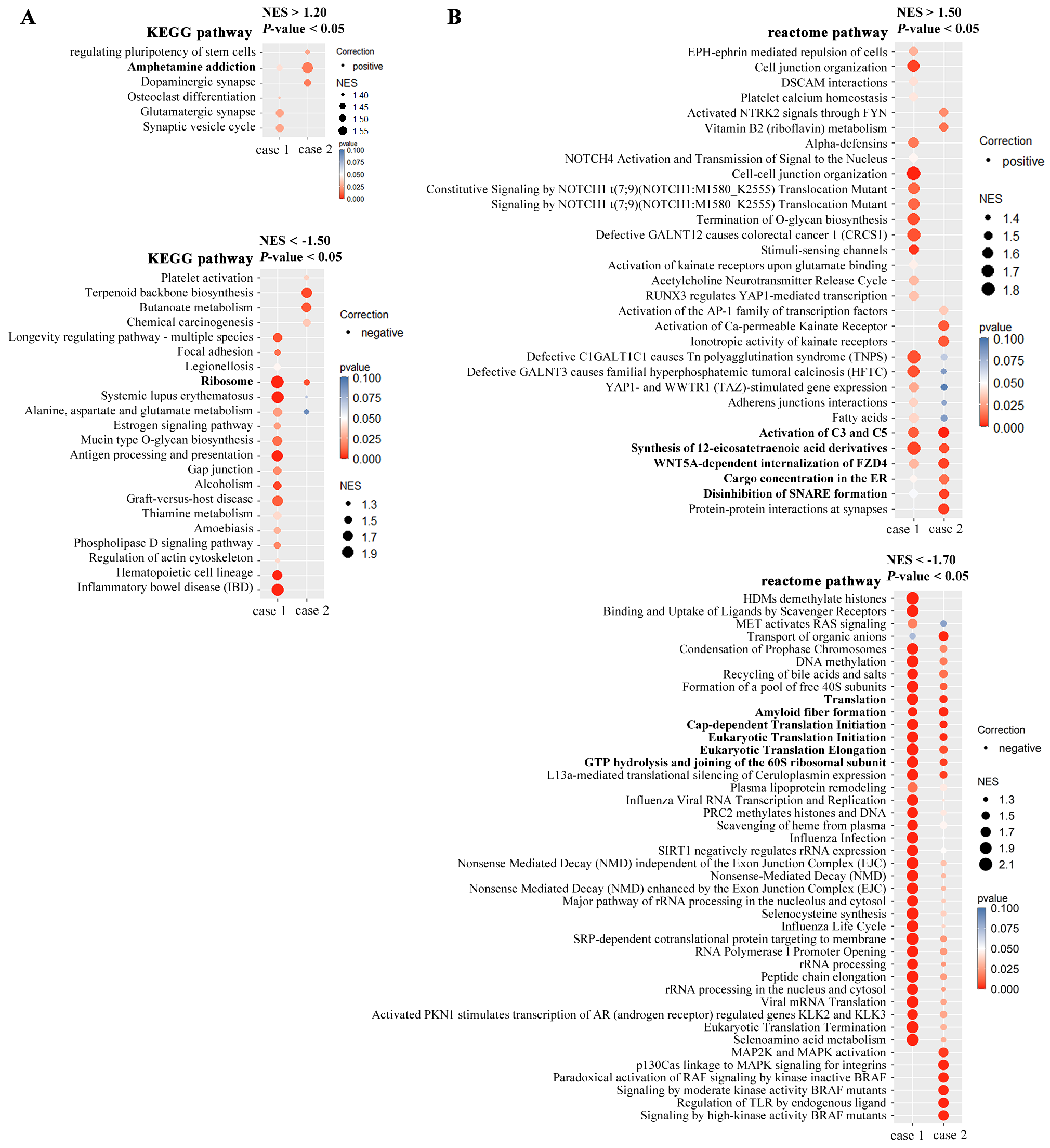


**Figure S3** Comparison of pathway enrichments identified by GSEA method in two case population.

(**A**) Comparison of KEGG pathway enrichment identified by GSEA in case population 1. (**B**) Comparison of Reactome pathway enrichment identified by GSEA in case population 2.

**Note**: GSEA, gene set enrichment analyses; NES, normalized enrichment score; Case 1, qi deficiency constitution; Case 2, Pi-qi-deficiency syndrome of chronic superficial gastritis.


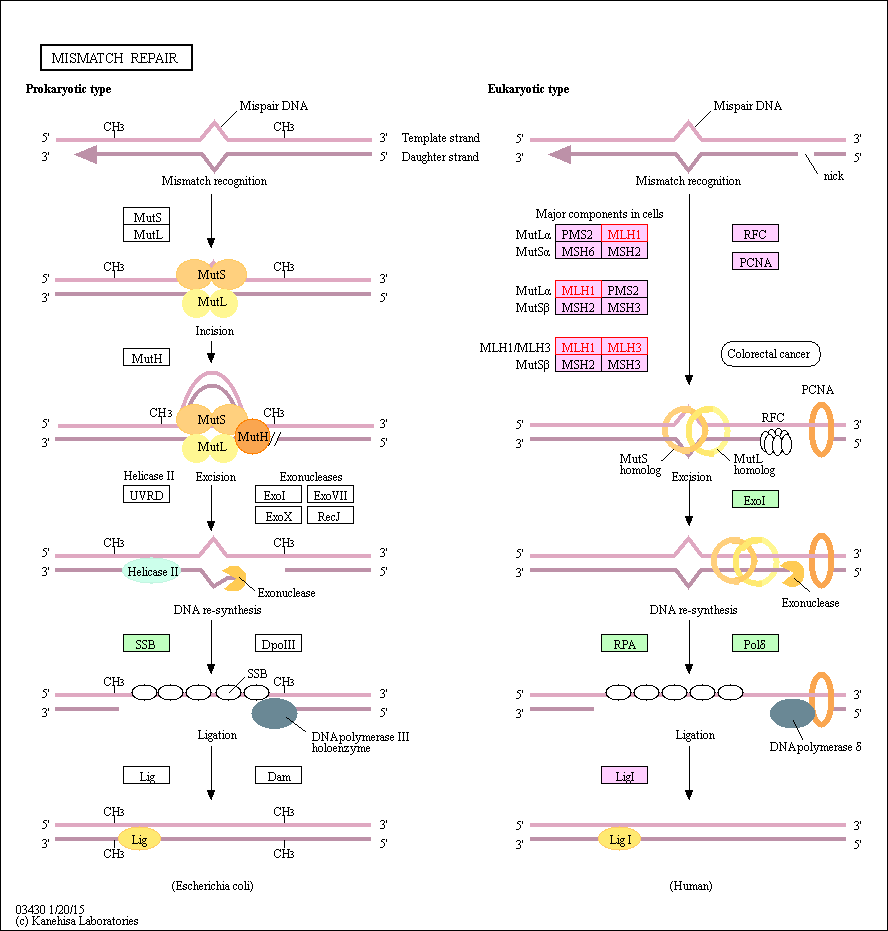


**Figure S4** Mismatch repair pathway (hsadd03430)


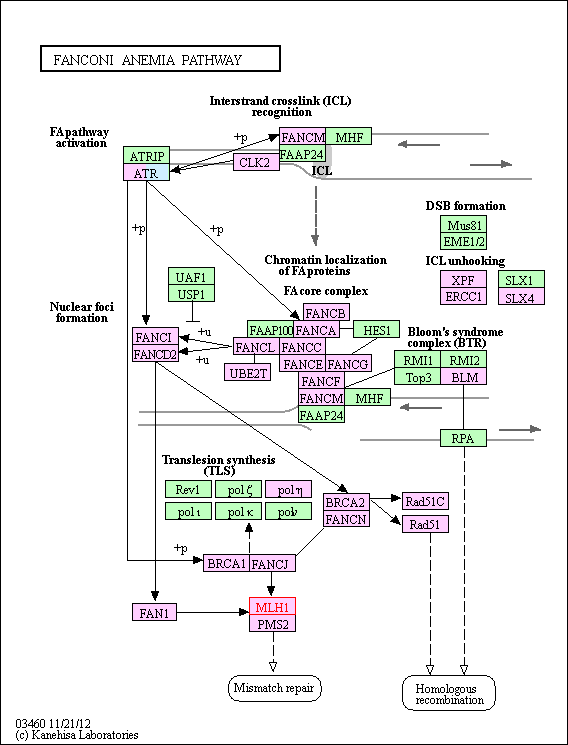


**Figure S5** Fanconi anemia pathway (hsadd03460)

**References**

1. China Association of Chinese Medicine, *Classification and Determination of Constitution in Traditional Chinese Medicine*. China Press of Traditional Chinese Medicine, Beijing, 1 edition, 2009.

2. Y.B. Zhu, Q. Wang, and H. Origasa. "Evaluation on reliability and validity of the constitution in Chinese medicine questionnaire (CCMQ)," *Chinese Journal of Behavioral Medical Science, vol.* 16, no. 7, pp. 651–654.

3. Q. Wang and Y.B. Zhu. "Epidemiological investigation of constitution types of Chinese medicine in general population: base on 21, 948 epidemiological investigation data of nine provinces in China," *China Journal of Traditional Chinese Medicine and Pharmacy, vol.* 24, no. 1, pp. 7-12.

4. J.Y. Fang, W.Z. Liu, Z.K. Li, et al. "China Chronic Gastritis Consensus (2012, Shanghai)," *Chin J Front Med Sci, vol.* 5, no. 07, pp. 44-55.

5. X.Y. Zheng, *Guiding Principle for Clinical Research on New Drugs of Traditional Chinese Medicine*. China Medical Science Press, Beijing, 1 edition, 2002.

6. F. Alkan, A. Wenzel, O. Palasca, et al. "RIsearch2: suffix array-based large-scale prediction of RNA-RNA interactions and siRNA off-targets," *Nucleic Acids Res, vol.* 45, no. 8, p. e60.

7. U.K. Muppirala, V.G. Honavar, and D. Dobbs. "Predicting RNA-protein interactions using only sequence information," *BMC Bioinformatics, vol.* 12, p. 489.

8. K.B. Cook, H. Kazan, K. Zuberi, Q. Morris, and T.R. Hughes. "RBPDB: a database of RNA-binding specificities," *Nucleic Acids Res, vol.* 39, no. Database issue, pp. D301-308.
